# Finding New Order in Biological Functions from the Network Structure of Gene Annotations

**DOI:** 10.1371/journal.pcbi.1004565

**Published:** 2015-11-20

**Authors:** Kimberly Glass, Michelle Girvan

**Affiliations:** 1 Department of Biostatistics and Computational Biology, Dana-Farber Cancer Institute and Harvard T. H. Chan School of Public Health, Boston, Massachusetts, United States of America; 2 Channing Division of Network Medicine, Department of Medicine, Brigham and Women’s Hospital and Harvard Medical School, Boston, Massachusetts, United States of America; 3 Physics Department, University of Maryland, College Park, Maryland, United States of America; 4 Institute for Physical Science and Technology, University of Maryland, College Park, Maryland, United States of America; 5 Santa Fe Institute, Santa Fe, New Mexico, United States of America; University of California San Diego, UNITED STATES

## Abstract

The Gene Ontology (GO) provides biologists with a controlled terminology that describes how genes are associated with functions and how functional terms are related to one another. These term-term relationships encode how scientists conceive the organization of biological functions, and they take the form of a directed acyclic graph (DAG). Here, we propose that the network structure of gene-term annotations made using GO can be employed to establish an alternative approach for grouping functional terms that captures intrinsic functional relationships that are not evident in the hierarchical structure established in the GO DAG. Instead of relying on an externally defined organization for biological functions, our approach connects biological functions together if they are performed by the same genes, as indicated in a compendium of gene annotation data from numerous different sources. We show that grouping terms by this alternate scheme provides a new framework with which to describe and predict the functions of experimentally identified sets of genes.

## Author Summary

Investigating how a set of genes might collectively work together to perform various cellular processes has become a routine part of many biological analyses. In such analyses, genes of interest are compared to sets of genes annotated to various biological functions (or pathways) defined within carefully curated databases. One of the most comprehensive and widely used resources of this type is the Gene Ontology (GO) database. The Gene Ontology database is comprised of two important elements: (1) the ontology itself, which provides a controlled vocabulary of terms describing genetic function and also specifies how these functional terms are related to one another via a hierarchical structure; and (2) the set of annotations made using GO that connect individual genes to different functional terms. In our paper we investigate a method for organizing functional terms that results from connecting terms based on shared gene annotations. We find that this alternate classification has an organization that is highly distinct from the Gene Ontology hierarchy, challenging the way we think about the relationships between different biological functions. Finally, we show that these alternate collections of terms are highly associated with published cancer gene signatures, demonstrating that this alternative organization of biological functions can highlight important relationships between cellular processes and has the potential to lead to new insights and discoveries.

## Introduction

The Gene Ontology (GO) [1][2] has been around for over a decade, during which time it has been widely used both to validate and to predict the results of biological experiments (see, for example [3–9]). The structure of the ontology, in which different functional “categories” or terms are related to each other in a hierarchical fashion, provides a well-established format with which to classify and subclassify all biological functions and processes. This classification approach is well-structured and well-characterized. However, we seek to determine if there is an alternate method for organizing biological functions that may in some instances be more biologically relevant or lead to important new insights. We focus on two main questions. First, can we use the information encoded in gene annotations (which report the relationships between individual genes and functional terms, and are derived from various sources of evidence) to identify an alternate organization for biological functions? Secondly, if such an alternate classification exists, how can it be used to interpret biological data?

In order to answer our first question, we link functional terms together if they are performed by many of the same genes, creating a *complex network* of term-term relationships. We point out that although many researchers have investigated relationships between GO terms, previous studies have focused on quantifying the similarity between biological functions using the distance between terms in the ontology [10] and/or their semantic similarity as derived from ancestor terms [11, 12], using functional relationships for improving gene set analysis [13] or for protein function prediction [14, 15], discovering and incorporating links between functional terms that were not previously in the Gene Ontology [5, 16, 17] and even building data-driven ontologies by combining annotations made using GO with empirical data on gene interactions [18]. By contrast, in this paper we focus on the *network structure* of the term-term relationships that result solely from shared annotations. In doing so, our method identifies an alternate organization of biological terms that is largely distinct from the ontological organization of GO.

In recent years, complex networks tools have been used alongside traditional bioinformatics techniques to study many different kinds of biological networks [19], including, but not limited to, gene regulatory networks [20, 21], protein-protein interaction networks [22, 23], and metabolic networks [24, 25]. Developments in network theory provide the computational tools needed to calculate the global properties of these networks, lending insights into the behavior of the systems they represent. For example, many networks exhibit community structure, meaning that there are clusters of nodes in the network within which edges are relatively dense [26]. Within the field of complex networks, many recent papers [27–33] have focused on methods to detect such modules in various types of networks in a computationally efficient and accurate manner. In this study, we leverage the community structure in gene annotation networks to develop an endogenous organization of biological functions.

**Fig 1:**
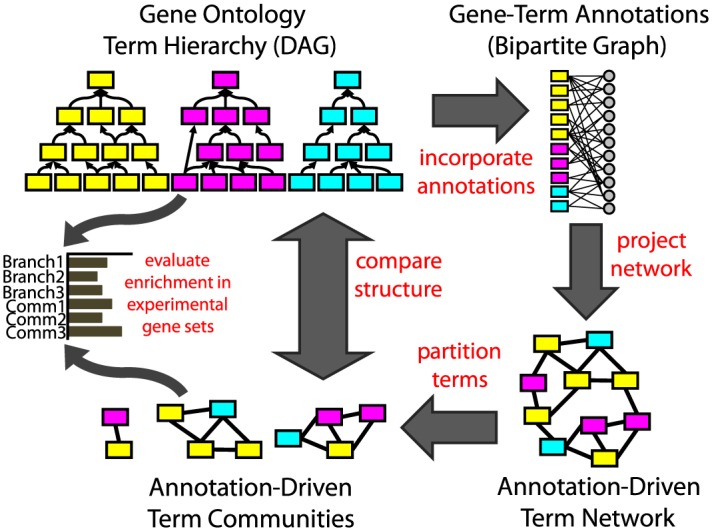
Visual Representation of Our Approach. First, we summarize gene annotations made to functional terms in the Gene Ontology hierarchy as a gene-term bipartite graph. From these gene-term relationships, we project a term-term network. We partition this network into communities and compare those term communities to branches of terms in the DAG. Finally, we perform functional enrichment analysis on experimentally-defined gene sets using both the term communities and GO branches.

Our complex networks approach to organizing biological functions using annotations made to the Gene Ontology is outlined in Fig 1. We begin by considering term relationships defined by the GO hierarchy. We then add in gene-term annotation information collected from different evidence sources and encapsulate these connections in the form of a bipartite network. Next, we use this bipartite network of gene-term relationships to construct another network describing connections between functional terms based on shared gene annotations. We apply community structure finding algorithms to partition this annotation-driven network into communities of terms and compare these communities to branches (ontological groupings of terms) from the GO hierarchy. We show that, although there are some similarities, there are also very strong differences between the two ways of organizing terms. Finally, we test the applicability of the community-derived classification, using functional analysis techniques to evaluate the enrichment of cancer signatures (sets of genes associated with cancer) in both term communities and GO branches. We find that certain signatures are enriched primarily in our term communities and not GO branches. Therefore, we suggest that by linking functional terms based on shared genes, we can create an alternate, biologically meaningful, network-derived organization of terms that is both distinct from the GO DAG and can also be used to investigate biological systems. We emphasize that our goal is not to supplant the traditional use of GO but rather to offer an alternate organization for biological functions that may in some cases provide important additional insights into the functional enrichment of experimentally derived gene sets.

The annotation files and code needed to reproduce the analysis and figures in this manuscript are included in the Supplemental Material (S1 Code). This information, as well as all intermediate and output data-files, can also be downloaded from [34].

## Methods

### Characterizing Gene Ontology Annotations in a Bipartite Graph

The Gene Ontology describes the relationships between different biological concepts or functions [1]. It breaks these concepts into three distinct ontologies, or primary domains: “Biological Process” (BP), describing sets of molecular events, “Molecular Function” (MF), describing the activities of gene products, and “Cellular Component” (CC), describing parts of a cell or its external environment. Each of the three primary domains in GO takes the form of a directed acyclic graph (DAG), in which “child” functional categories, or “terms”, are subclassified under one or more “parent” terms. Terms in the GO hierarchy can then be grouped into multiple overlapping sets called “branches,” with each individual branch corresponding to a parent term and all of its descendants. Using GO, genes are annotated to individual terms representing their particular role in a cell, and these annotations are transitive up the relationships in the DAG such that each “parent” term takes on all the gene annotations associated with any of its progeny [35].

In the following analysis we explore if there exists an alternate, annotation-driven way to classify terms that is distinct from this ontology structure. To begin, we use term-term ontology relationships and gene-term annotation information for human genes downloaded from the GO website (geneontology.org; access date: May 28, 2015) to construct a gene-term bipartite network. We choose to represent this network in the form of an *n*_*G*_ × *n*_*T*_ adjacency matrix, where *n*_*G*_ is the total number of genes and *n*_*T*_ is the total number of terms. In this matrix a value of one indicates a known connection between the corresponding gene and term, and a value of zero indicates that the gene is not associated with that term. Thus,

$$\begin{array}{c} B_{pi}=\{\begin{array}{cc} 1 & \text{if}\;\text{gene}\;p\;\text{is}\;\text{annotated}\;\text{to}\;\text{term}\;i\\ 0 & \text{if}\;\text{gene}\;p\;\text{is}\;\text{not}\;\text{annotated}\;\text{to}\;\text{term}\;i \end{array}. \end{array}$$ (1)

Because annotations are transitive, edges in *B* will not only extend from a gene to its annotated term, but also from that gene to all the term’s ancestors (parents, parents of parents, etc.) in the GO DAG.

The bipartite network described by *B* represents a summary of the relationships between 19329 human genes and 19403 functional terms, derived from many different types of biological evidence and contributed to by multiple laboratories [36]. We note that GO is divided into three primary domains and gene-annotations are made to the ontology for many species. However, for simplicity in the following analysis we combine information from all three domains and use annotation information only that pertains to human genes. Domain-specific and comparative species analysis is provided in the Supplemental Material (S1 Text).

### Constructing a Term Network from Gene Ontology Annotations

Next, we used gene-term annotations to construct a network representing term-term relationships. Using the bipartite network Eq (1) one could create a term network by simply joining together any pair of terms that share common genes; however, the number of genes annotated to each term has a heavy-tailed distribution [37, 38], thus this approach would lose a large amount of information as connections between pairs of terms with many genes annotated to them would be given the same weight as connections between pairs of terms that only have few gene annotations. We correct for the skewed term degree distribution by constructing a diagonal weighting matrix, *w*, and then projecting a term network *T*, whose edges are modified by this weighting matrix:

$$\begin{array}{c} w_{ij}=\frac{\delta (i,j)}{\sum_{q=1}^{n_{G}}B_{qi}},\;T=w^{\prime }B^{\prime }Bw, \end{array}$$ (2)

where *δ*(*i*, *j*) is the Kronecker delta function and takes a value of one when *i* = *j* and zero otherwise. The values of *T*_*ij*_ take a maximum value of one when terms *i* and *j* each only have the same single gene annotation and a minimum value of zero when none of the genes annotated to term *i* are annotated to term *j*. We note that because every parent term takes on all of the annotations of each its children, *T*_*ij*_ will necessarily be nonzero for every parent-child pair of terms. However, the weight of these relationship can be very low (this is especially likely when the parent has a large number of annotations). In other words, the use of the weighting matrix serves to accentuate relationships between low degree terms. Since these terms represent biological functions performed by only a handful of genes, we believe this weighting is more likely to capture highly-specific shared biological information.

We also note that because we are using gene annotations to terms in all three primary domains of GO, edges in *T* have the potential to link terms in different primary domains. The connections between terms in different domains has been investigated by others [39] and there are also documented “cross-domain” relationships in GO that are not subject to the DAG structure described above. In Supplemental Material, we explore these documented “cross-domain” relationships and show that they are enriched and have relatively higher edge-weights in the term-term network described by *T* (Figure A(a) in S1 Text).

### Identifying Communities of GO terms

We next sought to explore the community structure in annotation-driven term-term relationships, by identifying term communities, i.e. clusters of terms within which there are many or high-weight relationships in our projected network Eq (2), but between which there are only few or low-weight relationships. In order to quantify the strength of community structure we use a quantity known as modularity [27]. Modularity (*Q*) can be defined as:

$$\begin{array}{c} Q=\frac{1}{2m}\sum_{ij}[A_{ij}-(1+\frac{r}{\left\langle k\right\rangle })\frac{k_{i}k_{j}}{2m}]\delta \left(x_{i},x_{j}\right) \end{array}$$ (3)

where *δ* is the Kronecker delta function, *x*_*i*_ is the community of node *i*, *k*_*i*_ is the degree of node *i*, *A* is the adjacency matrix, a matrix with values representing the weight between nodes *i* and *j*, and *m* is the total weight of the edges in the network [40, 41]. Traditionally, in order to divide a network into communities, the resolution parameter, *r* in Eq (3), is set equal to zero and a heuristic is employed to identify a partition of the network that maximizes the modularity. Varying this value allows one to look for alternate divisions of a network into communities at different scales, or resolutions, with *r* > 0 uncovering sub-structures in the network [41].

We used a weighted version of the Fast Greedy Community Structure algorithm [28] to investigate the community structure of our term network, and found 51 communities at maximum modularity. We then implemented a modified version of the Fast Greedy that maximizes modularity for non-zero values of the resolution parameter in order to find many different viable partitions. We varied the resolution parameter several orders of magnitude, choosing values that resulted in communities whose sizes are roughly similar to those defined by the branches of the GO DAG (see Figure A(b) in S1 Text). This process identified 14013 different communities (see Table A in S1 Text). Like GO branches, which represent overlapping sets of functional categories rather than one discreet partition of terms, communities found at different resolutions are highly overlapping and represent functional structure at many different levels of specificity. We give our communities numeric identities that vary from TC:0000001 to TC:0014013 and will refer to them as such in the following analysis. A file including these communities and their term members can be found in the Supplemental Material (S1 Data).

## Results

### Term Communities and GO Branches Represent Distinct Collections of Biological Functions

**Fig 2:**
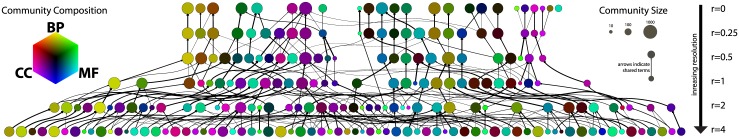
Visualization of Communities (Circles) of GO Terms Found at the Six Lowest Levels of Resolution (Rows), in Increasing Order (Top to Bottom). The width of the line connecting two communities is proportional to the percentage of terms in the child community that are also in the parent community. The size of communities is proportional to the log of the number of terms in the community. Color represents the normalized percentage of terms in the community which belong to the BP (yellow), MF (cyan) and CC (magenta) primary domains.

To better understand the relationships between the communities found at different resolutions, we visualized the term communities with ten or more members for the six lowest values of resolution used (Fig 2). In this visualization each community is represented by a single circle, whose radius scales as the log of the number of terms belonging to that community and whose color corresponds to the percentage of members from each primary domain that belong to that community. Between the communities found at adjacent resolutions, we draw a line from a community at a higher resolution to a community at a lower resolution if at least 10% of the members of the community from the higher resolution also belong to the community at the lower resolution. The thickness of the line is indicative of the overlap between the two communities. For more details on the creation of this figure see the Supplemental Material (S1 Text).

The structure of annotation-driven term relationships is distinct from the structure of those relationships as defined by GO branches. This is evidenced clearly by the fact that, although each GO branch can only belong to one primary ontology, and thus would be pure yellow, cyan or magenta in this type of visualization, communities, even smaller ones and those found at higher resolutions, generally contain members from multiple ontologies, resulting in a rainbow of colors. We also observe that communities at higher resolutions do not merely represent the “splitting apart” of communities at lower resolutions (represented by a child community only connecting to a single parent), but instead each resolution often brings about a new way of partitioning the network. An analogous visualization of GO branches reveals a similar complex partitioning, albeit segregated by primary domain (see Figure C in S1 Text).

**Fig 3:**
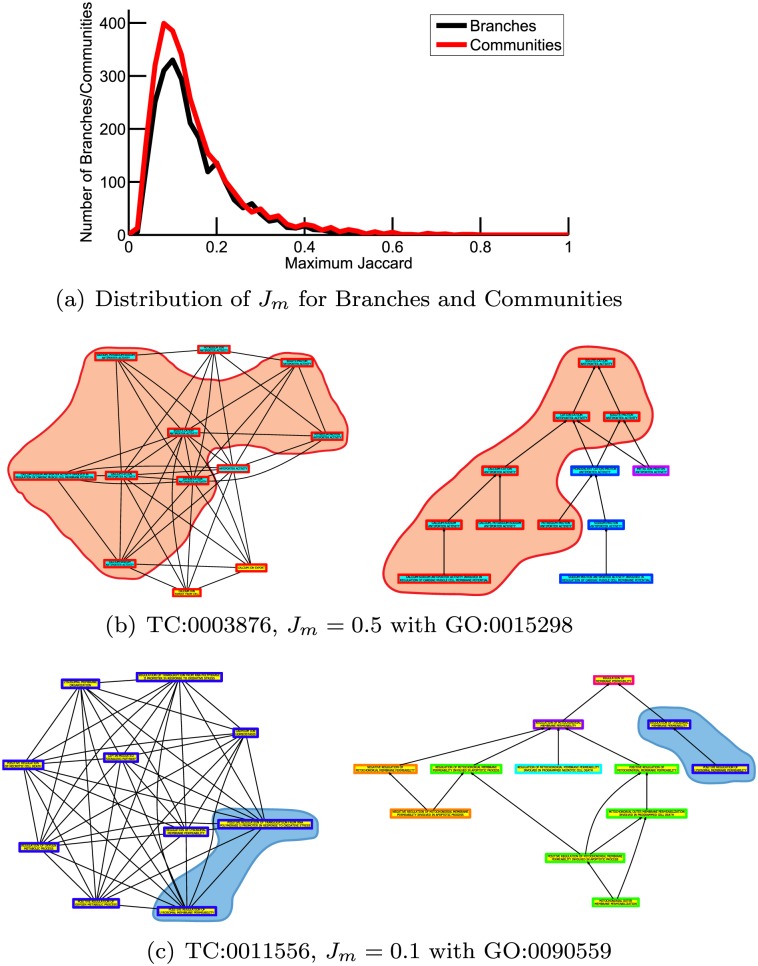
A Comparison of Branches in the GO DAG and Term Communities Found by Partitioning the Term Network. (a) Distribution of *J*_*m*_, the maximum similarity a community or branch with ten or more members has compared to all other branches or communities with ten or more members, respectively. Although a small number of communities and branches have similar memberships, most are highly dissimilar. (b)-(c) Two example comparisons between communities and branches: (b) TC:0003876 compared to GO:0015298, and (c) TC:0011556 compared to GO:0090559. In each panel on the left hand side a community and its inter-community connections in the annotation-driven term network is shown and on the right hand side the branch with which that community has the the highest Jaccard similarity is illustrated. In the right panel edges represent the ontological associations defined by the Gene Ontology term hierarchy. Each term member of the community or branch is colored both by its associated primary domain (inner color—BP:yellow, MF:cyan, CC:magenta) and its community membership (outer color), determined at the same resolution value as the illustrated community. Terms common between each community and branch pair are circled. To read term-labels, please zoom in.

Next we directly compared the membership of the term communities with that of branches in the GO DAG. In order to quantify the similarity between each community and branch, we calculated the Jaccard similarity, which takes the value *J*(*x*, *y*) = |*x*∩*y*|/|*x*∪*y*|. Then, for each community (*x*), we determined the corresponding branch (*y*) that has the highest overlap in membership by this measure: *J*_*m*_(*x*) = max{*J*(*x*, *y*):*y* ∈ *Y*}, and vice versa. Because the exact value of the Jaccard similarity is highly sensitive to incremental changes in set membership when comparing sets with only a few members, we limit all the following analysis to communities and branches that contain ten or more terms in order to focus on the most robust results. Fig 3(a) shows the distribution of *J*_*m*_ comparing these 2929 communities and 2439 branches. Although a handful of communities and branches are quite similar to each other, the majority of communities are dissimilar to the GO Branches and vice versa. We have repeated this analysis constructing the term network and corresponding partitions three more times, using annotations specific to each of the three primary domains, and observe similar results (see Figure B and Table B in S1 Text).

To better interpret these values, we selected several communities to inspect more closely. First we selected a community with a high *J*_*m*_ value to inspect (Fig 3(b)). TC:0003876 is most similar to GO:0015298 (“solute:cation antiporter activity”) with *J*_*m*_ = 0.5. Overall, we observe the terms found in the community but not the branch are consistent with known biology, indicating that these connections may lead to important insights into the relationships between these functions. For example, members of the community that are not in GO:0015298 include “antiporter activity” and “potassium ion antiporter activity”. It is also interesting that in addition to members from the MF domain, TC:0003876 also includes two members from the BP domain, “calcium ion export from cell” and “calcium ion export”. One of the primary mechanisms for calcium export from the cell is through an antiporter, or exchanger [42, 43].

Next we selected TC:0011556, which is most similar (*J*_*m*_ = 0.1) to GO:0090559 (Fig 3(c)). We note that the dissimilarity found between this community and branch cannot be attributed to community membership from multiple primary domains, as all of TC:0011556’s members belong to the “Biological Process” primary domain. Interestingly, the branch defined by GO:0090559 has members that belong to six different communities, demonstrating that not only are communities often distinct from branches, within the branches themselves the annotation-driven classification is often very distinct from the defined ontological relationships. The branch/community pair shown in Fig 3(c) is a representative example of the maximal shared information that is typically found between a community and branches, therefore we conclude that although there is occasional similarity between our found communities and GO branches, the communities are not simply a recapitulation of the DAG.

We remind the reader that because every parent term takes on the annotations of its children, in *T* a parent term is connected to all of its children, and vice versa. However, these relationships are differentially-weighted based on the specificity of shared annotation information (Eq (2)). Therefore, what this analysis is telling us is that the specificity of shared annotations is often not the highest between a parent and a child term, but between two terms that reside in different branches of the GO.

### Biological Information in Term Communities

One advantage of the hierarchical organization of GO is that the collection of terms that make up a GO branch can be easily summarized by considering only the parent node of that branch. At this point we have identified strongly connected groups of terms that are organized differently from the GO DAG, but we lack a way to probe the biological information captured in these communities. We know that on a mathematical level they represent sets of biological functions that are generally performed by the same collection of genes. However identifying and understanding the biological meaning behind these communities is vital if they are to have wide-range applications similar to the GO branches. As a step toward interpreting the contents of our term communities, we visualize the names of member terms in the form of word clouds.

To create a word cloud, we first make a list of all the member terms in the community, recording the primary domain of each. For each different word that appears in the list, we color it according to the percentage of its occurrences that come from terms in the different domains. For example, a coloring of yellow indicates that, within the specified community of terms, the word appeared only in term names from the domain “Biological Process.” Similarly, cyan indicates words derived solely from “Molecular Function” terms, and magenta denotes words derived solely from “Cellular Process” terms. In this scheme, words are colored black if they have an equal (normalized) percentage of occurrences from all three primary domains. We also count the number of times a word appears across all member terms in a community and compare that to the word’s frequency across all terms. We then set the size of the word proportional to its statistical enrichment in the community, calculated using the hypergeometric probability. Thus the size of a word does not simply reflect its number of occurrences in the list of terms that make up a community. Rather, it reflects the statistical enrichment of its frequency in the term community compared to its frequency across all terms. Additional details about the construction of word clouds can be found in the Supplemental Material (S1 Text).

**Fig 4:**
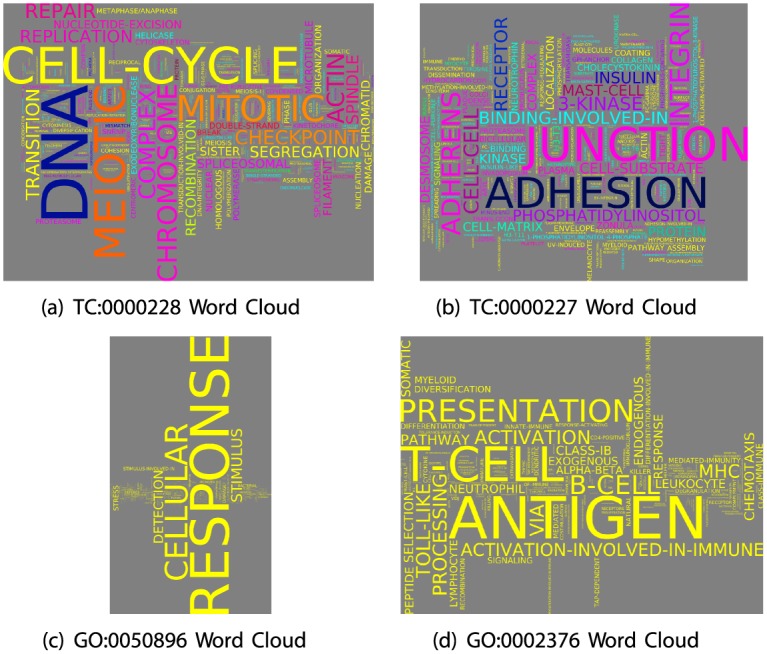
Biological Information in Term Communities. (a-d) Term Communities (TC:0000228, TC:0000227) and branches (GO:0050896, GO:0002376) summarized as word clouds. In each case the color of a word represents how often the term description containing that word belongs to each of the primary domains (BP:yellow, MF:cyan, CC:magenta, also see Fig 2 for mixed-domain coloration) and size represents that word’s statistical enrichment in that community/branch.

Following the word cloud construction technique described above and further detailed in Supplemental Material, we illustrate the biological content of two communities in Fig 4(a) and 4(b). These word clouds display the richness of the biological information contained in their corresponding term communities. For example, although Community TC:0000228 (Fig 4(a)) contains 945 members harking from all three primary domains, the word cloud presentation easily summarizes this information. We observe that this community includes biological concepts related to the cell-cycle and DNA repair, such as “mitotic”, “meiotic”, “checkpoint”, “repair”, “nucleotide-excision”, “recombination”, “replication” and more. Interestingly, the individual words are often contained in terms associated with multiple domains, resulting in a complex coloration. Our second example, TC:0000227 contains words such as “integrin”, “insulin”, “adherens”, “adhesion” and “junction” (Fig 4(b)). Neither community is very similar to any particular branch in GO, although they represent similar biological information. TC:0000228 is most similar (*J*_*m*_ = 0.12) to GO:0022402 or “cell cycle process”, and TC:0000227 has the highest similarity (*J*_*m*_ = 0.046) with GO:0016773, or “phosphotransferase activity, alcohol group as acceptor”.

We point out that one can also represent the biological information contained in branches in the form of word clouds, although, because the members of each branch can only belong to one of the three primary domains, all the words in the cloud will be the same color. Word Clouds for two branches are illustrated for comparison in Fig 4(c) and 4(d). The first, GO:0050896, or “response to stimulus” contains 905 member terms, but the corresponding word-cloud is dominated by a handful of words, including “cellular”, “stimulus”, “response” and “detection”. However, several of the smaller words, such as “stress”, “defense”, “damage” and “bacterial”, are also indicative of the types of functions encapsulated in this branch. Similarly, the cloud for GO:0002376, whose parent term name is “immune system process” contains words pertaining to the immune system. In contrast to GO:0050896, the richness of word-information in this cloud is more similar to that represented in the term community clouds.

### Term Communities Can Be Used to Evaluate and Predict Genetic Function

Finally, we wanted to test how our communities might be used in one common application of the Gene Ontology: functional enrichment analysis. The goal of functional enrichment analysis is to determine the biological functions associated with experimentally determined gene sets. Traditional methods for using the GO database to determine the functional enrichment of gene sets are designed to estimate the statistical significance of the overlap between two groups of genes: (a) gene set of interest and (b) the set of genes annotated to a particular GO term [44]. Because all genes annotated to the progeny of a given term are also annotated to that term itself, calculating the enrichment of a gene set for a specific functional term can be thought of as determining the functional enrichment of the set with respect to the *group* of terms represented by the term’s GO branch (i.e., the term itself and all of it’s descendants). In a similar way, we seek to determine the functional enrichment of experimentally derived gene sets with respect to the groups of terms defined by our term communities.

We note that the aforementioned gene-set overlap statistics for determining functional enrichment do not account for the high level of heterogeneity in the number of functions associated with individual genes or the number of genes annotated to individual functions. Because of these limitations, we instead use Annotation Enrichment Analysis, which has been shown to address these biases by properly accounting for the heterogeneities in the null model used to determine statistical significance [38]. In practice, the appropriate treatment of these heterogeneities is particularly important when evaluating the connection between an experimentally derived gene set and a group of terms that has many associated genes. In the Supplemental Material, we provide some comparisons between AEA and a traditional method using Fisher’s Exact Test to determine enrichment, illustrating that the traditional approach erroneously identifies gene signatures as being statistically enriched with randomly constructed groups of functional terms (Figure E in S1 Text). Despite the advantage of AEA in this context, we acknowledge that there are likely other biases in annotation data that it does not properly account for, and which may affect our functional enrichment results.

For our analysis, we downloaded a collection of experimentally derived genes sets from the Gene Signatures Database (GeneSigDB) [45]. This database is a manual curation of previously published gene expression signatures, focusing primarily on cancer and stem cell signatures [46], and includes 497 human signatures that contain 100–1000 genes annotated in the Gene Ontology. We then used Annotation Enrichment Analysis (AEA; [38]) with 10,000 randomizations to determine functional enrichment in both term communities and GO branches. For simplicity we focus on term communities and branches that have ten or more members, and exclude those with more than one thousand members.

**Fig 5:**
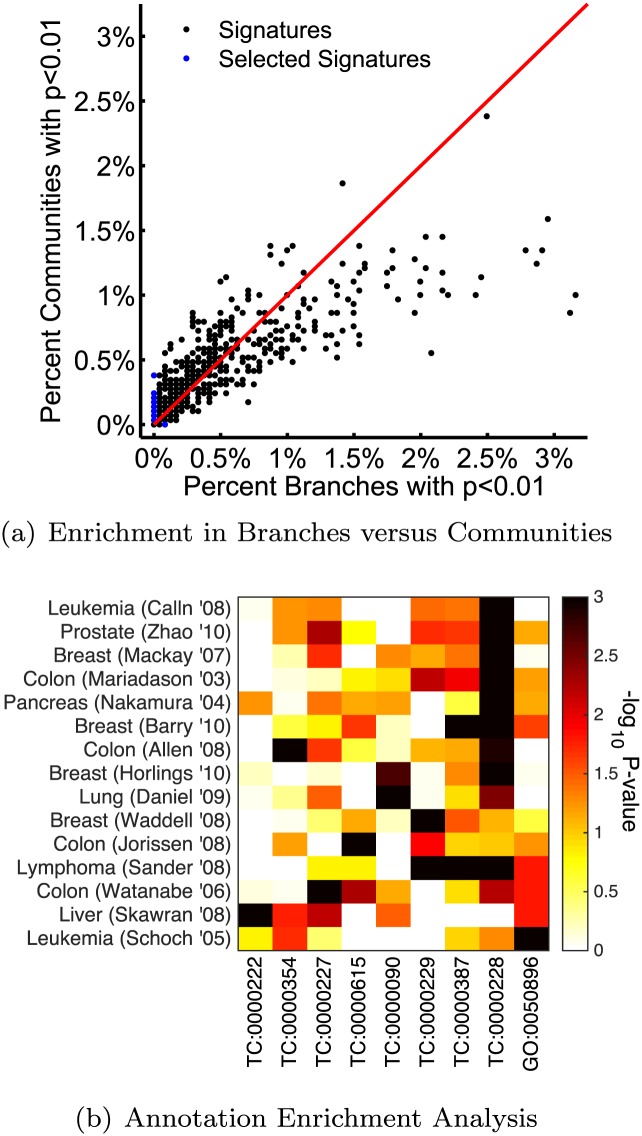
Functional Enrichment Analysis in Branches and Communities. (A) A plot of the percentage of branches and percent of communities found to be enriched at *p* < 0.01 in each gene signature. Although both communities and GO-branches have enrichment in these signatures, many signatures are only enriched in communities and not branches at the *p* < 0.01 significance. We chose a subset of signatures to investigate further, and note those with a blue dot. (b) A heat map showing the statistical enrichment of selected cancer signatures (noted in (a) with a blue dot), as measured by AEA, in both GO branches and term communities.

To evaluate whether term communities reflect important biological information, we determined, for each gene signature, the percentage of term communities that signature was enriched in at the *p* < 0.01 significance according to AEA. Similarly, we determined percentage of GO branches each signature was enriched in at the *p* < 0.01 significance. We then compared these values (Fig 5A). We observe that not only are cancer signatures enriched in GO branches (as might be expected), there is also a large level of enrichment in term communities. More interestingly, we observe a number of gene signatures that are enriched in at least one community at the *p* < 0.01 cutoff, but in no branches at this same cutoff. More specifically, there are 34 cancer signatures which are only enriched in communities at the *p* < 0.01 significance level, while only 2 signatures are only enriched in branches at the *p* < 0.01 significance level. Although the GO branches contain important biological information, this analysis demonstrates that the term communities can capture this information and additional, potentially important, functional associations.

Knowing that our communities are statistically associated with experimental gene signatures, we next sought to determine in what context our term communities captured biological information from these signatures that was missed by the branches, or vice versa. Along these lines, we selected signatures that are enriched in at least one community at *p* < 0.001 but no branches at *p* < 0.01. Fourteen signatures met this criteria. We also identified signatures that are enriched in at least one GO branch at *p* < 0.001 but no term communities at *p* < 0.01. Only one signature met this criteria. Fig 5(b) shows a heat map of the significance values representing the association of these fifteen signatures across any community or branch statistically enriched in at least one of those signatures. Additional information about these signatures can be found in the Supplemental Material (S2 Data).

The only signature enriched in at least one GO branch but no communities is a Leukemia signature (bottom signature in Fig 5(b)), which is significantly associated with GO:0050896, or “response to stimulus”. The word cloud for the branch defined by this term and all its progeny is shown in Fig 4(c). Curiously, this signature includes all genes localized on chromosome 8 in a copy-number variation experiment exploring trisomy 8 in Acute Myeloid Leukemia (AML). As noted in the original publication, the median expression of these genes was 1.27-fold higher in trisomy-8 cases compared to AML patients with a normal karyotype. Based on this, one could hypothesize that one effect of trisomy 8 in AML is a differential response to stimuli, something that has been observed for AML in other contexts [47].

Among the fourteen signatures that are enriched in at least one community, but no GO branches, we find that TC:0000228, illustrated in Fig 4(a), plays an important role. This community contains terms that are related to both cell proliferation (with words such as “cell-cycle” and “mitotic”) and DNA repair (with words such as “break”, “damage”, “mismatch”, and “DNA-integrity”). It makes sense that the cellular activities described in this community would be important across a range of cancer signatures, especially given the high rate of cell proliferation [48] and the importance of mutations in many cancers [49, 50]. We hypothesize that one reason that this community is highlighted in our functional enrichment results may be due to the fact that genes often perform multiple functions; for example they could be simultaneously involved in both cell-cycle processes and DNA-repair. However, if the number of overall cell-cycle genes or DNA-repair genes is relatively low in a given signature, the signature will not be enriched in the corresponding GO branches. By combining these concepts rather than evaluating them separately, we believe we are highlighting important information about the biological processes important for these genes.

In addition to TC:0000228, there are also several term communities in Fig 5(b) that are only enriched in a small number of our selected signatures. One example, TC:0000227, illustrated in Fig 4(b), is most enriched in a colon cancer signature. This signature includes genes that are differentially-expressed between responders and non-responders to preoperative radiotherapy. TC:0000227 included words such as “insulin”, which is known to be associated with colon cancer risk [51] and important for mediating tumor growth [52]. TC:0000227 also includes “adherens”, “integrin” and “adhesion”. In colon cancer cells, cadherin-17 has been found to interact with *α*2*β*1-integrin to regulate cell proliferation [53]. Overall, we find interesting functional information in this term community that is highly relevant to the biology of the associated gene signature.

In this section we have only discussed a subset of the term communities shown in Fig 5(b), which are themselves a small portion of all the term communities that are enriched in these cancer signatures. We note that in investigating these enrichment results, we found many other interesting biological features, which are too numerous to explore in sufficient detail here. However, our desire is that these term communities will be a resource that will help provide many future biological insights.

## Discussion

The network structure of gene annotations made to GO terms has not previously been exploited in a manner that reveals an organization of biological function unique from the published hierarchical classification of the Gene Ontology DAG. By analyzing the relationships between genes and functional terms reported in the GO database, we were able to construct an alternate, annotation-driven, and biologically-relevant way in which to categorize cellular functions. This categorization is structurally and conceptually distinct from the GO DAG and allows us to uncover multiple, strong connections between terms that do not share a parent/child relationship. It takes advantage of a large amount of data from a variety of sources and creates a classification scheme that is driven primarily by the data reported. Our aim is for this new organization of biological functions to be used alongside the one captured by the Gene Ontology to evaluate the functional properties of experimentally derived gene sets.

The term communities defined in this work represent an integration of information across all three primary domains in GO that, to the authors’ knowledge, has not previously been investigated in this manner. However, we do not suggest that the communities we define here are the only endogenous way to group functional terms outside of the ontology structure. A different construction of *T* or the application of other community structure methods, such as those published in [54–57], would likely lead different sets of functional communities. Such other alternate classifications represent a generalization of our approach and we hope to see such explorations in the future.

Because annotations are continually being improved and added to the GO database (reflecting the substantial efforts of many curators [58]), the organization of functional terms uncovered by our approach can change over time. This is both a drawback and a benefit of our method. It’s a drawback because researchers might reasonably desire that the results of functional enrichment calculations be independent of the state of the database at the time of the calculation. It’s a benefit because newly discovered connections between genes and functions can reveal previously missed relationships between functional terms. Here we have reported our results and analysis for a version of the GO database downloaded on May 28, 2015. We note that we obtained very similar results with older versions of the database. Consequently, we expect the results of our approach to be relatively stable over time, with a few exceptions that may reflect newly discovered biological phenomena.

In this study we investigated if an alternative classification of GO terms exists and whether this different organization of biological functions could be used to help interpret experimental data. We believe that our functional enrichment analysis demonstrates that the term communities we define are more than a mathematical artifact and have a high potential to be applied to better interpret biological data.

## Supporting Information

S1 Data CommunityMembers.txt. A text file that lists information about the term communities, including the GO terms belonging to each.
(TXT)

S2 Data SignatureInfo.txt. A text file containing information about the fifteen gene signatures highlighted in Fig 5. These signatures were derived from the collection in the Gene Signature DataBase [45].
(TXT)

S1 Text SupplementalMaterial.pdf. This file contains additional analyses and information that complement what is presented in the main text.
(PDF)

S1 Code TermCommunities.tgz. This file contains the input human annotation files and all the code needed to reproduce the analyses and figures presented in this manuscript. The complete collection of intermediate files (such as the predicted term-term networks, word clouds for all communities, etc), can be obtained from [34].
(TGZ)
