# Supplementary material for: Finding New Order in Biological Functions from the Network Structure of Gene Annotations
Source: PLoS Comput Biol. 2015 Nov 20;11(11):e1004565. doi: 10.1371/journal.pcbi.1004565 (PMC4654495; doi:10.1371/journal.pcbi.1004565)
Supplement: S1 Text — This file contains additional analyses and information that complement what is presented in the main text. (PDF) [file pcbi.1004565.s003.pdf]

Supplemental Material and Methods For: Finding New Order in Biological Functions from the Network Structure of Gene Annotations

1. SUPPLEMENTAL MATERIAL

In this section we provide additional materials meant to complement and expand upon the analysis in "Finding New Order in Biological Functions from the Network Structure of Gene Annotations." In the first section, "Enrichment of Term-Network Edges in Known Cross-Domain Relationships", we compare edges predicted in our network model to documented relationships in the Gene Ontology that extend between terms in different primary domains. In the second section, "Communities Found Across Multiple Resolutions", we provide more detail about how we used the resolution parameter to generate multiple, overlapping, yet unique communities of terms. In the third section, "Domain-specific Analysis" we provide analysis of communities of terms compared to branches within each primary domain of GO. Next, in "Visualizing GO Branches" we illustrate relationships between GO branches at different "levels" of the GO DAG. In the section entitled "Functional Enrichment Analysis", we briefly explain the methodology used to evaluate the statistical enrichment of gene sets in the term communities and GO branches, and provide analysis showing that, as desired, this enrichment is absent for randomly generated term communities or randomly generated sets of genes. Finally, in "Comparative Species Analysis" we construct and analyze annotation-driven term networks using gene annotations for seventeen additional organisms and compare those networks with each other as well as the human results presented in the main text.

For information regarding the methodology used to illustrate the term communities or generate the word clouds in the main text, see Section 2, "Supplemental Methods'.'

1.1. Enrichment of Term-Network Edges for Known Cross-Domain Relationships

Relationships between terms in the Gene Ontology DAG structure are primarily between terms that belong to the same primary domain. More specifically, "is a" relationships can exist only between two terms that belong to the same primary domain. On the other hand, although most term-term relationships in GO are between pairs of terms in the same primary domain, other relationships, such as "has part", and "regulates" can extend between two terms in different primary domains and are not subject to the DAG structure. We sought to evaluate if our term-term network was recovering these known "cross-domain" relationships.

We started with 2104 term-term relationships in GO that join two terms that are not in the same primary domain, including 1248 unique relationships that extend between terms with human gene annotations. We find that 970 (78%) of these "cross-domain" relationships in the latter set are also predicted edges in our term-term network (T). Since only 6.5% of all possible term-term relationships are included in T, this is a 12 fold-enrichment as compared to chance.

Next, we plotted the distribution of the weights for all edges in T and then separately evaluated the weight distribution for the 970 edges in T that have a documented cross-domain relationship (Figure S1(a)). We see a pro-

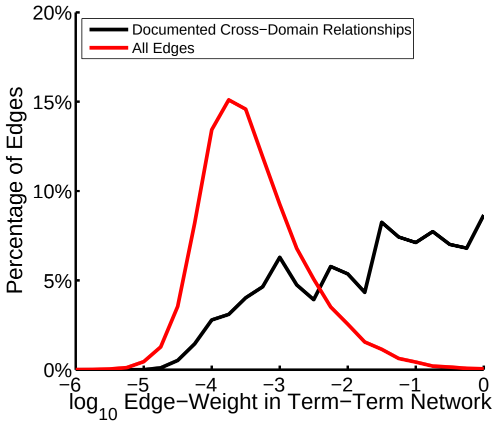

(a)Cross-Domain Enrichment

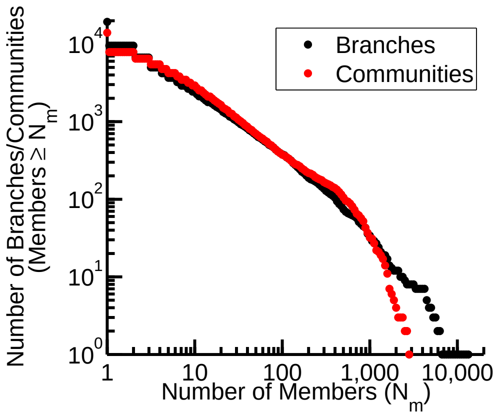

(b)Distribution of Branch and Community Sizes

FIG. A: (A) Distribution of all term-term network edge weights, and the distribution of the subset of those edges that also have a documented relationship that extends between the primary domains. (B) The cumulative distribution for the sizes of all branches in the Gene Ontology and all unique term communities found at the various resolutions. We observe that the number of terms in branches and term-communities is very similar except for less than a hundred branches that contain a very large number of terms.

Value of r (Resolution)	Number of Communities	Number of New Communities
0	51	51
0.25	59	39
0.5	73	44
1	116	65
2	129	83
4	179	163
8	357	309
16	643	499
32	1072	755
64	1738	1137
128	2716	1694
256	4138	2394
512	5921	3050
1024	8148	3730

TABLE A: The number of communities found at each value of resolution used. As the resolution is increased, the number of communities found at that resolution increases as well.

found enrichment for cross-domain relationships to have a higher predicted edge-weight in T. Cross-domain relationships have a median edge-weight of 2.86×10^{-2}, compared to an overall median edge-weight of 3.2×10^{-4}.

1.2. Communities Found Across Multiple Resolutions

In order to find additional viable partitions of the annotation-driven term network, representing different resolutions, we varied the weighting parameter (see r in Equation 3, and "Methods" in the main text). In addition to the fundamental partition ($r = 0$) we chose values of r in geometrically increasing steps ranging from $r = 2^{-2} = 0.25$ to $r = 2^{10} = 1024$ such that the final distribution of community sizes would resemble that of the branches. This procedure resulted in fourteen different partitions of the terms in the annotation-driven network such that, initially, each term can be assigned to exactly fourteen communities, one at each resolution. We emphasize that while communities at any given resolution do not overlap, communities at different resolutions can be highly overlapping.

We point out that it is possible for the membership of a community found at one resolution to be identical to the membership of a community found at another resolution. In order to eliminate completely redundant community information, at each resolution, we determined if any of the communities found at that resolution were identical in membership as a community found at a lower resolution. If so, we "collapsed" the two community assignments into that of the community from the lower resolution. For example, of the 59 communities found at a resolution value of $r = 0.25$, there were 20 which each had an identical membership to one of the 51 communities found at $r = 0$. We removed those 20 "redundant" communities from the $r = 0.25$ partitioning, retaining only their $r = 0$ assignment, and record that at $r = 0.25$ only 39 additional communities are found. Similarly, of the 73 communities found at a resolution of $r = 0.5$, there were 29 which each had an identical in membership to one of the 90 unique communities found at $r = 0$ or $r = 0.25$. These "redundant" communities were removed and we record only 44 additional communities found at $r = 0.5$. This procedure was repeated for the remaining resolutions, resulting in 14013 "unique" communities (see Table A). The cumulative distribution of the number of members in these communities is shown in Figure S1(b). For comparison, on the same graph we also plot the cumulative distribution of the number of terms in each of the GO branches. These distributions are heavy-tailed and demonstrate that the number and sizes of branches and communities are highly similar. The heavy-tailed distribution for branches is a result of the hierarchical DAG structure, as the members of each branch are also members of their parent branch(es) (see [1, 2]). Note than in contrast to the term communities, there are a handful of GO branches that are very large, with thousands of term members; these represent the very "top" terms in the GO DAG (for example "Biological Process").

1.3. Domain-specific Analysis

The Gene Ontology is broken into three, fully independent, primary domains, each of which takes the form of a directed acyclic graph [3]. In the main text we combined information from all three primary domains to construct the annotation-driven term network. Because of this, the dissimilarity between the GO branches and the term communities we find by partitioning this network may be at least partially attributable to the fact that members of a particular community can belong to any of the primary domains whereas members of a particular GO branch must all belong to the same primary domain based on the construction of the hierarchy. To address the extent of this issue, we constructed three "domain-specific" term networks by using only terms specific to a particular primary domain and the gene annotations made to those specific terms. We partitioned each of these networks using the same resolution values as were used previously and, as with the partitions using all annotations, we retained only the "unique" set of communities for the following analysis (see Section 1.2). Table B shows the number of communities found and the number of branches defined in GO for each of the primary domains.

Next we compared the membership of these communities and branches using the Jaccard similarity (see "Term Communities and GO Branches Represent Distinct Collections of Biological Functions" in the main text). We did the comparison three times, each time confining the

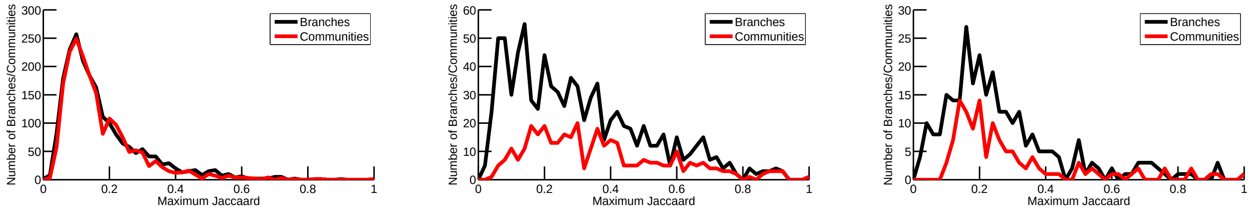

(a)Biological Process Terms (b)Molecular Function Terms (c)Cellular Component Terms

FIG. B: Distribution of J_m, the maximum similarity a domain-specific community or branch with ten or more members has compared to all other domain-specific branches or communities with ten or more members, respectively. Although a small number of communities and branches have similar memberships, most are very dissimilar.

comparison to those branches defined by a particular primary domain and the term communities derived from the network constructed using those terms and their corresponding gene annotations. The distribution of J_m (Figure SB), the maximum similarity a community has to any branch, or vice versa, for each of the domains, is not strikingly different from that presented when using information collected from all three domains (see Figure 3(a) in the main text). There might be slightly more similarity when directly comparing communities and branches derived from either the "Molecular Function", or "Cellular Component" primary domains, but we remind the reader that the majority (just over two-thirds) of the terms in GO belong to the "Biological Process" primary domain, and this distribution (Figure S2(a)) is practically indistinguishable from the one presented in Figure 3(a) of the main text.

1.4. Visualizing GO Branches

To better understand the relationships between the branches in the Gene Ontology, we visualized branches in a manner similar to the way we visualized the relationships between term communities found at different resolutions (see Figure 2 in the main text). First, we determined a "level" for each GO branch in order to segregate the branches in a manner similar to the resolution parameter. To begin, the head nodes of the three primary domains ("Biological Process", "Molecular Function", and "Cellular Component") were assigned to level one. Next, we determined branches for which the head-node is a term that has a parent-child relationship with one of these three level-one terms (but no other terms), and assigned those terms a level of two. Continuing, head-nodes that have parent-child relationships with only level-one or level-two terms were given a level assignment of three, and so on. This assignment was repeated until all head-nodes were assigned a "level". Branch levels were then defined based on the level of its head-node.

	Number of Branches	Number of Communities
All Terms	19329	14013
BP Terms	13508	11718
MF Terms	4229	5899
CC Terms	1592	2860

TABLE B: The number of branches defined within each primary domain as well as the number of communities found by varying the resolution parameter and partitioning a term-network derived by gene annotations made to terms in this primary domain. The large size of the "Biological Process" primary domain compared to the others is evident.

We visualized branches with one-hundred or more term-members at the six lowest levels (Figure C), lining up branches with the same level assignment horizontally. Each branch is represented by a single circle, whose radius scales as the log of the number of terms belonging to that branch and whose color represents whether the terms in the branch belong to the BP (yellow), MF (cyan) or CC (magenta) primary domain. Between the branches found at adjacent levels, we draw a line from a branch at a higher level to a branch at a lower level if at least 10% of the members of the branch from the higher level also belong to the branch at the lower level. The thickness of the line is indicative of the overlap in membership between the two branches.

Unsurprisingly, we observe three distinct sets of interconnected branches corresponding to branches belonging to each of the three primary domains. Like in the visual representation of the term communities across different resolutions (see Figure 2 in the main text), we observe a lot of "cross-talk" between branches at adjacent levels, whereby a branch at a given level is very likely to contain members from multiple branches at a lower level. We note that in this representation, an individual term can be a member of multiple branches at the same "level". This is in contrast to segregating communities by resolution, in which case each term only appears once on a given resolution-row. As a consequence, the inter-level connections between branches are somewhat structurally different from connections between communities found at adjacent resolutions. Namely, branches that share a term will necessarily also share a set of parent branches. Redundancy of the same term member(s) across multiple branches at a given level is visually evident among "Bi-

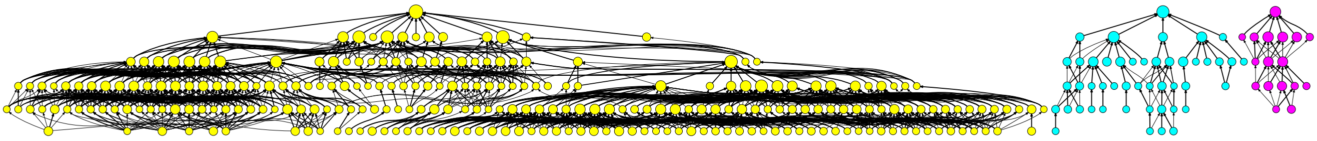

FIG. C: Visualization of branches of GO terms. Each branch is represented as a circle whose radius scales as the log of the number of terms in the branch. The width of the line connecting two branches is proportional to the percentage of terms in the child branch that are also in the parent branch. Color represents whether the terms in the branch belong to the BP (yellow), MF (cyan) or CC (magenta) primary domain.

ological Process" (yellow) branches, where there exists groups of branches at each level that connect primarily to the same set of branches at a lower level.

1.5. Functional Enrichment Analysis

We also wanted to test how our communities might be used in functional enrichment analysis, a very common application of the Gene Ontology. Traditionally, each branch of GO is collapsed to its head node and all the genes annotated to that head node are grouped into one "set." (Note that this set is the same as the genes annotated to the entire branch because of the propagation of gene annotation assignment, see the "Introduction" in the main text). Recently there has been evidence that this approach over-simplifies the complex structure of the Gene Ontology and has the potential to mis-represent the enrichment of gene sets in branches [2]. Therefore, we choose to use Annotation Enrichment Analysis (AEA) to evaluate the functional enrichment of experimentally-derived gene sets in both the GO branches and our term communities. AEA allows the user to specify a collection of terms and a collection of genes and uses a randomization protocol to evaluate the probability that these two sets are more connected than by chance.

We supplied AEA with the same gene-term bipartite network (B, see Equation 1 in the main text) as we used to construct our term-term network (T, see Equation 2 in the main text). We ran AEA using ten thousand randomizations defining collections of genes using a public database of Cancer Signatures [4] and using collections of terms defined by (1) membership in the term communities; or (2) membership in GO branches. For simplicity we focus on term communities and branches that have ten or more members, and exclude those with more than one thousand members.

To evaluate how well term communities reflect important biological information, we determined the number of gene signatures that were enriched in at least one community at a given p-value cutoff, but in no branches at this same cutoff. As a comparison we also determined the number of signatures that were enriched in at least one branch at a give p-value cutoff, but not enriched in any term communities at this same cutoff. For example, we find that 34 signatures are enriched in at least one community at $p < 0.01$ but no branches; at this same cutoff 2 gene signatures are enriched in at least one GO branch at $p < 0.01$ but no term communities. To make sure this result is not sensitive to the cutoff used, we varied our threshold value from 0.05 to 0.001 and plot the results in Figure SD. At the least significant cutoff ($p = 0.05$) almost all signatures that are enriched in communities are also enriched in branches, and vice versa. However, as we decrease the p-value cutoff, we observe a large number of signatures that are enriched only in term communities, and not in GO branches. This is evidence that our term communities are capturing real, biologically informative information that is absent in the GO branches.

Next, we plotted the total number of term and gene signature pairs that are enriched beneath various significance thresholds (Figure S5(a)). We observe clear statistical enrichment of experimentally-derived gene signatures in both term communities and GO branches. We wished to verify that this enrichment was not an artifact of the community construction – namely, we wanted to verify that experimental gene signatures are not enriched in random collections of terms and that term communities are not enriched in random collections of genes. Therefore we constructed "random" term communities by taking the community assignments of terms and swap-

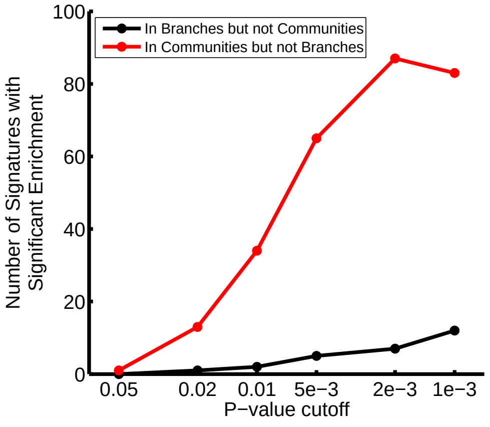

FIG. D: The number of cancer signatures that are statistically associated with only term communities and not branches, or only branches and not term-communities, across different p-value thresholds.

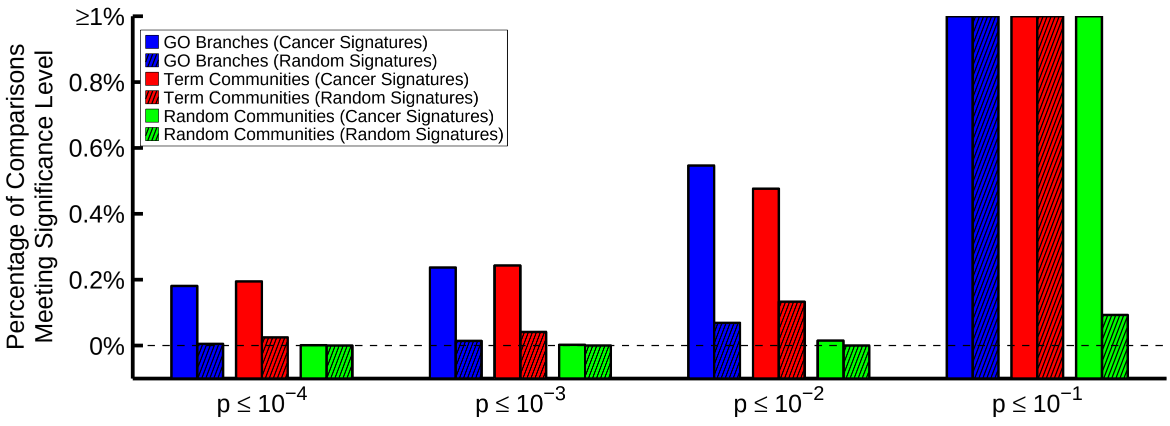

(a)Annotation Enrichment Analysis

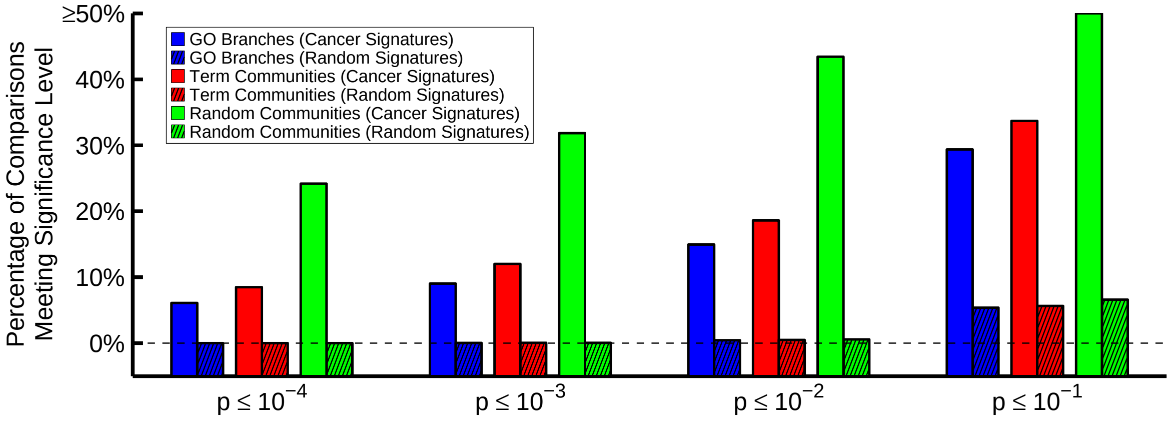

(b)Fisher's Exact Test

FIG. E: Level of statistical enrichment found when comparing branches, communities, and randomly generated communities with either cancer signatures or randomly generated gene sets. Only branches and our identified term communities show statistical enrichment in experimentally-derived gene signatures, with slightly more enrichment for the communities compared to the branches.

ping term labels. Similarly, we constructed "random" gene sets by randomly swapping gene labels. This gives us random term communities and random gene sets with both the same size and relative overlap as the real term communities and the experimentally-defined signatures. We then ran AEA an additional four times, using: (1) random communities and the experimentally-defined signatures; (2) term communities and random gene sets; (3) branches and random gene sets; and (4) random communities and random gene sets. The result of AEA indicated no enrichment using either random term communities or random sets of genes (Figure S5(a)), showing that the term communities generated by partitioning the annotation-driven term network contain distinct and useful biological information.

AEA evaluates the overlap in annotations made by a set of genes to a set of terms. Other, traditional functional enrichment analysis procedures, however, often use statistics such as Fisher's Exact Test (FET) to evaluate the overlap in two sets of genes – one defined by a gene set or signature of interest, and the other defined by taking the set of genes annotated to all the terms in a GO branch (same as the genes annotated to the parent node). Although there is evidence that such analysis is highly sensitive to the annotation degree of genes and terms [2], we wanted to see if our communities were enriched in cancer signatures using this more traditional approach. Therefore, for each GO branch, term community and random community, we took the collection of genes annotated to all terms in that branch/community/random community, and assigned this set of genes to represent that branch/community/random-community. We then evaluated the significance of overlap between theses sets of genes and sets of genes as defined by cancer signatures, or random sets of genes.

As with AEA, both term communities and GO branches show enrichment in experimentally-defined gene signatures using FET, with term communities perhaps having a slightly greater level of enrichment (Figure S5(b)). At first it is surprising to observe that *random*

communities also show a large amount of enrichment in the experimental gene signatures – much more than either the branches or real communities!! In retrospect, however, this serves to highlight a known weakness of FET to incorrectly over-estimate the significance of overlap when genes in a set contain a higher than expected number of annotations. Whereas our random gene sets represent a random sampling from all genes annotated to GO, the genes collected in the signatures published by GeneSigDB are biased in that they are generally annotated much more frequently to GO than one would expect by chance (see [2]). Furthermore, by taking all genes annotated to a collection of terms, highly annotated genes are also more likely to be represented in the gene sets representing the branches, term communities, and the random communities. The enrichment of the experimental gene signatures in the random communities, therefore, is attributable to the fact that FET finds significant overlap between sets containing an abundance of highly annotated genes, independent of the biological content of those sets. For more discussion, see [2].

1.6. Comparative Species Analysis

The Gene Ontology hierarchy establishes a species-independent terminology. However, by using species-specific gene annotation information, we can calculate species-specific term networks and use those to construct species-specific term communities. These communities will reflect the biological terms that are performed by the same sets of genes in a particular species and won't necessarily be the same across different species. Along these lines, in this section we evaluate the similarity between partitions of GO terms derived from the annotations of seventeen model organisms, including rice (*Oryza sativa*), thale cress (*Arabidopsis thaliana*), *Escherichia coli*, slime mold (*Dictyostelium discoideum*), *Aspergillus nidulans*, three types of yeast including *Candida albicans*, budding yeast (*Saccharomyces cerevisiae*), and fission yeast (*Schizosaccharomyces pombe*), worm (*Caenorhabditis elegans*), fruit fly (*Drosophila melanogaster*), zebrafish (*Danio rerio*), chicken (*Gallus gallus*), pig (*Sus scrofa*), cow (*Bos taurus*), dog (*Canine lupus familiaris*), mouse (*Mus muculus*), rat (*Rattus norvagicus*) and human (*Homo sapiens*). We downloaded gene annotation files for each of these species and projected term-term networks (see Section "Constructing a Term Network from Gene Ontology Annotations" in the main text). The number of genes, terms and annotations included in these networks is shown in Table C.

Next we partitioned each of these networks into communities (see Section "Identifying Communities of GO terms" in the main text). For simplicity we choose to focus only on the fundamental partition (resolution parameter $r = 0$, see Equation 3 in the main text). This results in exactly one discreet partitioning of GO terms associated with each species.

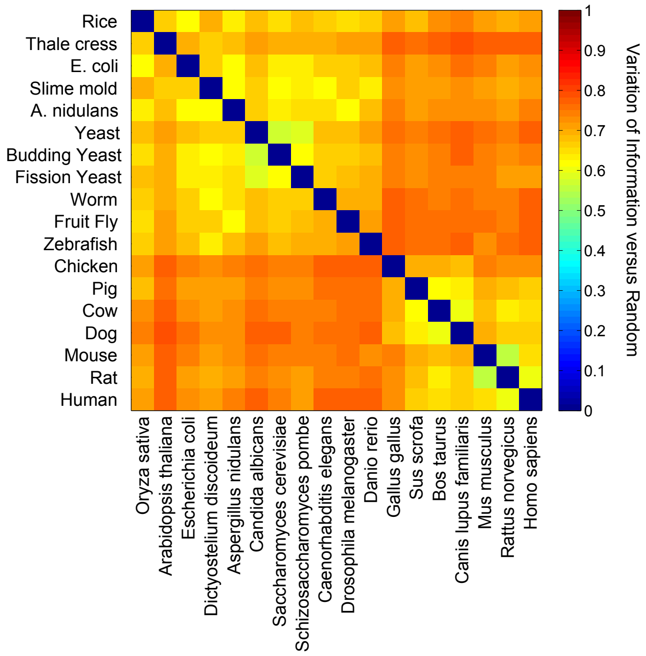

(a)Community Comparison between Species Networks Built Using all Annotations

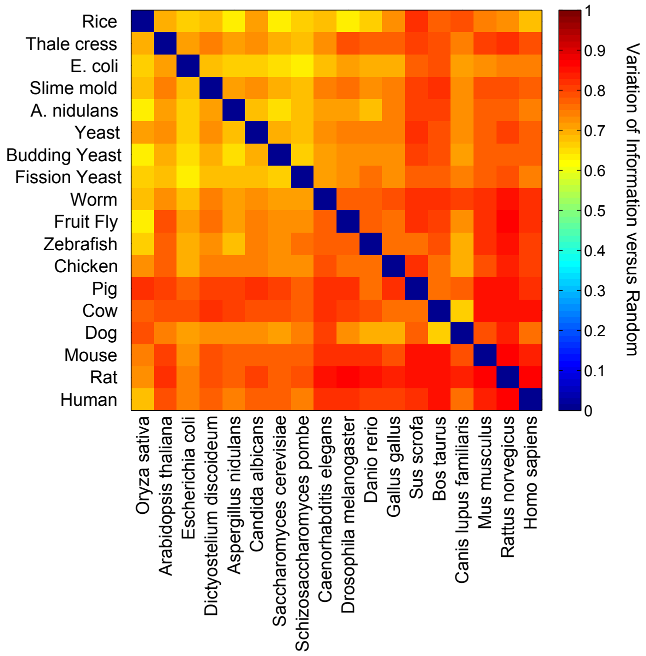

(b)Community Comparison between Species Networks Built Using only Experimental Annotations

FIG. F: The variation of information between term partitions generated from species-specific projected term networks. Terms networks were either constructed using (A) all annotations, or (B) only experimental annotations. Labels along the x-axis are the scientific name for the species and labels along the y-axis are the common name for the species. In both figures we see that many of the partitions are slightly more similar than random partitions, but are also not identical, indicating that these species-specific communities of functional terms carry distinct information.

Species	#Terms	#Genes	#Annotations
Rice	1568 (678)	40128 (106)	404726 (2726)
Thale cress	8489 (6946)	30214 (12238)	1199010 (322073)
E. coli	4575 (4490)	2844 (2755)	87624 (82334)
Slime mold	5847 (2496)	8180 (1289)	276516 (45010)
A. nidulans	5075 (2963)	138865 (1850)	3271510 (43363)
Yeast	9074 (2964)	46166 (1694)	1899369 (50033)
Budding Yeast	7822 (7300)	6378 (5459)	386042 (274523)
Fission Yeast	7559 (5471)	5379 (4569)	285867 (128583)
Worm	6749 (4557)	13653 (7780)	434318 (187819)
Fruit Fly	9851 (7633)	14640 (7674)	534111 (281427)
Zebrafish	10719 (4995)	19782 (3886)	656486 (93450)
Chicken	16336 (2156)	13106 (432)	846899 (11301)
Pig	16775 (1431)	17461 (207)	993064 (5529)
Cow	17565 (2175)	17647 (475)	1115561 (11189)
Dog	17251 (749)	16475 (85)	1028665 (2228)
Mouse	19646 (13771)	24177 (11123)	1444087 (542604)
Rat	19322 (10069)	20201 (5458)	1461152 (279239)
Human	19329 (13796)	19403 (13383)	1566684 (587853)

TABLE C: The number of annotated terms, genes and total annotations in each of the species-specific term-term networks. The values when only including annotations made based on experimental evidence are shown in parentheses.

Comparing community structure identification is an ongoing area of research in the complex systems field and there are multiple proposed methods for comparing two discreet partitions of a set of nodes (e.g. [5, 6]). One we will employ here is the variation of information (VI) [5]:

$$VI(X,Y) = H(X) + H(Y) - 2MI(X,Y) \qquad (1)$$

where $H(X)$ is the Shannon's entropy associated with a partition, X, and $MI(X,Y)$ is the mutual information between two partitions, X and Y. $VI(X,Y)$ represents a distance between the information shared between two partitions, X and Y, with $VI(X,Y) = 0$ indicating identical partitions.

We calculated the VI between the partitions for every pair of species, using only terms common to both partitions when different sets of terms are annotated in the different species. As a comparison we also calculated a VI value for one hundred random shufflings of community assignments in each pair of species. Figure S6(a) shows the ratio of the actual VI value to the average value calculated across the random shufflings. We observe that the actual VI is generally lower than the random values, showing that there is some shared information; however, most comparisons are closer to random ($VI/random \approx 1$) than to “perfect” agreement ($VI/random = 0$), indicating that these term partitions are not identical. Interestingly, there appears to be two main groups of species with a relatively higher level of similarity between them: all species belonging to the Chordata phylum and all species not in the Chordata phylum. The only exception is Zebrafish, which is in the Chordata phylum, but groups with the other species. We also observe a somewhat higher level of agreement between species belonging to the Fungi Kingdom (*A.nidulans*, yeast, budding yeast and fission yeast), and between mouse and rat.

Some of the differences between the species-specific term communities likely reflect real, biological differences in the cellular organization of these systems. However, they may also be due to variations in the annotation practices among groups that supply annotations to GO. To test this we created alternate versions of the species-specific term-term networks wherein we excluded annotations from genes to terms that are based on computational evidence – annotations made in this way may borrow information from other species. We generated the primary partition for each of these networks and compared using VI. We show the results of the analysis in Figure S6(b). We find that when we remove computationally-derived annotations that the VI value is closer to random, especially among the species in the Chordata phylum. This demonstrates that some of the similarity between the partitions can be attributed to computationally-derived annotations.

Overall, we suggest that using partitions of terms in a species-specific context may enhance the results of functional analysis for the model organisms. Furthermore, a more detailed comparison identifying the differences between these communities may uncover important cellular properties specific to the various species, an investigation we leave to future work.

2. SUPPLEMENTAL METHODS

In this section we provide additional information on the methodology used to illustrate the term communities across different resolutions (Figure 2 in the main text) and generate the word clouds representing the biological content of those communities (Figure 4 in the main text).

2.1. Illustrating Community Structure at different resolutions

To better understand the relationships between the communities found at different resolutions, we visualized the uniquely-found term communities with ten or more members for the six lowest values of resolution used ($r = \{0, 0.25, 0.5, 1, 2, 4\}$) (Figure 2 in the main text). We line up the communities found at each resolution and visualize each as a circle whose radius scales as the log of the number of term members found in that community and whose color corresponds to the percentage of "Biological Process", "Molecular Function" and "Cellular Component" terms that belong to that community. In other words, for each community we count the number of members in that community from the "Biological Process" domain and divide by the number of members in the entire "Biological Process" domain. We then do the same things for the other two domains. After these percentages are calculated, within each community they are "normalized" by dividing by the maximum found percentage such that the vector representing that community's domain content has at least one member with a value of one. We can think of these values in terms of a three-part cmy color vector. The normalization process causes communities with an equal percentage from all three primary domains to be colored black (cmy color vector equal to [1,1,1]), and those with members only from one primary domain to be exactly yellow ([0,0,1]) for "Biological Process", cyan ([1,0,0]) for "Molecular Function", or magenta ([0,1,0]) for "Cellular Component".

Between the communities found at adjacent resolutions, we draw a line from a community at a higher resolution to a community at a lower resolution if at least 10% of the members of the community from the higher resolution also belong to the community at the lower resolution. Line thickness scales linearly based on the percentage of members of the community from the higher resolution that belong to the community at the lower resolution. Note that although connections are only made between communities in adjacent resolutions, sometimes the parent community is identical to another community found at an even lower resolution, in which case the connection is made from the child community to the copy of the parent at its lowest found resolution.

2.2. Capturing Biological Information In Word Clouds

In order to easily interpret the contents of our communities we summarize the information contained in each in the form of word clouds using a free word-cloud making program [7]. This program automatically configures the orientation of the words in the clouds, but we manually assign each word a relative size and color to represent that word's statistical enrichment in the community and the primary domain that word is representing in the community, respectively.

To begin, for each community, we determine all the names (e.g. "Biological Process" for GO:0008150) corresponding to the member terms in that community and count the number of times an individual word appears across all these names. Then, for each of these words, we calculate the statistical enrichment (p-value) of the frequency of that word in the community compared to its frequency across the names of all GO terms using the hypergeometric probability:

$$p = P(N \geq N_{wc}|N_w, N_c, N_{tot}) = \sum_{i=N_{wc}}^{min[N_w,N_c]} \frac{\binom{N_c}{i}\binom{N_{tot}-N_c}{N_w-i}}{\binom{N_{tot}}{N_w}}, \quad (2)$$

where N_{wc} is the number of times that word appears in the term names specific to a community, N_w is the number of times that word appears across all term names, N_c is the number of individual words in the term names specific to a community, and N_{tot} is the number of individual words across all term names. We scale the sizes of the words in the word cloud as $-log_{10}(p)$ such that words with the lowest probability of being in the community by chance are given the largest size, and those one might expect by chance are given a size close to zero.

We also colored each word based on the percentage of times the terms that word comes from in a community belongs to each of the primary domains. Specifically, for each instance of a word in the term names, we determine the domain assignment of that term and give that word instance the same domain assignment. To color the word in a community-specific context, we determine the domain assignments made to all instances of that word in the community. We count these instances and divide by the domain assignments made to all words. This will generate a three-part vector representing the percentage of the primary domain represented by the word in the community. We "normalize" this vector by dividing by the maximum found percentage, resulting in a three-part cmy color vector has at least one member with a value of one. The described normalization causes a word with an equal percentage from all three primary domains to be colored black (cmy color vector equal to [1,1,1]), and those with members only from one primary domain to be exactly yellow ([0,0,1]) for "Biological Process", cyan ([1,0,0]) for "Molecular Function", or magenta ([0,1,0]) for "Cellular Component".

File Name	GOC Validation Date	Submission Date	Download Date	MD5Sum
gene_association.aspgd	5/24/2015	5/24/2015	5/28/2015	b2940214b5a843fb3f525e5eb1a6e7ee
gene_association.cgd	5/24/2015	5/24/2015	5/28/2015	54cd14486fcdde91023960250bfeefe9
gene_association.dictyBase	5/3/2015	5/3/2015	5/28/2015	b44858e715d4f5dcad4c52e6fe0067d9
gene_association.ecocyc	5/15/2015	6/26/2014	5/28/2015	0768e9a135be7bcfedd768c732dda6cb
gene_association.fb	4/16/2015	4/16/2015	5/28/2015	eb5d7c4aa99d455311f247e5e8d74a35
gene_association.goa_chicken	5/27/2015	5/27/2015	5/28/2015	33b8d137bda8324ac6ef7a1e9ad7e153
gene_association.goa_cow	5/27/2015	5/27/2015	5/28/2015	f70d5ddc5667e4cbc4fa9d594de09e03
gene_association.goa_dog	5/27/2015	5/27/2015	5/28/2015	625ce72fa19e1c269f3e3211b741ba9d
gene_association.goa_human	5/27/2015	5/27/2015	5/28/2015	ba5001c467183361ef04d43839b10107
gene_association.goa_pig	5/27/2015	5/27/2015	5/28/2015	950af471a45a2144d99bce7946d25616
gene_association.gramene_oryza	2/6/2015	9/22/2009	5/28/2015	773710001d58f4ccff706b4427dad29b
gene_association.mgi	5/22/2015	5/7/2015	5/28/2015	0cd9184fabc478d3ab4fb72c790986cc
gene_association.pombase	5/22/2015	4/24/2015	5/28/2015	4967909301e70cb6231094ea2730b5a3
gene_association.rgd	5/23/2015	5/23/2015	5/28/2015	4048d19c1305f886e7c163ffdeb0f745
gene_association.sgd	5/23/2015	5/23/2015	5/28/2015	9aa0cd4608d8d60cf336796788b444b0
gene_association.tair	4/30/2015	4/30/2015	5/28/2015	fda496ecc5a67fb880dfbb7ecf67df31
gene_association.wb	2/20/2015	9/30/2014	5/28/2015	22f3279a1e6b1ff4bf372631dffd61e4
gene_association.zfin	5/26/2015	5/26/2015	5/28/2015	a2f43c93ff5d8885e040db0e7808e3cb
go.obo.txt	n/a	n/a	5/28/2015	c6213896c02fcf9f940a703ba99a2371

TABLE D: Information about the data downloaded from geneontology.org and used in the analysis presented in this paper.

3. DATA AND CODE AVAILABILITY

All the code used to produce the results presented in this manuscript and supplement are included in a separate supplemental file. In addition, a complete copy of all input files, code, output files and analysis results can be obtained from `https://sites.google.com/a/channing.harvard.edu/kimberlyglass/tools/term-communities`. A list of the gene annotation and ontology files used in this analysis are listed in Table D. The ontology format-version for the go.obo.txt file was 1.2 and the data-version was "releases/2015-05-28". To annotate genes to terms and build bipartite graphs we used all annotations, independent of evidence code. The only exception was in the experimental-annotation only analysis presented in Section 1.6 in this supplement; in this case we only used the EXP, IDA, IPI, IMP, IGI, IEP evidence codes. Gene annotations to terms were propagated up all *is_a* relationships contained in the ontology file.

[1] Kimberly Glass, Edward Ott, Wolfgang Losert, and Michelle Girvan. Implications of functional similarity for gene regulatory interactions. *Journal of the Royal Society, Interface / the Royal Society*, February 2012.

[2] Kimberly Glass and Michelle Girvan. Annotation enrichment analysis: an alternative method for evaluating the functional properties of gene sets. *Scientific reports*, 4, 2014.

[3] The_gene_ontology_consortium. Creating the gene ontology resource: design and implementation. *Genome Res.*, 11(8):1425–1433, August 2001.

[4] Aedín C. Culhane, Markus S. Schröder, Razvan Sultana, Shaita C. Picard, Enzo N. Martinelli, Caroline Kelly, Benjamin Haibe-Kains, Misha Kapushesky, Anne-Alyssa St Pierre, William Flahive, Kermshlise C. Picard, Daniel Gusenleitner, Gerald Papenhausen, Niall O'Connor, Mick Correll, and John Quackenbush. GeneSigDB: a manually curated database and resource for analysis of gene expression signatures. *Nucleic Acids Research*, 40(D1):D1060–D1066, January 2012.

[5] Marina Meila. Comparing clusterings by the variation of information. *Learning Theory and Kernel Machines*, pages 173–187, 2003.

[6] Leon Danon, Albert Díaz-Guilera, Jordi Duch, and Alex Arenas. Comparing community structure identification. *Journal of Statistical Mechanics: Theory and Experiment*, 2005(09):P09008, September 2005.

[7] http://www.softpedia.com/get/office-tools/other-office-tools/ibm-word-cloud-generator.shtml.
