# Supplementary material for: Finding New Order in Biological Functions from the Network Structure of Gene Annotations
Source: PLoS Comput Biol. 2015 Nov 20;11(11):e1004565. doi: 10.1371/journal.pcbi.1004565 (PMC4654495; doi:10.1371/journal.pcbi.1004565)
Supplement: S1 Code — This file contains the input human annotation files and all the code needed to reproduce the analyses and figures presented in this manuscript. The complete collection of intermediate files (such as the predicted term-term networks, word clouds for all communities, etc), can be obtained from [34]. (TGZ) [file pcbi.1004565.s004.tgz › TermCommunities_code/MakeCloudFiles/IBM Word Cloud/license/cs.html]

Software License

Mezinárodní licenèní smlouva pro pøedbìžnì uvolnìné programy  
  
Èást 1 - Obecná ustanovení  
  
TATO MEZINÁRODNÍ LICENÈNÍ SMLOUVA NA PØEDBÌŽNÌ UVOLNÌNÉ
PROGRAMY ("SMLOUVA") JE PRÁVNÍM DOKUMENTEM MEZI VÁMI A IBM.
STAŽENÍM, INSTALACÍ, ZKOPÍROVÁNÍM, PØÍSTUPEM K PROGRAMU NEBO POUŽITÍM
PROGRAMU VYJADØUJETE SVÙJ SOUHLAS S TOUTO SMLOUVOU. JESTLIŽE
SOUHLASÍTE S TÌMITO PODMÍNKAMI JMÉNEM JINÉ FYZICKÉ NEBO PRÁVNICKÉ
OSOBY ÈI JINÉHO PRÁVNÍHO SUBJEKTU, PROHLAŠUJETE A ZARUÈUJETE, ŽE
MÁTE POTØEBNÉ OPRÁVNÌNÍ K TOMU, ABYSTE TUTO FYZICKOU ÈI
PRÁVNICKOU OSOBU ÈI PRÁVNÍ SUBJEKT VÁZALI TÌMITO PODMÍNKAMI.   
  
"Pøedbìžné vydání" je vydání programu, který (1) mùže být
ještì ve fázi vývoje (a proto je potenciálnì nespolehlivý), nebo
(2) již není ve fázi vývoje, avšak dosud nebyl uèinìn komerènì
dostupným pro uživatele.   
  
"IBM" je spoleènost International Business Machines
Corporation nebo nìkterá z jejích dceøiných spoleèností.   
  
"Licenèní informace" ("LI") je dokument, který uvádí
informace a podmínky specifické pro program. Dokument Licenèní
informace k programu je k dispozici v nìkterém souboru v adresáøi s
programem, je dostupný prostøednictvím systémového pøíkazu nebo je k
dispozici ve formì pøíruèky pøipojené k programu.   
  
"Program" je jedna nebo více níže uvedených položek, vèetnì
originálu a veškerých úplných nebo dílèích kopií: 1) strojovì èitelné
instrukce a data, 2) èlovìkem èitelné softwarové komponenty, 3)
audiovizuální obsah (jako jsou obrázky, text, nahrávky nebo grafika), 4)
související licencované materiály, 5) licenèní dokumenty nebo klíèe,
(6) související dokumentace a (7) jakákoliv zdokonalení,
aktualizace nebo materiály, které se IBM mùže dle vlastního uvážení
rozhodnout poskytnout Vám jako podporu (jak je popsáno níže).   
  
"Vy" a "Váš, Vaše" se vztahuje buï na jednotlivou osobu,
nebo na jeden právní subjekt.   
  
Tato smlouva zahrnuje Èást 1 - Všeobecné podmínky, Èást 2 -
Podmínky specifické pro jednotlivé zemì (jsou-li nìjaké) a dokument
Licenèní informace a tvoøí smlouvu mezi Vámi a IBM ohlednì užívání
programu. Nahrazuje veškerá pøedchozí ústní nebo písemná ujednání
mezi Vámi a IBM ohlednì užívání programu. Podmínky Èásti 2 a
dokumentu Licenèní informace mohou nahrazovat nebo pozmìòovat
podmínky uvedené v Èásti 1.   
  
1. Licence  
  
Program je vlastnictvím IBM nebo nìkterého dodavatele IBM a
je Vám licencován, nikoli prodáván.   
  
IBM Vám udìluje omezenou, nevýhradní, nepøenosnou licenci
na stažení, instalaci a užívání programu bìhem období
hodnocení, a to výhradnì pro úèely interního testování a hodnocení a
poskytnutí zpìtné vazby IBM.   
  
Jste oprávnìni vytvoøit záložní kopii programu za úèelem
podpory takového užívání. Nejste oprávnìni užívat program pro úèely
produktivního užívání, ani nejste oprávnìni distribuovat program nebo
kterékoliv jeho èásti. Nejste oprávnìni zmìnit program nebo vytváøet
odvozená díla z programu. Podmínky této licence se vztahují na
každou kopii, kterou vytvoøíte. Jste povinni reprodukovat všechny
copyrightové výhrady a veškeré jiné výhrady týkající se vlastnických
práv na každé kopii nebo dílèí kopii programu.   
  
Zavazujete se, že budete 1) vést záznamy o všech kopiích
programu a 2) zajistíte, aby kdokoliv, kdo užívá program (k nìmuž
pøistupuje buï lokálnì, nebo vzdálenì), tak èinil výhradnì pro úèely
Vašeho oprávnìného užívání a v souladu s podmínkami této smlouvy.   
  
Nejste oprávnìni: 1) používat, kopírovat, modifikovat,
pøenášet nebo distribuovat program s výjimkou, jak je uvedeno v této
smlouvì; 2) zpìtnì pøekládat, kompilovat nebo jinak pøekládat
program do èlovìkem èitelné formy nebo do jiného programového
jazyka (s výjimkou, jak mùže výslovnì povolovat zákon bez možnosti
smluvního zøeknutí se); 3) sublicencovat nebo pronajímat program;
nebo 4) používat program na bázi "service-bureau".   
  
Tato licence Vás neopravòuje k pøijetí dokumentace v
tištìné formì, podpory, telefonické asistence nebo zdokonalení èi
aktualizací programu (souhrnnì "podpora") od IBM, aèkoliv se IBM mùže,
výhradnì dle vlastního uvážení, rozhodnout, že Vám takovou podporu
poskytne. Jakákoliv zdokonalení, aktualizace a veškeré další
materiály, které IBM poskytne v rámci takové podpory, jsou považovány
za èást programu, a proto se na nì vztahují podmínky této
smlouvy.   
  
PROGRAM MÙŽE OBSAHOVAT BLOKOVACÍ ZAØÍZENÍ, KTERÉ ZABRÁNÍ V
JEHO POUŽITÍ PO UKONÈENÍ OBDOBÍ HODNOCENÍ. NESMÍTE PORUŠIT TOTO
BLOKOVACÍ ZAØÍZENÍ NEBO PROGRAM. MÌLI BYSTE ZAVÉST TAKOVÁ OPATØENÍ,
KTERÁ ZAMEZÍ MOŽNÉ ZTRÁTÌ DAT POTÉ, CO PROGRAM NEBUDETE MOCI DÁLE
POUŽÍVAT.  
  
2. Trvání  
  
Období hodnocení zaèíná okamžikem, kdy vyjádøíte svùj
souhlas s podmínkami této smlouvy, a konèí v datum, které nastane
døíve: 1) datum ukonèení (je-li nìjaké) uvedené v dokumentu
Licenèní informace, 2) datum, kdy se program sám automaticky
zablokuje, 3) datum, kdy IBM uèiní program komerènì dostupným. Vaše
licence na program konèí ke konci období hodnocení a Vy jste
povinni znièit program a veškeré kopie z nìj vytvoøené bìhem 10 dní
od ukonèení období hodnocení.   
  
Na Vaše užívání programu po dobu období hodnocení se
nevztahuje žádný poplatek.   
  
IBM je oprávnìna Vaši licenci ukonèit, nejednáte-li v
souladu s podmínkami této smlouvy. Jestliže tak IBM uèiní, jste
povinni znièit všechny kopie programu.   
  
3. Práva na data  
  
Postupujete IBM všechna práva, podíly a právní nároky
(vèetnì vlastnictví autorských práv), pokud jde o jakákoliv data,
návrhy nebo písemné materiály, které se 1) vztahují k programu a
které 2) poskytnete IBM. Pokud to bude IBM vyžadovat, podepíšete
pøíslušný dokument, kterým tato práva postoupíte. V míøe, na kterou
se nevztahují práva udìlená v první vìtì tohoto Oddílu 3,
udìlujete IBM, s ohledem na veškeré nápady, know-how, koncepce,
techniky, invence, objevy nebo zdokonalení (patentované èi
nepatentované), které se vztahují k programu a které poskytnete IBM,
nevýhradní, neodvolatelnou, neomezenou, celosvìtovou, bezplatnou
licenci a práva na zahrnutí výše uvedených položek do jakéhokoliv
produktu a služby a na užívání, výrobu a prodej každého takového
produktu èi služby, a zavazujete se, že povolíte ostatním èinit vše
výše uvedené.   
  
4. Vylouèení záruky  
  
V SOULADU S VEŠKERÝMI ZÁKONNÝMI ZÁRUKAMI, JSOU-LI NÌJAKÉ,
KTERÉ NELZE VYLOUÈIT, IBM VYLUÈUJE VEŠKERÉ ZÁRUKY NEBO PODMÍNKY,
VYJÁDØENÉ ÈI ODVOZENÉ VÈETNÌ, BEZ OMEZENÍ, ODVOZENÝCH ZÁRUK NEBO
PODMÍNEK USPOKOJIVÉ KVALITY, PRODEJNOSTI, VHODNOSTI PRO URÈITÝ ÚÈEL
A ZÁRUK PRÁVNÍHO NÁROKU A NEPORUŠENÍ PRÁV TØETÍCH STRAN S
OHLEDEM NA PROGRAM NEBO TECHNICKOU PODPORU (JE-LI NÌJAKÁ).   
  
Vylouèení záruky se vztahuje rovnìž na všechny vývojáøe a
dodavatele IBM.   
  
Výrobci, dodavatelé nebo vydavatelé programù, které
nepocházejí od IBM, mohou poskytovat své vlastní záruky.   
  
5. Omezení odpovìdnosti  
  
Mohou nastat okolnosti, kdy Vám z dùvodu neplnìní závazku
na stranì IBM èi z jiného dùvodu vznikne nárok na náhradu
škody ze strany IBM. Bez ohledu na základnu, na jejímž základì
mùžete být oprávnìni vznést nárok na náhradu škody od IBM (vèetnì
závažného porušení smlouvy, nedbalosti, uvedení v omyl nebo jiné
porušení smluvního èi mimozávazkového vztahu), není IBM odpovìdná za
více než: 1) škody v dùsledku fyzického úrazu (vèetnì úmrtí) a
škod na nemovitém a movitém majetku a 2) èástku veškerých jiných
skuteèných pøímých škod až do celkové výše 25.000 USD (nebo
ekvivalentu v místní mìnì) za všechny nároky na náhradu škody úhrnem.
Uvedené omezení odpovìdnosti se vztahuje rovnìž na vývojáøe
programu a dodavatele IBM. Jedná se o maximum, za které jsou
vývojáøi, dodavatelé a IBM kolektivnì odpovìdni.   
  
IBM ANI JEJÍ VÝVOJÁØI PROGRAMU NEJSOU ZA ŽÁDNÝCH OKOLNOSTÍ
ODPOVÌDNI ZA ŽÁDNÉ Z NÍŽE UVEDENÝCH ŠKOD, ANI KDYŽ BYLI O MOŽNOSTI
JEJICH VZNIKU PØEDEM INFORMOVÁNI:  
  
1. ZTRÁTA NEBO POŠKOZENÍ DAT;  
2. ZVLÁŠTNÍ, NAHODILÉ, NEPØÍMÉ, ZVLÁŠTNÍ ŠKODY, NÁSLEDNÉ
EKONOMICKÉ ŠKODY A ŠKODY SOUVISEJÍCÍ S TRESTNÝM ÈINEM; NEBO  
3. UŠLÝ ZISK, ZTRÁTA OBCHODNÍCH TRANSAKCÍ, VÝNOSU, DOBRÉHO
JMÉNA NEBO PØEDPOKLÁDANÝCH ÚSPOR.   
  
6. Všeobecné  
  
1. Nic v této smlouvì nemá vliv na žádná zákonná práva
spotøebitelù, kterých se nelze zøeknout nebo je smluvnì omezit.   
2. V pøípadì, že nìkteré ustanovení této smlouvy bude
shledáno neplatným nebo nevymahatelným, zbývající ustanovení této
smlouvy zùstávají plnì platná a úèinná.   
3. Nejste oprávnìni exportovat program, ani provádìt s
programem žádné akce, které by porušovaly pøíslušné vývozní pøedpisy
a naøízení.   
4. Zavazujete se, že spoleènosti International Business
Machines Corporation a jejím poboèkám povolíte ukládání a používání
Vašich obchodních kontaktních informací vèetnì jmen, podnikových
telefonních èísel a podnikových e-mailových adres, a to kdekoliv, kde
IBM provádí svou obchodní èinnost. Takové informace budou
zpracovány a použity ve spojení s naším obchodním vztahem a mohou být
poskytnuty dodavatelùm, kteøí jednají jménem IBM, obchodním partnerùm
IBM, kteøí nabízejí, prodávají a podporují urèité produkty a
služby IBM a zmocnìným zástupcùm a poboèkám spoleènosti
International Business Machines Corporation, a to pro užívání, které bude
v souladu s takovým obchodním vztahem.   
5. IBM nezaruèuje, že jakákoliv verze programu, která bude
formálnì uvolnìna nebo uèinìna komerènì dostupnou (bude-li nìjaká),
bude podobná nebo kompatibilní s pøedbìžnì uvolnìnou verzí
programu.   
6. Ani Vy, ani IBM nepodáte soudní žalobu na základì této
smlouvy pozdìji než dva roky od vzniku pøíèiny žaloby, s výjimkou
pøípadu, kdy místní právní pøedpisy stanoví jinak bez možnosti
smluvního zøeknutí se nebo omezení.   
7. Žádná ze smluvních stran není odpovìdná za nesplnìní
jakýchkoliv závazkù a povinností v dùsledku pøíèin, které jsou mimo
její kontrolu.   
8. Tato smlouva nezakládá pro žádnou tøetí stranu žádné
právo nebo pøíèinu soudní žaloby a IBM není odpovìdná za žádné
nároky na náhradu škody vznesené tøetími stranami vùèi Vám, s
výjimkou (jak je povoleno v bodu Omezení odpovìdnosti výše)
odškodnìní za fyzický úraz (vèetnì úmrtí) nebo škody na nemovitém nebo
movitém majetku, za které je IBM právnì odpovìdná.   
9. Nejste oprávnìni pøevést tuto smlouvu, zcela ani zèásti,
bez pøedchozího písemného souhlasu IBM. Jakýkoliv pokus o
takové pøevedení je neplatný.   
  
7. Rozhodné právo a jurisdikce  
  
Rozhodné právo, kterým se smlouva øídí  
  
Obì smluvní strany jsou srozumìny s tím, že se veškeré Vaše
povinnosti, práva a závazky vyplývající ze smlouvy nebo se nìjakým
zpùsobem vztahující k pøedmìtu smlouvy, bez ohledu na rozpor
právních principù, budou øídit, interpretovat a vymáhat v souladu s
právními pøedpisy zemì, v níž jste získali licenci na program.   
  
Konvence OSN o mezinárodním prodeji zboží se neaplikuje.   
  
Jurisdikce  
  
Veškeré povinnosti, práva a závazky podléhají soudùm zemì,
v níž jste získali licenci na program.   
  
Èást 2 - Podmínky specifické pro jednotlivé státy  
  
EVROPA, STØEDNÍ VÝCHOD, AFRIKA (EMEA)  
Práva na data (Oddíl 3): V zemích EMEA se podmínky tohoto
oddílu nahrazují v celé úplnosti textem:   
  
Postupujete IBM všechna práva, podíly a právní nároky na
celém svìtì (vèetnì vlastnictví autorských práv), pokud jde o
jakákoliv data, návrhy nebo písemné materiály, které se 1) vztahují k
Vašemu užívání programu a které 2) poskytnete IBM. Po celou dobu,
na kterou jsou práva a nároky postoupeny, zahrnuje takové
postoupení, mimo jiné, postoupení práva na vytváøení a práva nechat
vytváøet odvozená díla z písemných materiálù a používat, provádìt,
reprodukovat, pøenášet, zobrazovat, vykonávat, pøevádìt, distribuovat a
poskytovat licence k písemným materiálùm a takovým odvozeným dílùm na
jakémkoliv médiu èi pomocí jakékoliv distribuèní technologie a
udìlovat ostatním nìkterá nebo všechna práva zde uvedená. Pokud to
bude IBM vyžadovat, podepíšete pøíslušný dokument, kterým tato
práva postoupíte. S ohledem na veškeré nápady, know-how,
koncepce, techniky, invence, objevy nebo zdokonalení (patentované èi
nepatentované), které se vztahují k programu a které vytvoøíte Vy nebo Vaši
zamìstnanci bìhem období hodnocení, udìlujete IBM nevýhradní,
neodvolatelnou, neomezenou, celosvìtovou, bezplatnou licenci a práva na
zahrnutí výše uvedených položek do jakéhokoliv produktu a služby a
na užívání, výrobu a prodej každého takového produktu èi
služby, a zavazujete se, že povolíte ostatním èinit vše výše
uvedené. Žádná ze smluvních stran nebude úètovat druhé smluvní
stranì žádné poplatky za práva na data nebo jakékoliv dílo
realizované v dùsledku této smlouvy.   
  
Vylouèení záruky (Oddíl 4): V zemích Evropské unie se na
zaèátek tohoto oddílu pøidává text:  
  
V Evropské unii mají zákazníci zákonná práva vyplývající z
pøíslušné národní legislativy, jež upravuje prodej spotøebního zboží.
Na taková práva se nevztahují ustanovení tohoto Oddílu 4.  
  
Z125-5544-03 (10/2005)  
LICENÈNÍ INFORMACE  
  
Níže uvedené programy jsou licencovány na základì
následujících ustanovení a podmínek, které doplòují ustanovení a podmínky
smlouvy Mezinárodní licenèní smlouva pro pøedèasnì uvolnìné
programy.  
  
Jméno programu: alphaWorks Emerging Technology  
Èíslo programu: N/A  
  
Urèené provozní prostøedí  
  
Specifikace programu a informace o urèeném provozním
prostøedí jsou k dispozici v dokumentaci, která je pøipojena k
programu (je-li dostupná) jako soubor "Readme", nebo v jiných
informacích zveøejnìných IBM, jako jsou dokumenty s ohlášením produktù.  
  
Období hodnocení  
  
Období hodnocení zaèíná v dan, kdy vyjádøíte svùj souhlas s
podmínkami této smlouvy, a konèí po 90 dnech.  
  
D/N: L-JLCO-6HQ6QK  
P/N: L-JLCO-6HQ6QK   
