# Supplementary material for: Finding New Order in Biological Functions from the Network Structure of Gene Annotations
Source: PLoS Comput Biol. 2015 Nov 20;11(11):e1004565. doi: 10.1371/journal.pcbi.1004565 (PMC4654495; doi:10.1371/journal.pcbi.1004565)
Supplement: S1 Code — This file contains the input human annotation files and all the code needed to reproduce the analyses and figures presented in this manuscript. The complete collection of intermediate files (such as the predicted term-term networks, word clouds for all communities, etc), can be obtained from [34]. (TGZ) [file pcbi.1004565.s004.tgz › TermCommunities_code/MakeCloudFiles/IBM Word Cloud/license/de.html]

Software License

Internationale Nutzungsbedingungen für Vorabreleases von
Programmen  
  
Teil 1 - Allgemeine Bedingungen  
  
Die Internationalen Nutzungsbedingungen für Vorabreleases
von Programmen ("Vereinbarung") sind eine rechtsgültige
Vereinbarung zwischen Ihnen und IBM. Durch Herunterladen, Installieren,
Kopieren oder Zugreifen auf das Programm oder die Nutzung des
Programms erklären Sie sich mit den Bedingungen dieser Vereinbarung
einverstanden. Wenn Sie diese Bedingungen im Auftrag einer anderen Person
oder eines Unternehmens oder einer anderen juristischen Person
akzeptieren, gewährleisten Sie, dass Sie berechtigt sind, diese Person,
dieses Unternehmen oder diese juristische Person zur Einhaltung
dieser Bedingungen zu verpflichten.  
  
"Vorabrelease" ist ein Release eines Programms, das (1)
sich noch in der Entwicklung befinden kann (und daher potenziell
störungsanfällig ist) oder (2) sich nicht mehr in der Entwicklung befindet,
aber auch nicht für Benutzer zur kommerziellen Nutzung zur
Verfügung gestellt wurde.  
  
"IBM" steht für International Business Machines Corporation
oder eine IBM Tochtergesellschaft.  
  
"Lizenzinformation" ("LI") ist ein Dokument, das
programmspezifische Informationen und Bedingungen enthält. Die
Lizenzinformation des Programms wird in einer Datei im Verzeichnis des
Programms zur Verfügung gestellt, sie kann über einen entsprechenden
Systembefehl abgerufen werden oder ist dem Programm als Broschüre
beigelegt.   
  
Unter "Programm" ist das Originalprogramm sowie
vollständige oder teilweise Kopien davon zu verstehen. Ein Programm
besteht aus: 1) maschinenlesbaren Instruktionen und Daten, 2)
visuell lesbaren Softwarekomponenten, 3) audiovisuellen Inhalten
(z. B. Abbildungen, Texte, Aufzeichnungen oder Bilder), 4)
zugehörigem Lizenzmaterial, 5) Lizenznutzungsdokumenten oder -
schlüsseln, 6) zugehöriger Dokumentation und 7) funktionalen
Erweiterungen, Updates oder Materialien, die IBM Ihnen nach eigenem
Ermessen zur Unterstützung bereitstellen kann (siehe die
nachfolgende Beschreibung).   
  
"Sie" und "Ihr" bezieht sich entweder auf eine Einzelperson
oder eine einzelne juristische Person.   
  
Diese Vereinbarung umfasst Teil 1 - Allgemeine Bedingungen,
Teil 2 - Länderspezifische Bedingungen (sofern vorhanden) sowie
die Lizenzinformation und stellt die vollständige Vereinbarung
zwischen Ihnen und IBM zur Nutzung des Programms dar. Sie ersetzt
alle zuvor getroffenen mündlichen oder schriftlichen Absprachen
zwischen Ihnen und IBM in Bezug auf die Nutzung des Programms. Die
Bedingungen von Teil 2 und die Lizenzinformation können die Bedingungen
in Teil 1 ersetzen oder ergänzen.   
  
1. Lizenz  
  
Das Programm ist Eigentum von IBM oder eines IBM
Lieferanten, es ist urheberrechtlich geschützt und wird zur Nutzung
überlassen (lizenziert, nicht verkauft).   
  
IBM erteilt Ihnen eine eingeschränkte, nicht
ausschließliche, nicht übertragbare Lizenz zum Herunterladen, Installieren
und zur Nutzung des Programms während des Bewertungszeitraums
ausschließlich für interne Test- und Bewertungszwecke und für
Rückmeldungen an IBM.   
  
Sie dürfen eine Sicherungskopie des Programms zur
Unterstützung dieser Nutzung erstellen. Sie sind nicht berechtigt, das
Programm für produktive Zwecke zu nutzen oder das Programm oder
Teile des Programms weiterzugeben. Sie dürfen das Programm weder
ändern noch abgeleitete Werke davon erstellen. Die Bedingungen
dieser Lizenz gelten für jede Kopie. Sie verpflichten sich, auf
jeder Kopie oder Teilkopie des Programms den Copyrightvermerk und
alle anderen Eigentumshinweise anzubringen.   
  
Sie verpflichten sich, 1) ein Verzeichnis aller
Programmkopien anzulegen, und 2) gewährleisten, dass das Programm
(unabhängig davon, ob der Zugriff lokal oder von einem fernen System
aus erfolgt) nur im Rahmen der autorisierten Nutzung und in
Übereinstimmung mit den Bedingungen dieser Vereinbarung genutzt wird.   
  
Es ist Ihnen untersagt, 1) das Programm abweichend von den
Bedingungen dieser Vereinbarung zu nutzen, zu kopieren, zu ändern, zu
übertragen oder weiterzugeben; 2) das Programm umzuwandeln (reverse
assemble, reverse compile) oder auf andere Weise in eine lesbare Form
oder eine andere Programmiersprache zu übersetzen (sofern eine
solche Umwandlung nicht durch ausdrückliche gesetzliche Regelung
unabdingbar vorgesehen ist); 3) das Programm in Unterlizenz zu
vergeben, zu vermieten oder zu verleasen; oder 4) das Programm als
Bestandteil eines Serviceunternehmens zu nutzen.   
  
Diese Lizenz beinhaltet nicht das Recht, von IBM
Hardcopydokumentation, Unterstützung, telefonische Beratung, funktionale
Erweiterungen oder Updates für das Programm ("Unterstützung" genannt)
einzufordern, obwohl IBM nach eigenem Ermessen Unterstützung dieser Art
leisten kann. Alle funktionalen Erweiterungen, Updates oder
sonstigen Materialien, die von IBM im Rahmen der Unterstützung
bereitgestellt werden, sind Teil des Programms und unterliegen daher
dieser Vereinbarung.   
  
Das Programm kann eine Inaktivierungseinheit beinhalten,
die eine Nutzung des Programms nach Ablauf des
Bewertungszeitraums verhindert. Es ist Ihnen untersagt, die
Inaktivierungseinheit oder das Programm in irgendeiner Weise zu manipulieren. Sie
sollten entsprechende Vorkehrungsmaßnahmen treffen, um
Datenverluste zu vermeiden, wenn das Programm nicht mehr verwendet werden
kann.   
  
2. Laufzeit  
  
Der Bewertungszeitraum beginnt, wenn Sie dieser
Vereinbarung zustimmen, und endet 1) an dem in der Lizenzinformation
angegebenen Datum (sofern vorhanden); 2) an dem Datum, zu dem sich das
Programm automatisch selbst inaktiviert; oder 3) wenn IBM das
Programm kommerziell verfügbar macht, abhängig davon, welches
Ereignis zuerst eintritt. Ihre Lizenz für das Programm endet mit dem
Ablauf des Bewertungszeitraums; Sie müssen das Programm und alle
davon erstellten Kopien innerhalb von zehn (10) Tagen nach Ablauf
des Bewertungszeitraums vernichten.   
  
Während des Bewertungszeitraums fallen keine
Nutzungsgebühren für das Programm an.   
  
IBM kann Ihre Lizenz kündigen, wenn Sie die Bedingungen
dieser Vereinbarung nicht einhalten. In diesem Fall sind Sie
verpflichtet, alle Kopien des Programms zu vernichten.   
  
3. Rechte an Daten  
  
Sie überlassen IBM alle Rechte (inklusive aller infrage
kommenden Nutzungs- und Verwertungsrechte) und Rechtsansprüche auf
alle Daten, Vorschläge und schriftlichen Materialien, 1) die
sich auf das Programm beziehen und 2) die Sie IBM zur Verfügung
stellen. Auf Verlangen von IBM erklären Sie sich bereit, ein
entsprechendes Dokument zur Übertragung solcher Rechte zu unterzeichnen.
Zusätzlich zu den im ersten Satz dieses Abschnitts abgetretenen
Rechten gewähren Sie IBM eine nicht ausschließliche,
unwiderrufliche, uneingeschränkte, weltweit geltende und gebührenfreie
Berechtigung und Lizenz, Ideen, Know-how, Konzepte, Verfahren,
Erfindungen, Entdeckungen oder Verbesserungen (ob patentierbar oder
nicht), die sich auf das Programm beziehen und die Sie IBM zur
Verfügung stellen, in ein Produkt oder einen Service zu integrieren
und dieses Produkt oder diesen Service zu nutzen, herzustellen
bzw. anzubieten und zu vertreiben und Dritten ebenfalls diese
Rechte zu erteilen.   
  
4. Gewährleistungsausschluss  
  
Vorbehaltlich einer gesetzlichen Gewährleistung, die nicht
ausgeschlossen werden kann, gibt IBM keine ausdrückliche oder
stillschweigende Gewährleistung für zufriedenstellende Qualität oder die
Handelsüblichkeit, die Eignung für einen bestimmten Zweck oder die Freiheit
von Rechten Dritter in Bezug auf das Programm oder die
technische Unterstützung.  
  
Dieser Gewährleistungsausschluss gilt auch für die IBM
Programmentwickler und Lieferanten.   
  
Hersteller, Lieferanten oder Herausgeber von Nicht-IBM
Programmen können ihre eigenen Gewährleistungen mitliefern.  
  
5. Haftungsbegrenzung  
  
Soweit Sie aus Verschulden von IBM oder aus sonstigen
Gründen von IBM Schadenersatz fordern, ist die Haftung von IBM
unabhängig von der Rechtsgrundlage, auf der der Schadensersatzanspruch
an IBM beruht (einschließlich Verletzung wesentlicher
Vertragspflichten, Fahrlässigkeit, unrichtiger Angaben oder anderer Ansprüche
aus dem Vertrag oder auf Grund unerlaubter Handlungen) und
außer in Fällen der gesetzlich zwingenden Haftung, begrenzt auf
1) Personenschäden (einschließlich Tod) und Schäden an
Immobilien und beweglichen Sachen und 2) bei anderen direkten Schäden
bis zu einem Gesamtbetrag von 25.000 US-Dollar (oder den
entsprechenden Betrag in der Landeswährung) für alle
Schadensersatzansprüche insgesamt. Diese Haftungsbegrenzung gilt auch für die IBM
Programmentwickler und Lieferanten. Dies ist der Höchstbetrag, für den IBM und
diese gemeinsam haftbar gemacht werden können.   
  
Auf keinen Fall sind IBM oder ihre Programmentwickler und
Lieferanten in folgenden Fällen haftbar, auch wenn auf die Möglichkeit
solcher Schäden hingewiesen wurde:   
  
1. Verlust oder Beschädigung von Daten;   
2. unmittelbare, mittelbare oder sonstige Folgeschäden oder
andere wirtschaftliche Folgeschäden; oder   
3. entgangene Gewinne, Geschäftsabschlüsse, Umsätze,
Schädigung des guten Namens oder Verlust erwarteter Einsparungen.   
  
6. Allgemeines  
  
1. Gesetzlich unabdingbare Verbraucherschutzrechte gehen
den nachfolgenden Bestimmungen vor.  
2. Falls eine der Bedingungen dieser Vereinbarung im Rahmen
des geltenden Rechts ungültig oder undurchführbar ist, sind die
übrigen Bedingungen dieser Vereinbarung davon nicht betroffen und
gelten weiterhin in vollem Umfang.   
3. Sie dürfen das Programm nicht exportieren oder Maßnahmen
in Bezug auf das Programm ergreifen, die gegen geltende
Außenhandelsgesetze verstoßen.   
4. Sie erklären sich damit einverstanden, dass die
International Business Machines Corporation und ihre
Tochtergesellschaften Ihre Geschäftskontaktinformationen, einschließlich Namen,
Telefonnummern und E-Mail-Adressen, speichern und nutzen dürfen. Diese
Informationen werden im Rahmen der bestehenden Geschäftsbeziehung
verarbeitet und genutzt und dürfen an Subunternehmer, IBM Business
Partner, die bestimmte IBM Produkte und Services anbieten,
vermarkten und unterstützen, und Bevollmächtigte der International
Business Machines Corporation und ihrer Tochtergesellschaften zum
Zwecke der gemeinsamen Geschäftsaktivitäten weitergegeben werden.   
5. IBM gewährleistet nicht, dass eine Version des
Programms, die offiziell freigegeben oder kommerziell verfügbar
gemacht wird, mit den Vorabreleases identisch oder kompatibel sein
wird.   
6. Sowohl Sie als auch IBM verpflichten sich, keine Klage
im Rahmen dieser Vereinbarung später als zwei Jahre nach
Auftreten des Klagegegenstands einzureichen, soweit keine anders
lautenden gesetzlichen Regelungen unabdingbar vorgesehen sind.   
7. Weder Sie noch IBM sind für die Nichterfüllung von
vertraglichen Verpflichtungen aus Gründen haftbar, die von den
Vertragspartnern nicht beeinflusst werden können.   
8. Aus dieser Vereinbarung ergibt sich kein Recht auf Klage
oder Klagegegenstand für Dritte, und IBM ist nicht haftbar für
Ansprüche Dritter gegen Sie, die nicht im zuvor aufgeführten
Abschnitt zur Haftungsbegrenzung spezifiziert sind, und für
Personenschäden (einschließlich Tod) und Schäden an Immobilien und
beweglichen Sachen, für die IBM nach den gesetzlichen Bestimmungen
haftbar ist.   
9. Sie dürfen diese Vereinbarung weder ganz noch
auszugsweise ohne die vorherige schriftliche Zustimmung von IBM
abtreten. Entsprechende Versuche sind nichtig.   
  
7. Geltendes Recht und Rechtsprechung  
  
Geltendes Recht  
  
Sowohl Sie als auch IBM sind damit einverstanden, dass die
Gesetze des Landes zur Anwendung kommen, in dem Sie die
Programmlizenz erwerben, um die Rechte, Pflichten und Verpflichtungen von
Ihnen und IBM, die sich aus dem Inhalt dieser Vereinbarung
ergeben oder in irgendeiner Weise damit in Zusammenhang stehen, zu
regeln, zu interpretieren und durchzuführen, ungeachtet
unterschiedlicher Rechtsgrundlagen.   
  
Die Vertragskonvention der Vereinten Nationen für den
internationalen Warenverkauf kommt nicht zur Anwendung.   
  
Rechtsprechung  
  
Alle Rechte, Pflichten und Verpflichtungen von IBM
unterliegen der Rechtsprechung des Landes, in dem die Sie die
Programmlizenz erwerben.   
  
Teil 2 - Länderspezifische Bedingungen  
  
EUROPA, NAHER UND MITTLERER OSTEN, AFRIKA (EMEA)  
Rechte an Daten (Abschnitt 3): In Europa, im Nahen und
Mittleren Osten und in Afrika (EMEA) wird dieser Abschnitt
vollständig durch folgende Regelungen ersetzt:  
  
Sie überlassen IBM weltweit alle Rechte (inklusive aller
infrage kommenden Nutzungs- und Verwertungsrechte) und
Rechtsansprüche auf alle Daten, Vorschläge und schriftlichen Materialien,
1) die sich auf Ihre Nutzung des Programms beziehen und 2) die
Sie IBM zur Verfügung stellen. Die Überlassung solcher Rechte
umfasst, ist aber nicht beschränkt auf die Überlassung der Rechte
zur Erstellung abgeleiteter Werke der schriftlichen Materialien
und zur Nutzung, Ausführung, Vervielfältigung, Übertragung,
Anzeige, Weitergabe und Lizenzierung der schriftlichen Materialien
und solcher abgeleiteten Werke auf einem beliebigen Medium oder
mit einer beliebigen Verteilungstechnologie. Eingeschlossen ist
ferner das Recht zur Überlassung einiger oder aller in diesem
Dokument gewährten Rechte an andere für die Gültigkeitsdauer solcher
Rechte und Rechtsansprüche. Auf Verlangen von IBM erklären Sie
sich bereit, ein entsprechendes Dokument zur Übertragung solcher
Rechte zu unterzeichnen. In Bezug auf die Ideen, das Know-how, die
Konzepte, Verfahren, Erfindungen, Entdeckungen oder Verbesserungen
(ob patentierbar oder nicht), die sich auf das Programm
beziehen und die Sie oder Ihre Mitarbeiter während des
Bewertungszeitraums erarbeiten, gewähren Sie IBM eine nicht ausschließliche,
unwiderrufliche, uneingeschränkte, weltweit geltende und gebührenfreie
Berechtigung und Lizenz, diese in ein Produkt oder einen Service zu
integrieren und dieses Produkt oder diesen Service zu nutzen,
herzustellen bzw. anzubieten und zu vertreiben und Dritten ebenfalls
diese Rechte zu erteilen. Keine der beiden Parteien wird der
anderen Partei die Rechte an Daten oder die als Ergebnis dieser
Vereinbarung ausgeführten Tätigkeiten in Rechnung stellen.   
  
Gewährleistungsausschluss (Abschnitt 4): In der
Europäischen Union wird am Anfang dieses Abschnitts Folgendes eingefügt:  
  
In der Europäischen Union sind für Konsumenten unter den
geltenden nationalen rechtlichen Bestimmungen Rechte für den Verkauf
von Konsumgütern definiert. Diese Rechte sind von den
Bestimmungen in diesem Abschnitt 4 nicht betroffen.   
  
Haftungsbegrenzung (Abschnitt 5): In Österreich und in der
Schweiz wird dieser Abschnitt vollständig durch folgende Regelungen
ersetzt:  
  
Vorausgesetzt, dass keine anderweitigen verbindlichen
Rechtsbestimmungen gelten:   
  
1. Die Haftung von IBM für Schäden und Verluste, die als
Folge der Erfüllung der Verpflichtungen dieser Vereinbarung oder
in Zusammenhang mit dieser Vereinbarung verursacht wurden oder
die auf einem anderen, mit dieser Vereinbarung in Zusammenhang
stehenden Ursache beruhen, ist begrenzt auf die Kompensation der
Schäden und Verluste, die als unmittelbare und direkte Folge der
Nichterfüllung solcher Verpflichtungen (bei Verschulden von IBM) oder
durch die genannte Ursache entstanden und belegt sind. Der
Höchstbetrag für Schadensersatzansprüche ist in jedem Fall auf 25.000
Euro begrenzt.  
Die obige Einschränkung gilt nicht für Personenschäden
(einschließlich Tod) und für direkte Schäden an Immobilien und beweglichen
Sachen, für die IBM rechtlich haftbar ist.   
2. Auf keinen Fall ist IBM oder einer der
Programmentwickler in folgenden Fällen haftbar, auch wenn auf die Möglichkeit
solcher Schäden hingewiesen wurde: 1) Verlust oder Beschädigung von
Daten; 2) mittelbare Schäden oder Folgeschäden oder andere
wirtschaftliche Folgeschäden; 3) entgangene Gewinne, auch wenn sie als
direkte Folge des Ereignisses entstanden sind, das zu den Schäden
geführt hat; oder 4) entgangene Geschäftsabschlüsse, Umsätze,
Schädigung des guten Namens oder Verlust erwarteter Einsparungen.   
3. Die in dieser Vereinbarung spezifizierten
Haftungsbegrenzungen und -ausschlüsse gelten nicht nur für die von IBM
durchgeführten Aktivitäten, sondern auch für die Aktivitäten von IBM
Lieferanten und Programmentwicklern. Sie legen den Höchstbetrag fest,
für den IBM und diese gemeinsam haftbar sind.   
  
Geltendes Recht und Rechtsprechung (Abschnitt 7)  
  
Geltendes Recht  
  
Der Text "dass die Gesetze des Landes zur Anwendung kommen,
in dem Sie die Programmlizenz erwerben" wird ersetzt durch:  
  
"dass die Gesetze Österreichs" in Albanien, Armenien,
Aserbaidschan, Weißrussland, Bosnien-Herzegowina, Bulgarien, Kroatien, der
früheren jugoslawischen Republik Mazedonien, Georgien, Ungarn,
Kasachstan, Kirgisien, Moldawien, Polen, Rumänien, Russland, Serbien
und Montenegro, der Slowakei, Slowenien, Tadschikistan,
Turkmenistan, der Ukraine und Usbekistan zur Anwendung kommen.  
  
Rechtsprechung  
  
Folgende Ausnahmen werden zu diesem Abschnitt hinzugefügt:  
  
1) In Österreich gilt als Gerichtsstand für alle aus dieser
Vereinbarung erwachsenden und mit dieser in Zusammenhang stehenden
Streitfälle einschließlich Streitfällen bezüglich ihres Vorhandenseins
das zuständige Gericht in Wien, Österreich (Innenstadt); 2) in
Luxemburg unterliegen sämtliche Rechtsstreitigkeiten, die sich aus
dieser Vereinbarung oder im Zusammenhang mit deren Auslegung oder
Ausführung ergeben, den Gesetzen und den Gerichten der Hauptstadt des
Landes, in dem sich Ihr Firmensitz und/oder Ihre
Handelsniederlassung befindet; 3) in Russland unterliegen sämtliche
Rechtsstreitigkeiten, die sich aus dieser Vereinbarung oder im Zusammenhang mit
deren Auslegung, Verletzung, Beendigung und Unwirksamkeit
ergeben, dem Schiedsspruchgericht (Arbitration Court) in Moskau.  
  
Dieser Abschnitt wird wie folgt ergänzt:  
  
Schiedsspruchverfahren (Arbitration)  
  
In Albanien, Armenien, Aserbaidschan, Weißrussland, Bosnien-
Herzegowina, Bulgarien, Kroatien, Georgien, Ungarn, Kasachstan,
Kirgisien, der früheren jugoslawischen Republik Mazedonien, Moldawien,
Polen, Rumänien, Russland, der Slowakei, Slowenien, Tadschikistan,
Turkmenistan, der Ukraine, Usbekistan und der Bundesrepublik Jugoslawien
unterliegen sämtliche Rechtsstreitigkeiten, die sich aus dieser
Vereinbarung oder im Zusammenhang mit deren Verletzung, Beendigung oder
Unwirksamkeit ergeben, der Schieds- und Schlichtungsordnung des
Internationalen Schiedsgerichts der Wirtschaftskammer Österreich in Wien
(Wiener Regeln) durch die drei Schiedsrichter, die in
Übereinstimmung mit diesen Richtlinien ernannt wurden. Das
Schiedsspruchverfahren findet in Wien, Österreich, statt, und die offizielle
Sprache der Verfahren ist Englisch. Die Entscheidung der
Schiedsrichter ist endgültig und bindend für beide Parteien. Gemäß
Paragraph 598 (2) des österreichischen Zivilprozesscodes verzichten
die Parteien daher ausdrücklich auf die Anwendung von Paragraph
595 (1) Ziffer 7 des Codes. IBM kann jedoch veranlassen, dass
die Verfahren vor einem zuständigen Gericht im Land der
Installation verhandelt werden.   
  
ÖSTERREICH: Allgemeines (Abschnitt 6): Ziffer 4 wird wie
folgt ergänzt:  
  
Im Rahmen dieser Bestimmung umfassen die
Kontaktinformationen auch Ihre persönlichen Informationen als juristische
Person, z. B. Umsatzdaten und andere Informationen zu
Geschäftsaktivitäten.   
  
DEUTSCHLAND: Haftungsbegrenzung (Abschnitt 5): Der
folgende Absatz wird diesem Abschnitt hinzugefügt:  
  
Die in diesem Abschnitt genannten Begrenzungen und
Ausschlüsse entfallen bei Vorsatz und grober Fahrlässigkeit von IBM.   
  
Allgemeines (Abschnitt 6): Ziffer 6 wird wie folgt ersetzt:  
  
Ansprüche, die aus dieser Vereinbarung entstehen, verjähren
binnen drei Jahren nach ihrem Entstehen.   
  
SCHWEIZ: Allgemeines (Abschnitt 6): Ziffer 4 wird wie folgt
ergänzt:   
  
Im Rahmen dieser Bestimmung umfassen die
Kontaktinformationen auch Ihre persönlichen Informationen als juristische
Person, z. B. Umsatzdaten und andere Informationen zu
Geschäftsaktivitäten.   
  
Z125-5544-03 (10/2005)  
LIZENZINFORMATION  
  
Für die Lizenzierung der nachfolgend aufgelisteten
Programme gelten zusätzlich zu den Bedingungen in Internationale
Nutzungsbedingungen für Vorab-Releases von Programmen die folgenden
Vertragsbedingungen.  
  
Programmname: alphaWorks Emerging Technology  
Programmnummer: N/A  
  
Angegebene Betriebsumgebung  
  
Die Programmspezifikationen und Informationen zur
Betriebsumgebung befinden sich in der Dokumentation zu diesem Programm,
sofern verfügbar, wie z. B. in einer Readme-Datei, oder in
anderen, von IBM veröffentlichten Informationen, wie z. B. in einer
Vertriebsfreigabe.  
  
Bewertungszeitraum  
  
Der Bewertungszeitraum beginnt an dem Datum, an dem Sie den
Bedingungen dieser Vereinbarung zustimmen, und endet nach 90 Tagen.  
  
D/N: L-JLCO-6HQ6QK  
P/N: L-JLCO-6HQ6QK   
