# Supplementary material for: Finding New Order in Biological Functions from the Network Structure of Gene Annotations
Source: PLoS Comput Biol. 2015 Nov 20;11(11):e1004565. doi: 10.1371/journal.pcbi.1004565 (PMC4654495; doi:10.1371/journal.pcbi.1004565)
Supplement: S1 Code — This file contains the input human annotation files and all the code needed to reproduce the analyses and figures presented in this manuscript. The complete collection of intermediate files (such as the predicted term-term networks, word clouds for all communities, etc), can be obtained from [34]. (TGZ) [file pcbi.1004565.s004.tgz › TermCommunities_code/MakeCloudFiles/IBM Word Cloud/license/en.html]

Software License

International License Agreement for Early Release of
Programs  
  
Part 1 - General Terms  
  
THIS INTERNATIONAL LICENSE AGREEMENT FOR EARLY RELEASE OF
PROGRAMS ("AGREEMENT") IS A LEGAL AGREEMENT BETWEEN YOU AND IBM. BY
DOWNLOADING, INSTALLING, COPYING, ACCESSING, OR USING THE PROGRAM YOU
AGREE TO THE TERMS OF THIS AGREEMENT. IF YOU ARE ACCEPTING THESE
TERMS ON BEHALF OF ANOTHER PERSON OR A COMPANY OR OTHER LEGAL
ENTITY, YOU REPRESENT AND WARRANT THAT YOU HAVE FULL AUTHORITY TO
BIND THAT PERSON, COMPANY, OR LEGAL ENTITY TO THESE TERMS.  
  
"Early Release" is a release of a Program that (1) may
still be under development (and therefore, is potentially
unreliable) or (2) may no longer be under development but has not been
made commercially available to users.  
  
"IBM" is International Business Machines Corporation or one
of its subsidiaries.  
  
"License Information" ("LI") is a document that provides
information and terms specific to a Program. The Program's LI is
available in a file in the Program's directory, by the use of a
system command, or as a booklet that accompanies the Program.  
  
"Program" is one or more of the following, including the
original and all whole or partial copies: 1) machine-readable
instructions and data, 2) human readable software components, 3) audio-
visual content (such as images, text, recordings, or pictures), 4)
related licensed materials, 5) license use documents or keys, (6)
associated documentation, and (7) any enhancements, updates or
materials that IBM may elect in its discretion to provide to You as
Support (as described below).  
  
"You" and "Your" refer either to an individual person or to
a single legal entity.  
  
This Agreement includes Part 1 - General Terms, Part 2 -
Country-unique Terms (if any), and License Information and is the
complete agreement between You and IBM regarding the use of the
Program. It replaces any prior oral or written communications
between You and IBM concerning Your use of the Program. The terms
of Part 2 and the License Information may replace or modify
those of Part 1.  
  
1. License  
  
The Program is owned by IBM or an IBM supplier, and is
copyrighted and licensed, not sold.  
  
IBM grants You a limited, nonexclusive, nontransferable
license to download, install, and use the Program during the
evaluation period solely for internal testing and evaluation purposes
and to provide feedback to IBM.  
  
You may make a backup copy of the Program to support such
use. You are not authorized to use the Program for productive
purposes or to distribute the Program or any of its parts. You may
not modify or create derivative works of the Program. The
terms of this license apply to each copy that You make. You must
reproduce all copyright notices and all other legends of ownership on
each copy, or partial copy, of the Program.  
  
You will 1) maintain a record of any copies of the Program
and 2) ensure that anyone who uses the Program (accessed either
locally or remotely) does so only for Your authorized use, and
complies with the terms of this Agreement.  
  
You may not: 1) use, copy, modify, transfer or distribute
the Program except as provided in this Agreement; 2) reverse
assemble, reverse compile, or otherwise translate the Program into
human-readable form or into another program language (except as
may be specifically permitted by law without the possibility of
contractual waiver); 3) sublicense, rent, or lease the Program; or 4)
use the Program on a service-bureau basis.  
  
This license does not entitle You to receive from IBM hard-
copy documentation, support, telephone assistance, or
enhancements or updates to the Program (collectively, "Support"),
although IBM, at its sole discretion may choose to provide such
Support. Any enhancements, updates and other materials provided by
IBM as part of Support are considered to be part of the Program
and therefore governed by this Agreement.  
  
THE PROGRAM MAY CONTAIN A DISABLING DEVICE THAT WILL
PREVENT IT FROM BEING USED AFTER THE EVALUATION PERIOD ENDS. YOU
MAY NOT TAMPER WITH THIS DISABLING DEVICE OR THE PROGRAM. YOU
SHOULD TAKE PRECAUTIONS TO AVOID ANY LOSS OF DATA THAT MIGHT
RESULT WHEN THE PROGRAM CAN NO LONGER BE USED.  
  
2. Term  
  
The evaluation period begins when You agree to the terms of
this Agreement and ends upon the earliest of 1) the end date (if
any) specified in the License Information, 2) the date on which
the Program automatically disables itself, or 3) the date on
which IBM makes the program commercially available. Your license
to the Program terminates at the end of the evaluation period,
and You will destroy the Program and all copies made of it
within 10 days of the end of the evaluation period.  
  
There is no charge for Your use of the Program for the
duration of the evaluation period.  
  
IBM may terminate Your license if You fail to comply with
the terms of this Agreement. If IBM does so, You must destroy
all copies of the Program.  
  
3. Rights In Data  
  
You assign to IBM all right, title, and interest (including
ownership of copyright) in any data, suggestions, or written
materials that 1) are related to the Program and 2) You provide to
IBM. If IBM requires it, You will sign an appropriate document
to assign such rights. To the extent not otherwise covered by
your grant under the first sentence of this Section 3, with
respect to any idea, know-how, concept, technique, invention,
discovery or improvement, whether or not patentable, related to the
Program and that you provide to IBM, You grant to IBM a non-
exclusive, irrevocable, unrestricted, worldwide and paid-up right and
license to include the foregoing in any product or service, and to
use, manufacture and market any such product or service, and to
allow others to do any of the foregoing.  
  
4. No Warranty  
  
SUBJECT TO STATUTORY WARRANTIES, IF ANY, THAT CANNOT BE
EXCLUDED, IBM MAKES NO WARRANTIES OR CONDITIONS OF ANY KIND, EITHER
EXPRESS OR IMPLIED, INCLUDING BUT NOT LIMITED TO, THE IMPLIED
WARRANTIES OR CONDITIONS OF SATISFACTORY QUALITY, MERCHANTABILITY,
FITNESS FOR A PARTICULAR PURPOSE, AND WARRANTIES OF TITLE AND NON-
INFRINGEMENT, REGARDING THE PROGRAM OR TECHNICAL SUPPORT, IF ANY.  
  
The exclusion also applies to any of IBM's Program
developers and suppliers.  
  
Manufacturers, suppliers, or publishers of non-IBM Programs
may provide their own warranties.  
  
5. Limitation of Liability  
  
Circumstances may arise where, because of a default on
IBM's part or other liability, You are entitled to recover
damages from IBM. Regardless of the basis on which You may be
entitled to claim damages from IBM, (including fundamental breach,
negligence, misrepresentation, or other contract or tort claim), IBM is
liable for no more than 1) damages for bodily injury (including
death) and damage to real property and tangible personal property
and 2) the amount of any other actual direct damages up to a
total of U.S. $25,000 (or equivalent in local currency) for all
claims in the aggregate. This limitation of liability also
applies to IBM's Program developers and suppliers. It is the
maximum for which they and IBM are collectively responsible.  
  
UNDER NO CIRCUMSTANCES IS IBM, ITS PROGRAM DEVELOPERS OR
SUPPLIERS LIABLE FOR ANY OF THE FOLLOWING, EVEN IF INFORMED OF THEIR
POSSIBILITY:  
  
1. LOSS OF, OR DAMAGE TO, DATA;  
2. SPECIAL, INCIDENTAL, INDIRECT, EXEMPLARY, OR PUNITIVE
DAMAGES, OR FOR ANY ECONOMIC CONSEQUENTIAL DAMAGES; OR  
3. LOST PROFITS, BUSINESS, REVENUE, GOODWILL, OR
ANTICIPATED SAVINGS.  
  
6. General  
  
1. Nothing in this Agreement affects any statutory rights
of consumers that cannot be waived or limited by contract.  
2. In the event that any provision of this Agreement is
held to be invalid or unenforceable, the remaining provisions of
this Agreement remain in full force and effect.  
3. You may not export the Program or take any action with
respect to the Program that violates applicable export control laws.  
4. You agree to allow International Business Machines
Corporation and its subsidiaries to store and use Your business contact
information, including names, business phone numbers, and business e-
mail addresses, anywhere they do business. Such information will
be processed and used in connection with our business
relationship; and may be provided to contractors acting on IBM's behalf,
IBM Business Partners who promote, market, and support certain
IBM products and services, and assignees of International
Business Machines Corporation and its subsidiaries, for uses
consistent with such business relationship.  
5. IBM does not warrant that any version of the Program
that is formally released or made commercially available (if
any) will be similar to, or compatible with, Early Release
versions.  
6. Neither You nor IBM will bring a legal action under this
Agreement more than two years after the cause of action arose unless
otherwise provided by local law without the possibility of
contractual waiver or limitation.  
7. Neither You nor IBM is responsible for failure to
fulfill any obligations due to causes beyond its control.  
8. This Agreement will not create any right or cause of
action for any third party, nor will IBM be responsible for any
third party claims against You except, as permitted by the
Limitation of Liability section above, for bodily injury (including
death) or damage to real or tangible personal property for which
IBM is legally liable.  
9. You may not assign this Agreement, in whole or in part,
without IBM's prior written consent. Any attempt to do so is void.  
  
7. Governing Law and Jurisdiction  
  
Governing Law  
  
Both You and IBM consent to the application of the laws of
the country in which You obtain the Program license to govern,
interpret, and enforce all of Your and IBM's rights, duties, and
obligations arising from, or relating in any manner to, the subject
matter of this Agreement, without regard to conflict of law
principles.  
  
The United Nations Convention on Contracts for the
International Sale of Goods does not apply.  
  
Jurisdiction  
  
All of our rights, duties, and obligations are subject to
the courts of the country in which You obtain the Program
license.  
  
Part 2 - Country-unique Terms  
  
AMERICAS  
ARGENTINA: Governing Law and Jurisdiction (Section 7): The
following exception is added to this section:  
  
Any litigation arising from this Agreement will be settled
exclusively by the Ordinary Commercial Court of the city of Buenos
Aires.  
  
BRAZIL: Governing Law and Jurisdiction (Section 7): The
following exception is added to this section:  
  
Any litigation arising from this Agreement will be settled
exclusively by the court of Rio de Janeiro, RJ.  
  
CANADA: Limitation of Liability (Section 5): The following
replaces item 1 in the first paragraph, second sentence of this
section:  
  
1) damages for bodily injury (including death) and physical
harm to real property and tangible personal property caused by
IBM's negligence; and  
  
General (Section 6): The following replaces the terms in
item 8:  
  
8. This Agreement will not create any right or cause of
action for any third party, nor will IBM be responsible for any
third party claims against You except as permitted by the
Limitation of Liability section above for bodily injury (including
death) or physical harm to real or tangible personal property
caused by IBM's negligence for which IBM is legally liable.  
  
Governing Law and Jurisdiction (Section 7): The phrase "the
laws of the country in which You obtain the Program license" in
the Governing Law subsection is replaced by the following:  
  
the laws in the Province of Ontario  
  
CHILE: Governing Law and Jurisdiction (Section 7): The
following exception is added to this section:  
  
Any litigation arising from this Agreement will be settled
exclusively by the Civil Courts of Justice of Santiago.  
  
ECUADOR: Governing Law and Jurisdiction (Section 7): The
following exception is added to this section:  
  
Any litigation arising from this Agreement will be settled
exclusively by the civil judges of Quito for executory or summary
proceedings (as applicable).  
  
MEXICO: Governing Law and Jurisdiction (Section 7): The
phrase "the laws of the country in which You obtain the Program
license" in the Governing Law subsection is replaced by the
following:  
  
the federal laws of the Republic of Mexico  
  
The following exception is added to this section:  
  
Any litigation arising from this Agreement will be settled
exclusively by the courts located in Mexico City, Federal District.  
  
PERU: Limitation of Liability (Section 5): The following is
added at the end of this section:  
  
In accordance with Article 1328 of the Peruvian Civil Code,
the limitations and exclusions specified in this section will
not apply to damages caused by IBM's willful misconduct
("dolo") or gross negligence ("culpa inexcusable").  
  
The following exception is added to this section:  
  
Any litigation arising from this Agreement will be settled
exclusively by the judges and tribunals of the judicial district of
Lima, Cercado.  
  
UNITED STATES OF AMERICA: General (Section 6): The
following is added to this section:  
  
U.S. Government Users Restricted Rights - Use, duplication
or disclosure restricted by the GSA ADP Schedule Contract with
the IBM Corporation.  
  
Governing Law and Jurisdiction (Section 7): The phrase "the
laws of the country in which You obtain the Program license" in
the Governing Law subsection is replaced by the following:  
  
the laws of the State of New York, United States of America  
  
Insert the following after the subsection entitled
Jurisdiction  
  
Waiver of Jury Trial  
  
Each of us expressly waives any right to a jury trial in
any proceeding directly or indirectly arising out of or
relating to this Agreement.  
  
URUGUAY: Governing Law and Jurisdiction (Section 7): The
following exception is added to this section:  
  
Any litigation arising from this Agreement will be settled
exclusively by the courts of the city of Montevideo.  
  
VENEZUELA: Governing Law and Jurisdiction (Section 7): The
phrase "the laws of the country in which You obtain the Program
license" in the Governing Law subsection is replaced by the
following:  
  
the laws of the Bolivarian Republic of Venezuela  
  
The following exception is added to this section:  
  
Any litigation arising from this Agreement will be settled
exclusively by the courts of the metropolitan area of the city of
Caracas.  
  
ASIA PACIFIC  
  
AUSTRALIA: No Warranty (Section 4): The following is added:  
  
Although IBM specifies that there are no warranties, You
may have certain rights under the Trade Practices Act 1974 or
other legislation and are only limited to the extent permitted by
the applicable legislation.  
  
Limitation of Liability (Section 5): The following is added:  
  
Where IBM is in breach of a condition or warranty implied
by the Trade Practices Act 1974, IBM's liability is limited to
the repair or replacement of the goods, or the supply of
equivalent goods. Where that condition or warranty relates to right to
sell, quiet possession or clear title, or the goods are of a kind
ordinarily acquired for personal, domestic or household use or
consumption, then none of the limitations in this paragraph apply.  
  
Governing Law and Jurisdiction (Section 7): The phrase "the
laws of the country in which You obtain the Program license" in
the Governing Law subsection is replaced by the following:  
  
the laws of the State or Territory in which the You obtain
the Program license.  
  
CAMBODIA, LAOS, and VIETNAM: Governing Law and Jurisdiction
(Section 7): The phrase "the laws of the country in which You obtain
the Program license" in the Governing Law subsection is
replaced by the following:  
  
the laws of the State of New York, United States of America  
  
The following is added to this section:  
  
Arbitration  
  
Disputes arising out of or in connection with this
Agreement shall be finally settled by arbitration which shall be held
in Singapore in accordance with the Arbitration Rules of
Singapore International Arbitration Center ("SIAC Rules") then in
effect. The arbitration award shall be final and binding for the
parties without appeal and shall be in writing and set forth the
findings of fact and the conclusions of law.  
  
The number of arbitrators shall be three, with each side to
the dispute being entitled to appoint one arbitrator. The two
arbitrators appointed by the parties shall appoint a third arbitrator
who shall act as chairman of the proceedings. Vacancies in the
post of chairman shall be filled by the president of the SIAC.
Other vacancies shall be filled by the respective nominating
party. Proceedings shall continue from the stage they were at when
the vacancy occurred.  
  
If one of the parties refuses or otherwise fails to appoint
an arbitrator within 30 days of the date the other party
appoints its, the first appointed arbitrator shall be the sole
arbitrator, provided that the arbitrator was validly and properly
appointed.  
  
All proceedings shall be conducted, including all documents
presented in such proceedings, in the English language. The English
language version of this Agreement prevails over any other language
version.  
  
HONG KONG S.A.R. and MACAU S.A.R. of China: Governing Law
and Jurisdiction (Section 7): The phrase "the laws of the
country in which You obtain the Program license" in the Governing
Law subsection is replaced by the following:  
  
the laws of Hong Kong Special Administrative Region of China  
  
INDIA: Limitation of Liability (Section 5): The following
replaces the terms of items 1 and 2 of the first paragraph, second
sentence:  
  
1) liability for bodily injury (including death) or damage
to real property and tangible personal property will be
limited to that caused by IBM's negligence; and 2) as to any other
actual damage arising in any situation involving nonperformance by
IBM pursuant to, or in any way related to the subject of this
Agreement, IBM's liability will be limited to the charge paid by You
for the individual Program that is the subject of the claim.  
  
General (Section 6): The following replaces the terms of
item 6:  
  
If no suit or other legal action is brought, within three
years after the cause of action arose, in respect of any claim
that either party may have against the other, the rights of the
concerned party in respect of such claim will be forfeited and the
other party will stand released from its obligations in respect
of such claim.  
  
Governing Law and Jurisdiction (Section 7): The following
is added to this section:  
  
Arbitration  
  
Disputes arising out of or in connection with this
Agreement shall be finally settled by arbitration which shall be held
in Bangalore, India in accordance with the laws of India then
in effect. The arbitration award shall be final and binding
for the parties without appeal and shall be in writing and set
forth the findings of fact and the conclusions of law.  
  
The number of arbitrators shall be three, with each side to
the dispute being entitled to appoint one arbitrator. The two
arbitrators appointed by the parties shall appoint a third arbitrator
who shall act as chairman of the proceedings. Vacancies in the
post of chairman shall be filled by the president of the Bar
Council of India. Other vacancies shall be filled by the respective
nominating party. Proceedings shall continue from the stage they were
at when the vacancy occurred.  
  
If one of the parties refuses or otherwise fails to appoint
an arbitrator within 30 days of the date the other party
appoints its, the first appointed arbitrator shall be the sole
arbitrator, provided that the arbitrator was validly and properly
appointed.  
  
All proceedings shall be conducted, including all documents
presented in such proceedings, in the English language. The English
language version of this Agreement prevails over any other language
version.  
  
JAPAN: General (Section 6): The following is inserted after
item 6:  
  
Any doubts concerning this Agreement will be initially
resolved between us in good faith and in accordance with the
principle of mutual trust.  
  
MALAYSIA: Limitation of Liability (Section 5): The word
"SPECIAL" in item 2 is deleted.  
  
NEW ZEALAND: No Warranty (Section 4): The following is
added:  
  
Although IBM specifies that there are no warranties, You
may have certain rights under the Consumer Guarantees Act 1993
or other legislation which cannot be excluded or limited. The
Consumer Guarantees Act 1993 will not apply in respect of any goods
which IBM provides, if You require the goods for the purposes of
a business as defined in that Act.  
  
Limitation of Liability (Section 5): The following is added:  
  
Where Programs are not obtained for the purposes of a
business as defined in the Consumer Guarantees Act 1993, the
limitations in this Section are subject to the limitations in that Act.  
  
PEOPLE'S REPUBLIC OF CHINA: Governing Law and Jurisdiction
(Section 7): The phrase "the laws of the country in which You obtain
the Program license" in the Governing Law subsection is
replaced by the following:  
  
the laws of the State of New York, United States of America
(except when local law requires otherwise)  
  
PHILIPPINES: Limitation of Liability (Section 5): The
following replaces the terms of item 2:  
  
2. special (including nominal and exemplary damages),
moral, incidental, or indirect damages or for any economic
consequential damages; or  
  
Governing Law and Jurisdiction (Section 7): The following
is added to this section:  
  
Arbitration  
  
Disputes arising out of or in connection with this
Agreement shall be finally settled by arbitration which shall be held
in Metro Manila, Philippines in accordance with the laws of
the Philippines then in effect. The arbitration award shall be
final and binding for the parties without appeal and shall be in
writing and set forth the findings of fact and the conclusions of
law.  
  
The number of arbitrators shall be three, with each side to
the dispute being entitled to appoint one arbitrator. The two
arbitrators appointed by the parties shall appoint a third arbitrator
who shall act as chairman of the proceedings. Vacancies in the
post of chairman shall be filled by the president of the
Philippine Dispute Resolution Center, Inc. Other vacancies shall be
filled by the respective nominating party. Proceedings shall
continue from the stage they were at when the vacancy occurred.  
  
If one of the parties refuses or otherwise fails to appoint
an arbitrator within 30 days of the date the other party
appoints its, the first appointed arbitrator shall be the sole
arbitrator, provided that the arbitrator was validly and properly
appointed.  
  
All proceedings shall be conducted, including all documents
presented in such proceedings, in the English language. The English
language version of this Agreement prevails over any other language
version.  
  
SINGAPORE: Limitation of Liability (Section 5): The words
"SPECIAL" and "ECONOMIC" are deleted from item 2.  
  
General (Section 6): The following replaces the terms of
item 8:  
  
3. Subject to the rights provided to IBM's suppliers and
Program developers as provided in Section 5 above (Limitation of
Liability), a person who is not a party to this Agreement shall have no
right under the Contracts (Right of Third Parties) Act to enforce
any of its terms.  
  
EUROPE, MIDDLE EAST, AFRICA (EMEA)  
Rights in Data (Section 3): In EMEA, the following replaces
the terms of this section in their entirety:  
  
You assign to IBM all right, title, and interest throughout
the world (including ownership of copyright) in any data,
suggestions, and written materials that 1) are related to Your use of
the Program and 2) You provide to IBM. Such assignment of
rights includes, but is not limited to, assignment of the rights
to prepare and have prepared derivative works of the written
materials, and to use, have used, execute, reproduce, transmit,
display, perform, transfer, distribute and license the written
materials and such derivative works in any medium or distribution
technology, and to grant others some or all of the rights granted
herein, for the duration of all such rights, title and interest. If
IBM requires it, You will sign an appropriate document to
assign such rights. With respect to any idea, know-how, concept,
technique, invention, discovery or improvement, whether or not
patentable, related to the Program and made by You or Your employees
during the term of the evaluation, You grant to IBM a non-
exclusive, irrevocable, unrestricted, worldwide and paid-up right and
license to include the foregoing in any product or service, and to
use, manufacture and market any such product or service, and to
allow others to do any of the foregoing. Neither party will
charge the other for rights in data or any work performed as a
result of this Agreement.  
  
No Warranty (Section 4): In the European Union, the
following is added at the beginning of this section:  
  
In the European Union, consumers have legal rights under
applicable national legislation governing the sale of consumer goods.
Such rights are not affected by the provisions of this Section 4.  
  
Limitation of Liability (Section 5): In Austria, Belgium,
Denmark, Finland, France, Greece, Italy, Netherlands, Norway,
Portugal, Spain, Sweden and Switzerland, the following replaces the
terms of this section in its entirety:  
  
Except as otherwise provided by mandatory law:  
  
1. IBM's liability for any damages and losses that may
arise as a consequence of the fulfillment of its obligations
under or in connection with this agreement or due to any other
cause related to this agreement is limited to the compensation of
only those damages and losses proved and actually arising as an
immediate and direct consequence of the non-fulfillment of such
obligations (if IBM is at fault) or of such cause, for a maximum amount
not to exceed ?25,000 in any event.  
The above limitation shall not apply to damages for bodily
injuries (including death) and damages to real property and tangible
personal property for which IBM is legally liable.  
2. UNDER NO CIRCUMSTANCES IS IBM, OR ANY OF ITS PROGRAM
DEVELOPERS, LIABLE FOR ANY OF THE FOLLOWING, EVEN IF INFORMED OF THEIR
POSSIBILITY: 1) LOSS OF, OR DAMAGE TO, DATA; 2) INCIDENTAL OR INDIRECT
DAMAGES, OR FOR ANY ECONOMIC CONSEQUENTIAL DAMAGES; 3) LOST PROFITS,
EVEN IF THEY ARISE AS AN IMMEDIATE CONSEQUENCE OF THE EVENT THAT
GENERATED THE DAMAGES; OR 4) LOSS OF BUSINESS, REVENUE, GOODWILL, OR
ANTICIPATED SAVINGS.  
3. The limitation and exclusion of liability herein agreed
applies not only to the activities performed by IBM but also to the
activities performed by its suppliers and Program developers, and
represents the maximum amount for which IBM as well as its suppliers
and Program developers, are collectively responsible.  
  
Governing Law and Jurisdiction (Section 7)  
  
Governing Law  
  
The phrase "the laws of the country in which You obtain the
Program license" is replaced by:  
  
1) "the laws of Austria" in Albania, Armenia, Azerbaijan,
Belarus, Bosnia-Herzegovina, Bulgaria, Croatia, Former Yugoslav
Republic of Macedonia-FYROM, Georgia, Hungary, Kazakhstan,
Kyrgyzstan, Moldavia, Poland, Romania, Russia, Serbia and Montenegro,
Slovakia, Slovenia, Tajikistan, Turkmenistan, Ukraine, and
Uzbekistan; 2) "the laws of France" in Algeria, Benin, Burkina Faso,
Cameroon, Cape Verde, Central African Republic, Chad, Comoros, Congo
Republic, Djibouti, Democratic Republic of Congo, Equatorial Guinea,
French Guiana, French Polynesia, Gabon, Gambia, Guinea, Guinea-
Bissau, Ivory Coast, Lebanon, Madagascar, Mali, Mauritania,
Mauritius, Mayotte, Morocco, New Caledonia, Niger, Reunion, Senegal,
Seychelles, Togo, Tunisia, Vanuatu, and Wallis & Futuna; 3) "the laws
of Finland" in Estonia, Latvia, and Lithuania; 4) "the laws of
England" in Angola, Bahrain, Botswana, Burundi, Egypt, Eritrea,
Ethiopia, Ghana, Jordan, Kenya, Kuwait, Liberia, Malawi, Malta,
Mozambique, Nigeria, Oman, Pakistan, Qatar, Rwanda, Sao Tome, Saudi
Arabia, Sierra Leone, Somalia, Tanzania, Uganda, United Arab
Emirates, the United Kingdom, West Bank/Gaza, Yemen, Zambia, and
Zimbabwe; and 5) "the laws of South Africa" in South Africa, Namibia,
Lesotho and Swaziland.  
  
Jurisdiction  
  
The following exceptions are added to this section:  
  
1) In Austria the choice of jurisdiction for all disputes
arising out of this Agreement and relating thereto, including its
existence, will be the competent court of law in Vienna, Austria
(Inner-City); 2) in Angola, Bahrain, Botswana, Burundi, Egypt,
Eritrea, Ethiopia, Ghana, Jordan, Kenya, Kuwait, Liberia, Malawi,
Malta, Mozambique, Nigeria, Oman, Pakistan, Qatar, Rwanda, Sao
Tome, Saudi Arabia, Sierra Leone, Somalia, Tanzania, Uganda,
United Arab Emirates, West Bank/Gaza, Yemen, Zambia, and Zimbabwe
all disputes arising out of this Agreement or related to its
execution, including summary proceedings, will be submitted to the
exclusive jurisdiction of the English courts; 3) in Belgium and
Luxembourg all disputes arising out of this Agreement or related to
its interpretation or its execution, the law, and the courts of
the capital city, of the country of Your registered office
and/or commercial site location only are competent; 4) in France,
Algeria, Benin, Burkina Faso, Cameroon, Cape Verde, Central African
Republic, Chad, Comoros, Congo Republic, Djibouti, Democratic
Republic of Congo, Equatorial Guinea, French Guiana, French
Polynesia, Gabon, Gambia, Guinea, Guinea-Bissau, Ivory Coast, Lebanon,
Madagascar, Mali, Mauritania, Mauritius, Mayotte, Morocco, New
Caledonia, Niger, Reunion, Senegal, Seychelles, Togo, Tunisia,
Vanuatu, and Wallis & Futuna all disputes arising out of this
Agreement or related to its violation or execution, including summary
proceedings, will be settled exclusively by the Commercial Court of
Paris; 5) in Russia all disputes arising out of or in relation to
the interpretation, the violation, the termination, the nullity
of the execution of this Agreement shall be settled by
Arbitration Court of Moscow; 6) in South Africa, Namibia, Lesotho and
Swaziland both of us agree to submit all disputes relating to this
Agreement to the jurisdiction of the High Court in Johannesburg; 7)
in Turkey all disputes arising out of or in connection with
this Agreement shall be resolved by the Istanbul Central
(Sultanahmet) Courts and Execution Directorates of Istanbul, the Republic
of Turkey; 8) in each of the following specified countries,
any legal claim arising out of this Agreement will be brought
before, and settled exclusively by, the competent court of a)
Athens for Greece, b) Tel Aviv-Jaffa for Israel, c) Milan for
Italy, d) Lisbon for Portugal, and e) Madrid for Spain; and 9) in
the United Kingdom, both of us agree to submit all disputes
relating to this Agreement to the jurisdiction of the English courts.  
  
The following is added to this section:  
  
Arbitration  
  
In Albania, Armenia, Azerbaijan, Belarus, Bosnia-
Herzegovina, Bulgaria, Croatia, Georgia, Hungary, Kazakhstan,
Kyrgyzstan, FYR Macedonia, Moldavia, Poland, Romania, Russia, Slovakia,
Slovenia, Tajikistan, Turkmenistan, Ukraine, Uzbekistan, and FR
Yugoslavia all disputes arising out of this Agreement or related to
its violation, termination or nullity will be finally settled
under the Rules of Arbitration and Conciliation of the
International Arbitral Center of the Federal Economic Chamber in Vienna
(Vienna Rules) by three arbitrators appointed in accordance with
these rules. The arbitration will be held in Vienna, Austria, and
the official language of the proceedings will be English. The
decision of the arbitrators will be final and binding upon both
parties. Therefore, pursuant to paragraph 598 (2) of the Austrian
Code of Civil Procedure, the parties expressly waive the
application of paragraph 595 (1) figure 7 of the Code. IBM may,
however, institute proceedings in a competent court in the country
of installation.  
  
In Estonia, Latvia and Lithuania all disputes arising in
connection with this Agreement will be finally settled in arbitration
that will be held in Helsinki, Finland in accordance with the
arbitration laws of Finland then in effect. Each party will appoint one
arbitrator. The arbitrators will then jointly appoint the chairman. If
arbitrators cannot agree on the chairman, then the Central Chamber of
Commerce in Helsinki will appoint the chairman.  
  
AUSTRIA: General (Section 6): The following is added to
item 4:  
  
For purposes of this clause, contact information will also
include information about You as a legal entity, for example
revenue data and other transactional information.  
  
GERMANY: Limitation of Liability (Section 5): The following
paragraph is added to this Section:  
  
The limitations and exclusions specified in this Section
will not apply to damages caused by IBM intentionally or by
gross negligence.  
  
General (Section 6): The following replaces the terms of
item 6:  
  
Any claims resulting from this Agreement are subject to a
statute of limitation of three years.  
  
IRELAND: No Warranty (Section 4): The following is added to
this section:  
  
Except as expressly provided in these terms and conditions,
or section 12 of the Sale of Goods Act 1893 (as amended by the
Sale of Goods and Supply of Services Act 1980 ("the 1980 Act")),
all conditions and warranties (express or implied, statutory or
otherwise) are hereby excluded including, without limitation, any
warranties implied by the Sale of Goods Act 1893 as amended by the
1980 Act (including, for the avoidance of doubt, section 39 of
the 1980 Act).  
  
Limitation of Liability (Section 5): The following replaces
the terms of this section in its entirety:  
  
For the purposes of this section, a "Default" means any
act, statement, omission, or negligence on the part of IBM in
connection with, or in relation to, the subject matter of an Agreement
in respect of which IBM is legally liable to You whether in
contract or tort. A number of Defaults which together result in, or
contribute to, substantially the same loss or damage will be treated
as one Default occurring on the date of occurrence of the last
such Default.  
  
Circumstances may arise where, because of a Default, You
are entitled to recover damages from IBM. This section sets out
the extent of IBM's liability and Your sole remedy.  
  
1. IBM will accept unlimited liability for (a) death or
personal injury caused by the negligence of IBM, and (b) subject
always to the Items for Which IBM is Not Liable below, for
physical damage to Your tangible property resulting from the
negligence of IBM.  
2. Except as provided in item 1 above, IBM's entire
liability for actual damages for any one Default will not in any
event exceed ?25,000. These limits also apply to any of IBM's
suppliers and Program developers. They state the maximum for which
IBM and such suppliers and Program developers are collectively
responsible.  
  
Items for Which IBM is Not Liable  
  
Save with respect to any liability referred to in item 1
above, under no circumstances is IBM or any of its suppliers or
Program developers liable for any of the following, even if IBM or
they were informed of the possibility of such losses:  
  
1. loss of, or damage to, data;  
2. special, indirect, or consequential loss; or  
3. loss of profits, business, revenue, goodwill, or
anticipated savings.  
  
ITALY: General (Section 6): The following is added to this
section:  
  
IBM and Customer (hereinafter, individually, "Party") shall
comply with all the obligations of the applicable provisions of
law and/or regulation on personal data protection. Each of the
Parties will indemnify and keep the other Party harmless from any
damage, claim, cost or expense incurred by the latter, directly and
or indirectly, as a consequence of an infringement of the
other Party of the mentioned provisions of law and/or regulations.  
  
SLOVAKIA: Limitation of Liability (Section 5): The
following is added to the end of the last paragraph:  
  
The limitations apply to the extent they are not prohibited
under �� 373-386 of the Slovak Commercial Code.  
  
General (Section 6): The terms of item 6 are replaced with
the following:  
  
THE PARTIES AGREE THAT, AS DEFINED BY APPLICABLE LOCAL LAW,
ANY LEGAL OR OTHER ACTION RELATED TO A BREACH OF THIS AGREEMENT
MUST BE COMMENCED NO LATER THAN FOUR YEARS FROM THE DATE ON
WHICH THE CAUSE OF ACTION AROSE.  
  
SWITZERLAND: General (Section 6): The following is added to
item 4:  
  
For purposes of this clause, contact information will also
include information about You as a legal entity, for example
revenue data and other transactional information.  
  
UNITED KINGDOM: Limitation of Liability (Section 5): The
following replaces the terms of this section in its entirety:  
  
For the purposes of this section, a "Default" means any
act, statement, omission, or negligence on the part of IBM in
connection with, or in relation to, the subject matter of an Agreement
in respect of which IBM is legally liable to You, whether in
contract or tort. A number of Defaults which together result in, or
contribute to, substantially the same loss or damage will be treated
as one Default.  
  
Circumstances may arise where, because of a Default, You
are entitled to recover damages from IBM. This section sets out
the extent of IBM's liability and Your sole remedy.  
  
1. IBM will accept unlimited liability for:  
a. death or personal injury caused by the negligence of IBM;  
b. any breach of its obligations implied by Section 12 of
the Sale of Goods Act 1979 or Section 2 of the Supply of Goods
and Services Act 1982, or any statutory modification or re-
enactment of either such Section; and  
c. subject always to the Items for Which IBM is Not Liable
below, for physical damage to Your tangible property resulting
from the negligence of IBM.  
2. IBM's entire liability for actual damages for any one
Default will not in any event, except as provided in item 1 above,
exceed �15,000. These limits also apply to IBM's suppliers and
Program developers. They state the maximum for which IBM and such
suppliers and Program developers are collectively responsible.  
  
Items for Which IBM is Not Liable  
  
Save with respect to any liability referred to in item 1
above, under no circumstances is IBM or any of its suppliers or
Program developers liable for any of the following, even if IBM or
they were informed of the possibility of such losses:  
  
1. loss of, or damage to, data;  
2. special, indirect, or consequential loss; or  
3. loss of profits, business, revenue, goodwill, or
anticipated savings.  
  
Z125-5544-03 (10/2005)  
LICENSE INFORMATION  
  
The Programs listed below are licensed under the following
terms and conditions in addition to those of the International
License Agreement for Early Release of Programs.  
  
Program Name: alphaWorks Emerging Technology  
Program Number: N/A  
  
Specified Operating Environment  
  
The Program's specifications and specified operating
environment information may be found in documentation accompanying the
Program, if available, such as a read-me file, or other information
published by IBM, such as an announcement letter.  
  
Evaluation Period  
  
The evaluation period begins on the date that You agree to
the terms of this Agreement and ends after 90 days.  
  
D/N: L-JLCO-6HQ6QK  
P/N: L-JLCO-6HQ6QK   
