# Supplementary material for: Finding New Order in Biological Functions from the Network Structure of Gene Annotations
Source: PLoS Comput Biol. 2015 Nov 20;11(11):e1004565. doi: 10.1371/journal.pcbi.1004565 (PMC4654495; doi:10.1371/journal.pcbi.1004565)
Supplement: S1 Code — This file contains the input human annotation files and all the code needed to reproduce the analyses and figures presented in this manuscript. The complete collection of intermediate files (such as the predicted term-term networks, word clouds for all communities, etc), can be obtained from [34]. (TGZ) [file pcbi.1004565.s004.tgz › TermCommunities_code/MakeCloudFiles/IBM Word Cloud/license/es.html]

Software License

Acuerdo Internacional de Licencias para la Distribución
Previa de Programas  
  
Parte 1 - Términos generales  
  
ESTE ACUERDO DE LICENCIA INTERNACIONAL PARA PROGRAMAS DE
DISTRIBUCIÓN PREVIA (en adelante el "ACUERDO") ES UN CONTRATO LEGAL
ENTRE VD. E IBM. AL BAJAR, INSTALAR, COPIAR, ACCEDER O UTILIZAR
EL PROGRAMA, VD. ACEPTA LOS TÉRMINOS DE ESTE ACUERDO. SI VD.
ACEPTA ESTOS TÉRMINOS EN NOMBRE DE OTRA PERSONA O DE UNA COMPAÑÍA
U OTRA ENTIDAD LEGAL, MANIFIESTA Y GARANTIZA QUE TIENE
AUTORIDAD TOTAL PARACOMPROMETER A ESA PERSONA, EMPRESA O ENTIDAD
LEGAL CON ESTOS TÉRMINOS.  
  
"Distribución Previa" es la release de un Programa que (1)
puede estar todavía en fase de desarrollo (y por lo tanto, es
potencialmente poco fiable) o (2) puede que ya no esté en fase de
desarrollo, pero todavía no está comercialmente disponible a los
usuarios.  
  
"IBM" es International Business Machines Corporation o una
de sus subsidiarias.  
  
"Información sobre Licencia" ( "LI") es un documento que
proporciona información y términos específicos de un Programa. La LI se
puede encontrar en un archivo en el directorio del Programa,
mediante el uso de un mandato de sistema, o como un folleto que
acompaña al Programa.  
  
"Programa" es uno o más de lo siguiente, incluyendo el
original y todas las copias completas o parciales: 1) datos e
instrucciones legibles por máquina, 2) componentes de software legibles
por el usuario, 3) contenido audiovisual (como imágenes, texto,
grabaciones o fotos), 4) materiales relacionados con la licencia, 5)
documentos o claves de licencia de uso, (6) documentación relacionada
y (7) cualquier mejora, actualización o material que IBM
pueda decidir, a su discreción, proporcionarle a Vd. como Soporte
(tal y como se describe a continuación).  
  
Se entenderá por "Vd." o "Usted" tanto una persona como una
única entidad legal.  
  
En este Acuerdo se incluye Parte 1 - Términos Generales,
Parte 2 - Términos exclusivos de cada país (si los hubiera), e
Información sobre Licencia que constituyen el acuerdo completo entre
Vd. e IBM en lo que al uso del Programa se refiere. Sustituye
cualquier comunicación verbal o escrita anterior entre Vd. e IBM
referente al uso del Programa. Los términos de la Parte 2 y la
Información sobre Licencia pueden reemplazar o modificar los términos
de la Parte 1.  
  
1. Licencia  
  
El Programa es propiedad de IBM o de un proveedor de IBM,
está sujeto a derechos de autor y a licencia de uso y no se
vende.   
  
IBM le otorga a Vd. una licencia limitada, no exclusiva e
intransferible para descargar, instalar y utilizar el Programa durante el
periodo de evaluación únicamente con el fin de efectuar pruebas
internas y de evaluación y para proporcionar comentarios a IBM.  
  
Vd. puede realizar una copia de seguridad del Programa para
dar soporte a dicho uso. Vd. no está autorizado para utilizar
el Programa con fines productivos o para distribuir el
Programa o cualquiera de sus partes. Vd. no puede modificar o crear
trabajos derivados del Programa. Los términos de esta licencia se
aplican a cada copia que Vd. realice. Vd. debe reproducir todos los
avisos de copyright y leyendas de propiedad en cada copia, total o
parcial, del Programa.  
  
Vd. 1) debe mantener un registro de todas las copias del
Programa y 2) debe asegurarse de que cualquier persona que utilice
el Programa (tanto si se accede de forma remota como si se
accede de forma local) lo haga sólo para el uso autorizado y
cumple con los términos de este Acuerdo.  
  
Vd. no puede 1) utilizar, copiar, modificar, transferir o
distribuir el Programa de un modo distinto al especificado en este
Acuerdo; 2) desensamblar, descompilar o de otro modo traducir el
Programa a un formato legible por el usuario o a otro lenguaje de
programación (excepto que específicamente lo permita la legislación, sin
que sea posible su limitación o renuncia por contrato); 3)
sublicenciar, alquilar o arrendar el Programa; o 4) utilizar el Programa
para prestar servicios a terceras partes..  
  
Esta licencia no le autoriza a Vd. a recibir de IBM
documentación impresa, soporte, asistencia telefónica o mejoras y
actualizaciones del Programa (de forma global, "Soporte"), aunque IBM, a su
entera discreción, puede decidir proporcionar dicho Soporte.
Cualquier mejora, actualización o material de otra índole que
proporcione IBM como parte del Soporte se considerará parte del
Programa y se regirá por este Acuerdo.   
  
EL PROGRAMA PUEDE CONTENER UN DISPOSITIVO INHABILITADOR QUE
IMPIDA QUE PUEDA SER UTILIZADO UNA VEZ QUE HAYA EXPIRADO EL
PERÍODO DE EVALUACIÓN. VD. NO PUEDE MODIFICAR ESTE DISPOSITIVO
INHABILITADOR NI EL PROGRAMA. VD. DEBE TOMAR PRECAUCIONES PARA EVITAR LA
PÉRDIDA DE DATOS QUE SE PODRÍA PRODUCIR CUANDO EL PROGRAMA YA NO
PUEDA UTILIZARSE.  
  
2. Vigencia  
  
El periodo de evaluación empieza cuando Vd. acepte los
términos de este Acuerdo y finaliza cuando llegue la primera de las
siguientes fechas 1) la fecha de finalización (si la hubiera) que se
especifica en la Información sobre Licencia, 2) la fecha en la que el
Programa se inhabilite automáticamente, o 3) la fecha en la que IBM
comercialice el Programa. Su licencia del Programa finaliza al final del
periodo de evaluación y Vd. destruirá el Programa y todas las
copias realizadas dentro del plazo de los diez días posteriores a
la finalización del periodo de evaluación.   
  
No hay ningún cargo por el uso del Programa por su parte
durante el periodo de evaluación.   
  
IBM puede terminar su licencia si Vd. no cumpliera con los
términos de este Acuerdo. En caso de que IBM lo hiciera, Vd. deberá
destruir todas las copias del Programa.   
  
3. Derechos sobre los datos  
  
Vd. cede a IBM todos los derechos, títulos e intereses
(incluida la propiedad de copyright) sobre cualquier dato, sugerencia
o material escrito que 1) estén relacionados con el Programa
y 2) que proporcione Vd. a IBM. Si IBM lo requiere, Vd.
tendrá que firmar un documento apropiado para asignar dichos
derechos. En la medida en que no se establezca de otro modo en la
cesión por su parte conforme a lo establecido en la primera frase
de esta Sección 3, en relación con cualquier idea,
conocimiento, concepto, técnica, invención, descubrimiento o mejora, sea
o no patentable, relacionada con el Programa y que Vd.
proporciona a IBM, Vd. otorgará a IBM una licencia y un derecho pagado,
no exclusivo, irrevocable, sin restricción y a nivel mundial
para incluir lo citado anteriormente en cualquier producto o
servicio y para utilizar, fabricar y comercializar dicho producto o
servicio y permitir a otros realizar lo anteriormente especificado.   
  
4. Sin garantía  
  
SUJETO A CUALQUIER GARANTÍA ESTATUTARIA, SI LA HUBIERA, QUE
NO PUDIERA EXCLUIRSE, IBM NO OTORGA NINGÚN TIPO DE GARANTÍA NI
CONDICIÓN, EXPLÍCITA NI IMPLÍCITA, INCLUYENDO, PERO SIN LIMITARSE A,
LAS GARANTÍAS Y CONDICIONES IMPLÍCITAS DE CALIDAD
SATISFACTORIA, COMERCIALIZACIÓN, IDONEIDAD PARA UNA FINALIDAD DETERMINADA
Y GARANTÍAS DE TITULARIDAD Y DE NO INFRACCIÓN DEL DERECHO DE
PROPIEDAD INTELECTUAL O INDUSTRIAL, EN RELACIÓN CON EL PROGRAMA O EL
SOPORTE TÉCNICO, SI LO HUBIERA.  
  
Esta exclusión también se aplica a cualquiera de los
desarrolladores y proveedores del Programa de IBM.   
  
Los fabricantes, proveedores o publicadores de Programas no
IBM pueden proporcionar sus propias garantías.  
  
5. Limitación de responsabilidad  
  
Pueden presentarse circunstancias en las que, debido a un
incumplimiento de IBM o cualquier otra responsabilidad, Vd. tuviera
derecho a reclamar daños a IBM. Independientemente de las razones
por las que Vd. está legitimado para reclamar daños a IBM
(incluyendo incumplimiento, negligencia, falsedad o cualquier otra
reclamación contractual o extracontractual), IBM será únicamente
responsable de: 1) daños físicos a las personas (incluida muerte) o
daños a las propiedades y 2) la cantidad de cualquier otro daño
directo real hasta un total de 25.000 dólares americanos (o el
equivalente en la moneda local) para todas las reclamaciones en
conjunto. Este límite de responsabilidad también se aplica a los
desarrolladores y proveedores del Programa de IBM y es la cantidad máxima
por la cual IBM y sus proveedores serán responsables
colectivamente.  
  
BAJO NINGUNA CIRCUNSTANCIA, IBM, SUS DESARROLLADORES DE
PROGRAMAS O PROVEEDORES SERÁN RESPONSABLES DE LOS SIGUIENTES CASOS,
INCLUSO EN EL CASO DE QUE HUBIERAN SIDO ADVERTIDOS DE TAL
POSIBILIDAD:   
  
1. PÉRDIDAS DE, O DAÑOS A, LOS DATOS;   
2. DAÑOS ESPECIALES, FORTUITOS, INDIRECTOS, EJEMPLARES O
PUNITIVOS O CUALQUIER DAÑO ECONÓMICO CONSECUENCIAL; O  
3. PÉRDIDAS DE BENEFICIOS, NEGOCIO, INGRESOS, PLUSVALÍAS O
ECONOMÍAS PREVISTAS  
  
6. General  
  
1. Ningún derecho estatutario de los consumidores podrá ser
cancelado o limitado por este Acuerdo.   
2. En el supuesto de que alguna cláusula de este Acuerdo no
fuera válida o bien no se pudiera hacer cumplir, el resto de
cláusulas permanecerán en vigor y con efecto pleno.  
3. Vd. no puede exportar el Programa ni realizar ningún
tipo de acción en relación con el Programa que viole las leyes
de control de exportación correspondientes.   
4. Vd. acuerda permitir a International Business Machines
Corporation y a sus subsidiarias almacenar y utilizar su información de
contactos comerciales, incluidos los nombres, los números de
teléfonos comerciales y las direcciones de correo electrónico
comerciales, dondequiera que lleven a cabo su actividad empresarial.
Dicha información se procesará y se utilizará en el contexto de
sus relaciones empresariales; puede ser ofrecida a los
contratistas que actúen en nombre de IBM, IBM Business Partners que
promueven, comercializan y dan soporte a determinados servicios y
productos de IBM y cesionarios de International Business Machines
Corporation y sus subsidiarias, para usos relacionados con dichas
relaciones empresariales.   
5. IBM no garantiza que cualquier versión del Programa que
se comercialice o que se ponga a disposición general (si la
hubiera) sea similar a, o compatible con, las versiones de
Distribución Previa.   
6. Ninguna de las partes podrá interponer acción alguna a
la que pudiera dar lugar este Acuerdo transcurridos más de dos
años desde que se produjo su causa, salvo que la Ley lo
establezca de manera distinta y no sea posible su limitación o
renuncia expresa.  
7. Ninguna de las partes será responsable del
incumplimiento de sus obligaciones cuando sea debido a causas de fuerza
mayor.  
8. Este Acuerdo no creará ningún derecho ni causa de acción
a ningún proveedor, ni IBM será responsable de las
reclamaciones contra Vd. excepto, según lo permitido por la sección de
Limitación de Responsabilidad anterior, por daño a las personas
(incluida muerte) o daño a las propiedades del cual IBM fuera
legalmente responsable.   
9. Vd. no puede ceder este Acuerdo, parcial o totalmente,
sin el previo consentimiento por escrito de IBM. Cualquier
intento de hacerlo se considerará nulo.  
  
7. Ley aplicable y Jurisdicción  
  
Ley aplicable  
  
Ambas partes se someten a la aplicación de las leyes del
país en las que Vd. haya adquirido la licencia de Programa para
interpretar, regir y hacer cumplir e implementar todos los derechos,
deberes y obligaciones de las partes que puedan surgir en virtud de
este Acuerdo, o estén relacionados de alguna manera con el
Acuerdo, a pesar de cualquier posible conflicto de ley.   
  
No se aplica la Convención de las Naciones Unidas sobre
Contratos para la Venta Internacional de Bienes.  
  
Jurisdicción  
  
Todos los derechos, deberes y obligaciones de IBM están
sujetos a los tribunales del país donde Vd. adquirió la licencia de
Programa.  
  
Parte 2 - Términos exclusivos de cada país  
  
AMÉRICA  
ARGENTINA: Ley aplicable y Jurisdicción (Sección 7): Se
añade la siguiente excepción a esta sección:   
  
Cualquier litigio que surja de este Acuerdo se someterá
exclusivamente al Tribunal de lo comercial ordinario de la ciudad de
Buenos Aires.  
  
CHILE: Ley aplicable y Jurisdicción (Sección 7): Se añade
la siguiente excepción a esta sección:   
  
Cualquier litigio que surja de este Acuerdo se someterá
exclusivamente al Tribunal Civil de Justicia de Santiago.  
  
ECUADOR: Ley aplicable y Jurisdicción (Sección 7): Se añade
la siguiente excepción a esta sección:   
  
Cualquier litigio que surja de este Acuerdo se someterá
exclusivamente al Tribunal civil de Quito para los procesos judiciales
sumarios o ejecutorios.   
  
MÉXICO: Ley aplicable y Jurisdicción (Sección 7): La frase
"las leyes del país en las que Vd. haya adquirido la licencia de
Programa" en el subapartado Ley aplicable se reemplazará por lo
siguiente:  
  
las leyes federales de la República de México  
  
La siguiente excepción se añade a esta sección:  
  
Cualquier litigio que surja de este Acuerdo se someterá
exclusivamente a los tribunales que estén ubicados en Ciudad de México,
Distrito Federal.   
  
PERÚ: Limitación de responsabilidad (Sección 5):Se añade lo
siguiente al final de esta sección:  
  
De acuerdo con el Artículo 1328 del Código Civil Peruano,
las limitaciones y exclusiones especificadas en esta sección no
se aplicarán a los daños causados por la mala conducta
deliberada de IBM ("dolo") o negligencia ("culpa inexcusable").  
  
La siguiente excepción se añade a esta sección:  
  
Cualquier litigio que surja de este Acuerdo se someterá
exclusivamente a los magistrados y tribunales del distrito federal de
Lima, Cercado.   
  
URUGUAY: Ley aplicable y Jurisdicción (Sección 7): Se añade
la siguiente excepción a esta sección:   
  
Cualquier litigio que surja de este Acuerdo se someterá
exclusivamente a los tribunales de la ciudad de Montevideo.   
  
VENEZUELA: Ley aplicable y Jurisdicción (Sección 7): La
frase "las leyes del país en las que Vd. haya adquirido la
licencia de Programa" en el subapartado Ley aplicable se reemplazará
por lo siguiente:   
  
las leyes de la República Bolivariana de Venezuela  
  
La siguiente excepción se añade a esta sección:  
  
Cualquier litigio que surja de este Acuerdo se someterá
exclusivamente a los tribunales del área metropolitana de la ciudad de
Caracas.   
  
EUROPA, ORIENTE MEDIO, ÁFRICA (EMEA)  
Derechos sobre los Datos (Sección 3): En la zona EMEA, lo
siguiente reemplaza los términos de esta sección en su totalidad:  
  
Vd. cede a IBM todos los derechos, títulos e intereses en
todo el mundo (incluyendo la propiedad del copyright) sobre
cualquier dato, sugerencia y material escrito que 1) esté relacionado
con el uso, por parte de Vd., del Programa y 2) Vd. proporcione
a IBM. Dicha cesión de derechos incluye, pero sin limitarse
a, la cesión de los derechos para preparar y haber preparado
los trabajos derivados de los materiales escritos, y para usar,
haber usado, ejecutar, reproducir, transmitir, visualizar,
realizar, transferir, distribuir y licenciar los materiales escritos
y dichos trabajos derivados en cualquier soporte o tecnología
de distribución, y para otorgar a otros algunos o todos los
derechos que aquí se ceden, mientras duren todos los derechos,
títulos e intereses mencionados. Si IBM lo requiere, Vd. tendrá que
firmar un documento apropiado para asignar dichos derechos. Con
respecto a cualquier idea, conocimiento, concepto, técnica,
invención, descubrimiento o mejora, sea o no patentable, relacionada
con el Programa y que provenga de Vd.o de sus empleados durante
el plazo de evaluación, Vd. otorgará a IBM una licencia y un
derecho pagado, no exclusivo, irrevocable, sin restricción y a
nivel mundial para incluir lo citado anteriormente en cualquier
producto o servicio y para utilizar, fabricar y comercializar dicho
producto o servicio y para permitir a otros hacer lo establecido con
anterioridad. No habrá ningún cargo a la otra parte por los derechos
sobre los datos o por trabajos realizados como resultado de este
Acuerdo.  
  
Sin Garantía (Sección 4): En la Unión Europea, se añade lo
siguiente al principio de esta sección:  
  
En la Unión Europea, los consumidores tienen derechos
legales bajo la legislación nacional aplicable que regula la venta
de bienes a los consumidores. Dichos derechos no se ven
afectados por las estipulaciones de esta Sección 4.  
  
Limitación de Responsabilidad (Sección 5): En España, lo
siguiente reemplaza los términos de esta sección en su totalidad:  
  
Excepto si se especificara de otra manera obligatoriamente
en la ley:  
  
1. La responsabilidad de IBM ante cualquier daño o pérdida
que pudiera surgir como consecuencia del cumplimiento de sus
obligaciones surgidas de este Acuerdo o en relación con él, o debido a
cualquier otra causa relacionada con el mismo, está limitada
únicamente a la compensación de aquellos daños y pérdidas probados que
realmente sean consecuencia inmediata del incumplimiento de dichas
obligaciones y que sean imputables a IBM, por una cantidad máxima que en
ningún caso excederá los 25.000 euros.   
La limitación anterior no se aplicará a daños físicos a las
personas (incluida la muerte) ni daños a las propiedades de los que
IBM sea legalmente responsable.   
2. BAJO NINGUNA CIRCUNSTANCIA, NI IBM NI LOS
DESARROLLADORES DEL PROGRAMA SERÁN RESPONSABLES EN LOS SIGUIENTES CASOS,
AUNQUE SE LES HUBIERA INFORMADO DE SU POSIBILIDAD: 1) DAÑOS O
PÉRDIDA DE DATOS; 2) DAÑOS FORTUITOS O INDIRECTOS O DAÑOS
ECONÓMICOS CONSECUENCIALES DE NINGÚN TIPO; 3) PÉRDIDA DE BENEFICIOS,
AUNQUE SURJAN COMO CONSECUENCIA INMEDIATA DEL HECHO QUE GENERÓ LOS
DAÑOS; O 4) PÉRDIDA DE NEGOCIOS, DE INGRESOS, DE PLUSVALÍA O DE
ECONOMÍAS PREVISTAS.  
3. La limitación y exclusión de la responsabilidad acordada
en este documento no sólo se aplica a las actividades
realizadas por IBM sino también a las actividades que realizan sus
proveedores y desarrolladores del Programa, y representa la cantidad
máxima por la que IBM así como sus proveedores y desarrolladores
del Programa, son responsables colectivamente.  
  
Ley aplicable y Jurisdicción (Sección 7)  
  
Jurisdicción  
  
La siguiente excepción se añade a esta sección:  
  
En España, cualquier procedimiento que pudiera surgir en
virtud de este Acuerdo o relacionado con él, se someterá a la
jurisdicción de los Tribunales y Juzgados de Madrid capital.  
  
Z125-5544-03 (10/2005)  
INFORMACIÓN SOBRE LICENCIA  
  
Los Programas que se enumeran a continuación son programas
bajo licencia sujetos a los siguientes términos y condiciones
adicionalmente a los contenidos en el Acuerdo Internacional de Licencias
para la Distribución Previa de Programas.  
  
Nombre de Programa: alphaWorks Emerging Technology  
Número de Programa: N/A  
  
Entorno Operativo Especificado  
  
La información sobre las especificaciones del Programa y
sobre el entorno operativo especificado se pueden encontrar en la
documentación que acompaña al Programa, si estuviera disponible, en forma
de archivo readme, o cualquier otra información publicada por
IBM, por ejemplo, una carta de anuncio.  
  
Periodo de Evaluación  
  
El periodo de evaluación comenzará en la fecha en que el
Cliente acepta los términos de este Acuerdo y finalizará después de
90 días.  
  
D/N: L-JLCO-6HQ6QK  
P/N: L-JLCO-6HQ6QK   
