# Supplementary material for: Finding New Order in Biological Functions from the Network Structure of Gene Annotations
Source: PLoS Comput Biol. 2015 Nov 20;11(11):e1004565. doi: 10.1371/journal.pcbi.1004565 (PMC4654495; doi:10.1371/journal.pcbi.1004565)
Supplement: S1 Code — This file contains the input human annotation files and all the code needed to reproduce the analyses and figures presented in this manuscript. The complete collection of intermediate files (such as the predicted term-term networks, word clouds for all communities, etc), can be obtained from [34]. (TGZ) [file pcbi.1004565.s004.tgz › TermCommunities_code/MakeCloudFiles/IBM Word Cloud/license/fr.html]

Software License

Conditions Internationales d'Utilisation des Pré-versions
de Logiciels IBM  
  
Chapitre 1 - Dispositions Générales  
  
LES PRÉSENTES CONDITIONS INTERNATIONALES D'UTILISATION DES
PRÉ-VERSIONS DE LOGICIELS (CI-APRÈS, LE "CONTRAT") CONSTITUENT
UN CONTRAT JURIDIQUE ENTRE VOUS ET IBM. LE TÉLÉCHARGEMENT,
L'INSTALLATION, LA COPIE, L'ACCÈS OU L'UTILISATION DU LOGICIEL SERA
CONSIDÉRÉ COMME UNE ACCEPTATION DE VOTRE PART DES DISPOSITIONS DU
PRÉSENT CONTRAT. SI VOUS ACCEPTEZ CES DISPOSITIONS POUR LE COMPTE
D'UNE AUTRE PERSONNE, D'UNE SOCIÉTÉ OU D'UNE AUTRE PERSONNE
MORALE, VOUS CERTIFIEZ AVOIR QUALITÉ POUR ENGAGER CETTE PERSONNE,
SOCIÉTÉ OU PERSONNE MORALE À RESPECTER CES DISPOSITIONS.  
  
Le terme "Pré-version" désigne la version d'un Logiciel qui
(1) peut encore être en cours de développement (et qui risque
par conséquent d'être peu fiable) ou (2) n'est peut-être plus
en cours de développement mais qui n'a pas encore été rendue
disponible à la commercialisation.  
  
Le terme "IBM" désigne la compagnie International Business
Machines Corporation ou l'une de ses filiales.  
  
Le terme "Informations sur la Licence" ("LI") désigne un
document qui fournit des informations et des dispositions
spécifiques à un Logiciel donné. Les Informations sur la Licence
peuvent se trouver dans un fichier fourni avec le Logiciel et
accessible par une commande système ou dans une brochure fournie avec
le Logiciel.  
  
Le terme "Logiciel" désigne un ou plusieurs des éléments
suivants, y compris l'original et toutes les copies partielles ou
intégrales de chaque élément : 1) les instructions et les données
lisibles par machine, 2) les composants logiciels lisibles par une
personne, 3) les informations audio et/ou visuelles (par exemple,
images, textes, enregistrements ou dessins), 4) les éléments sous
licence associés, 5) les clés ou les documents relatifs à
l'utilisation de la licence, 6) la documentation associée, et 7) les
améliorations, mises à jour ou éléments qu'IBM peut décider, à sa seule
discrétion, de Vous fournir en tant que Support (tel que décrit ci-
dessous).  
  
Les termes "Vous" et "Votre" font référence à un individu
ou à une personne morale.  
  
Ce Contrat est constitué du présent document, qui comprend
un premier chapitre intitulé Chapitre 1 - Dispositions
Générales et un second chapitre intitulé Chapitre 2 - Dispositions
Nationales Particulières (le cas échéant), et du document Informations
sur la Licence. Le Contrat exprime l'intégralité de l'accord
intervenu entre Vous et IBM en ce qui concerne l'utilisation du
Logiciel. Il prévaut sur tout autre accord ou communication
antérieur, oral ou écrit, intervenu entre les parties, concernant
Votre utilisation du Logiciel. Les dispositions du second
chapitre et du document Informations sur la Licence peuvent
remplacer ou modifier celles du premier chapitre.  
  
1. Licence  
  
Le Logiciel est la propriété d'IBM ou d'un fournisseur
d'IBM et est protégé par les droits d'auteur. Le Logiciel est
concédé sous licence et non vendu.  
  
IBM Vous concède une licence d'utilisation du Logiciel
limitée, non exclusive et non transférable Vous autorisant à
télécharger, installer et utiliser le Logiciel pendant la période
d'évaluation, uniquement à des fins d'évaluation et de test en interne,
et à envoyer des commentaires en retour à IBM.  
  
Vous êtes autorisé à faire une copie de sauvegarde du
Logiciel, pour permettre une telle utilisation. Vous n'êtes pas
autorisé à utiliser le Logiciel à des fins de production, ni à
distribuer le Logiciel ou tout composant de celui-ci.  
  
Vous n'êtes pas autorisé à modifier le Logiciel, ni à en
créer des oeuvres dérivées. Les dispositions de la présente
licence s'appliquent à toutes les copies que Vous effectuez. Vous
devez reproduire toutes les mentions relatives aux droits
d'auteur et toute autre mention de propriété sur chaque copie,
totale ou partielle, du Logiciel.  
  
Vous Vous engagez 1) à tenir à jour un enregistrement de
toutes les copies du Logiciel et 2) à Vous assurer que toute
personne utilisant le Logiciel (que ce soit au travers de son réseau
privé ou d'un réseau public) ne le fasse que pour Votre usage
dans la limite des droits concédés et conformément aux
dispositions du présent Contrat.  
  
Vous n'êtes pas autorisé à : 1) utiliser, copier, modifier,
céder ou distribuer le Logiciel, sauf indication contraire dans
le présent Contrat ; 2) désassembler, décompiler ou traduire
de quelque façon que ce soit le Logiciel dans une forme
lisible par une personne ou dans un autre langage informatique, à
moins d'y être autorisé par une disposition légale d'ordre public
; 3) concéder des sous-licences ou donner le Logiciel en
location sous quelque forme que ce soit ; ni 4) utiliser le Logiciel
en mode service bureau.   
  
La présente licence ne Vous permet pas de bénéficier de la
documentation papier, du support, de l'assistance téléphonique IBM, ni
des améliorations ou des mises à jour du Logiciel
(collectivement, le "Support"), même si IBM, à sa seule discrétion, peut
décider de fournir ce Support. Les améliorations, mises à jour et
autres éléments fournis par IBM en tant que Support sont
considérés comme faisant partie intégrante du Logiciel et sont donc
soumis aux dispositions du présent Contrat.  
  
LE LOGICIEL PEUT CONTENIR UN DISPOSITIF DE DÉSACTIVATION
POUR EN INTERDIRE L'UTILISATION À L'EXPIRATION DE LA PÉRIODE
D'ÉVALUATION. VOUS VOUS ENGAGEZ À NE PAS ALTÉRER CE DISPOSITIF NI LE
LOGICIEL. VOUS DEVEZ PRENDRE LES PRÉCAUTIONS NÉCESSAIRES POUR ÉVITER
DE PERDRE DES DONNÉES LORSQUE VOUS NE POURREZ PLUS UTILISER LE
LOGICIEL.  
  
2. Durée  
  
La période d'évaluation prend effet lors de Votre
acceptation des dispositions du présent Contrat et prend fin à la
première des dates suivantes : 1) à la date indiquée dans le
document Informations sur la Licence, 2) à la date de la
désactivation automatique du Logiciel ou 3) à la date à laquelle IBM
commercialise le Logiciel. Votre licence d'utilisation du Logiciel est
résiliée à la fin de la période d'évaluation et Vous devez détruire
le Logiciel, ainsi que toutes les copies que Vous avez faites,
dans les dix jours suivant l'expiration de la période
d'évaluation.   
  
L'utilisation du Logiciel est gratuite pendant la période
d'évaluation.   
  
IBM peut résilier la licence qui Vous a été concédée si
Vous ne respectez pas les dispositions du présent Contrat. Dans
ce cas, Vous devrez détruire tous les exemplaires du Logiciel.  
  
3. Propriété et droits d'auteur  
  
Vous cédez à IBM par le présent Contrat tous vos droits de
propriété sur les données, suggestions et éléments écrits 1) relatifs
au Logiciel et 2) que Vous communiquez à IBM, ainsi que
l'intégralité des droits d'auteur y afférents pour toute la durée de leur
existence. Si IBM le juge nécessaire, Vous Vous engagez à signer tout
document approprié pour parfaire ce transfert de droits. Dans les
limites non couvertes par Votre cession de droits au titre de la
première phrase du présent Article 3, en ce qui concerne tout savoir-
faire, idée, concept, technique, invention, découverte ou
amélioration concernant le Logiciel, faisant ou non l'objet d'un brevet,
que Vous fournissez à IBM, Vous accordez à IBM, sans frais
supplémentaire, une licence non exclusive, irrévocable, non limitée et
mondiale l'autorisant à inclure les éléments sus-mentionnés dans
tout produit ou service, à utiliser, fabriquer et commercialiser
ce produit ou service, et à autoriser des tiers à en faire
autant.   
  
4. Exclusion de garantie  
  
SOUS RÉSERVE DE TOUTE GARANTIE LÉGALE QUI NE PEUT ÊTRE
EXCLUE, IBM NE FOURNIT AUCUNE GARANTIE EXPLICITE OU IMPLICITE, Y
COMPRIS, ET DE FAÇON NON LIMITATIVE, TOUTE GARANTIE OU CONDITION
IMPLICITE D'APTITUDE À L'EXÉCUTION D'UN TRAVAIL DONNÉ, ET TOUTE
GARANTIE EN NON-CONTREFAÇON CONCERNANT LE LOGICIEL OU LE SUPPORT
TECHNIQUE, LE CAS ÉCHÉANT.  
  
Cette exclusion s'applique également aux développeurs et
fournisseurs d'un Logiciel IBM.  
  
Les fabricants, fournisseurs ou éditeurs de Logiciels non
IBM peuvent fournir leurs propres garanties.  
  
5. Limitation de responsabilité  
  
Des circonstances peuvent survenir Vous donnant le droit,
en raison d'un manquement de la part d'IBM ou d'une autre
forme de responsabilité, de réclamer des dommages-intérêts à IBM.
Quel que soit le fondement de l'action que Vous pourriez engager
contre IBM (notamment pour violation d'une condition essentielle
de ce Contrat, négligence, tromperie ou autre faute
contractuelle), la responsabilité d'IBM sera limitée 1) au montant des
dommages corporels (y compris le décès) et des dommages aux biens
matériels, mobiliers ou immobiliers, et 2) au montant de tout autre
dommage direct et réel plafonné à un montant total de 25 000,00 U.
S. dollars pour l'ensemble des réclamations. Cette limitation
de responsabilité s'applique également aux développeurs et
fournisseurs d'un Logiciel d'IBM. C'est le maximum pour lequel IBM, ses
développeurs et ses fournisseurs sont collectivement responsables.  
  
IBM, SES DÉVELOPPEURS OU FOURNISSEURS NE PEUVENT EN AUCUN
CAS ÊTRE TENUS RESPONSABLES DES DOMMAGES SUIVANTS, ET CE, MÊME
S'ILS ONT ÉTÉ INFORMÉS DE LEUR POSSIBLE SURVENANCE :   
  
1. PERTE OU DÉTÉRIORATION DE VOS DONNÉES ;  
2. PRÉJUDICES MORAUX, ACCESSOIRES OU INDIRECTS ; OU   
3. PERTE DE BÉNÉFICE, D'ACTIVITÉ COMMERCIALE, DE REVENU, DE
CLIENTÈLE, OU D'ÉCONOMIES ESCOMPTÉES.  
  
6. Autres dispositions  
  
1. Le présent Contrat ne porte atteinte à aucune des
dispositions légales d'ordre public relatives aux droits des
consommateurs.  
2. Si l'une quelconque des dispositions du présent Contrat
est considérée comme nulle ou inapplicable, elle n'entraînera
pas la nullité des autres dispositions.  
3. Vous n'êtes pas autorisé à exporter le Logiciel, ni à
entreprendre aucune action concernant le Logiciel qui serait en
violation des lois en vigueur sur le contrôle des exportations.  
4. Vous acceptez par le présent Contrat qu'International
Business Machines Corporation  
et ses filiales enregistrent et utilisent les coordonnées
professionnelles de Vos contacts, comprenant leurs noms, numéros de
téléphone professionnels et adresses électroniques professionnelles,
quel que soit le lieu où ils exercent leurs activités. Ces
informations seront traitées et utilisées dans le cadre de nos relations
commerciales et pourront être communiquées aux sous-traitants agissant
pour le compte d'IBM, aux Partenaires Commerciaux IBM qui
commercialisent certains produits et services IBM, et en assurent la
promotion et le support, et aux cessionnaires d'International
Business Machines Corporation, ainsi qu'à ses filiales, pour être
utilisées dans le cadre de leurs activités commerciales.   
5. En cas d'annonce ou de commercialisation du Logiciel,
IBM ne garantit pas que toute version de ce Logiciel sera
similaire à la présente Pré-version ou compatible avec celle-ci.  
6. Ni Vous-même, ni IBM ne pourra intenter une action
contre l'autre plus de deux ans après l'apparition de son fait
générateur, sauf disposition légale contraire d'ordre public.  
7. Ni Vous-même, ni IBM ne sera responsable d'un manquement
à ses obligations si un tel manquement résulte d'un cas de
force majeure.  
8. Ce Contrat ne crée aucun droit ni intérêt pour agir à
tout tiers et IBM n'est responsable d'aucune réclamation d'un
tiers contre Vous sauf, tel qu'il est prévu dans l'article
"Limitation de responsabilité" ci-dessus, au titre de dommages
corporels (incluant le décès) et dommages aux biens matériels,
mobiliers et immobiliers, pour lesquels IBM est légalement
responsable.  
9. Vous n'êtes pas autorisé à céder tout ou partie du
présent Contrat sans l'accord écrit préalable d'IBM. Toute
tentative en ce sens est nulle et non avenue.  
  
7. Droit applicable et juridiction compétente  
  
Droit applicable  
  
Les parties (Vous et IBM) sont d'accord pour que soient
appliquées les lois du pays où Vous avez acquis la licence de Logiciel
pour régir, interpréter et faire respecter leurs droits, devoirs
et obligations découlant, directement ou indirectement, de
l'objet du présent Contrat, sans donner effet aux principes de
conflit de lois.   
  
La Convention des Nations Unies sur les contrats régissant
le Commerce International de Biens ne s'applique pas.  
  
Juridiction compétente  
  
Tous les droits, devoirs et obligations des parties sont
soumis aux tribunaux du pays dans lequel Vous avez acquis la
licence du Logiciel.  
  
Chapitre 2 - Dispositions nationales particulières  
  
AMÉRIQUE  
CANADA : Limitation de responsabilité (Article 5) : La
ligne suivante remplace l'alinéa 1 du premier paragraphe, dans la
deuxième phrase de cet article :  
  
1) au montant des dommages corporels (y compris le décès)
et des dégâts matériels aux biens matériels, mobiliers et
immobiliers causés par une négligence d'IBM, et  
  
Autres dispositions (Article 6) : L'alinéa 8 est remplacé
comme suit :  
  
8. Le présent Contrat ne crée aucun droit ni intérêt pour
agir à tout tiers et IBM n'est responsable d'aucune réclamation
d'un tiers contre Vous sauf, tel qu'il est prévu dans l'article
"Limitation de responsabilité" ci-dessus, au titre de dommages
corporels (incluant le décès) et dégâts matériels aux biens
matériels, mobiliers et immobiliers, causés par une négligence pour
laquelle IBM est légalement responsable.  
  
Droit applicable et juridiction compétente (Article 7) : La
mention "les lois du pays où Vous avez acquis la licence du
Logiciel" dans la sous-section Droit applicable est remplacée par la
mention suivante :  
  
les lois en vigueur dans la Province de l'Ontario  
  
EUROPE, MOYEN-ORIENT, AFRIQUE (EMEA)  
Propriété et droits d'auteur (Article 3) : Dans les pays
EMEA, le paragraphe suivant remplace les dispositions de cet
article dans son intégralité :  
  
Vous cédez à IBM par le présent Contrat tous Vos droits de
propriété sur les données, suggestions et éléments écrits 1) relatifs
au Logiciel et 2) que Vous communiquez à IBM, ainsi que
l'intégralité des droits d'auteur y afférents dans tout pays et pour
toute la durée de leur existence. Cette cession de droits inclus
notamment mais sans que cette liste soit limitative : les droits de
faire et de faire faire des oeuvres dérivées des éléments écrits,
d'utiliser et de faire utiliser, d'exécuter, reproduire, transmettre,
afficher, représenter, transférer, et distribuer ces éléments et
leurs oeuvres dérivées sur tout support et au moyen de toute
technique de distribution, le droit d'accorder des licences sur ces
éléments et leurs oeuvres dérivées, ainsi que le droit d'accorder à
tout tiers l'ensemble ou partie de ses droits. Si IBM le juge
nécessaire, vous vous engagez à signer tout document approprié pour
parfaire ce transfert de droits. En ce qui concerne tout savoir-
faire, idée, concept, technique, invention, découverte ou
amélioration concernant le Logiciel, faisant ou non l'objet d'un brevet,
mis au point par Vous ou Vos employés pendant la période
d'évaluation, Vous accordez à IBM, sans frais supplémentaire, une licence
non exclusive, irrévocable, non limitée et mondiale
l'autorisant à inclure les éléments sus-mentionnés dans tout produit ou
service, à utiliser, fabriquer et commercialiser ce produit ou
service, et à autoriser des tiers à en faire autant. Aucune des deux
parties ne demandera de compensation financière à l'autre partie
pour les cessions de droits ou les travaux effectués dans le
cadre de ce Contrat.  
  
Exclusion de garantie (Article 4) : Pour l'Union
européenne, le paragraphe suivant est ajouté au début de cet article :  
  
Dans l'Union européenne, les consommateurs disposent de
droits selon la loi nationale en vigueur régissant la vente de
biens de consommation. Ces droits ne sont pas affectés par les
dispositions de l'Article 4 du présent Contrat.  
  
Limitation de responsabilité (Article 5) : En Belgique, en
France et en Suisse, les paragraphes suivants remplacent les
dispositions de cet article dans son intégralité :  
  
Sauf disposition légale impérative contraire :  
  
1. La responsabilité d'IBM, concernant tout dommage et
perte pouvant survenir dans le cadre de l'exercice de ses
obligations liées directement ou indirectement au présent Contrat ou
résultant d'autres causes liées à ce Contrat, est limitée au
dédommagement des seuls dommages et pertes prouvés et résultant
immédiatement et directement du manquement à ces obligations (en cas de
faute d'IBM) ou d'une telle cause, pour un montant maximum ne
pouvant au aucun cas excéder 25 000 euros.  
La limitation sus-mentionnée ne s'applique pas aux dommages
corporels (incluant le décès) et dommages aux biens matériels,
mobiliers et immobiliers, pour lesquels IBM est légalement
responsable.  
2. IBM ET SES DÉVELOPPEURS NE PEUVENT EN AUCUN CAS ÊTRE
TENUS RESPONSABLES DES DOMMAGES SUIVANTS, ET CE, MÊME S'ILS ONT
ÉTÉ INFORMÉS DE LEUR POSSIBLE SURVENANCE : 1) PERTE OU
DÉTÉRIORATION DE DONNÉES ; 2) DOMMAGES INDIRECTS OU SPÉCIAUX ; 3) PERTE
DE BÉNÉFICES, MÊME SI CELLE-CI EST LA CONSÉQUENCE IMMÉDIATE DE
L'ÉVÉNEMENT À L'ORIGINE DES DOMMAGES ; ET 4) PRÉJUDICE COMMERCIAL,
PERTE DE CHIFFRE D'AFFAIRES, PERTE DE CLIENTÈLE, OU PERTE
D'ÉCONOMIES ESCOMPTÉES.  
3. Les limitations et exclusions convenues ci-dessus
s'appliquent non seulement aux activités d'IBM mais également à celles
de ses fournisseurs et ses développeurs, et définissent le
montant maximum pour lequel IBM, ses fournisseurs et ses
développeurs sont collectivement responsables.  
  
Droit applicable et juridiction compétente (Article 7)  
  
Droit applicable  
  
La mention "soient appliquées les lois du pays où Vous avez
acquis la licence du Logiciel" est remplacée par :  
  
"soit appliqué le droit français" en Algérie, au Bénin, au
Burkina Faso, au Cameroun, au Cap-Vert, en République
centrafricaine, au Tchad, aux Comores, au Congo, à Djibouti, en République
démocratique du Congo, en Guinée Equatoriale, en Guyane française, en
Polynésie française, au Gabon, en Gambie, en Guinée, en Guinée-
Bissau, en Côte d'Ivoire, au Liban, à Madagascar, au Mali, en
Mauritanie, dans l'Ile Maurice, dans l'Ile Mayotte, au Maroc, en
Nouvelle Calédonie, au Niger, dans l'Ile de la Réunion, au Sénégal,
aux Seychelles, au Togo, en Tunisie, dans les îles Vanuatu, et
Wallis & Futuna.  
  
Juridiction compétente  
  
Les exceptions suivantes sont ajoutées à cet article :  
  
1) En Belgique et au Luxembourg, pour tout conflit
résultant de ou relatif au présent Contrat, à son interprétation ou à
son exécution, seules sont compétentes la loi et les cours de
justice de la capitale du pays où se situent Votre siège social
et/ou Votre bureau commercial ; 2) En France, en Algérie, au
Bénin, au Burkina Faso, au Cameroun, au Cap-Vert, en République
centrafricaine, au Tchad, aux Comores, au Congo, à Djibouti, en République
démocratique du Congo, en Guinée Equatoriale, en Guyane française, en
Polynésie française, au Gabon, en Gambie, en Guinée équatoriale, en
Guinée-Bissau, en Côte d'Ivoire, au Liban, à Madagascar, au Mali,
en Mauritanie, dans l'Ile Maurice, dans l'Ile Mayotte, au
Maroc, en Nouvelle Calédonie, au Niger, dans l'Ile de la Réunion,
au Sénégal, aux Seychelles, au Togo, en Tunisie, dans les îles
Vanuatu, et Wallis & Futuna , tout conflit résultant de ou relatif
au présent Contrat, à sa violation ou à son exécution sera
exclusivement réglé par le Tribunal de Commerce de Paris, nonobstant
pluralité de défendeurs ou appel en garantie ; cette attribution de
compétence s'applique également pour les procédures d'urgence et les
procédures conservatoires.  
  
SUISSE : Autres dispositions (Article 6) : La phrase
suivante est ajoutée à l'alinéa 4 :  
  
Dans le cadre de cette clause, les données relatives aux
contacts comporteront également des informations Vous concernant en
tant que personne morale, par exemple données sur Votre chiffre
d'affaires ou autres données commerciales.  
  
Z125-5544-03 (10/2005)  
INFORMATIONS SUR LA LICENCE  
  
Les dispositions suivantes s'ajoutent à celles contenues
dans le document Conditions Internationales d'Utilisation des
Pré-versions de Logiciels IBM, pour l'utilisation des Logiciels
indiqués ci-dessous.  
  
Nom du Logiciel : alphaWorks Emerging Technology  
Référence du Logiciel : N/A  
  
Environnement Opérationnel Spécifié  
  
Les spécifications du Logiciel et les informations
relatives à l'Environnement Opérationnel Spécifié se trouvent dans la
documentation fournie avec le Logiciel, le cas échéant, par exemple, dans
un avertissement (un fichier "README") ou tout autre document
d'information, tel qu'une lettre d'annonce.  
  
Période d'évaluation  
  
La période d'évaluation prend effet le jour où Vous
acceptez les termes de ce Contrat et prend fin après 90 jours.  
  
D/N: L-JLCO-6HQ6QK  
P/N: L-JLCO-6HQ6QK   
