# Supplementary material for: Finding New Order in Biological Functions from the Network Structure of Gene Annotations
Source: PLoS Comput Biol. 2015 Nov 20;11(11):e1004565. doi: 10.1371/journal.pcbi.1004565 (PMC4654495; doi:10.1371/journal.pcbi.1004565)
Supplement: S1 Code — This file contains the input human annotation files and all the code needed to reproduce the analyses and figures presented in this manuscript. The complete collection of intermediate files (such as the predicted term-term networks, word clouds for all communities, etc), can be obtained from [34]. (TGZ) [file pcbi.1004565.s004.tgz › TermCommunities_code/MakeCloudFiles/IBM Word Cloud/license/it.html]

Software License

Accordo di Licenza Internazionale per Release Preliminare
di Programmi (ILAR)  
  
Parte 1 - Condizioni Generali  
  
IL PRESENTE ILAR (ACCORDO DI LICENZA INTERNAZIONALE PER IL
RELEASE PRELIMINARE DI PROGRAMMI) ("ACCORDO") E' UN ACCORDO LEGALE
TRA L'UTENTE ED IBM. SCARICANDO, INSTALLANDO, COPIANDO,
ACCEDENDO O UTILIZZANDO IL PROGRAMMA SI ACCETTANO LE DISPOSIZIONI DEL
PRESENTE ACCORDO. SE SI ACCETTANO TALI CLAUSOLE PER CONTO DI
UN'ALTRA SOCIETÀ O PERSONA FISICA O GIURIDICA, SI DICHIARA E SI
GARANTISCE DI AVERE PIENA AUTORITÀ DI VINCOLARE TALE SOCIETÀ O PERSONA
FISICA O GIURIDICA ALLE PRESENTI CLAUSOLE.   
  
Per "Release preliminare" si intende un release di un
Programma che (1) può essere ancora in fase di sviluppo (e pertanto,
è potenzialmente inaffidabile) o (2) può non essere più in
fase di sviluppo, ma non è stato reso commercialmente
disponibile agli utenti.  
  
Per "IBM" si intende International Business Machines
Corporation o una delle sue consociate.   
  
Per "Informazioni relative alla Licenza" ("LI") si intende
un documento che fornisce informazioni e condizioni specifiche
per un Programma. Le LI del Programma sono disponibili in un
file nella directory del Programma, utilizzando un comando di
sistema, o nel libretto che accompagna il Programma.   
  
Per "Programma" si intende sia il programma originale che
tutte le copie integrali o parziali: 1) istruzioni in formato
leggibile dalla macchina e dati, 2) le componenti software leggibili
dall'uomo, 3) contenuto audio-visivo (quale immagini, testo,
registrazioni o figure), 4) materiali su licenza associati, 5) documenti
o chiavi di utilizzo della licenza, 6) la documentazione
associata e (7) qualsiasi miglioramento, aggiornamento o materiale
scelto da IBM, a sua discrezione, da fornire come Supporto (come
descritto di seguito).   
  
Per "Utente" e "dell'Utente" si intende sia una singola
persona fisica che una società.  
  
Il presente Accordo è suddiviso in Parte 1 - Condizioni
Generali, Parte 2 - Disposizioni specifiche per i singoli paesi (se
esistenti), e Informazioni relative alla Licenza e costituisce
l'accordo integrale tra l'utente ed IBM relativamente all'uso del
Programma. Sostituisce qualsiasi altra precedente comunicazione
verbale o scritta intercorsa tra l'utente ed IBM relativa all'uso
del Programma. Le disposizioni incluse nella Parte 2 e nelle
Informazioni relative alla Licenza possono sostituire o modificare
quelle contenute nella Parte 1.  
  
1. Licenza  
  
Il Programma è di proprietà di IBM o di un fornitore IBM,
ed è tutelato dalle leggi sul copyright, viene concesso in
licenza, ma non viene venduto.  
  
IBM concede all'utente una licenza limitata, non esclusiva
e non trasferibile per scaricare, installare ed utilizzare il
Programma durante il periodo di valutazione solo per scopi di test e
valutazione interni e per fornire feedback ad IBM.  
  
E' possibile effettuare una copia di backup del Programma
per supportare tale uso. Non si è autorizzati ad utilizzare il
Programma per uso produttivo o a distribuire il Programma o qualsiasi
sua parte. Non è possibile modificare o creare opere derivate
del Programma. Le disposizioni della presente licenza si
applicano a ciascuna copia effettuata. Il licenziatario dovrà
riprodurre su ciascuna copia, anche parziale, del Programma le
indicazioni relative al copyright e ogni altra indicazione relativa a
diritti di proprietà.   
  
L'utente dovrà 1) conservare una registrazione di tutte le
copie del Programma e 2) assicurarsi che chiunque utilizzi il
Programma (con accesso locale o remoto) rispetti l'uso autorizzato ed
accetti le disposizioni del presente Accordo.   
  
Non è possibile: 1) utilizzare, copiare, modificare,
trasferire o distribuire il Programma, ad eccezione di quanto previsto
nel presente accordo; 2) assemblare a ritroso, compilare a
ritroso, o in altro modo tradurre il Programma in formato leggibile
dall'uomo o in un altro linguaggio di programmazione (salvo quanto
espressamente previsto dalle norme di legge escludendo la possibilità di
rinuncia contrattuale); 3) sublicenziare, concedere in noleggio o
locazione il Programma; o 4) utilizzare il Programma nell'ambito di
una agenzia di servizi.  
  
La presente licenza non concede il diritto a ricevere da
IBM documentazione cartacea, supporto, assistenza telefonica o
miglioramenti o aggiornamenti al Programma (nell'insieme, "Supporto"),
sebbene IBM, a propria discrezione, possa decidere di fornire tale
Supporto. Qualsiasi miglioramento, aggiornamento o altro materiale
fornito da IBM come parte del Supporto verrà considerato come parte
del Programma e, pertanto, sarà regolato dal presente Accordo.   
  
IL PROGRAMMA PUÒ CONTENERE UN DISPOSITIVO DI DISATTIVAZIONE
ATTO AD IMPEDIRNE L'UTILIZZO DOPO LA SCADENZA DEL PERIODO DI
VALUTAZIONE. NON SI DOVRÀ ALTERARE TALE DISPOSITIVO DI DISATTIVAZIONE O
IL PROGRAMMA. SARÀ NECESSARIO PRENDERE DELLE PRECAUZIONI PER
EVITARE QUALSIASI PERDITA DI DATI CHE POTREBBE VERIFICARSI QUANDO
IL PROGRAMMA NON POTRÀ PIÙ ESSERE UTILIZZATO.  
  
2. Durata  
  
Il periodo di valutazione decorre dalla data in cui
l'utente accetta le disposizioni del presente Accordo e termina al
verificarsi del primo evento tra quelli di seguito indicati: 1) alla
data di scadenza (se applicabile) specificata nelle Informazioni
relative alla Licenza, 2) alla data in cui il Programma si
disabilita automaticamente, o 3) alla data in cui IBM rende il
Programma commercialmente disponibile, a seconda di quale si
verifichi per primo. La licenza al Programma terminerà alla fine del
periodo di valutazione e, entro 10 giorni dalla revoca del periodo
di valutazione, si dovrà distruggere il Programma e tutte le
copie effettuate.   
  
Non è previsto alcun canone per l'utilizzo del Programma
durante il periodo di valutazione.  
  
IBM potrà revocare la licenza se non si rispettano le
disposizioni del presente Accordo. In tal caso, l'utente dovrà
distruggere tutte le copie del Programma.  
  
3. Diritti relativi ai Dati  
  
Si assegna ad IBM ogni diritto, titolarità ed interesse
(inclusa la proprietà del copyright) relativo a dati, suggerimenti e
materiale cartaceo che 1) è relativo al Programma e 2) è fornito ad
IBM. Se IBM lo richiede, si firmerà un documento appropriato per
assegnare tali diritti. Laddove non diversamente concesso
dall'utente in base alla prima frase della presente Sezione 3, in
relazione a qualsiasi idea, know-how, concetto, tecnica, invenzione,
scoperta o miglioramento, brevettabili o meno, relativo al Programma
e fornito ad IBM, si concede ad IBM un diritto ed una licenza
non esclusivi, irrevocabili, illimitati, validi in tutto il
mondo e senza alcun obbligo di pagamento per includere quanto
precedentemente detto in qualsiasi prodotto o servizio e di utilizzare,
produrre e vendere uno qualsiasi di tali prodotti o servizi e di
consentire a terzi di procedere secondo quanto affermato in precedenza.  
  
4. Esclusione della Garanzia  
  
FATTE SALVE LE GARANZIE INDEROGABILI DI LEGGE, SE
ESISTENTI, IBM NON FORNISCE GARANZIE O DICHIARAZIONI, DI ALCUN TIPO,
ESPRESSE O IMPLICITE, INCLUSE, A TITOLO ESEMPLIFICATIVO, GARANZIE O
CONDIZIONI IMPLICITE DI QUALITÀ SODDISFACENTE, COMMERCIABILITÀ O
IDONEITÀ PER UNO SCOPO PARTICOLARE, INCLUSE LE GARANZIE DI
TITOLARITA' E DI FUNZIONAMENTO ININTERROTTO, RELATIVE AL PROGRAMMA O AL
SUPPORTO TECNICO, SE ESISTENTE.  
  
La presente esclusione si applica anche a qualsiasi
sviluppatore e fornitore dei Programmi IBM.  
  
Produttori, fornitori o editori di Programmi non IBM
possono prestare proprie garanzie.  
  
5. Limitazione di Responsabilità  
  
Possono verificarsi circostanze in cui, per fatto
imputabile ad IBM o per altri fatti di cui IBM debba rispondere, si
abbia il diritto ad un risarcimento da parte IBM.
Indipendentemente da ciò che dà titolo all'utente a richiedere il
risarcimento danni ad IBM, (inclusi colpa grave, negligenza, erronee
dichiarazioni o altri fatti di natura contrattuale o extracontrattuale),
la responsabilità di IBM è limitata 1) ai danni fisici alla
persona (incluso il decesso), danni alla proprietà immobiliare e
personale e 2) nel caso di ogni altro danno diretto, fino alla cifra
complessiva di U.S. $25.000 (o l'equivalente nella valuta locale) per
qualsiasi richiesta di risarcimento danni. Questa limitazione di
responsabilità si applica anche agli sviluppatori del Programma IBM e ai
fornitori. Ciò rappresenta il massimo per cui IBM ed i suoi fornitori
sono collettivamente responsabili.  
  
IN NESSUN CASO IBM, GLI SVILUPPATORI DEL PROGRAMMA O I
FORNITORI SARANNO RESPONSABILI PER QUANTO SEGUE, ANCHE SE INFORMATI
DELLA POSSIBILITÀ DEL LORO VERIFICARSI:  
  
1. PERDITA O DANNEGGIAMENTO DI DATI;  
2. DANNI SPECIALI, INCIDENTALI, INDIRETTI O ESEMPLARI O
QUALSIASI ALTRO DANNO ECONOMICO CONSEGUENTE; O  
3. MANCATI GUADAGNI, PERDITA DI OPPORTUNITÀ COMMERCIALE, DI
REDDITO, DI BENEFICI, O MANCATI RISPARMI.  
  
6. Disposizioni Generali  
  
1. Le disposizioni del presente Accordo non pregiudicano in
alcun modo le garanzie legali del consumatore che non possono
essere escluse o limitate per contratto.  
2. Nel caso in cui una qualsiasi clausola del presente
Accordo dovesse essere dichiarata invalida o inefficace, le
restanti clausole del presente Accordo rimarranno pienamente in
vigore ed efficaci.  
3. Non è possibile esportare il Programma o procedere in
alcun senso relativamente al Programma che viola le leggi sul
controllo delle esportazioni applicabili.  
4. Si consente ad International Business Machines
Corporation e alle sue associate di memorizzare ed utilizzare le
informazioni relative ai propri contatti, inclusi nomi, numeri
telefonici aziendali, ed indirizzi e-mail aziendali, laddove vi siano
rapporti commerciali. Tali informazioni saranno elaborate ed
utilizzate nell'ambito delle relazioni commerciali; e potranno essere
fornite agli appaltatori che agiscono per conto di IBM, ai Business
Partner IBM che promuovono, commercializzano e supportano alcuni
prodotti e servizi IBM, e ai cessionari di International Business
Machines Corporation e alle sue associate per usi relativi a tali
relazioni commerciali.  
5. IBM non garantisce che qualsiasi versione del Programma
formalmente rilasciata o resa commercialmente disponibile alla
generalità dei clienti (se esistente), sarà simile o compatibile con
le versioni del Release Preliminare.  
5. Ciascuna parte potrà adire l'autorità giudiziaria in
relazione al presente Accordo solamente entro due anni a far data
dall'evento che ha causato l'azione legale a meno che la legge del
paese non disponga diversamente escludendo la possibilità di
rinuncia o limitazione contrattuale.  
7. Ciascuna parte non sarà ritenuta responsabile per
eventuali inadempimenti dovuti a cause che esulino dal proprio
controllo.  
8. Il presente Accordo non creerà alcun diritto o azione
legale da terze parti, né IBM sarà responsabile di alcun reclamo
da parte di terzi fatto salvo, laddove consentito
dall'articolo Limitazione di responsabilità, per danni fisici (incluso il
decesso) o danni tangibili a proprietà personale per cui IBM è
legalmente responsabile.  
9. Non è possibile assegnare il presente Accordo, in parte
o nella sua interezza, senza previo consenso scritto da parte
di IBM. Ogni tentativo di fare ciò non è valido.  
  
7. Legge regolatrice e Giurisdizione  
  
Legge regolatrice  
  
Le parti concordano sull'applicazione delle leggi del paese
in cui è stata ottenuta la licenza del Programma al fine di
disciplinare, interpretare ed applicare tutti i diritti e le obbligazioni
che derivano, o in qualche modo sono correlati al presente
Accordo, indipendentemente dal conflitto di principi di legge.  
  
La Convenzione delle Nazioni Unite sui Contratti per la
Vendita Internazionale di Merci non è applicabile.  
  
Giurisdizione  
  
Tutti i diritti, i doveri e le obbligazioni sono soggetti
agli organi giudiziari del paese in cui è stata ottenuta la
licenza al Programma.  
  
Parte 2 - Disposizioni specifiche per i singoli Paesi  
  
EUROPA, MEDIO ORIENTE, AFRICA (EMEA)  
Diritti relativi ai Dati (Articolo 3): In EMEA, il testo
seguente sostituisce integralmente le disposizioni di questo
articolo:  
  
Si assegna ad IBM ogni diritto, titolarità ed interesse su
scala mondiale (inclusa la proprietà dei diritti d'autore)
relativo a dati, suggerimenti e materiale cartaceo che 1) è relativo
all'utilizzo del Programma e 2) è fornito ad IBM. Tale assegnazione di
diritti include, ma non è limitata all'assegnazione dei diritti di
preparare e far preparare opere derivate dei materiali cartacei, e di
utilizzare, far utilizzare, eseguire, riprodurre, trasmettere,
visualizzare, elaborare, trasferire, distribuire e concedere in licenza i
materiali scritti e tali opere derivate con qualsiasi supporto o
tecnologia di distribuzione, e di concedere agli altri tutti o parte
dei diritti qui garantiti, per la durata di tali diritti,
titolarità ad interessi. Se IBM lo richiede, si firmerà un documento
appropriato per assegnare tali diritti. Relativamente a qualsiasi idea,
know-how, concetto, tecnica, invenzione, scoperta o
miglioramento, brevettabili o meno, relativo al Programma e rilasciato
dall'utente o dai suoi dipendenti durante il periodo di valutazione, si
concede ad IBM un diritto ed una licenza non esclusivi,
irrevocabili, privi di limitazioni, su scala mondiale e pagati di
includere quanto precedentemente detto in qualsiasi prodotto o
servizio e di utilizzare, produrre e vendere uno qualsiasi di tali
prodotti o servizi e di consentire a terzi di procedere secondo
quanto affermato in precedenza. Nessuna parte richiederà all'altra
il pagamento dei corrispettivi per i diritti sui dati o su
qualsiasi lavoro effettuato in base al presente Accordo.  
  
Esclusione della garanzia (Articolo 4): Nell'Unione
Europea, quanto segue viene aggiunto all'inizio del presente
articolo:  
  
Nell'Unione Europea, i consumatori hanno diritti legali in
base alla legislazione nazionale applicabile che regola la
vendita dei beni al consumatore. Quanto descritto nell'Articolo 4
non riguarda tali diritti.  
  
Limitazione di responsabilità (Articolo 5): In Italia e
Svizzera, il testo seguente sostituisce integralmente le disposizioni
di questo articolo:  
  
Fatto salvo quanto stabilito da norme inderogabili di legge:  
  
1. La responsabilità di IBM per qualsiasi danno e perdita
derivante dall'adempimento dei propri obblighi in base a o in
associazione con il presente accordo o dovuta ad altre cause correlate
all'accordo è limitata solo alla compensazione di quei danni e quelle
perdite provate e realmente derivanti come conseguenza diretta del
mancato adempimento di tali obblighi (se è colpa di IBM) o di tale
causa, per un ammontare massimo che non può superare in alcun caso
?25.000.  
La suddetta limitazione non si applicherà ai danni alle
persone (incluso il decesso) e ai danni al patrimonio e alla
proprietà privata per cui IBM è legalmente responsabile.  
2. IN NESSUN CASO IBM, O UNO QUALSIASI DEI SUOI
SVILUPPATORI DI PROGRAMMI, SARÀ RESPONSABILE DEI SEGUENTI EVENTI, ANCHE
SE INFORMATA DELLA POSSIBILITÀ DEL LORO VERIFICARSI: 1)
PERDITA O DANNEGGIAMENTO DI DATI; 2) DANNI INCIDENTALI O INDIRETTI,
O QUALSIASI DANNO ECONOMICO CONSEGUENTE; 3) LUCRO CESSANTE,
ANCHE SE DERIVANTE COME IMMEDIATA CONSEGUENZA DALL'EVENTO CHE HA
PRODOTTO IL DANNO; O 4)MANCATI AFFARI, PERDITA COMMERCIALE, DI
REDDITO, DI BENEFICI, O MANCATI RISPARMI.  
3. La limitazione ed esclusione di responsabilità qui
accettata si applica non solo alle attività eseguite da IBM, ma anche
a quelle eseguite dai suoi fornitori e sviluppatori di
Programmi, e rappresenta l'ammontare massimo per il quale IBM, i suoi
fornitori e sviluppatori di Programmi, sono complessivamente
responsabili.  
  
Legge regolatrice e Giurisdizione (Articolo 7)  
  
Giurisdizione  
  
Le seguenti eccezioni si aggiungono a questo articolo:  
  
In Italia qualsiasi pretesa legale derivante dal presente
Accordo verrà portata dinnanzi e discussa esclusivamente dal foro
di Milano.  
  
ITALIA: Disposizioni Generali (Articolo 6): Quanto segue si
aggiunge a questo articolo:  
  
IBM e l'utente originario (di seguito, individualmente,
"Parte") soddisferanno tutti gli obblighi delle disposizioni e/o
regolamenti di legge applicabili sulla protezione dei dati personali.
Ciascuna delle Parti indennizzerà e proteggerà l'altra da qualsiasi
danno, pretesa, costo o spesa derivante, direttamente o
indirettamente, dalla violazione delle disposizioni e/o regolamenti di
legge commesse dall'altra Parte.  
  
SVIZZERA: Disposizioni Generali (Articolo 6): Quanto segue
si aggiunge al punto 4:  
  
Per quanto concerne questa clausola, le informazioni
relative ai contatti includeranno anche informazioni sull'utente
originario in qualità di persona giuridica, ad esempio dati relativi
al reddito ed altre informazioni che riguardano la fornitura.  
  
Z125-5544-03 (10/2005)  
INFORMAZIONI DI LICENZA  
  
I Programmi descritti di seguito sono forniti in licenza in
base alle seguenti disposizioni che integrano quelle di Accordo
di Licenza Internazionale per Release Preliminare di Programmi
(ILAR).  
  
Nome Programma: alphaWorks Emerging Technology  
Numero Programma: N/A  
  
Ambiente Operativo Specificato  
  
Le informazioni sulle Specifiche del Programma e
sull'ambiente operativo specificato sono contenute nella documentazione
che accompagna il Programma, se disponibile, ad esempio un file
read-me, o in altre informazioni pubblicate da IBM, ad esempio
una lettera d'annuncio.  
  
Periodo di Valutazione  
  
Il periodo di valutazione ha inizio alla data in cui si
accettano le clausole del presente Accordo e termina dopo 90 giorni.  
  
D/N: L-JLCO-6HQ6QK  
P/N: L-JLCO-6HQ6QK   
