# Supplementary material for: Finding New Order in Biological Functions from the Network Structure of Gene Annotations
Source: PLoS Comput Biol. 2015 Nov 20;11(11):e1004565. doi: 10.1371/journal.pcbi.1004565 (PMC4654495; doi:10.1371/journal.pcbi.1004565)
Supplement: S1 Code — This file contains the input human annotation files and all the code needed to reproduce the analyses and figures presented in this manuscript. The complete collection of intermediate files (such as the predicted term-term networks, word clouds for all communities, etc), can be obtained from [34]. (TGZ) [file pcbi.1004565.s004.tgz › TermCommunities_code/MakeCloudFiles/IBM Word Cloud/license/ja.html]

Software License

vOÌúñÉÖ·é²gpð  
  
æ 1 Í - ¤Ê  
  
{uvOÌúñÉÖ·é²gpðv(ÈºugpðvÆ¢¢Ü·B) ÍA¨qlÆ IBM ÆÌÔÅ÷³êé@
IÈ\_ñÅ·B ¨qlªuvOvð\_E[hA±üA¡»AANZX é¢Ígp³êéÆA{gpðÉ¯Ó³ê½àÌ
Æ©È³êÜ·B ¼ÌÂlAïÐ é¢Í@lÉãíÁÄ±ÌgpðÉ¯Ó³êéêÍA¨qlÍ»êçÌÂlAïÐ é¢Í@
lÉ±Ìgpððç³¹éS ÀðLµÄ¢é±Æð\¾EÛØ¢½¾­àÌÆµÜ·B  
  
uúñv ÆÍAi1jÜ¾J­iµ½ªÁÄM«ªá¢êª èÜ·BjAÜ½Íi2jùÉJ­ÅÍÈ¢ªÜ¾[
U[ÉÍ¤ÆIñª×³êÄ¢È¢vOÌ­\Ì±ÆÅ·B  
  
uIBMv ÆÍAInternational Business Machines Corporation Ü½Í»Ì¼Ú
àµ­ÍÔÚÌqïÐð¢¢Ü·B  
  
uCZXîñv (ÈºuLIvÆ¢¢Ü·B) ÆÍAuvOvÉÅLÌîñ¨æÑððñ·é¶ð¢¢Ü
·B uvOvÌ LI ÍAuvOvÌfBNg[àÌt@CÆµÄ (VXeER}hðgpµÜ·B)AÜ½
ÍuvOvÉYt³êé¬ûqÆµÄñ³êÜ·B   
  
uvOv ÍAvOÌ´{¨æÑ»Ì·×ÄÌ¡»¨iSÌ¡»©ª¡»©ðâ¢Ü¹ñBjðÜßÄAÌ¢¸ê©
àµ­Í»ÌgÝí¹ðÓ¡µÜ·B 1) @BÅÇÝæè¤é`Ì½ß¨æÑf[^A2) lªÇÝæêé`Ì\tgEFAÌ
\¬vfA3) ®oRec (C[WAeLXgA^¹AæÈÇjA4) ÖA·éCZX¿A5) CZXE
[XEhLgÜ½ÍL[A(6) ÖA¶AÈçÑÉ (7) IBM ªÆ©Ì»fÅ (ºLLÚÌ)uT|[gvÆµÄ
¨qlÉñ·ég£AXVAÜ½Í¿B  
  
u¨qlv ÆÍAÂlÜ½ÍÐÆÂÌ@lðÓ¡µÜ·B  
  
{gpðÍAæ 1 Í¤ÊðAæ 2 ÍÌeÅLÌð (Y·éê)ALI ©ç\¬³êA{uvOvÌg
pÉÖ·é¼ÒÔÌ®SABêÌÓ¶Å·B ¨qlÌuvOvÌgpÉÖ·éAOÌ¼ÒÌûªÜ½ÍÊÉæ
éÊmÌ·×ÄÉãíéàÌÅ·B æ 2 Í¨æÑ LI ÉAæ 1 ÍÌðÉÎ·éÇÁðÜ½ÍÏXðªLÚ³êÄ
¢éêª èÜ·B  
  
1. gp   
  
uvOvÍAIBM Ü½ÍuvOvÌñÒªL ðLµÄ¢Ü·BuvOvÍAì ÉæèÛì³êÄ¨
èAgpø³êéàÌÅ ÁÄAÌÎÛÆÈéàÌÅÍ èÜ¹ñB  
  
IBM Í¨qlÉÎµÄAuvOvÌ§Àt«ÅñÆèIÈ÷ns\Ìgp ðøµÜ·BuvOvÌ]¿úÔA
¨qlÍA¨qlÌÐàÉ¨¯éeXg¨æÑ]¿AÈçÑÉ IBM ÉÎ·étB[hobNðñ·éÚIÉÀèAuvO
vð\_E[hA±üA¨æÑgp·é±ÆªÅ«Ü·B  
  
¨qlÍA©©égpÌÍÍÉ¨¢ÄAuvOvÌobNAbvERs[ðì¬·é±ÆªÅ«Ü·B¨qlÍA¶YIÚIÅ
uvOvðgp·é±ÆAuvOvÜ½ÍuvOvÌêðzz·é±ÆÍÅ«Ü¹ñB  
  
¨qlÍAuvOvðüÏ·é±ÆAuvOvÌñIì¨ðì¬·é±ÆàÅ«Ü¹ñB  
  
{gpðÍA¨qlªì¬·éuvOvÌe¡»¨ÉàKp³êÜ·B  
  
uvOvÌ¡»¨ÉÍAS¡»©ª¡»©ðâí¸A¨qlÍuvOvÉ\¦³êÄ¢éàÌÆ¯êÌì \¦»
Ì¼ÌL \¦ðK¸sÈ¤àÌÆµÜ·B  
  
¨qlÍA1) uvOvÌ¡»{ÌL^ðÛ¶EÇµA©Â 2) un©çÌANZXðÜßÄuvOvðg
p·é½làA¨qlÉFßçê½gpøÍÍàÅÌÝgpµA©ÂA{gpðÉèßé`±ðçéæ¤KØÈ[uðu¶éàÌÆ
µÜ·B  
  
¨qlÍA1) {ugpðvÉ¾L³êÄ¢éêð«AuvOvðgpA¡»AüÏAÚ]àµ­Ízz·é±ÆA
2) @¥Ì­sKèÌ éêð«AuvOvðtAZuAtRpCAàµ­ÍlªÇÝ©Æêé`®àµ­ÍÊÌv
O¾êÖ|Ä·é±ÆA3) uvOvðÄgpøAÀÝàµ­ÍÝ^·é±ÆAÜ½ÍA4) æOÒÉT[rXðñ
·é±ÆðÚIÆµÄuvOvðgp·é±ÆÍÅ«Ü¹ñB  
  
{gp ÍA¨qlÉÎµAIBM ©çóü³ê½¶AT|[gAdbÉæéAVX^XAÜ½ÍuvOvÌg£ é¢
ÍXV (ÈºuT|[gvÆÌµÜ·B) Ì ðt^·éàÌÅÍ èÜ¹ñB½¾µAIBM ÍAIBM Æ©Ì»fÉæ
è©©éuT|[gvðñ·é±Æª èÜ·B uT|[gvÌêÂÆµÄ IBM ªñ·é¢©Èég£AXV¨æÑ»Ì¼
Ì¿Í·×ÄAuvOvÌêÆ©È³êA{gpðªKp³êÜ·B  
  
±ÌuvOvÉÍA]¿úÔÌ¹ðàÁÄ©®IÉgpsÂ\ÉÈé@\ðL·éêª èÜ·B ¨qlÍA±Ì©®g
pâ~@\¨æÑuvOvðÏX·é±ÆÍÅ«Ü¹ñB  
  
¨qlÍAuvOvªgpsÂ\ÉÈéOÉf[^ÌobNAbvð¨æè­¾³¢B  
  
2. úÔ  
  
]¿úÔÍA¨qlª±ÌgpðÉ¯Ó³ê½Æ«ÉJnµA1) uCZXîñvÉLÚÌÅIúiLÚª éêBjA
2) uvOvÌ©®gpâ~úAÜ½Í 3) IBM ª³®ÅÌuvOvÌcÆ®ðJnµ½úÌ¢¸ê©ªµ½
úÉI¹µÜ·B ¨qlÌuvOvgp ÍA]¿úÔÌI¹ðàÁÄI¹µA]¿úÔI¹ã 10 úÈàÉAuvO
v¨æÑuvOv©çì¬³ê½·×ÄÌ¡»¨ðjü·éàÌÆµÜ·B  
  
]¿úÔA¨qlªuvOvðgp·é½ßÌ¿àÍKp³êÜ¹ñB   
  
¨qlª±ÌgpðÉá½µ½êÉÍAIBM Í¨qlÉÎ·égpøðI¹·é±ÆªÅ«Ü·B  
  
±ÌêA¨qlÍAuvOvÌ·×ÄÌ¡»¨ð¬â©Éjü·éàÌÆµÜ·B  
  
3. f[^Ì   
  
¨qlÍA1) ¨qlÌuvOv²gpÉÖµ 2) ¨qlª IBM Éñµ½Af[^AñÄÜ½Í¿ÉÂ¢ÄÌ
 A ´¨æÑ v (ú{ì @æQVð¨æÑæQWðÌ ðÜÞ·×ÄÌxª ð¹¹½ì ðÜÝÜ·B) Ì·×
Äð IBM É÷n·éàÌÆµÜ·B  
  
Ü½A¨qlÍAIBM ªKvÆ·éê©©é Ì÷nÉKvÈ¶É¼·éàÌÆµÜ·B  
  
{Ræê¶ÉîÃ­¨qlÉæé÷nÌÎÛÅÈ¢êA¨qlª IBM Éñ·éuvOvÉÖ·éACfAAmEn
EARZvgAZpA­¾A­©Ü½ÍüÇÉÖµÄA¨qlÍAÁæ¾Â\«ÌL³ðâí¸A»iÜ½ÍT[rXÉÜßAgp
µA»¢µAÌ·é±ÆðÜÞÀ{ðµAÜ½æOÒÉÀ{³¹é±ÆªÅ«éñÆèIAæÁµs\Ì§ÀÌÈ¢S¢EÉí½éx¥
ÏÝÌ ¨æÑÀ{ ð IBM Éø·éàÌÆµÜ·B  
  
4. ÛØÌsKp  
  
­s@KÉ½·éêð«AIBM ÍuvOvðÁè¨ÆµÄ»¶·éÜÜÌóÔÅñµAuvOvÜ½ÍZpT
|[g (ñ³êéê) ÉÂ¢ÄA@¥ãÌàêárSÛÓCðÜßAæOÒÌ ÌsNQÌÛØAi¿ÌÛØA¤i«ÌÛØAÁè
ÚIK«¨æÑ ´ÌÛØðÜÞ¾¦àµ­ÍÙ¦Ì¢©ÈéÛØÓCàíÈ¢àÌÆµÜ·B   
  
±ÌsñÌKèÍAIBM ÌuvOvJ­Ò¨æÑñÒÉÎµÄàKp³êÜ·B  
  
È¨AIBM uvOvÈOÌuvOvÉÂ¢ÄÍA»Ì»¢ÒAñÒÜ½Í­sÒÉæèÛØªñ³êéêª 
èÜ·B  
  
5. ÓCÌ§À  
  
¨qlª IBM ÌÓÉA·×«R (\_ñssAß¸AsÀ\¦Ü½Ís@s×ðÜÝÜ·B) ÉîÃ­¹QÉÎµÄ~
ÏðßéêAIBM ÌÓCÍA¿Ì´öðâí¸AÌeÉèßéàÌÉÀçêÜ·B  
  
1) IBM ÌÌÓÜ½Íß¸ÉæÁÄ¨qlÉ¶¶½gÌA¶½¨æÑLÌ¨ÉÎ·éÓCB 2) ¨qlÉ»ÀÉ­¶µ
½Êí©Â¼ÚÌ¹QÉÎµA¨qlªZ·éÌÊÝÅz 25,000 ÄhzðÀxÆµ½àKÓCB  
  
{ÌÓCÌ§ÀÍAIBM ÉuvOvðñµ½uvOvJ­Ò¨æÑñÒÉÎ·é¹Q¿ÉàKp³êé
àÌÆµÜ·B  
  
¨qlÍAIBM ÈçÑÉuvOvJ­Ò¨æÑñÒÉÎµÄd¡µÄ¹Qð¿·é±ÆÍÅ«Ü¹ñB  
  
¢©ÈéêÉ¨¢ÄàAIBMAuvOvJ­Ò¨æÑñÒÍA»Ì\©ÌL³ðâí¸­¶µ½ÈºÌ¹QÉÂ¢ÄÍ
ÓCð¢Ü¹ñB  
  
1. f[^Ìr¸Ü½Í¹  
2. ÁÊ¹QAtI¹QAÔÚ¹QA¦±I¹Q¨æÑ»Ì¼Ìgå¹Q  
3. í¸v (rWlXAûvAMp é¢Íßñ·×©èµïpðÜÝÜ·)B  
  
6. »Ì¼  
  
1. ±Ìgp\_ñÍAÁïÒÛì@KÉæé¨qlÌ ðÏX·éàÌÅÍ èÜ¹ñB  
2. ±ÌgpðÌ¢¸ê©Ìðª³øÜ½Ís­§ªÅ«È¢Æ³ê½êÅàA»Ì¼ÌðÍLøÉ¶±·éàÌÆµÜ
·B  
3. ¨qlÍAuvOvÌAoÜ½ÍuvOvÉÖµÄY·éAoÇ@É½·é¢©Èés×ðs¤±ÆàÅ«Ü
¹ñB  
4. ¨qlÍAIBMª¨qlÌAæîñi¼OAïÐÌdbÔAïÐÌdq[EAhXðÜÝÜ·BjðAIBM ª
cÆðs¤næÉÛ¶µgp·é±ÆÉ¯Ó³êéàÌÆµÜ·B  
©©éîñÍ IBM Æ¨qlÆÌæøÉÖAµÄÇAgp³êéàÌÆµAIBM ÌãÆµÄ®·éÏõæAIBM »
i¨æÑT[rXÌÌ£iAÌAxðs¤ IBM rWlXEp[gi[A¨æÑ International
Business Machines Corporation Æ»Ì¼ÚÜ½ÍÔÚÌqïÐÌÆp³æÉÎµÄA©©éæøÖWÆv·épr
Ì½ßÉñ³êé±Æª èÜ·B  
5. IBM ÍA³®Éñàµ­ÍcÆ®³êéuvOvÌo[WªAúñÅÆ¯Ì@\âÝ·«ðL·é±
ÆÉÂ¢ÄÛØµÈ¢àÌÆµÜ·B  
6. ±ÌgpðÉàÆÃ­¿ ÍA¿Ì¢©ñÉ©©íç¸A»Ì´öª­¶µ½ú©ç 2 Nðoßµ½Æ«ÉAøÉ
æèÁÅ·éàÌÆµÜ·B  
7. ¢¸êÌÒàA©ÈÌÓßÉA·±ÆÌÅ«È¢R©ç¶¶½¹QÉÂ¢ÄÍAÓCðíÈ¢àÌÆµÜ·B  
8. ±ÌgpðÉæèæOÒÉÎµÄ¢©Èéi  é¢Í¿ à¶¶éàÌÅÍÈ­AÜ½¨qlÉÎ·éæOÒ©çÌ
¿ÉÂ¢Ä IBM ªÓCð¤àÌÅÍ èÜ¹ñB½¾µAOqÌuÓCÌ§ÀvðÅFßçê½AIBM ª@IÉÓCð
L·éAgÌi¶½ðÜÝÜ·jA¨æÑLÌ¨ÉÎ·éÓCÍ«Ü·B  
9. ¨qlÍASÌÅ êêÅ êAOÉ IBM ©çÊÉæé¯Óð¾é±ÆÈ­A{gpðÉæé ÌSÜ½Í
êð÷n·é±ÆÍÅ«Ü¹ñB »Ìæ¤È¢©ÈéÝà³øÆµÜ·B  
  
7. @¨æÑÙ»Ç   
  
@  
  
¼ÒÍA@´ÌµÉÖ·éêð«A{\_ñ©ç¶¶éAàµ­Í{\_ñÉÖA·é¼ÒÌ·×ÄÌ `±ðAK
§AðßAÀ{·é½ßÉA¨qlªuvOvÌgp ðæ¾·éÌ@¥ðKp·é±ÆÉ¯Ó·éàÌÆµÜ·B  
  
Û\_ñÉÖ·éAðñiUnited Nations Convention on Contracts for
the International Sale of GoodsjÍKp³êÜ¹ñB  
  
Ù»Ç   
  
¼ÒÌ ¨æÑ`±ÉÂ¢ÄÍA¨qlªuvOvÌgp ðæ¾·éÌÙ»Ì»fÉ]¤àÌÆµÜ·B  
  
æ 2 Í - eÅLÌð  
  
AWA¾½mnæ  
  
ú{: æ 6  ( »Ì¼ ): æ 6 ÌãÉÈºÌ¶¾ðÇÁµÜ·B  
  
{ÉÖ·é^`ª¶¶½êÍAÒoûªM`½ÀÌ´¥É]ÁÄ¦c·éàÌÆµÜ·B  
  
Z125-5544-03 (10/2005)  
CZXîñ  
  
ÈºÉ\¦³êÄ¢éuvOvÉÍAvOÌúñÉÖ·é²gpð ¨æÑÌÇÁðÉîÃ¢Ägp ªø
³êÜ·B  
  
vO¼: alphaWorks Emerging Technology  
vOÔ: N/A  
  
IBM èÌÒ®Â«  
  
uvOvÌdl¨æÑ IBM èÌÒ®Â«ÉÂ¢ÄÍAuvOvÆ¤Éñ³êé¶ÉLÚª³êÄ¢Ü·BÜ
½AuREADMEvt@CÜ½Í­\^[ IBM ÉæèöJ³ê½îñÉLÚ³êéêà èÜ·B  
  
]¿úÔ  
  
]¿úÔÍA¨qlª±ÌgpðÉ¯Ó³êéúðàÁÄJn³êAJnúæè 90 úðàÁÄI¹µÜ·B  
  
D/N: L-JLCO-6HQ6QK  
P/N: L-JLCO-6HQ6QK   
