# Supplementary material for: Finding New Order in Biological Functions from the Network Structure of Gene Annotations
Source: PLoS Comput Biol. 2015 Nov 20;11(11):e1004565. doi: 10.1371/journal.pcbi.1004565 (PMC4654495; doi:10.1371/journal.pcbi.1004565)
Supplement: S1 Code — This file contains the input human annotation files and all the code needed to reproduce the analyses and figures presented in this manuscript. The complete collection of intermediate files (such as the predicted term-term networks, word clouds for all communities, etc), can be obtained from [34]. (TGZ) [file pcbi.1004565.s004.tgz › TermCommunities_code/MakeCloudFiles/IBM Word Cloud/license/ko.html]

Software License

ÇÁ·Î±×·¥ ÃÊ±â ¸±¸®½º¿¡ °üÇÑ ¶óÀÌ¼¾½º °è¾à(ILAR)  
  
Á¦ 1 ºÎ - ÀÏ¹Ý Á¶Ç×  
  
º» ÇÁ·Î±×·¥ ÃÊ±â ¸±¸®½º¿¡ °üÇÑ ¶óÀÌ¼¾½º °è¾à("°è¾à")Àº ±ÍÇÏ¿Í IBM°£ÀÇ ¹ý·ü °è¾àÀÔ´Ï´Ù. º»
ÇÁ·Î±×·¥À» ´Ù¿î·Îµå, ¼³Ä¡, º¹»ç, ¾×¼¼½º ¶Ç´Â »ç¿ëÇÔÀ¸·Î½á ±ÍÇÏ´Â º» °è¾àÀÇ Á¶Ç×¿¡ µ¿ÀÇÇÏ°Ô µË´Ï´Ù. ¸¸ÀÏ ±ÍÇÏ°¡
Á¦3ÀÚ °³ÀÎÀÌ³ª È¸»ç ¶Ç´Â ±âÅ¸ ¹ýÀÎÃ¼¸¦ ´ëÇ¥ÇÏ¿© º» °è¾àÀÇ Á¶Ç×À» ½ÂÀÎÇÏ´Â °æ¿ì, ±ÍÇÏ´Â ±ÍÇÏ¿¡°Ô ÇØ´ç Á¦3ÀÚ
°³ÀÎÀÌ³ª È¸»ç ¶Ç´Â ¹ýÀÎÃ¼°¡ ÀÌµé Á¶Ç×À» ÁØ¼öÇÏµµ·Ï ÇÒ ¿ÏÀüÇÑ ±ÇÇÑÀÌ ÀÖÀ½À» º¸ÁõÇÏ°í Áø¼úÇÕ´Ï´Ù.  
  
"ÃÊ±â ¸±¸®½º"¶õ (1) °³¹ß Áß¿¡ ÀÖ°Å³ª( µû¶ó¼­ ½Å·ÚÇÒ ¼ö ¾ø°Å³ª) (2) ´õ ÀÌ»ó °³¹ß Áß¿¡ ÀÖ´Â
°æ¿ì´Â ¾Æ´ÏÁö¸¸ »ó¾÷ÀûÀ¸·Î ÆÇ¸Å °¡´ÉÇÑ °æ¿ì°¡ ¾Æ´Ñ ÇÁ·Î±×·¥ ¸±¸®½º¸¦ ÀÇ¹ÌÇÕ´Ï´Ù.  
  
"IBM"Àº International Business Machines Corporation ¶Ç´Â ±× ÀÚÈ¸»ç
Áß ÇÏ³ª¸¦ ÀÇ¹ÌÇÕ´Ï´Ù.  
  
"¶óÀÌ¼¾½º Á¤º¸"¶õ ÇÁ·Î±×·¥¿¡ ´ëÇÑ Á¤º¸ ¹× Á¶°ÇÀ» Á¦°øÇÏ´Â ¹®¼­¸¦ ÀÇ¹ÌÇÕ´Ï´Ù. º» ÇÁ·Î±×·¥ÀÇ ¶óÀÌ¼¾½º
Á¤º¸´Â ½Ã½ºÅÛ ¸í·ÉÀ» »ç¿ëÇÏ¿© º» ÇÁ·Î±×·¥ÀÇ µð·ºÅä¸® ¶Ç´Â ÇÁ·Î±×·¥¿¡ µ¿ºÀµÈ ¼ÒÃ¥ÀÚ¿¡¼­ È®ÀÎÇÒ ¼ö ÀÖ½À´Ï´Ù.   
  
"ÇÁ·Î±×·¥"ÀÌ¶õ ÇÁ·Î±×·¥ ¿øº» ¹× ¿øº»ÀÇ ÀüÃ¼ ¶Ç´Â ºÎºÐ »çº»À» Æ÷ÇÔÇÏ¿©: 1) ±â°è ÆÇµ¶ °¡´É ÁöÄ§ ¹×
µ¥ÀÌÅÍ, 2) »ç¿ëÀÚ ÀÌ¿ë °¡´É ¼ÒÇÁÆ®¿þ¾î ±¸¼º¿ä¼Ò, 3) À½¼º/¿µ»ó ³»¿ë¹°(¿¹¸¦ µé¾î, ÀÌ¹ÌÁö, ÅØ½ºÆ®, ³ìÀ½
ÀÚ·á ¶Ç´Â ¿µ»ó), 4) °ü·Ã ¶óÀÌ¼¾½º ÀÚ·á, 5) ¶óÀÌ¼¾½º »ç¿ë ¹®¼­ ¶Ç´Â Å°, (6) °ü·Ã ¹®¼­ ¹× (7)
IBMÀÇ Àç·®À¸·Î, Áö¿ø(ÀÌÇÏ¿¡¼­ ¼³¸í)ÀÇ ÀÏºÎ·Î Á¦°øµÉ ¼ö ÀÖ´Â ±â´ÉÈ®Àå(enhancement), ¾÷µ¥ÀÌÆ® ¶Ç´Â
ÀÚ·á.  
  
"±ÍÇÏ"´Â ÇÑ ¸íÀÇ °³ÀÎÀ» ÀÇ¹ÌÇÏ°Å³ª ¶Ç´Â ´ÜÀÏ ¹ýÀÎÃ¼¸¦ ÀÇ¹ÌÇÕ´Ï´Ù.  
  
º» °è¾àÀº º» ÇÁ·Î±×·¥ÀÇ »ç¿ë¿¡ ´ëÇÑ ±ÍÇÏ¿Í IBM°£ÀÇ ¿ÏÀüÇÑ °è¾àÀ¸·Î, Á¦ 1 ºÎ - ÀÏ¹Ý Á¶Ç×, Á¦ 2
ºÎ - ±¹°¡º° °íÀ¯ Á¶Ç×(ÇØ´ç Á¶Ç×ÀÌ ÀÖ´Â °æ¿ì) ¹× ¶óÀÌ¼¾½º Á¤º¸·Î ±¸¼ºµÇ¾î ÀÖ½À´Ï´Ù. º» °è¾àÀÌ º»
ÇÁ·Î±×·¥ »ç¿ë°ú °ü·ÃµÈ ±ÍÇÏ¿Í IBM °£ÀÇ ¸ðµç ÀÌÀü ±¸µÎ ¶Ç´Â ¼­¸é ÀÇ»çÇ¥½Ã¸¦ ´ëÃ¼ÇÕ´Ï´Ù. Á¦ 2 ºÎÀÇ Á¶Ç×°ú
¶óÀÌ¼¾½º Á¤º¸°¡ Á¦ 1 ºÎÀÇ Á¶Ç×À» ´ëÃ¼ ¶Ç´Â ¼öÁ¤ÇÒ ¼ö ÀÖ½À´Ï´Ù.  
  
1. ¶óÀÌ¼¾½º  
  
º» ÇÁ·Î±×·¥ÀÇ ¼ÒÀ¯±ÇÀº IBM ¶Ç´Â IBM °ø±Þ¾÷Ã¼¿¡°Ô ÀÖÀ¸¸ç ÀúÀÛ±ÇÀÇ º¸È£¸¦ ¹Þ½À´Ï´Ù.  
  
IBMÀº ±ÍÇÏ¿¡°Ô Æò°¡ ±â°£ µ¿¾È ³»ºÎ Å×½ºÆ® ¹× Æò°¡ ¸ñÀû°ú IBM¿¡ ÇÇµå¹éÀ» Á¦°øÇÏ±â À§ÇÏ¿© º»
ÇÁ·Î±×·¥À» ´Ù¿î·Îµå, ¼³Ä¡ ¹× »ç¿ëÇÒ ¼ö ÀÖ´Â Á¦ÇÑÀû, ºñµ¶Á¡Àû, ¾çµµºÒ°¡ÇÑ ¶óÀÌ¼¾½º¸¦ ºÎ¿©ÇÕ´Ï´Ù.   
  
±ÍÇÏ´Â ÀÌ·¯ÇÑ »ç¿ëÀ» Áö¿øÇÒ ¸ñÀûÀ¸·Î º» ÇÁ·Î±×·¥ÀÇ ¹é¾÷ »çº»À» ÀÛ¼ºÇÏ¿© ¼³Ä¡ÇÒ ¼ö ÀÖ½À´Ï´Ù. ±ÍÇÏ´Â º»
ÇÁ·Î±×·¥À» ½ÇÁ¦ ¾÷¹«¿ëÀ¸·Î »ç¿ëÇÒ ¼ö ¾øÀ¸¸ç, º» ÇÁ·Î±×·¥ ¶Ç´Â ±× ÀÏºÎ¸¦ ¹èÆ÷ÇÒ ¼ö ¾ø½À´Ï´Ù. ±ÍÇÏ´Â º» ÇÁ·Î±×·¥À»
¼öÁ¤ÇÒ ¼ö ¾øÀ¸¸ç, 2Â÷Àû ÀúÀÛ¹°À» ÀÛ¼ºÇÒ ¼ö ¾ø½À´Ï´Ù. º» ¶óÀÌ¼¾½º Á¶Ç×Àº ±ÍÇÏ°¡ ÀÛ¼ºÇÏ´Â °¢ »çº»¿¡µµ
Àû¿ëµË´Ï´Ù. ±ÍÇÏ´Â º» ÇÁ·Î±×·¥ÀÇ ¸ðµç ÀüÃ¼ »çº» ¶Ç´Â ºÎºÐ »çº»¿¡ ¹Ýµå½Ã ¸ðµç ÀúÀÛ±Ç Ç¥½Ã ¹× ±âÅ¸ ¸ðµç ¼ÒÀ¯±Ç
Ç¥½Ã¸¦ ÇØ¾ß ÇÕ´Ï´Ù.  
  
±ÍÇÏ´Â 1) º» ÇÁ·Î±×·¥ÀÇ ¸ðµç »çº» ±â·ÏÀ» À¯Áöº¸¼öÇÏ¸ç, 2) ·ÎÄÃ ¶Ç´Â ¿ø°ÝÀ¸·Î ¾×¼¼½ºÇÏ¿© º»
ÇÁ·Î±×·¥À» »ç¿ëÇÏ´Â ¸ðµç »ç¿ëÀÚ°¡ ±ÍÇÏ¿¡°Ô »ç¿ëÀÌ Çã°¡µÈ ºÎºÐ¸¸À» »ç¿ëÇÏ°í, ÀÌµé »ç¿ëÀÚ°¡ º» °è¾àÀÇ Á¶Ç×À»
ÁØ¼öÇÏµµ·Ï ÇÒ °ÍÀ» º¸ÁõÇÕ´Ï´Ù.   
  
´ÙÀ½°ú °°Àº ÇàÀ§´Â ±ÝÁöµË´Ï´Ù: 1) º» °è¾à¿¡ ±ÔÁ¤µÈ °æ¿ì¸¦ Á¦¿ÜÇÏ°í º» ÇÁ·Î±×·¥À» »ç¿ë, º¹»ç, ¼öÁ¤
¶Ç´Â ¹èÆ÷ÇÏ´Â ÇàÀ§ 2) º» ÇÁ·Î±×·¥À» ¸®¹ö½º ¾î¼Àºí, ¸®¹ö½º ÄÄÆÄÀÏ ¶Ç´Â »ç¿ëÀÚ°¡ ÀÐÀ» ¼ö ÀÖ´Â Çü½Ä ¶Ç´Â
´Ù¸¥ ÇÁ·Î±×·¥ ¾ð¾î·Î º¯È¯ÇÏ´Â ÇàÀ§(±¸Ã¼ÀûÀ¸·Î °è¾à¿¡ ÀÇÇØ Æ÷±â ¶Ç´Â Á¦ÇÑµÉ ¼ö ¾ø´Â °­Çà ¹ý±Ô¿¡¼­ Çã¿ëµÇ´Â
°æ¿ì Á¦¿Ü) 3) º» ÇÁ·Î±×·¥À» Àç»ç¿ë±Ç ºÎ¿©(sublicense), ´ë¿© ¶Ç´Â ¸®½ºÇÏ´Â ÇàÀ§ ¶Ç´Â 4) ¸ÞÀÏ
¼­ºñ½º¸¦ Á¦°øÇÏ´Â ±â°ü(service-bureau)¿¡¼­ º» ÇÁ·Î±×·¥À» »ç¿ëÇÏ´Â ÇàÀ§.  
  
±ÍÇÏ¿¡ ´ëÇÑ ÇÏµåÄ«ÇÇ ¹®¼­, Áö¿ø, ÀüÈ­»ó Áö¿ø, ÇÁ·Î±×·¥¿¡ ´ëÇÑ ±â´ÉÈ®Àå(enhancement) ¶Ç´Â
¾÷µ¥ÀÌÆ®ÀÇ Á¦°ø(ÀÌÇÏ ÅëÄªÇÏ¿© "Áö¿ø")Àº º» ¶óÀÌ¼¾½º¿¡ Æ÷ÇÔµÇÁö ¾Ê½À´Ï´Ù. ±×·¯³ª IBMÀº Àç·®À¸·Î ÀÌ¿Í °°Àº
Áö¿øÀ» Á¦°øÇÏ±â·Î °áÁ¤ÇÒ ¼ö ÀÖ½À´Ï´Ù. IBMÀÌ Áö¿øÀÇ ÀÏºÎ·Î Á¦°øÇÏ´Â ±â´ÉÈ®Àå(enhancement),
¾÷µ¥ÀÌÆ® ¹× ±âÅ¸ ÀÚ·á´Â º» ÇÁ·Î±×·¥ÀÇ ÀÏºÎ·Î °£ÁÖµÇ¹Ç·Î º» °è¾àÀÌ Àû¿ëµË´Ï´Ù.  
  
º» ÇÁ·Î±×·¥¿¡´Â Æò°¡ ±â°£ÀÌ ³¡³ª¸é º» ÇÁ·Î±×·¥ÀÇ »ç¿ëÀ» ¸·´Â ÀÛµ¿ ÁßÁö Á¦¾î ÀåÄ¡°¡ µé¾î ÀÖÀ» ¼ö
ÀÖ½À´Ï´Ù. ±ÍÇÏ´Â ÀÌ ÀÛµ¿ ÁßÁö Á¦¾î ÀåÄ¡³ª º» ÇÁ·Î±×·¥À» ¹«´Ü º¯°æÇÒ ¼ö ¾ø½À´Ï´Ù. ±ÍÇÏ´Â º» ÇÁ·Î±×·¥ÀÇ »ç¿ëÀÌ
ÁßÁöµÇ¾úÀ» ¶§ ÃÊ·¡µÉ ¼ö ÀÖ´Â µ¥ÀÌÅÍÀÇ ¼Õ½Ç¿¡ ÁÖÀÇÇØ¾ß ÇÕ´Ï´Ù.  
  
2. ±â°£  
  
Æò°¡ ±â°£Àº ±ÍÇÏ°¡ º» °è¾àÀÇ Á¶Ç×¿¡ µ¿ÀÇÇÏ¸é ½ÃÀÛµÇ¾î 1) º» ¶óÀÌ¼¾½º Á¤º¸¿¡ ÁöÁ¤µÈ Á¾·áÀÏ, 2) º»
ÇÁ·Î±×·¥ÀÌ ÀÚµ¿ÀûÀ¸·Î »ç¿ëÇÒ ¼ö ¾ø°Ô µÇ´Â ÀÏÀÚ ¶Ç´Â 3) IBMÀÌ º» ÇÁ·Î±×·¥À» »ó¾÷ÀûÀ¸·Î ÆÇ¸Å °¡´ÉÇÏ°Ô ÇÏ´Â ÀÏÀÚ
Áß ¸ÕÀú µµ´ÞÇÏ´Â ³¯¿¡ Á¾·áµË´Ï´Ù. ±ÍÇÏÀÇ ÇÁ·Î±×·¥ ¶óÀÌ¼¾½º´Â Æò°¡ ±â°£ÀÇ Á¾·á¿Í ÇÔ²² È¿·ÂÀ» »ó½ÇÇÏ¸ç,
±ÍÇÏ´Â Æò°¡ ±â°£ Á¾·á ÈÄ 10ÀÏ ÀÌ³»¿¡ º» ÇÁ·Î±×·¥ ¹× º» ÇÁ·Î±×·¥ÀÇ ¸ðµç »çº»À» ÆÄ±âÇØ¾ß ÇÕ´Ï´Ù.   
  
Æò°¡ ±â°£¿¡´Â ÇÁ·Î±×·¥ »ç¿ë¿¡ ´ëÇØ ¿ä±ÝÀ» ÁöºÒÇÏÁö ¾Ê½À´Ï´Ù.   
  
IBMÀº ±ÍÇÏ°¡ º» °è¾àÀÇ Á¶Ç×À» ÁØ¼öÇÏÁö ¾Ê´Â °æ¿ì, ±ÍÇÏÀÇ ¶óÀÌ¼¾½º¸¦ ÇØÁöÇÒ ¼ö ÀÖ½À´Ï´Ù. IBMÀÌ
±ÍÇÏÀÇ ¶óÀÌ¼¾½º¸¦ ÇØÁöÇÏ´Â °æ¿ì, ±ÍÇÏ´Â º» ÇÁ·Î±×·¥ÀÇ ¸ðµç »çº»À» ÆÄ±âÇØ¾ß ÇÕ´Ï´Ù.  
  
3. µ¥ÀÌÅÍ¿¡ ´ëÇÑ ±Ç¸®  
  
±ÍÇÏ´Â 1) ±ÍÇÏÀÇ º» ÇÁ·Î±×·¥ »ç¿ë°ú °ü·ÃÀÌ ÀÖ°Å³ª 2) IBMÀÌ ±ÍÇÏ¿¡°Ô Á¦°øÇÏ´Â ¸ðµç µ¥ÀÌÅÍ, Á¦¾È¼­
¹× ¼­¸é ÀÚ·á¿¡ ´ëÇÑ ±Ç¸®, ¼ÒÀ¯±Ç ¹× ÀÌÀÍ(ÀúÀÛ±Ç¿¡ ´ëÇÑ ¼ÒÀ¯±Ç Æ÷ÇÔ)À» IBM¿¡°Ô ¾çµµÇÕ´Ï´Ù. IBMÀÌ
¿äÃ»ÇÏ´Â °æ¿ì, ±ÍÇÏ´Â ÀÌ·¯ÇÑ ±Ç¸®¸¦ IBM¿¡°Ô ¾çµµÇÑ´Ù´Â °ÍÀ» Ç¥½ÃÇÏ´Â ¹®¼­¿¡ ¼­¸í ³¯ÀÎÇØ¾ß ÇÕ´Ï´Ù. º» 3Á¶ÀÇ
Ã¹¹øÂ° ¹®Àå°ú º°µµ·Î Á¦°øÇÏ´Â °æ¿ì¸¦ Á¦¿ÜÇÏ°í, ±ÍÇÏ°¡ IBM¿¡ Á¦°øÇÏ´Â ¸ðµç ¾ÆÀÌµð¾î, ³ëÇÏ¿ì, °³³ä
(concept), ±â¼ú, ¹ß¸í, ¹ß°ß ¶Ç´Â °³¼±(Æ¯Çã Ãëµæ ¿©ºÎ¿Í °ü°è¾øÀÌ)¿¡ ´ëÇØ¼­, ±ÍÇÏ´Â IBM¿¡°Ô ÀÚ·á¸¦ Á¦Ç° ¶Ç´Â
¼­ºñ½º¿¡ Æ÷ÇÔÇÏ¿© Á¦Ç° ¶Ç´Â ¼­ºñ½º¸¦ ÀÌ¿ë, Á¦ÀÛ ¹× ¸¶ÄÉÆÃÇÏ´Âµ¥ ÀÌ¿ëÇÏ°í Á¦3ÀÚ¿¡°Ô ÀÌ¿Í °°Àº ±ÇÇÑÀ» Çã°¡ÇÒ ¼ö
ÀÖ´Â ºñµ¶Á¡ÀûÀÌ°í, Ãë¼Ò ºÒ´ÉÀÇ, Á¦ÇÑ¾ø´Â, Àü¼¼°èÀûÀÌ¸ç ÁöºÒÀÌ ¿Ï·áµÈ ±Ç¸® ¹× ¶óÀÌ¼¾½º¸¦ ºÎ¿©ÇÕ´Ï´Ù.   
  
4. ¹«º¸Áõ  
  
°­Çà ¹ý±Ô¿¡ µû¶ó Á¦°øÇÏ´Â º¸ÁõÀÇ °æ¿ì¸¦ Á¦¿ÜÇÏ°í, IBMÀº ÇÁ·Î±×·¥ Áö¿ø ¶Ç´Â ±â¼úÀû Áö¿ø°ú °ü·ÃÇÏ¿©
(ÇØ´çµÇ´Â °æ¿ì) »óÇ°¼º, Æ¯Á¤ ¸ñÀû¿¡ÀÇ ÀûÇÕ¼º ¹× Å¸ÀÎÀÇ ±Ç¸® ºñÄ§ÇØ¿¡ ´ëÇÑ ¹¬½ÃÀû º¸Áõ ¶Ç´Â Á¶°ÇÀ» Æ÷ÇÔÇÏ¿©(´Ü,
ÀÌ¿¡ ÇÑÇÏÁö ¾ÊÀ½) ¸í½ÃÀûÀÌ°Å³ª ¹¬½ÃÀûÀÎ ÀÏÃ¼ÀÇ º¸Áõ ¶Ç´Â Á¶°ÇÀ» Á¦°øÇÏÁö ¾Ê½À´Ï´Ù.  
  
ÀÌ·¯ÇÑ Á¦¿Ü»çÇ×Àº IBMÀÇ ¸ðµç ÇÁ·Î±×·¥ °³¹ßÀÚ ¹× °ø±ÞÀÚ¿¡°Ôµµ Àû¿ëµË´Ï´Ù.   
  
ºñIBM ÇÁ·Î±×·¥ÀÇ Á¦Á¶ÀÚ³ª °ø±ÞÀÚ ¶Ç´Â ¹ßÇàÀÚ´Â µ¶ÀÚÀûÀÎ º¸ÁõÀ» Á¦°øÇÒ ¼öµµ ÀÖ½À´Ï´Ù.  
  
5. Ã¥ÀÓ Á¦ÇÑ»çÇ×  
  
IBMÀÇ °è¾à ºÒÀÌÇà ¶Ç´Â ±âÅ¸ ±ÍÃ¥ »çÀ¯·Î ±ÍÇÏ¿¡°Ô ¼ÕÇØ°¡ ¹ß»ýÇÑ °æ¿ì, ±ÍÇÏ´Â IBMÀ¸·ÎºÎÅÍ ¼ÕÇØ
¹è»óÀ» ¹ÞÀ» ¼ö ÀÖ½À´Ï´Ù. ±ÍÇÏ°¡ IBMÀ¸·ÎºÎÅÍ ¹è»óÀ» Ã»±¸ÇÒ ¼ö ÀÖ´Â ¿øÀÎ(°è¾à À§¹Ý ¶Ç´Â °ú½Ç, ÇãÀ§ Áø¼ú,
±âÅ¸ °è¾à ¶Ç´Â ¹è»ó Ã»±¸ µîÀ» Æ÷ÇÔ)¿¡ °ü°è¾øÀÌ IBMÀº ´ÙÀ½¿¡ ÇÑÇÏ¿© Ã¥ÀÓÀ» Áý´Ï´Ù. 1) ½ÅÃ¼ »óÇØ(»ç¸Á
Æ÷ÇÔ)¿Í ºÎµ¿»ê°ú À¯Ã¼µ¿»êÀÇ ¼ÕÇØ ¹× 2) ÃÑ ÇÕ°è U.S. $25,000(¶Ç´Â ÇØ´ç Áö¿ªÀÇ È¯À²¿¡ µû¸¥ ±Ý¾×)
À» ÇÑµµ·Î ÇÏ´Â ±âÅ¸ Á÷Á¢ÀûÀÎ ½Ç¼ÕÇØ¾×. ÀÌ·¯ÇÑ Á¦ÇÑ»çÇ×Àº IBMÀÇ ÇÁ·Î±×·¥ °³¹ßÀÚ ¹× °ø±ÞÀÚ¿¡°Ôµµ
Àû¿ëµË´Ï´Ù. IBM ÇÁ·Î±×·¥ °³¹ßÀÚ ¹× °ø±ÞÀÚ¿Í IBMÀÇ Ã¥ÀÓÀº °¢ÀÚÀÇ Ã¥ÀÓÀ» ÇÕÇÏ¿© À§ Ã¥ÀÓ ÇÑµµ¸¦ ÃÊ°úÇÒ ¼ö
¾ø½À´Ï´Ù.  
  
IBM, IBM ÇÁ·Î±×·¥ °³¹ßÀÚ ¶Ç´Â °ø±ÞÀÚ´Â, ¼ÕÇØ ¹ß»ýÀÇ °¡´É¼ºÀ» ÅëÁö ¹ÞÀº °æ¿ì¸¦ Æ÷ÇÔÇÑ ¾î¶°ÇÑ
°æ¿ì¿¡µµ, ´ÙÀ½¿¡ ´ëÇÑ Ã¥ÀÓÀÌ ¾ø½À´Ï´Ù:  
  
1. µ¥ÀÌÅÍÀÇ ¼Õ½Ç ¶Ç´Â ¼Õ»ó  
2. Æ¯º° ¼ÕÇØ, ºÎ¼ö ¼ÕÇØ, °£Á¢ ¼ÕÇØ, Â¡¹úÀû ¼ÕÇØ ¶Ç´Â °æÁ¦ÀûÀÎ °á°úÀû ¼ÕÇØ  
3. ±â´ëÇß´ø ÀÌÀÍ, »ç¾÷, ¼öÀÍ, ¿µ¾÷±Ç ¶Ç´Â ºñ¿ë Àý°¨ÀÌ ½ÇÇöµÇÁö ¸øÇÔÀ¸·Î ÀÎÇØ ¹ß»ýÇÏ´Â ¼ÕÇØ.  
  
6. ÀÏ¹Ý Á¶Ç×  
  
1. º» °è¾àÀÇ ¾î¶°ÇÑ Á¶Ç×µµ °è¾à¿¡ ÀÇÇØ Æ÷±â ¶Ç´Â Á¦ÇÑµÉ ¼ö ¾ø´Â °­Çà ¹ý±Ô »óÀÇ ¼ÒºñÀÚ ±Ç¸®¿¡ ¿µÇâÀ»
¹ÌÄ¡Áö ¾Ê½À´Ï´Ù.   
2. º» °è¾àÀÇ ÀÏºÎ Á¶Ç×ÀÌ ¹«È¿ÀÌ°Å³ª ½ÃÇàÀÌ ºÒ°¡´ÉÇÑ °æ¿ì¿¡µµ, º» °è¾àÀÇ ³ª¸ÓÁö Á¶Ç×Àº ¿ÏÀüÈ÷
À¯È¿ÇÕ´Ï´Ù.  
3. ±ÍÇÏ´Â ÇÁ·Î±×·¥À» ¼öÃâÇÒ ¼ö ¾øÀ¸¸ç ¼öÃâ ±ÔÁ¦ ¹ý·üÀ» À§¹ÝÇÒ ¼ö ÀÖ´Â ÇàÀ§¸¦ ÇÏÁö ¾Ê½À´Ï´Ù.   
4. ±ÍÇÏ´Â IBM ¹× ±× °è¿­»ç°¡ ¿µ¾÷À» ÇÏ´Â °÷¿¡¼­´Â ¾îµð¼­³ª ±ÍÇÏÀÇ ´ã´çÀÚ Á¤º¸(ÀÌ¸§, ÀüÈ­¹øÈ£ ¹×
ÀüÀÚ¿ìÆí ÁÖ¼Ò Æ÷ÇÔ)¸¦ ÀúÀåÇÏ°í »ç¿ëÇÒ ¼ö ÀÖµµ·Ï Çã¿ëÇÒ °Í¿¡ µ¿ÀÇÇÕ´Ï´Ù. ÀÌ·¯ÇÑ Á¤º¸´Â ±ÍÇÏ¿Í IBM°£ÀÇ ¿µ¾÷
°ü°è¸¦ À§ÇÏ¿© Ã³¸®µÇ°Å³ª »ç¿ëµÉ °ÍÀÌ¸ç, IBMÀÇ ÇÏµµ±ÞÀÚ, IBM Á¦Ç° ¹× ¼­ºñ½º¸¦ ÆÇ¸Å, ¿µ¾÷ ÃËÁø ¹×
Áö¿øÇÏ´Â IBM ºñÁî´Ï½º ÆÄÆ®³Ê, IBMÀÇ ¾çµµÀÎ ¹× ÀÚÈ¸»çÀÇ ¿µ¾÷ È°µ¿À» À§ÇØ Á¦°øµÉ ¼ö ÀÖ½À´Ï´Ù.   
5. IBMÀº ÇÁ·Î±×·¥ÀÌ Á¤½ÄÀ¸·Î ¸±¸®½ºµÇ°Å³ª »ó¾÷ÀûÀ¸·Î ÆÇ¸Å °¡´ÉÇÏ°Ô µÇ´õ¶óµµ, ÇØ´ç ÇÁ·Î±×·¥ÀÌ ÃÊ±â
¸±¸®½º¿Í À¯»çÇÏ°Å³ª ÀÌ¿Í ÀÏÄ¡ÇÒ °ÍÀÌ¶ó´Â °ÍÀ» º¸ÁõÇÏÁö ¾Ê½À´Ï´Ù.  
6. °è¾à¿¡ ÀÇÇØ Æ÷±â ¶Ç´Â Á¦ÇÑµÉ ¼ö ¾ø´Â °­Çà ¹ý±Ô¿¡¼­ ´Þ¸® Á¤ÇÏÁö ¾Ê´Â ÇÑ, º» °è¾àÀÇ ¾ç ´ç»çÀÚ´Â
¼Ò¼Û Á¦±âÀÇ ¿øÀÎÀÌ ¹ß»ýÇÑ Áö 2³âÀÌ °æ°úÇÑ ÈÄ¿¡´Â º» °è¾à¿¡ ÀÇ°ÅÇÑ ¹ý·üÀû ¼Ò¼ÛÀ» Á¦±âÇÒ ¼ö ¾ø½À´Ï´Ù.   
7. ¾ç ´ç»çÀÚ´Â ÅëÁ¦ ºÒ°¡´ÉÇÑ »çÀ¯·Î ÀÇ¹«»çÇ×À» ÀÌÇàÇÏÁö ¸øÇÑ °æ¿ì, ÀÌ¿¡ ´ëÇØ ¸éÃ¥µË´Ï´Ù.  
8. º» °è¾àÀº Á¦3ÀÚ¸¦ À§ÇÑ ¾î¶°ÇÑ ±Ç¸®³ª ¼Ò¼Û Á¦±âÀÇ ¿øÀÎÀ» Á¦°øÇÏÁö ¾ÊÀ¸¸ç, IBMÀº Á¦3ÀÚ°¡
±ÍÇÏ¿¡°Ô Á¦±âÇÏ´Â ¾î¶°ÇÑ Ã»±¸¿¡ ´ëÇØ¼­µµ Ã¥ÀÓÀ» ÁöÁö ¾Ê½À´Ï´Ù. ´Ü, »ó±â Ã¥ÀÓ Á¦ÇÑ»çÇ×¿¡¼­ ¾ð±ÞÇÑ ´ë·Î, ¹ýÀûÀ¸·Î
IBM¿¡°Ô Ã¥ÀÓÀÌ ÀÖ´Â ½ÅÃ¼ »óÇØ(»ç¸Á Æ÷ÇÔ)³ª ºÎµ¿»ê ¹× À¯Ã¼µ¿»êÀÇ ¼ÕÇØÀÇ °æ¿ì´Â IBMÀÌ Ã¥ÀÓÀ» Áý´Ï´Ù.   
9. ±ÍÇÏ´Â, IBMÀÇ »çÀü ¼­¸é µ¿ÀÇ¾øÀÌ, º» °è¾àÀÇ ÀüºÎ ¶Ç´Â ÀÏºÎ¸¦ ¾çµµÇÒ ¼ö ¾ø½À´Ï´Ù. ÀÌ¸¦ À§ÇÑ
¸ðµç ÇàÀ§´Â ¹«È¿ÀÔ´Ï´Ù.  
  
7. ÁØ°Å¹ý ¹× °üÇÒ±Ç  
  
ÁØ°Å¹ý   
  
±ÍÇÏ¿Í IBM ¾ç ´ç»çÀÚ´Â º» °è¾àÀ¸·ÎºÎÅÍ ¶Ç´Â º» °è¾à°ú °ü·ÃÇÏ¿© ¹ß»ýÇÑ ±ÍÇÏ¿Í IBMÀÇ ¸ðµç ±Ç¸® ¹×
ÀÇ¹«¸¦ ÅëÁ¦, ÇØ¼® ¹× °­Á¦ÇÏ´Â µ¥ ÀÖ¾î ÁØ°Å¹ý °áÁ¤ÀÇ ¿øÄ¢¿¡ °ü°è¾øÀÌ ±ÍÇÏ°¡ º» ÇÁ·Î±×·¥ ¶óÀÌ¼¾½º¸¦ ÃëµæÇÑ
±¹°¡ÀÇ ¹ý·üÀÌ Àû¿ëµÈ´Ù´Â °Í¿¡ µ¿ÀÇÇÕ´Ï´Ù.  
  
±¹Á¦Àû ¹°Ç°¸Å¸Å °è¾à¿¡ ´ëÇÑ À¯¿£ Á¶¾à(United Nations Convention on
Contracts for the International Sale of Goods)´Â Àû¿ëµÇÁö ¾Ê½À´Ï´Ù.  
  
°üÇÒ±Ç   
  
±ÍÇÏ¿Í IBMÀÇ ¸ðµç ±Ç¸® ¹× ÀÇ¹«¿¡ ´ëÇØ¼­´Â ±ÍÇÏ°¡ º» ÇÁ·Î±×·¥ ¶óÀÌ¼¾½º¸¦ ÃëµæÇÑ ±¹°¡ÀÇ ¹ý·üÀÌ
Àû¿ëµË´Ï´Ù.  
  
Á¦ 2 ºÎ - ±¹°¡º° °íÀ¯ Á¶Ç×  
  
ÇØ´çµÇ´Â ±¹°¡º° °íÀ¯ Á¶Ç×ÀÌ ¾ø½À´Ï´Ù.  
  
Z125-5544-03 (10/2005)  
¶óÀÌ¼¾½º Á¤º¸  
  
¾Æ·¡¿¡ ³ª¿­µÈ ÇÁ·Î±×·¥Àº ÇÁ·Î±×·¥ ÃÊ±â ¸±¸®½º¿¡ °üÇÑ ¶óÀÌ¼¾½º °è¾à(ILAR) ¿Ü¿¡ ´ÙÀ½ Á¶°Ç¿¡ ÀÇ°ÅÇÏ¿©
¶óÀÌ¼¾½º°¡ ºÎ¿©µË´Ï´Ù.  
  
ÇÁ·Î±×·¥ ÀÌ¸§: alphaWorks Emerging Technology  
ÇÁ·Î±×·¥ ¹øÈ£: N/A  
  
¸í½ÃµÈ ¿î¿µ È¯°æ  
  
º» ÇÁ·Î±×·¥ÀÇ ¸í¼¼ ¹× ¸í½ÃµÈ ¿î¿µ È¯°æ Á¤º¸´Â º» ÇÁ·Î±×·¥¿¡ µ¿ºÀµÇ´Â ¹®¼­, ¿¹¸¦ µé¾î, read-me
ÆÄÀÏÀÌ³ª, ¹ßÇ¥ ¹®¼­(announcement letter)¿Í °°ÀÌ IBM¿¡¼­ ¹ßÇàÇÏ´Â ±âÅ¸ Á¤º¸¿¡ µé¾î ÀÖ½À´Ï´Ù.  
  
Æò°¡ ±â°£  
  
Æò°¡ ±â°£Àº ±ÍÇÏ°¡ º» °è¾àÀÇ Á¶Ç×¿¡ µ¿ÀÇÇÏ¸é ½ÃÀÛµÇ¾î 90ÀÏ ÈÄ¿¡ Á¾·áµË´Ï´Ù.  
  
D/N: L-JLCO-6HQ6QK  
P/N: L-JLCO-6HQ6QK   
