# Supplementary material for: Finding New Order in Biological Functions from the Network Structure of Gene Annotations
Source: PLoS Comput Biol. 2015 Nov 20;11(11):e1004565. doi: 10.1371/journal.pcbi.1004565 (PMC4654495; doi:10.1371/journal.pcbi.1004565)
Supplement: S1 Code — This file contains the input human annotation files and all the code needed to reproduce the analyses and figures presented in this manuscript. The complete collection of intermediate files (such as the predicted term-term networks, word clouds for all communities, etc), can be obtained from [34]. (TGZ) [file pcbi.1004565.s004.tgz › TermCommunities_code/MakeCloudFiles/IBM Word Cloud/license/pl.html]

Software License

Miêdzynarodowa Umowa Licencyjna na Wstêpne Wydanie Programów  
  
Czêæ 1 - Warunki ogólne  
  
NINIEJSZA MIÊDZYNARODOWA UMOWA LICENCYJNA IBM NA WSTÊPNE
WYDANIE PROGRAMÓW (ZWANA DALEJ "UMOW¥") JEST UMOW¥ ZAWART¥ MIÊDZY
U¯YTKOWNIKIEM I IBM. POBIERANIE, INSTALOWANIE, KOPIOWANIE ORAZ U¯YWANIE
PROGRAMU OZNACZA AKCEPTACJÊ WARUNKÓW NINIEJSZEJ UMOWY.
ZAAKCEPTOWANIE PRZEZ U¯YTKOWNIKA WARUNKÓW NINIEJSZEJ UMOWY W IMIENIU OSOBY
TRZECIEJ (FIZYCZNEJ LUB PRAWNEJ) OZNACZA, ¯E U¯YTKOWNIK GWARANTUJE
POSIADANIE UPRAWNIEÑ DO PODPORZ¥DKOWANIA TAKIEJ OSOBY (FIZYCZNEJ LUB
PRAWNEJ) NINIEJSZYM WARUNKOM.   
  
"Wstêpne Wydanie" oznacza wydanie Programu, który (1) mo¿e
byæ jeszcze w fazie projektowania (i dlatego mo¿e nie byæ w
pe³ni niezawodny) lub (2) mo¿e ju¿ nie byæ w fazie projektowania
i jeszcze nie byæ dostêpny dla u¿ytkowników w ofercie
handlowej.  
  
"IBM" oznacza firmê International Business Machines
Corporation lub jedno z jej przedsiêbiorstw podporz¹dkowanych.  
  
"Informacje licencyjne" to dokument zawieraj¹cy informacje
i warunki specyficzne dla Programu. Informacje licencyjne
dotycz¹ce Programu s¹ dostêpne w katalogu Programu. Mo¿na z nich
skorzystaæ, u¿ywaj¹c komend systemowych. Dokument ten mo¿e byæ równie¿
do³¹czony do Programu w postaci broszury.   
  
"Program" wystêpuje w jednej lub kilku z poni¿szych
postaci, obejmuj¹cych orygina³ oraz pe³ne lub czêciowe kopie: 1)
instrukcji i danych w formie zapisu maszynowego, 2) komponentów
oprogramowania w formie czytelnej dla cz³owieka, 3) zapisów
audiowizualnych (takich jak obrazy, tekst, nagrania lub ilustracje), 4)
odnosz¹cych siê do Programu materia³ów licencjonowanych, 5) dokumentów
dotycz¹cych licencjonowanego u¿ywania i kluczy, (6) dokumentacji
do³¹czonej oraz (7) wszelkich rozszerzeñ, aktualizacji lub materia³ów,
które IBM mo¿e, wed³ug w³asnego uznania, dostarczyæ U¿ytkownikowi
w formie Wsparcia (zgodnie z poni¿szym opisem).   
  
"U¿ytkownik" oznacza albo osobê fizyczn¹, albo osobê prawn¹.  
  
Niniejsza Umowa sk³ada siê z Czêci 1 - Warunki ogólne,
Czêci 2 - Warunki specyficzne dla poszczególnych krajów (jeli
maj¹ zastosowanie) oraz Informacji licencyjnych, które tworz¹
kompletn¹ umowê miêdzy U¿ytkownikiem i IBM dotycz¹c¹ u¿ywania
Programu. Umowa ta zastêpuje wszelkie wczeniejsze ustne lub pisemne
ustalenia dokonane miêdzy U¿ytkownikiem i IBM w zakresie u¿ywania
Programu. Warunki zawarte w Czêci 2 oraz warunki dokumentu
"Informacje licencyjne" mog¹ zast¹piæ lub zmodyfikowaæ warunki Czêci
1.   
  
1. Licencja  
  
Program ten jest w³asnoci¹ IBM lub dostawcy IBM. Program
ten jest chroniony prawem autorskim i stanowi przedmiot umowy
licencyjnej, a nie umowy sprzeda¿y.  
  
IBM udziela U¿ytkownikowi ograniczonej, niewy³¹cznej,
nieprzenoszalnej licencji na pobieranie, instalowanie i u¿ywanie Programu w
okresie próbnego u¿ywania, wy³¹cznie w celu testowania wewn¹trz
przedsiêbiorstwa U¿ytkownika, próbnego u¿ywania i przekazywania opinii do
IBM.   
  
U¿ytkownik ma prawo do sporz¹dzenia kopii zapasowej takiego
Programu w celu realizacji powy¿szych czynnoci. U¿ytkownik nie
jest upowa¿niony do u¿ywania Programu do celów produkcyjnych ani
do dystrybuowania tak Programu, jak i jego czêci. U¿ytkownik
nie ma prawo do modyfikowania Programu ani tworzenia z niego
prac pochodnych. Warunki niniejszej licencji maj¹ zastosowanie
do ka¿dej wykonanej przez U¿ytkownika kopii Programu.
U¿ytkownik musi odtworzyæ wszelkie uwagi dotycz¹ce praw autorskich
oraz wszelkie informacje o prawach w³asnoci na ka¿dej pe³nej
lub czêciowej kopii Programu.   
  
U¿ytkownik 1) bêdzie prowadziæ rejestr wszystkich kopii
Programu oraz 2) zapewni, ¿e ka¿da osoba korzystaj¹ca z Programu
(lokalnie lub zdalnie) dokonuje tego jedynie w ramach posiadanego
upowa¿nienia oraz przestrzega warunków niniejszej Umowy.   
  
U¿ytkownik nie mo¿e 1) u¿ywaæ, kopiowaæ, modyfikowaæ,
przenosiæ ani dystrybuowaæ Programu, z wyj¹tkiem sytuacji
przewidzianych w niniejszej Umowie; 2) deasemblowaæ, dekompilowaæ Programu
ani dokonywaæ jego translacji w inny sposób, z wyj¹tkiem
sytuacji dozwolonych przez bezwzglêdnie obowi¹zuj¹ce przepisy prawa;
3) udzielaæ dalszych licencji na Program, wynajmowaæ go ani
wydzier¿awiaæ; ani te¿ 4) u¿ywaæ Programu do wiadczenia us³ug
mailingowych.   
  
Niniejsza licencja nie upowa¿nia U¿ytkownika do
otrzymywania od IBM dokumentacji w postaci drukowanej, wsparcia, asysty
przez telefon, udoskonaleñ ani aktualizacji dla Programu (zwanych
³¹cznie "Wsparciem"), chocia¿ IBM mo¿e, wed³ug w³asnego uznania,
podj¹æ decyzjê o udzieleniu takiego Wsparcia. Wszelkie
udoskonalenia, aktualizacje i inne materia³y dostarczane przez IBM w
ramach Wsparcia s¹ traktowane jako czêæ Programu i dlatego
podlegaj¹ postanowieniom niniejszej Umowy.   
  
PROGRAM MO¯E ZAWIERAÆ MECHANIZM BLOKUJ¥CY, ZABEZPIECZAJ¥CY
PROGRAM PRZED U¯YWANIEM GO PO ZAKOÑCZENIU OKRESU PRÓBNEGO U¯YWANIA.
U¯YTKOWNIK NIE MA PRAWA DOKONYWAÆ ¯ADNYCH MODYFIKACJI W TAKIM
MECHANIZMIE ANI W SAMYM PROGRAMIE. U¯YTKOWNIK POWINIEN PRZEDSIÊWZI¥Æ
RODKI OSTRO¯NOCI, ABY ZABEZPIECZYÆ SIÊ PRZED UTRAT¥ DANYCH, CO
MO¯E NAST¥PIÆ W SYTUACJI, GDY NIE BÊDZIE MO¯LIWOCI DALSZEGO
U¯YWANIA PROGRAMU.   
  
2. Termin  
  
Okres próbnego u¿ywania rozpoczyna siê z chwil¹, gdy
U¿ytkownik wyrazi zgodê na warunki niniejszej Umowy, a koñczy siê 1) z
dat¹ zakoñczenia okrelon¹ w dokumencie "Informacje licencyjne",
o ile zostanie tam wskazana, 2) z chwil¹, gdy Program
automatycznie wy³¹czy siê sam lub 3) z chwil¹ udostêpnienia Programu
przez IBM w ofercie handlowej, w zale¿noci od tego, która z tych
dat przypadnie wczeniej. Licencja na Program wygasa wraz z
zakoñczeniem okresu próbnego u¿ywania i U¿ytkownik zobowi¹zany jest
zniszczyæ Program oraz wszelkie wykonane z niego kopie w ci¹gu 10
(dziesiêciu) dni po zakoñczeniu okresu próbnego u¿ywania.   
  
W okresie próbnego u¿ywania nie stosuje siê jakichkolwiek
op³at za u¿ywanie Programu.   
  
IBM ma prawo wypowiedzieæ licencjê udzielon¹ U¿ytkownikowi
w przypadku niewype³nienia przez U¿ytkownika warunków
niniejszej Umowy. W razie wypowiedzenia umowy przez IBM, U¿ytkownik
jest zobowi¹zany do zniszczenia wszelkich egzemplarzy Programu.   
  
3. Prawa do danych  
  
U¿ytkownik przenosi na IBM wszelkie prawa (w tym prawa
autorskie), tytu³y i korzyci odnosz¹ce siê do wszelkich danych,
propozycji lub materia³ów w formie pisemnej 1) zwi¹zanych z u¿ywaniem
Programu oraz 2) dostarczonych przez U¿ytkownika do IBM. Na ¿¹danie
IBM U¿ytkownik podpisze odpowiednie dokumenty, aby dokonaæ
przeniesienia takich praw. W zakresie nie pozostaj¹cym w sprzecznoci z
postanowieniami pierwszego zdania niniejszego paragrafu, U¿ytkownik udziela
IBM niewy³¹cznej, nieodwo³alnej, nieograniczonej,
ogólnowiatowej i w pe³ni op³aconej licencji na do³¹czenie do dowolnego
produktu lub us³ugi wszelkich pomys³ów, znajomoci rzeczy (know-
how), koncepcji, technik, wynalazków, odkryæ czy udoskonaleñ,
objêtych ochron¹ patentow¹ lub nie, maj¹cych zwi¹zek z Programem i
dostarczonych do IBM. Powy¿sza licencja obejmuje równie¿ u¿ywanie,
produkowanie i sprzeda¿ wszelkich takich produktów i us³ug oraz zezwala
innym na wykonywanie powy¿szych czynnoci.   
  
4. Brak gwarancji  
  
Z ZASTRZE¯ENIEM EWENTUALNYCH GWARANCJI WYNIKAJ¥CYCH Z
BEZWZGLÊDNIE OBOWI¥ZUJ¥CYCH PRZEPISÓW PRAWA, KTÓRYCH NIE MO¯NA
WYKLUCZYÆ, IBM NIE UDZIELA NA NINIEJSZY PROGRAM CZY TE¯ W ZAKRESIE
WSPARCIA TECHNICZNEGO JAKICHKOLWIEK GWARANCJI, W TYM TAK¯E RÊKOJMI,
ANI TE¯ NIE USTALA JAKICHKOLWIEK WARUNKÓW, WYRANYCH CZY
DOMNIEMANYCH, A W SZCZEGÓLNOCI DOMNIEMANYCH GWARANCJI CZY WARUNKÓW
ZADAWALAJ¥CEJ JAKOCI, PRZYDATNOCI HANDLOWEJ, PRZYDATNOCI DO
OKRELONEGO CELU ANI TE¯ GWARANCJI CZY WARUNKÓW NIENARUSZANIA PRAW
STRON TRZECICH.   
  
Zastrze¿enie to ma równie¿ zastosowanie do wszelkich
programistów i dostawców IBM.  
  
Producenci, dostawcy i wydawcy zajmuj¹cy siê Programami
innymi ni¿ Programy IBM mog¹ dostarczaæ swe w³asne gwarancje.  
  
5. Ograniczenie odpowiedzialnoci  
  
W przypadku wyst¹pienia okrelonych okolicznoci, za które
odpowiedzialnoæ ponosi IBM, a w wyniku których U¿ytkownik dozna³ szkody,
U¿ytkownik bêdzie uprawniony do uzyskania od IBM odszkodowania. Bez
wzglêdu na podstawê, na jakiej U¿ytkownik jest uprawniony do
domagania siê odszkodowania od IBM (w³¹czywszy w to naruszenie
istotnych postanowieñ niniejszej Umowy, niedbalstwo, wprowadzenie w
b³¹d lub inne roszczenia z tytu³u odpowiedzialnoci kontraktowej
lub deliktowej), odpowiedzialnoæ IBM ogranicza siê jedynie do
1) odpowiedzialnoci za uszczerbek na zdrowiu (³¹cznie ze
mierci¹) oraz za szkody wyrz¹dzone w nieruchomociach lub maj¹tku
ruchomym, a 2) w przypadku innych rzeczywicie poniesionych szkód do
wysokoci 25.000 USD (lub równowartoci w walucie krajowej) za ³¹czn¹
wartoæ wszystkich roszczeñ. Niniejsze ograniczenie
odpowiedzialnoci ma zastosowanie tak¿e do programistów i dostawców Programów
IBM. Wskazany powy¿ej zakres odpowiedzialnoci stanowi ca³oæ
odpowiedzialnoci ponoszonej przez IBM.   
  
W ¯ADNYCH OKOLICZNOCIACH IBM ANI TE¯ PROGRAMICI CZY
DOSTAWCY PROGRAMÓW IBM NIE PONOSZ¥ ODPOWIEDZIALNOCI ZA PONI¯SZE
SZKODY, NAWET JELI ZOSTALI POINFORMOWANI O MO¯LIWOCI ICH
WYST¥PIENIA:  
  
1. UTRATÊ LUB USZKODZENIE DANYCH;  
2. SZKODY SZCZEGÓLNE, UBOCZNE, POREDNIE, SZKODY
SPOWODOWANE NARUSZENIEM DÓBR OSOBISTYCH CZY TE¯ SZKODY, KTÓRYCH NIE
MO¯NA BY£O PRZEWIDZIEÆ PRZY ZAWIERANIU UMOWY ANI TE¯  
3. UTRACONE ZYSKI, KONTAKTY HANDLOWE, DOCHODY, REPUTACJÊ
(GOODWILL), ANI TE¯ PRZEWIDYWANE OSZCZÊDNOCI.  
  
6. Postanowienia ogólne  
  
1. Postanowienia zawarte w niniejszej Umowie pozostaj¹ bez
wp³ywu na uprawnienia przys³uguj¹ce konsumentom, które nie mog¹
byæ wy³¹czone lub ograniczone na podstawie bezwzglêdnie
obowi¹zuj¹cych przepisów prawa.   
2. W przypadku niewa¿noci lub niewykonalnoci
któregokolwiek z postanowieñ niniejszej Umowy, pozosta³e postanowienia
niniejszej Umowy zachowuj¹ pe³n¹ moc prawn¹ i skutecznoæ.   
3. U¿ytkownik nie mo¿e eksportowaæ Programu ani podejmowaæ
¿adnych innych dzia³añ w stosunku do Programu, które narusza³yby
przepisy eksportowe.  
4. U¿ytkownik zezwala International Business Machines
Corporation oraz przedsiêbiorstwom afiliowanym International Business
Machines Corporation na przechowywanie swych informacji
kontaktowych, w³¹czaj¹c w to imiona i nazwiska, numery telefonów
s³u¿bowych i adresy poczty telefonicznej, bez wzglêdu na miejsce
prowadzenia dzia³alnoci. Informacje takie bêd¹ przetwarzane i
wykorzystywane w zwi¹zku z prowadzon¹ dzia³alnoci¹; mog¹ byæ one
przekazywane wykonawcom dzia³aj¹cym w imieniu IBM, Partnerom Handlowych
IBM, którzy prowadz¹ promocjê i sprzeda¿ okrelonych produktów i
us³ug IBM oraz udzielaj¹ dla nich wsparcia, a tak¿e
cesjonariuszom International Business Machines Corporation i
przedsiêbiorstwom podporz¹dkowanym w celu wykorzystania w relacjach
handlowych.   
5. IBM nie gwarantuje, ¿e dowolna wersja Programu,
oficjalnie wydana lub udostêpniona w ofercie handlowej, bêdzie
zbli¿ona do Wstêpnie Wydanej wersji Programu czy te¿ bêdzie z ni¹
kompatybilna.   
6. Wszelkie roszczenia zwi¹zane z realizacj¹ niniejszej
Umowy ulegaj¹ przedawnieniu po up³ywie dwóch lat od powstania
przyczyny takich roszczeñ, chyba ¿e bezwzglêdnie obowi¹zuj¹ce
przepisy prawa stanowi¹ inaczej.   
7. Ani U¿ytkownik, ani IBM nie odpowiadaj¹ za
niedope³nienie jakichkolwiek obowi¹zków, jeli przyczyny takiego
niedope³nienia le¿a³y poza ich kontrol¹.  
8. Niniejsza Umowa nie daje stronom trzecim jakichkolwiek
praw ani te¿ podstawy do roszczeñ, a IBM nie ponosi
odpowiedzialnoci za jakiekolwiek roszczenia stron trzecich wobec
U¿ytkownika, chyba ¿e zezwalaj¹ na to warunki powy¿szego paragrafu
"Ograniczenie odpowiedzialnoci" w czêci dotycz¹cej uszczerbku na
zdrowiu (³¹cznie ze mierci¹) lub szkód wyrz¹dzonych w
nieruchomociach lub w maj¹tku ruchomym, za które IBM ponosi
odpowiedzialnoæ prawn¹.   
9. Nie mo¿na dokonywaæ cesji niniejszej Umowy ani w
ca³oci, ani w czêci, bez wczeniejszej pisemnej zgody IBM. Ka¿da
taka próba bêdzie uznawana za niewa¿n¹.  
  
7. Obowi¹zuj¹ce ustawodawstwo i jurysdykcja  
  
Obowi¹zuj¹ce ustawodawstwo  
  
Obie Strony przyjmuj¹ jako obowi¹zuj¹ce prawo kraju, w
którym licencja na Program zosta³a uzyskana, oraz wyra¿aj¹ zgodê
na stosowanie takiego prawa przy interpretowaniu oraz
egzekwowaniu wszelkich praw i obowi¹zków Stron, wynikaj¹cych z
niniejszej Umowy lub maj¹cych w jakikolwiek sposób z ni¹ zwi¹zek, bez
odwo³ywania siê do norm kolizyjnych dotycz¹cych przepisów prawnych.   
  
Konwencja ONZ dotycz¹ca umów o Miêdzynarodowej Sprzeda¿y
Towarów (United Nations Convention on Contracts for the
International Sale of Goods) nie ma zastosowania.  
  
Jurysdykcja  
  
Wszelkie prawa, obowi¹zki i zobowi¹zania IBM podlegaj¹
w³aciwym s¹dom kraju uzyskania licencji na Program.  
  
Czêæ 2 - Warunki specyficzne dla poszczególnych krajów  
  
EUROPA, BLISKI WSCHÓD, AFRYKA (EUROPE, MIDDLE EAST, AFRICA -
EMEA)  
  
Prawa do danych (paragraf 3): W krajach Europy, Bliskiego
Wschodu i Afryki (EMEA) nastêpuj¹cy tekst zastêpuje w ca³oci
warunki zawarte w paragrafie 3:   
  
U¿ytkownik przenosi na IBM wszelkie prawa (w tym prawa
autorskie), tytu³y i korzyci odnosz¹ce siê do wszelkich danych,
propozycji i materia³ów w formie pisemnej 1) zwi¹zanych z u¿ywaniem
Programu przez U¿ytkownika oraz 2) dostarczonych przez U¿ytkownika
do IBM. Takie przeniesienie praw obejmuje w szczególnoci
przeniesienie praw do wykonania i zlecenia wykonania prac pochodnych z
materia³ów w formie pisemnej oraz na u¿ywanie, umo¿liwienie u¿ywania,
wykonanie, odtwarzanie, przesy³anie, wywietlanie, przenoszenie,
dystrybuowanie i licencjonowanie materia³ów w formie pisemnej oraz takich
prac pochodnych, znajduj¹cych siê na dowolnych nonikach lub
rozpowszechnianych za porednictwem dowolnej technologii dystrybucyjnej. Takie
przeniesienie obejmuje tak¿e przeniesienie praw do udzielania innym
osobom (fizycznym lub prawnym) niektórych lub wszelkich praw
przyznanych w niniejszym dokumencie na czas obowi¹zywania praw do
korzystania z wszelkich takich praw, tytu³ów i korzyci. Na ¿¹danie IBM
U¿ytkownik podpisze odpowiednie dokumenty, aby dokonaæ przeniesienia
takich praw. U¿ytkownik udziela IBM niewy³¹cznej, nieodwo³alnej,
nieograniczonej, ogólnowiatowej i w pe³ni op³aconej licencji na do³¹czenie
do dowolnego produktu lub us³ugi wszelkich pomys³ów,
znajomoci rzeczy (know-how), koncepcji, technik, wynalazków, odkryæ
czy udoskonaleñ, objêtych ochron¹ patentow¹ lub nie, maj¹cych
zwi¹zek z Programem i opracowanych przez U¿ytkownika lub jego
pracownika w okresie próbnego u¿ywania. Powy¿sza licencja obejmuje
równie¿ u¿ywanie, produkowanie i sprzeda¿ wszelkich takich
produktów i us³ug oraz zezwala innym na wykonywanie powy¿szych
czynnoci. ¯adna ze Stron nie bêdzie obci¹¿aæ drugiej Strony z tytu³u
praw do danych ani wykonanych prac, bêd¹cych wynikiem niniejszej
Umowy.   
  
Brak gwarancji (paragraf 4): W krajach Unii Europejskiej
poni¿sze zdanie zostaje dodane na pocz¹tku tego paragrafu:  
  
W krajach Unii Europejskiej konsumentom przys³uguj¹ prawa
wynikaj¹ce z obowi¹zuj¹cych krajowych aktów prawnych reguluj¹cych
zasady sprzeda¿y artyku³ów konsumpcyjnych. Postanowienia paragrafu
4 pozostaj¹ bez wp³ywu na postanowienia tych¿e aktów.  
  
Obowi¹zuj¹ce ustawodawstwo, jurysdykcja (paragraf 7)  
  
Obowi¹zuj¹ce ustawodawstwo  
  
Sformu³owanie "prawo kraju, w którym licencja na Program
zosta³a uzyskana" zostaje zast¹pione sformu³owaniem:  
  
"prawo Austrii". Zasada ta dotyczy Polski.  
  
Nastêpuj¹cy tekst zostaje dodany do tego paragrafu:  
  
Arbitra¿  
  
W Polsce wszelkie kwestie sporne wynikaj¹ce z niniejszej
Umowy lub zwi¹zane z jej naruszeniem, wypowiedzeniem lub
niewa¿noci¹ bêd¹ rozstrzygane w sposób ostateczny zgodnie z Zasadami
Postêpowania Arbitra¿owego i Ugodowego Miêdzynarodowego Centrum
Arbitra¿u przy Federalnej Izbie Gospodarczej (Rules of Arbitration
and Conciliation of the International Arbitral Center of the
Austrian Federal Economic Chamber) w Wiedniu (Vienna Rules), przez
trzech arbitrów, powo³anych zgodnie ze wspomnianymi zasadami.
Arbitra¿ bêdzie mia³ miejsce w Wiedniu, w Austrii. Jêzykiem
obowi¹zuj¹cym podczas postêpowania bêdzie jêzyk angielski. Orzeczenia
arbitrów bêd¹ ostateczne i wi¹¿¹ce dla obu Stron. Oznacza to, ¿e
zgodnie z artyku³em 598(2) Austriackiego Kodeksu Postêpowania
Cywilnego (Austrian Code of Civil Procedure), Strony rezygnuj¹ ze
stosowania artyku³u 595(1) punkt 7 tego Kodeksu. Tym niemniej
powy¿sze warunki w ¿adnym stopniu nie ograniczaj¹ prawa IBM do
wszczêcia postêpowania przed dowolnym w³aciwym s¹dem w kraju
instalacji.   
  
Z125-5544-03 (10/2005)  
INFORMACJE LICENCYJNE  
  
Na Programy wyszczególnione poni¿ej udzielane s¹ licencje
na warunkach, stanowi¹cych uzupe³nienie warunków, które
zawiera Miêdzynarodowa Umowa Licencyjna na Wstêpne Wydanie
Programów.  
  
Nazwa Programu: alphaWorks Emerging Technology  
Numer Programu: N/A  
  
Okrelone rodowisko Pracy  
  
Specyfikacje Programu oraz informacje dotycz¹ce okrelonego
rodowiska pracy mog¹ znajdowaæ siê w dokumentacji za³¹czonej do
Programu, jeli takowa istnieje, na przyk³ad w postaci pliku readme
lub w formie innej informacji og³oszonej przez IBM, na przyk³ad
w dokumencie announcement letter.  
  
Okres próbnego u¿ywania  
  
Okres próbnego u¿ywania zaczyna siê z dat¹ zaakceptowania
przez U¿ytkownika warunków niniejszej Umowy i koñczy siê po
up³ywie 90 dni.  
  
D/N: L-JLCO-6HQ6QK  
P/N: L-JLCO-6HQ6QK   
