# Supplementary material for: Finding New Order in Biological Functions from the Network Structure of Gene Annotations
Source: PLoS Comput Biol. 2015 Nov 20;11(11):e1004565. doi: 10.1371/journal.pcbi.1004565 (PMC4654495; doi:10.1371/journal.pcbi.1004565)
Supplement: S1 Code — This file contains the input human annotation files and all the code needed to reproduce the analyses and figures presented in this manuscript. The complete collection of intermediate files (such as the predicted term-term networks, word clouds for all communities, etc), can be obtained from [34]. (TGZ) [file pcbi.1004565.s004.tgz › TermCommunities_code/MakeCloudFiles/IBM Word Cloud/license/pt.html]

Software License

Acordo Internacional de Licenciamento para Programas "Early
Release"  
  
Parte 1 - Termos Gerais  
  
ESTE CONTRATO DE LICENÇA INTERNACIONAL PARA
DISPONIBILIZAÇÃO ANTECIPADA DE PROGRAMAS ("Early Release"), DORAVANTE
DESIGANDO "ACORDO", É UM ACORDO LEGAL ENTRE O CLIENTE E A IBM. AO
FAZER DOWNLOAD, INSTALAR, COPIAR, ACESSAR/ACEDER OU UTILIZAR O
PROGRAMA, O CLIENTE CONCORDA COM OS TERMOS DESTE ACORDO. SE O CLIENTE
ACEITAR OS TERMOS DESTE ACORDO EM NOME DE OUTRA PESSOA, OU UMA
EMPRESA OU OUTRA ENTIDADE LEGAL, O CLIENTE DECLARA E GARANTE QUE
POSSUI AUTORIDADE TOTAL PARA VINCULAR TAL PESSOA, EMPRESA OU
ENTIDADE LEGAL A ESTES TERMOS.  
  
"Disponibilização Antecipada (Early Release)" é uma release
de um Programa que (1) ainda pode estar em desenvolvimento (e
portanto, é potencialmente instável) ou (2) pode já não estar em
desenvolvimento mas ainda não foi tornado comercialmente disponível para os
usuários/utilizadores.  
  
"IBM" é a International Business Machines Corporation ou
uma das suas subsidiárias.  
  
"Informação sobre Licenciamento" ("LI") é um documento que
fornece informação e termos específicos sobre um Programa. A LI do
Programa está disponível em um arquivo/ficheiro no
diretório/directório do Programa, através da utilização de um comando do sistema
ou como um booklet que acompanha o Programa.  
  
"Programa" é um ou mais dos seguintes, incluindo o original
e todas as cópias totais ou parciais do mesmo: 1) instruções
e dados legíveis por máquina, 2) componentes de software
legíveis por humanos, 3) conteúdo audiovisual (tais como imagens,
textos, gravações ou figuras), 4) materiais licenciados
relacionados, 5) documentos ou chaves de utilização da licença, 6)
documentação associada e (7) quaisquer aprimoramentos/aperfeiçoamentos,
atualizações/actualizações ou materiais que a IBM pode escolher, a seu próprio
critério, para fornecer ao Cliente como Suporte (conforme descrito
abaixo).   
  
"Cliente" e "do Cliente" refere-se a uma pessoa individual
ou a uma única entidade legal.  
  
Este Acordo inclui Parte 1 - Termos Gerais, Parte 2 -
Termos Exclusivos do País (se houver) e Informações sobre
Licenciamento e é o acordo integral entre o Cliente e a IBM no que diz
respeito à utilização do Programa. Este Acordo substitui qualquer
comunicação anterior oral ou escrita entre o Cliente e a IBM relativa à
utilização do Programa. Os termos da Parte 2 e as Informações sobre
Licenciamento podem substituir ou modificar os termos da Parte 1.  
  
1. Licença  
  
O Programa é de propriedade da IBM ou de um fornecedor da
IBM, estando protegido por direitos autorais/de autor
(copyright) sob licença e não é vendido.  
  
A IBM concede ao Cliente uma licença limitada, não-
exclusiva, não-transferível para fazer download, instalar e utilizar o
Programa durante o período de avaliação, exclusivamente para
propósitos/fins de teste e avaliação internos e para fornecer feedback para
a IBM.  
  
O Cliente pode fazer uma cópia de backup do Programa para
suportar tal utilização. O Cliente não está autorizado a utilizar o
Programa para propósitos/fins produtivos ou para distribuir o
Programa ou qualquer uma de suas partes. O Cliente não pode
modificar ou criar trabalhos derivados do Programa. Os termos desta
licença aplicam-se à cada cópia que o Cliente possa fazer. O
Cliente deve reproduzir todos os avisos de direitos autorais/de
autor (copyright) e todas as outras legendas de propriedade em
cada cópia total ou parcial do Programa.  
  
O Cliente irá 1) manter um registro/registo de quaisquer
cópias do Programa e 2) garantir que qualquer pessoa que utilizar
o Programa (por acesso local ou remoto) o fará apenas para a
utilização autorizada do Cliente e de acordo com os termos deste
Acordo.  
  
O Cliente não pode: 1) utilizar, copiar, modificar,
transferir ou distribuir o Programa, salvo como previsto neste Acordo;
2) inverter a montagem, a compilação ou por qualquer outro
modo, converter o Programa em formato legível por humanos ou em
outra linguagem de programação (salvo se expressamente permitido
por lei, sem a possibilidade de renúncia contratual); 3)
sublicenciar, alugar ou arrendar o Programa; ou 4) utilizar o Programa em
uma base de bureau de serviços.  
  
Esta licença não autoriza o Cliente a receber da IBM
documentação em papel, suporte, assistência telefônica ou
aprimoramentos/aperfeiçoamentos ou atualizações/actualizações do Programa
(coletivamente/colectivamente designado por "Suporte") se bem que a IBM, reserva-se o
direito de poder determinar em fornecer tal Suporte. Quaisquer
aprimoramentos/aperfeiçoamentos, atualizações/actualizações e outros materiais fornecidos
pela IBM como parte do Suporte são considerados como parte do
Programa e por conseguinte regulados por este Acordo.  
  
O PROGRAMA PODE CONTER UM DISPOSITIVO
DESATIVADOR/DESACTIVADOR QUE O IMPEDE DE SER UTILIZADO APÓS O TERMO DO PERÍODO DE
AVALIAÇÃO. O CLIENTE NÃO PODE VIOLAR ESTE DISPOSITIVO DE
DESATIVAÇÃO/DESACTIVAÇÃO OU O PROGRAMA. O CLIENTE DEVERÁ TOMAR PRECAUÇÕES DE MODO A
EVITAR QUALQUER PERDA DE DADOS QUE POSSA OCORRER QUANDO O PROGRAMA
NÃO PUDER MAIS SER UTILIZADO.  
  
2. Duração  
  
O período de avaliação começa quando o Cliente aceita os
termos deste Acordo e termina mediante 1) a data de encerramento
(se houver) especificada nas Informações de Licenciamento, 2) a
data na qual o Programa se desativa/desaciva automaticamente ou
3) a data na qual a IBM torna o programa comercialmente
disponível. A licença do Cliente para o Programa termina no final do
período da avaliação e o Cliente destruirá o Programa e todas as
cópias existentes dentro de 10 dias a contar do final do período
de avaliação.  
  
Não há encargos para a utilização do Programa durante o
período de avaliação.  
  
A IBM pode cancelar a licença do Cliente se este não
cumprir com os termos deste Acordo. Se a IBM assim o fizer, o
Cliente deve destruir todas as cópias do Programa.  
  
3. Direitos dos Dados  
  
O Cliente cede à IBM todos os direitos, titularidade e
benefícios, incluindo propriedade de direitos autorais/de autor
(copyright) sobre quaisquer dados, sugestões ou materiais escritos que
1) estejam relacionados com o Programa e 2) o Cliente forneça
à IBM. Se a IBM solicitar, o Cliente assinará um documento
apropriado para ceder tais direitos. Até ao limite de outro modo não
coberto pela concessão do Cliente sob a/ao abrigo da primeira frase
desta Seção/Secção 3, no respeitante a qualquer idéia/ideia, know-
how, conceito, técnica, invenção, descoberta ou
aprimoramento/aperfeiçoamento, seja ou não patenteável, com o Programa e que o Cliente
forneça à IBM, o Cliente concede à IBM o direito e a licença não-
exclusivos, irrevogáveis, não-restritos, mundiais e pagos para incluir
o anterior em qualquer produto ou serviço e para utilizar,
fabricar ou comercializar qualquer produto ou serviço e para
permitir que outros façam qualquer uma das ações/acções anteriores.  
  
4. Sem Garantia  
  
SUJEITO ÀS GARANTIA LEGAIS, SE HOUVER, QUE NÃO PODEM SER
EXCLUÍDAS, A IBM NÃO CONCEDE QUAISQUER GARANTIAS OU CONDIÇÕES DE
NENHUM TIPO, SEJAM EXPRESSAS OU IMPLÍCITAS, INCLUINDO MAS NÃO SE
LIMITANDO ÀS GARANTIAS OU CONDIÇÕES IMPLÍCITAS DE QUALIDADE
SATISFATÓRIA, COMERCIALIZAÇÃO, ADEQUAÇÃO A UM DETERMINADO PROPÓSITO/FIM E
GARANTIA DE TITULARIDADE E DE NÃO-VIOLAÇÃO RELATIVAS AO PROGRAMA OU
SUPORTE TÉCNICO, SE HOUVER.  
  
A exclusão aplica-se, também, a quaisquer entidades que
desenvolvam Programas da IBM ou fornecedores.  
  
Fabricantes, fornecedores ou editores de Programas não-IBM
podem fornecer as suas próprias garantias.  
  
5. Limitação de Responsabilidade  
  
Podem surgir circunstâncias em que, devido ao não
cumprimento/incumprimento das suas responsabilidades pela IBM, o Cliente tenha
direito de exigir que a IBM o compense por danos sofridos.
Independente da base sobre a qual o Cliente possa estar autorizado a
reclamar danos da IBM (incluindo violação fundamental, negligência,
falsas declarações ou outra reclamação contratual ou
extracontratual), a IBM é responsável por não mais do que 1) danos corporais
(incluindo os que provoquem morte) e danos em bens imóveis e móveis e
2) o valor de qualquer outro dano direto/directo real até um
limite de 25.000 dólares americanos (ou o equivalente em moeda
local) para todas as reclamações em agregado. Esta limitação de
responsabilidade também se aplica às entidades que desenvolvem Programas IBM
e fornecedores. Este é o limite máximo pelo qual a IBM, seus
fornecedores ou entidades que desenvolvem Programas são
coletivamente/colectivamente responsáveis.  
  
EM CASO ALGUM A IBM, ENTIDADES QUE DESENVOLVEM PROGRAMAS
IBM OU FORNECEDORES SERÃO RESPONSABILIZADOS PELO SEGUINTE,
MESMO SE INFORMADOS DE SUA POSSIBILIDADE:  
  
1. PERDA OU DANOS EM DADOS;  
2. DANOS ESPECIAIS, ACIDENTAIS, INDIRETOS/INDIRECTOS,
EXEMPLARES OU PUNITIVOS, OU PARA QUAISQUER DANOS ECONÔMICOS
CONSEQÜENCIAIS; OU  
3. LUCROS CESSANTES, NEGÓCIOS, RECEITA, CLIENTELA OU LUCROS
ANTECIPADOS.  
  
6. Geral  
  
1. Nada neste Acordo afeta/afecta quaisquer direitos legais
dos consumidores que não sejam passíveis de renúncia ou
limitação contratual.  
2. No caso de alguma disposição deste Acordo vir a ser
considerada inválida ou ineficaz, as restantes disposições do Acordo
permanecem em vigor.  
3. O Cliente não pode exportar o Programa ou executar
qualquer ação/acção com relação ao Programa que viole as leis de
controle de exportação aplicáveis.  
4. O Cliente concorda em autorizar que a International
Business Machines Corporation e as suas subsidiárias armazenem e
utilizem as informações de contato/contacto comercial do Cliente,
incluindo nomes, números de telefone comerciais e endereços de e-mail
comerciais, onde quer que a IBM tenha negócios. Estas informações serão
processadas e utilizadas no âmbito do relacionamento comercial entre
ambas as partes e poderão ser facultadas a subcontratados que
atuam/actuam em nome da IBM, Parceiros de Negócio IBM que promovam,
comercializem e fornceçam suporte a determinados produtos e serviços IBM
e cessionários da International Business Machines Corporation
e suas subsidiárias para fins consentâneos com as respectivas
atividades/actividades comerciais.  
5. A IBM não garante que qualquer versão do Programa que
seja formalmente liberada/aprovada ou comercialmente
disponibilizada (se houver), será semelhante ou compatível com as versões
de Disponibilização Antecipada (Early Release).  
6. Nem o Cliente nem a IBM poderão iniciar uma ação/acção
legal relacionada com este Acordo decorridos mais de dois anos
depois de ter ocorrido a causa da acção, a não ser que seja
estabelecido de outra forma pela lei local sem a possibilidade de
renúncia ou limitação contratual.  
7. Nem o Cliente nem a IBM serão responsabilizados pelo não
cumprimento das obrigações devido a causas fora de seu controle.  
8. Este Acordo não criará nenhum direito ou forma de
ação/acção a qualquer terceiro, nem a IBM será responsável por
quaisquer reclamações de terceiros contra o Cliente, salvo, como
permitido pela Limitação de Responsabilidade na seção/secção acima,
por danos corporais (incluindo os que provoquem morte) ou danos
em bens imóveis e móveis sobre as quais a IBM é legalmente
responsável.  
9. O Cliente não pode ceder este Contrato, total ou
parcialmente, sem a autorização prévia e por escrito da IBM. Qualquer
tentativa nesse sentido será nula.  
  
7. Lei Aplicável e Jurisdição  
  
Lei Aplicável  
  
O Cliente e a IBM concordam com a aplicação das leis do
país no qual o Cliente obteve a licença do Programa para reger,
interpretar e fazer cumprir todos os direitos, deveres e obrigações do
Cliente e da IBM emergentes, ou por alguma maneira relacionados,
com este Acordo, sem prejuízo de qualquer conflito com os
princípios de direito.  
  
A Convenção das Nações Unidas não se aplica nos Acordos
para Venda Internacional de Bens.  
  
Jurisdição  
  
Todos os direitos, deveres e obrigações do Cliente e da IBM
estão sujeitos aos tribunais do país em que o Cliente obteve a
licença do Programa.  
  
Parte 2 - Termos Exclusivos do País  
  
AMÉRICAS  
BRASIL: Lei Aplicável e Jurisdição (Seção/Secção 7): A
exceção a seguir é incluída nesta seção/secção:  
  
Qualquer litígio emergente deste Acordo será dirimido
exclusivamente pelo tribunal do Rio de Janeiro, RJ.  
  
EUROPA, ORIENTE MÉDIO, ÁFRICA (EMEA)  
  
Direitos dos Dados (Seção/Secção 3): Na EMEA, o seguinte
substitui os termos desta seção/secção na sua totalidade:  
  
O Cliente cede à IBM todos os direitos, titularidade e
benefícios, incluindo propriedade de direitos autorais/de autor
(copyright) sobre quaisquer dados, sugestões e materiais escritos que
1) estejam relacionados com a utilização do Programa pelo
Cliente e 2) sejam fornecidos pelo Cliente à IBM. Tal cessão de
direitos inclui, mas não se limita à, cessão de direitos para
preparar e por ter preparado trabalhos derivados dos materiais
escritos e para utilizar, por ter utilizado, executar, reproduzir,
transmitir, exibir, desempenhar, transferir, distribuir e licenciar os
materiais escritos e tais trabalhos derivados por qualquer meio ou
tecnologia de distribuição, e para conceder a terceiros alguns ou
todos os direitos aqui concedidos, pela duração de tais direitos
e titularidades. Se a IBM solicitar, o Cliente assinará um
documento apropriado para ceder tais direitos. No respeitante a
qualquer idéia/ideia, know-how, conceito, técnica, invenção,
descoberta ou aprimoramento/aperfeiçoamento, seja ou não patenteável,
relacionados com o Programa e criados pelo Cliente ou seus funcionários
durante o período de avaliação, o Cliente concede à IBM um direito
e uma licença não-exclusivos, irrevogáveis, não-restritos,
mundiais e pagos para incluir o anterior em qualquer produto ou
serviço e para utilizar, fabricar ou comercializar qualquer produto
ou serviço e para permitir que outros façam qualquer uma das
ações/acções anteriores. Nenhuma parte cobrará à outra por direitos
sobre dados ou qualquer trabalho executado no âmbito deste Acordo.  
  
Sem Garantia (Seção/Secção 4): Na União Européia/Europeia,
o seguinte é incluído no início desta seção/secção:  
  
Na União Européia/Europeia, os consumidores possuem
direitos legais ao abrigo da legislação nacional aplicável que
regula a venda de bens de consumo. Tais direitos não são
afetados/afectados pelas disposições desta Seção/Secção 4.  
  
Limitação de Responsabilidade (Seção/Secção 5): Em
Portugal, o seguinte substitui os termos desta seção/secção na sua
totalidade:  
  
Salvo disposição legal imperativa em contrário:  
  
1. A responsabilidade da IBM por danos e perdas que possam
surgir como conseqüência/consequência do cumprimento das suas
obrigações ou devido a qualquer outro motivo relacionado com este
Acordo, encontra-se limitada à compensação apenas pelos danos e
perdas provados e que surjam de fato/facto como
conseqüência/consequência direta/directa e imediata do não cumprimento das referidas
obrigações (se a IBM estiver em falta) ou nesse caso por um valor
máximo que não exceda, em nenhuma hipótese, a ?25.000.  
A limitação acima, não se aplica a danos corporais
(incluindo os que provoquem morte), e a danos em bens imóveis e bens
móveis pelos quais a IBM é legalmente responsável.  
2. EM CIRCUNSTÂNCIA ALGUMA A IBM, ENTIDADES QUE DESENVOLVAM
PROGRAMAS OU FORNECEDORES SERÃO RESPONSÁVEIS PELO SEGUINTE, MESMO QUE
TENHAM SIDO INFORMADOS DESSA POSSIBILIDADE: 1) PERDA OU DANOS EM
DADOS; 2) DANOS ESPECIAIS, INCIDENTAIS OU INDIRETOS/INDIRECTOS, OU
PUNITIVOS OU POR DANOS DE CONSEQÜÊNCIA ECONÔMICA/ECONÓMICA; 3) LUCROS
CESSANTES, MESMO QUE ESTES SEJAM CONSEQÜÊNCIA/CONSEQUENCIA IMEDIATA DO
EVENTO QUE GEROU TAIS DANOS; OU 4) PERDA DE NEGÓCIOS, RECEITAS,
CLIENTELA OU POUPANÇAS PREVISTAS.  
3. A limitação e a exclusão de responsabilidade
estabelecidas aqui aplicam-se não só às atividades/actividades executadas
pela IBM mas também às atividades/actividades executadas pelos
seus fornecedores e entidades que desenvolvam Programas e
representam o valor máximo pelo qual a IBM, seus fornecedores e
entidades que desenvolvam Programas são coletivamente/colectivamente
responsáveis.  
  
Lei Aplicável e Jurisdição (Seção/Secção 7)  
  
Jurisdição  
  
A seguinte exceção é incluída nesta seção/secção:  
  
Em Portugal qualquer ação/acção judicial decorrente deste
Acordo será instaurada e resolvida exclusivamente pelo tribunal
competente de Lisboa.  
  
Z125-5544-03 (10/2005)  
INFORMAÇÕES SOBRE LICENCIAMENTO  
  
Os Programas abaixo indicados estão licenciados sob/ao
abrigo dos seguintes termos e condições além daqueles constantes
do Acordo Internacional de Licenciamento para Programas "Early
Release".  
  
Nome do Programa: alphaWorks Emerging Technology  
Número do Programa: N/A  
  
Ambiente Operacional/Operativo Especificado  
  
As especificações do programa e a informação especificada
do ambiente operacional/operativo podem ser encontradas na
documentação que acompanha o programa, se disponíveis, como um arquivo
"readme", ou outra informação publicada pela IBM, tal como uma carta
de anúncio.  
  
Período de Avaliação  
  
O período de avaliação inicia-se na data em que o Cliente
aceita os termos deste Acordo e termina após 90 dias.  
  
D/N: L-JLCO-6HQ6QK  
P/N: L-JLCO-6HQ6QK   
