# Supplementary material for: Finding New Order in Biological Functions from the Network Structure of Gene Annotations
Source: PLoS Comput Biol. 2015 Nov 20;11(11):e1004565. doi: 10.1371/journal.pcbi.1004565 (PMC4654495; doi:10.1371/journal.pcbi.1004565)
Supplement: S1 Code — This file contains the input human annotation files and all the code needed to reproduce the analyses and figures presented in this manuscript. The complete collection of intermediate files (such as the predicted term-term networks, word clouds for all communities, etc), can be obtained from [34]. (TGZ) [file pcbi.1004565.s004.tgz › TermCommunities_code/MakeCloudFiles/IBM Word Cloud/license/tc.html]

Software License

°ê»Ú±ÂÅv¦X¬ù - ´ú¸Õª©  
  
²Ä¤@³¡¤À - ¤@¯ë±ø´Ú  
  
¥»°ê»Ú±ÂÅv¦X¬ù - ´ú¸Õª©¡]¡u¥»¦X¬ù¡v¡^«Y ¶Q«È¤á»P IBM ©Ò­q¦Xªk¦X¬ù¡C ¶Q«È¤á¤@¥¹¤U¸ü¡B¦w¸Ë¡B½Æ»s¡B¦s¨ú©Î¨Ï¥Î
¥»¡uµ{¦¡¡v¡A§Yªí¥Ü ¶Q«È¤á¦P·N¥»¦X¬ù¤§±ø´Ú¡C­Y ¶Q«È¤á«Y¥Nªí¥L¤H¡B¨ä¥L¤½¥q©Î¨ä¥Lªk¤H¦P·N¦¹µ¥±ø´Ú¡A«h ¶Q«È¤á«OÃÒ
¶Q«È¤á¾Ö¦³¥R¤ÀÅv­­±o¥H¨Ï¸Ó¥L¤H¡B¸Ó¤½¥q©Î¸Óªk¤H¨ü¦¹µ¥±ø´Ú©ë§ô¡C  
  
¡u´ú¸Õª©¡v«Y«ü (1) ¤´¦b¶}µo¤¤¤§µ{¦¡ª©¥»¡]¦]¦¹¥i¯à¤£¥i¾a¡^¡F©Î (2) ¥i¯à¤w¤£¦b¶}µo¤¤¡A¦ý¥¼¥H°Ó«~¤Æ¤è¦¡´£¨Ñ¤©¨Ï¥Î
ªÌ¡C  
  
"IBM" «Y«ü International Business Machines Corporation ©Î¨ä¤l¤½¥q¡C  
  
¡u±ÂÅv¤â¥U¡v ("LI") «Y«ü¤º§t¬Yµ{¦¡¯S©w¸ê°T¤Î±ø´Ú¤§¤å¥ó¡C¥»µ{¦¡¤§±ÂÅv¤â¥U¦ì©ó¥»µ{¦¡¬Y¥Ø¿ý¤§ÀÉ®×¤º¡A¥H¨Ï¥Î¨t²Î«ü¥O
¤è¦¡«K¥i§ä¨ì¡A¦¹¥~¡A¥ç¥i¯à¥H¤p¥U¤l§Î¦¡ÀËªþ©ó¥»µ{¦¡¡C  
  
¡uµ{¦¡¡v«Y«üµ{¦¡­ìª©¤Î¨ä¥þ³¡©Î³¡¤À½Æ¥»¡A¥]¬A¡G1) ¾÷¾¹¥i¾\Åª«ü¥O¤Î¸ê®Æ¡F2) ¥i¤H¤u¾\Åª³nÅé¤¸¥ó¡F3) Án/¼v³¡¤À¡]¦p
¹Ï¹³¡B¤å½Z¡B¿ý­µ¡B©Î·Ó¤ùµ¥¡^¡F4) ¬ÛÃö±ÂÅvµÛ§@ª«¡F 5) ±ÂÅv¨Ï¥Î¤å¥ó©Î±ÂÅv½X¡F6) ¬ÛÃö»¡©ú¤å¥ó¡F¤Î 7) IBM ±o
¦Û¦æ¿ï¾Ü´£¨Ñ¤© ¶Q«È¤á¥H§@¬°¤ä´©¤§¥[±j¥\¯à¡B§ó·s¸ê®Æ©ÎµÛ§@ª«¡]»¡©ú¦p¤U¡^¡C   
  
¡u¶Q«È¤á¡v«Y«ü³æ¤@­Ó¤H©Î³æ¤@ªk¤H¡C  
  
¥»¦X¬ù¥]¬A²Ä¤@³¡¤À - ¤@¯ë±ø´Ú¡B²Ä¤G³¡¤À - ¦U°ê±M¦³±ø´Ú¡]¦p¦³¦¹µ¥±ø´ÚªÌ¡^¤Î±ÂÅv¤â¥U¡A¥B¬° ¶Q«È¤á»P IBM ´N¥»
µ{¦¡¤§¨Ï¥Î©Ò­q§¹¾ã¦X¬ù¡C¥B¨ú¥N ¶Q«È¤á¥ý«e»P IBM ©Ò§@ªº¤@¤Á¤fÀY©Î®Ñ­±¨óÄ³¡C²Ä¤G³¡¤À±ø´Ú¤Î±ÂÅv¤â¥U±o¨ú¥N©Î­×­q²Ä¤@³¡
¤À¤§±ø´Ú¡C  
  
1. ±ÂÅv  
  
IBM ©Î IBM ¤§¨ÑÀ³°Ó¾Ö¦³¥»¡uµ{¦¡¡v¤§µÛ§@Åv¡A¥»¦X¬ù¬°±ÂÅv¦X¬ù¦Ó«DµÛ§@ÅvÅý°â¦X¬ù¡C  
  
IBM ±Â¤© ¶Q«È¤á¦³­­¡B«D±MÄÝ©Ê¡B¤£¥iÂàÅý¤§±ÂÅv¡A©ó¸Õ¥Î´Á¶¡¥i¤U¸ü¡B¦w¸Ë¤Î¨Ï¥Î¥»µ{¦¡¡A±©¶È±o±N¨ä¥Î©ó¤º³¡´ú¸Õ¤Îµû¦ô¥Î
³~¡F¨Ã´£¨Ñ·N¨£¤© IBM¡C  
  
¶Q«È¤á±o»s§@¥»µ{¦¡³Æ¥÷¡A¥H¤ä´©¸Ó¨Ï¥Î¦æ¬°¡C ¶Q«È¤á¤£±o±N¥»µ{¦¡¥Î©ó¥Í²£¥Î³~¡A¥ç¤£±o´²¥¬¥»µ{¦¡©Î¨ä¥ô¦ó³¡¤À¡C ¶Q«È¤á¤£
±o­×§ï©Î«Ø¥ß¥»µ{¦¡¤§­l¥ÍµÛ§@¡C¥»±ÂÅv¤§±ø´Ú¾A¥Î©ó ¶Q«È¤á©Ò»s§@¤§¨C¤@¥÷½Æ¥»¡C ¶Q«È¤á½Æ»s¥»µ{¦¡®É¡A¤£½×¨ä«Y¥þ³¡©Î³¡¤À½Æ
¥»¡A§¡¶·©ó¸Ó½Æ¥»¤W½Æ»s¥»µ{¦¡¤§µÛ§@Åv¤Î¨ä¥L¦³Ãö¤§Åv§Q¼Ð¥Ü¡C  
  
¶Q«È¤áÀ³ 1) «O¦s¥»µ{¦¡¥ô¦ó½Æ¥»¤§°O¿ý¡F2) ½T«O¥ô¦ó¤H©ó¨Ï¥Î¥»µ{¦¡®É¡]¤£½×±Ä¥»ºÝ©Î»·ºÝ¤è¦¡¦s¨ú¡^¡A§¡¯à¿í¦u¥»¦X¬ù±ø´Ú
¤§³W©w¡C  
  
°£¥»¦X¬ù¥t¦³³W©w¥~¡A ¶Q«È¤á¤£±o¡G(1) ¨Ï¥Î¡B½Æ»s¡B­×§ï¡BÂàÅý©Î´²¥¬¥»µ{¦¡¡F (2) °f¦V²Õ¦X¡B°f¦V½sÄ¡¥»µ{¦¡¡A©Î±N
¨äÂà´«¬°¥i¤H¤u¾\Åª®æ¦¡©Î¨ä¥Lµ{¦¡»y¨¥¡]±©ªk«ß©ú¤å³W©w¤£±o¥H«´¬ù©ß±óªÌ¡A¤£¦b¦¹­­¡^¡F(3) Âà±ÂÅv©Î¥X¯²¥»µ{¦¡¡F©Î 4) ¥H
´£¨ÑªA°È¤§¾÷ºc¬°°òÂ¦¨Ï¥Î¥»µ{¦¡¡C  
  
¥»±ÂÅv¦X¬ù¥¼±Â¤© ¶Q«È¤á¤U¦CÅv§Q¡G¦¬¨ü IBM ´£¨Ñ¤§¦L¨ê¥»»¡©ú¤å¥ó¡B¤ä´©¡B¹q¸Ü¨ó§U©Î¥»µ{¦¡¤§¥[±j¥\¯à©Î§ó·s¸ê®Æ¡]²ÎºÙ
¡u¤ä´©¡v¡^¡AÁöµM IBM ±o¦Û¦æ¨M©w´£¨Ñ¸Ó¶µ¤ä´©¡A¥ç¦P¡C¥Ñ IBM ´£¨Ñ¡A§@¬°¤ä´©¤§¤@³¡¤À¤§¥ô¦ó¥[±j¥\¯à¡B§ó·s¸ê®Æ¤Î¨ä¥LµÛ
§@ª«¡Aµø¦P¥»µ{¦¡¤§¤@³¡¤À¡A¬G¨ü¥»¦X¬ù©ë§ô¡C  
  
¥»µ{¦¡¥i¯à¥]§t¤@­Ó°±¥Î¸Ë¸m¡A¥H¨¾¤î¸Õ¥Î´Áº¡«á¤§¨Ï¥Î¡C ¶Q«È¤á¤£±o¾Õ§ï¦¹°±¥Î¸Ë¸m©Î¥»¡uµ{¦¡¡v¡C·í¥»µ{¦¡¤£¯à¨Ï¥Î«e¡A ¶Q
«È¤áÀ³±Ä¨ú¹w¨¾±¹¬I¥HÁ×§K¦]¤£¦A¯à¨Ï¥Î¥»µ{¦¡¦Ó¥i¯à³y¦¨¤§¸ê®Æ·À¥¢¡C   
  
2. ±ø´Ú  
  
¸Õ¥Î´Á¦Û ¶Q«È¤á¦P·N¥»¦X¬ù±ø´Ú¤§·í¤é°\_¥Í®Ä¡A¨Ã¥H¤U¦C¤é´Á¤¤¸û¦­ªÌ¬°¨ä²×¤î¤é¡G1) ±ÂÅv¤â¥U¤¤«ü©w¤§²×¤î¤é¡]­Y¦³¤§¡^
¡F2) ¥»µ{¦¡¥»¨­¦Û°Ê°±¥Î¤§·í¤é¡F©Î 3) IBM ±N¥»µ{¦¡°Ó«~¤Æ¤§·í¤é¡C ¶Q«È¤á©Ò±o¥»µ{¦¡±ÂÅv©ó¸Õ¥Î´Áµ²§ô®É²×¤î¡A ¶Q«È¤á
À³©ó¸Õ¥Î´Á²×¤î«á¤Q¤Ñ¤º¾P·´¥»µ{¦¡¤Î¨ä¤@¤Á½Æ¥»¡C  
  
¶Q«È¤á©ó¸Õ¥Î´Á¨Ï¥Î¥»µ{¦¡ªÌ¡AµL»Ý¤ä¥I¥ô¦ó¶O¥Î¡C  
  
­Y ¶Q«È¤á¹H¤Ï¥»¦X¬ù±ø´Ú¡AIBM ±o²×¤î¹ï ¶Q«È¤á¤§±ÂÅv¡C­Y IBM ²×¤î±ÂÅv¡A ¶Q«È¤á»Ý¾P·´¥»¡uµ{¦¡¡v¤§©Ò¦³½Æ
¥»¡C  
  
3. ¸ê®ÆÅv§Q  
  
¶Q«È¤á¦P·N±N 1) ¦³Ãö¥»µ{¦¡¤Î 2) ¶Q«È¤á´£¨Ñ¤© IBM ¤§¥ô¦ó¸ê®Æ¡B«ØÄ³©Î®Ñ­±µÛ§@ª«¤§¤@¤ÁÅv§Q¡B©Ò¦³Åv¤Î§Q¯q¡]¥]¬A
µÛ§@Åv¤§©Ò¦³Åv¡^ÂàÅý¤© IBM¡C­Y IBM »Ý­n¤å¥óÃÒ©ú¡A«h ¶Q«È¤áÀ³Ã±¸p¾A·í¤å¥ó¥HÂàÅý¦¹µ¥Åv§Q¡CÃö©ó¦³Ãö¥»µ{¦¡¤Î ¶Q
«È¤á´£¨Ñ¤© IBM ¤§¥ô¦óÆ[©À¡B§Þ¯à¡B·§©À¡B§Þ³N¡Bµo©ú¡Bµo²{©Î§ï¨}¡]¤£½×¬O§\_¬°Àò±o±M§QªÌ¡^¡A¦p¦³ ¶Q«È¤á©ó¥»²Ä 3 ¸`²Ä
¤@¥y¥¼²[»\¤§ ¶Q«È¤á±Â¤©½d³òªÌ¡A ¶Q«È¤á±Â¤© IBM «D±MÄÝ©Ê¡B¤£¥iºM¾P¡B¥¼­­¨î¤§¥þ²y©Ê¤w¥I´ÚÅv§Q¤Î±ÂÅv¡A¥H¥]§t¥ô¦ó²£
«~©ÎªA°È¤¤¤§«e­z¶µ¥Ø¡A¤Î¨Ï¥Î¡B»s³y¤Î¦æ¾P¥ô¦ó¸Ó²£«~©ÎªA°È¡A¨Ã³\¥i¥L¤H°õ¦æ«e­z¤§¥ô¦ó¶µ¥Ø¡C  
  
4. µL«OÃÒ  
  
°£ªk«ß³W©w¤£±o±Æ°£¤§«OÃÒ¥~¡AIBM ¤£ªþ¨ã¥ô¦ó©ú¥Ü©ÎÀq¥Ü¤§«OÃÒ¡A¥]¬A¥B¤£­­©ó«~½èº¡·N«×¡B¥i°Ó¥Î©Ê¡B²Å¦X¯S©w¥Î³~¤§Àq¥Ü«OÃÒ
©Î±ø¥ó¡A©Î¦³Ãö¥»µ{¦¡©Î§Þ³N¤ä´©¤§©Ò¦³Åv¤ÎµL«IÅv¦æ¬°¤§«OÃÒ¡C  
  
¸Ó¶µ±Æ°£¥ç¾A¥Î©ó¥ô¦ó IBM ¤§¡uµ{¦¡¡v¶}µoªÌ»P¨ÑÀ³°Ó¡C  
  
«D IBM ¤§¡uµ{¦¡¡v»s³y°Ó¡B¨ÑÀ³°Ó©Îµo¦æ¤H¥i¯à´£¨Ñ¨ä¥»¨­ªº«OÃÒ¨Æ¶µ¡C  
  
5. ½ßÀv¤W­­  
  
¦] IBM ¹H¬ù©Î¨ä¥L¥iÂk³d¨Æ¥Ñ¡A­P ¶Q«È¤á±o¦V IBM ´£¥X·l®`½ßÀv¤§½Ð¨D®É¡AµL½× ¶Q«È¤á°ò©ó¦óºØÅv§Q½Ð¨D½ßÀv¡]¥]
¬A­«¤j¹H¬ù¡B¹L¥¢¡B¤£¹ê³¯­z©Î¨ä¥L«´¬ù©Î«IÅv¦æ¬°¤§½Ð¨D¡^¡AIBM ¤§½ßÀv³d¥ô¶È­­©ó 1) ¤H¨­¶Ë®`¡]¥]¬A¦º¤`¡^¡B¤£°Ê²£©Î­Ó¤H
¦³§Î¸ê²£¤§·´·l 2) ¨ä¥Lª½±µ¹ê»Ú·l®`¡A±©²Ö­pÁ`¨DÀvª÷ÃB¥H¬üª÷ 25,000 ¤¸¡]©Î·í¦a³f¹ôµ¥­Èª÷ÃB¡^¬°¨ä¤W­­¡C¦¹½ßÀv¤W­­
¥ç¾A¥Î©ó IBM ¤§¡uµ{¦¡¡v¶}µoªÌ»P¨ÑÀ³°Ó¡C¦¹½ßÀv¤W­­«Y IBM ¤Î IBM ¤§¡uµ{¦¡¡v¶}µoªÌ»P¨ÑÀ³°Ó¤§¦@¦P½ßÀv³d¥ô¤W­­¡C  
  
¦b¥ô¦ó±¡ªp¤U¡AIBM¡BIBM ¤§µ{¦¡¶}µoªÌ©Î¨ÑÀ³°Ó¹ï¤U¦C±¡¨Æ§¡¤£­t½ßÀv³d¥ô¡A§Y¨Ï³Q§iª¾¸Ó±¡¨Æ¦³¥i¯àµo¥Í®É¡A¥ç¦P¡G  
  
1. ¸ê®Æ¤§·À¥¢©Î·´·l¡F  
2.¯S®í¡Bªþ±a¡B¶¡±µ¡BÃg§Ù©Ê¡BÃg»@©Ê·l®`©Î¨ä¥L­l¥Í©Ê¸gÀÙ·l®`¡F©Î  
3. §Q¼í¡BÀç·~¡BÀç¦¬¡B°ÓÅA©Î¹w´Áµ²¾lµ¥¶µ¤§·l¥¢¡C  
  
6. ¤@¯ë±ø´Ú  
  
1. ¦X¬ù¤£¼vÅT¥ô¦ó¤£±o¥H«´¬ù­­¨î©Î©ß±ó¤§ªk©w®ø¶OªÌÅv¯q¡C  
2. Áa¨Ï¥»¦X¬ù¤¤¦³¥ô¦ó±ø´Ú³Q»{©w¬°µL®Ä©ÎµLªk°õ¦æ¡A¥»¦X¬ù¤§¨ä¥L±ø´Ú¤´¨ã§¹¾ã¤§ªk«ß®Ä¤O¡C  
3. ¶Q«È¤á¤£±o¿é¥X¥»µ{¦¡¡A©Î´£¥X¹H¤Ï¾A¥Î¿é¥XºÞ¨îªk¤§¥ô¦ó¦³Ãö¥»µ{¦¡¤§¶D³^¡C  
4. ¶Q«È¤á¦P·N International Business Machines Corporation ¤Î¨ä¤l¤½¥q±o©ó
¶i¦æ·~°È¤§¥ô¦ó³B©Ò¹ï ¶Q«È¤á¤§°Ó·~Ápµ¸¸ê°T¦æ¨ÏÀx¦s¤Î¨Ï¥Î¤§¦æ¬°¡A¸Ó°Ó·~Ápµ¸¸ê°T¥]¬A©m¦W¡B°Ó¥Î¹q¸Ü¸¹½X¤Î°Ó¥Î¹q¤l¶l¥ó¦ì§}¡C
¸Ó¸ê°T±o´N¥»¤½¥q¤§¨Æ·~Ãö«Y¦Ó³B²z¤Î¨Ï¥Î¤§¡A¥ç±o±N¨ä´£¨Ñ¤©¥Nªí IBM ¤§©Ó¥]°Ó¡B­t³d«Å¶Ç¡B¦æ¾P¤Î¤ä´©¬Y¨Ç IBM ²£«~¤ÎªA
°È¤§ IBM ¨Æ·~¹Ù¦ñ¡A¥H¤Î International Business Machines Corporation ¤Î¨ä
¤l¤½¥q¤§¨üÅý¤H¡A¬°²Å¦X¸Ó¨Æ·~Ãö«Y¤§¨Ï¥Î¡C  
5. IBM ¤£«OÃÒ¤½¶}µo¦æ©Î°Ó«~¤Æ¤§¥ô¦óª©¥»¤§¥»µ{¦¡¡]­Y¦³¤§¡^¡A±N»P¥»´ú¸Õª©¥»¬Û¦ü©Î¬Û®e¡C  
6. ¶Q§ÚÂù¤è§¡¦P·N¡A©ó¥ô¤@¶D³^­ì¦]µo¥Í¨â¦~«á¡A¤£¦A´£°\_¥ô¦ó§Î¦¡¤§¶D³^¡A°£«D·í¦aªk«ß³W©w¤£±o¥H«´¬ù¤è¦¡©ñ±ó©Î­­¨î¡C  
7. ¶Q§ÚÂù¤è§¡µL»Ý¹ï¤£¥i§Ü¤O¦]¯À³y¦¨¤§·l®`­t³d¡C  
8. ¥»¦X¬ù¤£¬°¥ô¦ó²Ä¤T¤H³Ð³]¶D³^Åv©Î¶D³^­ì¦]¡A¦¹¥~¡A¥ô¦ó²Ä¤T¤H¦V ¶Q«È¤á´£¥X¤§¯Á½ß­n¨D¡AIBM ¥ç·§¤£­t³d¡A±©¥H¤W
¡u½ßÀv¤W­­¡v¤@¸`©Ò®e³\ªÌ¡]IBM ¨ÌªkÀ³­t³d¤§¤H¨­¶Ë®`¡]¥]¬A¦º¤`¡^©Î¤£°Ê²£¡B­Ó¤H¦³§Î¸ê²£¤§·´·l¡^¡A¤£¦b¦¹­­¡C  
9. ¥¼¸g IBM ¨Æ¥ý¥H®Ñ­±¦P·NªÌ¡A ¶Q«È¤á¤£±oÅý»P¥»¦X¬ù¤§¥þ³¡©Î³¡¤À¡C¥ô¦ó¹H³W¤§·N¹Ï§¡¬°µL®Ä¡C  
  
7. ·Ç¾Úªk¤ÎºÞÁÒ  
  
·Ç¾Úªk  
  
¶Q«È¤á»P IBM Âù¤è§¡¦P·N¥H ¶Q«È¤á¨ú±o¥»¡uµ{¦¡¡v±ÂÅv·í¦a¤§ªk«ß¬°·Ç¾Úªk¡A¾Ú¥H³W½d¡B¸ÑÄÀ¤Î°õ¦æ¥»¦X¬ù¥Øªº©Ò­l¥Í©Î¦³Ãö
©ó ¶Q«È¤á»P IBM Âù¤è¤§¤@¤ÁÅv§Q¡BÂ¾³d¤Î¸q°È¡F¥BµL¤Ï¹ï­ì«h¤§¾A¥Î¡C  
  
»P°ê»Ú³f«~¾P°â«´¬ù¬ÛÃö¤§Áp¦X°êºD¨Ò¤£¾A¥Î©ó¥»¦X¬ù¡C  
  
ºÞÁÒ  
  
Âù¤è¤§¤@¤ÁÅv§Q¡BÂ¾³d¤Î¸q°È§¡¨ü ¶Q«È¤á¨ú±o¥»µ{¦¡±ÂÅv·í¦a¤§ªk®xºÞÁÒ¡C  
  
²Ä¤G³¡¤À - ¦U°ê±M¦³±ø´Ú  
  
¨S¦³¾A¥Îªº¦U°ê±M¦³±ø´Ú¡C  
  
Z125-5544-03 (10/2005)  
±ÂÅv¤â¥U  
  
°£ °ê»Ú±ÂÅv¦X¬ù - ´ú¸Õª© ¥~¡A¥H¤U±ø´Ú¥ç¾A¥Î©ó¤U¦Cµ{¦¡¡C  
  
µ{¦¡¦WºÙ¡GalphaWorks Emerging Technology  
µ{¦¡½s¸¹¡GN/A  
  
¯S©w¹B§@Àô¹Ò  
  
¦pªG¥»µ{¦¡¦³ÀËªþ¤å¥ó¡A«h¦bµ{¦¡©ÒÀËªþ¤å¥ó¡]¦p Readme ÀÉ¡^©Î¦b IBM ©Ò¤½§Gªº¨ä¥L¸ê°T¡]¦p³qª¾«H¨ç¡^¤¤¡A¥i§ä¨ìµ{
¦¡³W®æ¤Î«ü©wªº§@·~Àô¹Ò¸ê°T¡C  
  
µû¦ô´Á¶¡  
  
µû¦ô´Á¦Û ¶Q«È¤á¦P·N¥»¦X¬ù¤§±ø´Ú¤é°\_ºâ¡A©ó 90 ¤é«á²×¤î¡C  
  
D/N: L-JLCO-6HQ6QK  
P/N: L-JLCO-6HQ6QK   
