# Supplementary material for: Finding New Order in Biological Functions from the Network Structure of Gene Annotations
Source: PLoS Comput Biol. 2015 Nov 20;11(11):e1004565. doi: 10.1371/journal.pcbi.1004565 (PMC4654495; doi:10.1371/journal.pcbi.1004565)
Supplement: S1 Code — This file contains the input human annotation files and all the code needed to reproduce the analyses and figures presented in this manuscript. The complete collection of intermediate files (such as the predicted term-term networks, word clouds for all communities, etc), can be obtained from [34]. (TGZ) [file pcbi.1004565.s004.tgz › TermCommunities_code/MakeCloudFiles/IBM Word Cloud/license/tr.html]

Software License

Programlarýn Ön Yayýn Düzeyleri Ýçin Uluslararasý Lisans
Sözleþmesi  
  
Bölüm 1 - Genel Koþullar  
  
PROGRAMLARIN ÖN YAYIN DÜZEYLERÝ ÝÇÝN ULUSLARARASI LÝSANS
SÖZLEÞMESÝ ("SÖZLEÞME"), SÝZÝNLE IBM ARASINDA GERÇEKLEÞTÝRÝLEN BÝR
YASAL SÖZLEÞMEDÝR. BU PROGRAMI YÜKLEYEREK, KURARAK, KOPYALAYARAK,
KULLANARAK YA DA BU PROGRAMA ERÝÞEREK, BU SÖZLEÞMENÝN KOÞULLARINI
KABUL EDÝYORSUNUZ. BU KOÞULLARI BÝR BAÞKA KÝÞÝ YA DA ÞÝRKET YA DA
TÜZEL KÝÞÝLÝK ADINA KABUL EDÝYORSANIZ, BU KÝÞÝ, ÞÝRKET YA DA
TÜZEL KÝÞÝNÝN BU KOÞULLARA UYMASINI SAÐLAMAK ÝÇÝN ELÝNÝZDE TAM
YETKÝ BULUNDUÐUNU BEYAN VE GARANTÝ EDERSÝNÝZ.  
  
"Ön Yayýn Düzeyi", (1) henüz geliþtirilmekte olan (ve bu
nedenle de potansiyel olarak güvenilmez olan) veya (2) daha önce
geliþtirilmiþ olan ancak ticari olarak piyasaya sunulmamýþ olan bir
Programýn yayýn düzeyi anlamýna gelir.   
  
"IBM", International Business Machines Corporation ya da
yan kuruluþlarýndan herhangi biri anlamýndadýr.  
  
"Lisans Bilgileri" ("LB"), bir Programa iliþkin özel
bilgileri ve koþullarý saðlayan bir belge anlamýndadýr. Programýn
LB'si ayný zamanda, bir sistem komutu kullanýlarak eriþilen
Program dizinindeki bir dosyada bulunabilir ya da Programla
birlikte bir kitapçýk biçiminde gönderilebilir.  
  
"Program", orijinalleri ve onun tüm kýsmi veya tam
kopyalarý da dahil olmak üzere, aþaðýdakilerden biri veya birden
fazlasý anlamýna gelir: 1) makinede okunabilir komutlar ve veriler,
2) kullanýcý tarafýndan okunabilir yazýlým bileþenleri, 3)
iþitsel-görsel içerik (örneðin, görüntüler, metinler, kayýtlar ya
da resimler), 4) iliþkili lisanslý malzeme, 5) lisans kullaným
belgeleri veya anahtarlarý, (6) iliþkili belgeler ve (7) tüm
geliþtirmeler, güncellemeler veya IBM'in kendi kararýyla Size Destek
olarak (aþaðýda açýklandýðý þekilde) saðlamayý seçebileceði
malzemeler.   
  
"Siz" ve "Sizin" sözcükleri, tek bir kiþi ya da tek bir
tüzel kiþilik için kullanýlýr.  
  
Bu Sözleþme, Bölüm 1 - Genel Koþullar, Bölüm 2 - Ülkeye
Özgü Koþullar (varsa) ve Lisans Bilgileri'ni içerir ve bu
Programýn kullanýmýna iliþkin Sizinle IBM arasýndaki sözleþmenin
tamamýný oluþturur. Bu Sözleþme, Programý kullanmanýzla ilgili
olarak Sizinle IBM arasýndaki daha önce gerçekleþtirilen her türlü
sözlü veya yazýlý iletiþimin yerine geçer. Bölüm 2'de ve Lisans
Bilgileri'nde yer alan koþullar, Bölüm 1'dekilerin yerine geçebilir veya
bunlarý deðiþtirebilir.  
  
1. Lisans  
  
Bu Programýn sahibi IBM ya da bir IBM saðlayýcýsýdýr.
Program telif hakkýna tabidir ve lisanslanýr, satýlmaz.  
  
IBM, Size bu Programý deðerlendirme süresi içinde yalnýzca
kurumunuz dahilinde denemeniz, deðerlendirmeniz ve IBM'e
görüþlerinizi bildirmeniz amaçlarýyla yüklemeniz, kurmanýz ve kullanmanýz
için sýnýrlý, münhasýr olmayan ve devredilemeyen bir lisans
verir.  
  
Bu tür bir kullanýmý desteklemek amacýyla bu Programýn bir
yedekleme kopyasýný çýkartabilirsiniz. Bu Programý üretim amaçlarýyla
kullanamaz veya Programý veya herhangi bir bölümünü daðýtamazsýnýz. Bu
Programý deðiþtiremez veya Programdan türetilen iþler
yaratamazsýnýz. Bu lisansýn koþullarý, çýkardýðýnýz her kopya için
geçerlidir. Programýn her bir tam ya da kýsmi kopyasýna, telif hakký
bildirimini ve mülkiyeti belirleyen diðer tüm iþaretleri eklemelisiniz.  
  
1) Programýn tüm kopyalarýnýn bir kaydýný saklamayý ve 2)
bu Programý kullanan herkesin (yerel ya da uzaktan eriþim
ile), yalnýzca Sizin yetkilendirildiðiniz kullaným kapsamýnda
Programý kullanmasýný ve bu Sözleþmenin koþullarýna uymasýný
saðlamayý kabul etmiþ sayýlýrsýnýz.   
  
1) Bu Programý bu Sözleþmede belirtilenler dýþýnda baþka
bir biçimde kullanamaz, kopyalayamaz, deðiþtiremez, devredemez
ya da daðýtamazsýnýz; 2) geçerli yasalarýn sözleþme ile
deðiþtirilmesine olanak tanýmayarak özel olarak izin verdiði durumlar
dýþýnda, Programý ters çeviremez, ters derleyemez veya baþka
yöntemlerle Programýn kullanýcýlar tarafýndan okunabilecek þekilde
çevirisini yapamazsýnýz veya diðer bir program diline çeviremezsiniz;
3) Programý alt lisanslayamaz, Programý kiralayamaz veya
kiraya veremezsiniz; ya da 4) Programý bir hizmet bürosunda
kullanamazsýnýz.  
  
Bu lisans ile Size IBM'den basýlý belgeler, destek, telefon
desteði veya Programa iliþkin geliþtirmeler veya güncellemeler
(topluca "Destek" olarak anýlýr) almanýz için yetki verilmez. Ancak,
IBM yalnýzca kendi vereceði karar doðrultusunda bu tür bir
Destek saðlamayý seçebilir. Desteðin bir parçasý olarak IBM
tarafýndan saðlanan tüm geliþtirmelerin, güncellemelerin ve diðer
malzemelerin bu Programýn bir bölümü olduðu ve bu nedenle bu tür
malzemelerin de bu Sözleþmeye tabi olduðu varsayýlýr.  
  
BU PROGRAM, DEÐERLENDÝRME SÜRESÝNÝN SONA ERMESÝNDEN SONRA
PROGRAMIN KULLANILMASINI ENGELLEYEN BÝR KULLANIMDAN ÇIKARMA AYGITI
(DISABLING DEVICE) ÝÇEREBÝLÝR. KULLANIMDAN ÇIKARMA AYGITLARINA YA DA
BU PROGRAMA MÜDAHALE EDEMEZSÝNÝZ. PROGRAM KULLANILAMAZ
OLDUÐUNDA ORTAYA ÇIKABÝLECEK VERÝ KAYBINA KARÞI ÖNLEMLERÝNÝZÝ
ALMALISINIZ.  
  
2. Süre  
  
Deðerlendirme süresi bu Sözleþmenin koþullarýný kabul
ettiðinizde baþlar ve aþaðýdakilerden hangisi daha önceyse, o tarihte
sona erer: 1) Lisans Bilgileri'nde belirtilen sona erme
tarihinde (eðer varsa), 2) Programýn kendisini otomatik olarak
geçersiz kýldýðý tarihte; ya da 3) IBM'in Programý ticari olarak
piyasaya sunduðu tarihte. Bu Programa iliþkin lisansýnýz
deðerlendirme süresinin sonunda sona erer; deðerlendirme süresi sona
erdikten sonra, on (10) gün içinde Programý ve Program kullanýlarak
oluþturulmuþ tüm kopyalarý imha etmeniz gerekir.  
  
Deðerlendirme süresi içinde Programý kullanýmýnýz herhangi
bir ücrete tabi deðildir. Bu Sözleþmenin koþullarýna uymamanýz
durumunda, IBM lisansýnýzý sona erdirebilir. IBM lisansýnýzý sona
erdirirse, Programýn tüm kopyalarýný imha etmeniz gerekir.  
  
3. Veriler Üzerindeki Haklar  
  
IBM'e, 1) Programla iliþkili ve 2) IBM'e Sizin tarafýnýzdan
saðlanan herhangi bir veri, öneri veya yazýlý malzeme üzerindeki
telif hakký da dahil olmak üzere tüm hak, ünvan ve menfaatlerini
devredersiniz. IBM isterse, bu tür haklarý devretmek için uygun bir
belgeyi imzalamayý kabul edersiniz. Bölüm 3'te yer alan ilk cümle
kapsamýnda IBM'e devrettiðiniz haklarýn baþka maddeleri kapsamamasý
koþuluyla, bu Programla iliþkili olan ve Sizin IBM'e saðladýðýnýz
patenti alýnabilen veya alýnamayan tüm fikirlerle, teknolojik
bilgiyle, kavramlarla, teknikle, buluþlarla, keþiflerle veya
geliþtirmelerle iliþkili olarak yukarýda belirtilenlerin tüm ürün veya
hizmetlerde yer almasý ve bu tür ürün veya hizmetlerin kullanýlmasý,
üretilmesi ve pazarlanmasý ve yukarýda belirtilenlerin diðer kiþilerce
yapýlmasýna olanak saðlamak amacýyla IBM'e münhasýr olmayan, geri
alýnamaz, sýnýrsýz, dünya çapýnda ve telif ücreti ödenmiþ bir hak ve
lisans verirsiniz.   
  
4. Garantinin Uygulanmamasý  
  
TABÝ BULUNDUÐU KANUNLARIN GARANTÝ KAPSAMI DIÞINDA
TUTULAMAYACAÐINI BELÝRTTÝÐÝ GARANTÝLERE TABÝ OLARAK (EÐER VARSA) IBM,
PROGRAMA VEYA TEKNÝK DESTEÐE (EÐER VARSA) ÝLÝÞKÝN OLARAK, ÜNVANA VE
HAK ÝHLALÝ YAPILMAYACAÐINA DAÝR GARANTÝLER ÝLE YETERLÝ KALÝTE,
TÝCARÝLÝK VE BELÝRLÝ BÝR AMACA UYGUNLUK ÝÇÝN ZIMNÝ GARANTÝLER VEYA
KOÞULLAR DA DAHÝL, VE FAKAT BUNLARLA SINIRLI OLMAKSIZIN, AÇIK VEYA
ZIMNÝ HÝÇBÝR GARANTÝ VERMEZ VEYA KOÞUL ÖNE SÜRMEZ.  
  
Bu hariç tutma, IBM'in tüm Program geliþtiricileri ve
saðlayýcýlarý için de geçerlidir.  
  
IBM dýþý Programlarýn üreticileri, saðlayýcýlarý ya da
yayýmcýlarý kendi garantilerini saðlayabilirler.  
  
5. Sorumluluklarýn Sýnýrý  
  
IBM'in bir kusuru veya sorumluluklarýný yerine
getirmemesinden kaynaklanan zararlarýnýzý IBM'den talep etmeye hak
kazandýðýnýz durumlar olabilir. Zararýnýzý IBM'den talep etmenize olanak
saðlayan yasal dayanak (Sözleþmenin esaslý ihlali, ihmal, yanlýþ
beyan, diðer ahdi iddialar veya haksýz fiil iddialarý dahil olmak
üzere) her ne olursa olsun, IBM yalnýzca: 1) ölüm dahil cismani
zarar ile taþýnmaz ve taþýnabilir kiþisel mallara verilen
zararlardan; ve 2) tüm iddialar için toplam olarak, herhangi bir diðer
gerçek doðrudan zarardan toplamý 25.000 ABD Dolarýný (veya yerel
para birimiyle eþdeðerini) aþmayacak þekilde sorumlu olur.
Sorumluluklarýn bu sýnýrý, IBM'in Program geliþtiricileri ve saðlayýcýlarý
için de geçerli olup, IBM'in, Program geliþtiricilerinin ve
saðlayýcýlarýnýn toplu olarak sorumlu olduklarý azami tutardýr.   
  
OLASILIKLARI ÖNCEDEN BÝLDÝRÝLMÝÞ OLSA BÝLE IBM, IBM'ÝN
PROGRAM GELÝÞTÝRÝCÝLERÝ VEYA SAÐLAYICILARI:   
  
1. VERÝLERDEKÝ KAYIP VEYA HASARDAN;   
2. ÖZEL, ARIZÝ, DOLAYLI, ÖRNEK NÝTELÝÐÝNDE VEYA CEZAÝ
ZARARLARDAN VEYA SONUÇTA ORTAYA ÇIKAN EKONOMÝK ZARARLARDAN; VEYA   
3. KAR KAYBI, ÝÞ KAYBI, GELÝR KAYBI, ÝTÝBAR KAYBI VEYA
BEKLENEN TASARRUF KAYBINDAN HÝÇBÝR KOÞUL ALTINDA SORUMLU OLMAZ.   
  
6. Genel  
  
1. Bu Sözleþmenin hiçbir koþulu, tüketicilerin sözleþme
yoluyla vazgeçilemeyen veya sýnýrlandýrýlamayan yasal haklarýný
etkilemez.  
2. Bu Sözleþmenin herhangi bir hükmü geçersiz ya da
uygulanamaz olduðunda, Sözleþmenin geri kalan hükümleri tam olarak
yürürlükte kalmaya ve geçerli olmaya devam eder.  
3. Bu Programý ihraç edemez veya Programla ilgili olarak
geçerli ihracat denetim yasalarýna aykýrý herhangi bir eylem
yapamazsýnýz.   
4. International Business Machines Corporation ve baðlý
kuruluþlarýnýn iþ yaptýklarý her yerde, isimler, iþ telefon numaralarý ve
iþ e-posta adresleri de dahil olmak üzere, Sizin iþ iletiþim
bilgilerinizi IBM ve baðlý kuruluþlarýnýn saklamalarýna ve kullanmalarýna
izin vermeyi kabul edersiniz. Bu tür bilgiler, IBM ile Sizin iþ
iliþkinizle baðlantýlý olarak iþlenecek ve kullanýlacak ve IBM adýna
faaliyet gösteren yüklenicilere, belirli IBM ürünlerinin ve
hizmetlerinin promosyonunu yapan, bunlarý pazarlayan ve bunlara iliþkin
destek saðlayan IBM Çözüm Ortaklarýna ve International Business
Machines Corporation ve baðlý kuruluþlarý tarafýndan atananlara bu
tür iþ iliþkilerine uygun olarak kullanýlmak üzere
verilebilecektir.   
5. IBM, bir Programýn herhangi bir sürümü resmi olarak
yayýnlandýðýnda veya ticari olarak piyasaya sunulduðunda (eðer varsa),
Programýn Ön Yayýn Düzeyleri sürümleri gibi olacaðýný ya da Ön Yayýn
Düzeyleri sürümleri ile uyumlu olacaðýný garanti etmez.  
6. Yerel yasalarýn, sözleþme ile deðiþtirilmesine veya
sýnýrlandýrýlmasýna olanak tanýmayarak aksini belirttiði durumlar dýþýnda,
taraflardan hiçbiri, dava nedeninin doðuþundan 2 yýldan fazla bir süre
geçtikten sonra bu Sözleþme kapsamýnda yasal bir iþleme baþvuramaz.  
7. Taraflardan hiçbiri, elinde olmayan nedenlerden ötürü
yükümlülüklerini yerine getirememekten sorumlu tutulamaz.   
8. Bu Sözleþme herhangi bir üçüncü kiþi için bir dava hakký
ya da bir dava nedeni yaratmamaktadýr ve ayrýca yukarýdaki
"Sorumluluklarýn Sýnýrý" baþlýklý bölümde izin verildiði ölçüde IBM'in yasal
olarak sorumlu olduðu (ölüm dahil) cismani zarar veya taþýnmaz
veya taþýnabilir kiþisel mallara verilen zararlar hariç olmak
üzere, üçüncü kiþiler tarafýndan Size yöneltilen iddialar
karþýsýnda IBM'in sorumluluðu bulunmamaktadýr.  
9. IBM'in önceden yazýlý izni olmaksýzýn, bu Sözleþmenin
bütününü veya herhangi bir bölümünü temlik edemezsiniz. Bu yöndeki
tüm giriþimler hükümsüzdür.   
  
7. Uygulanacak Hukuk ve Yetkili Mahkeme  
  
Uygulanacak Hukuk   
  
Taraflar, kanunlarýn ihtilafý prensibine bakýlmaksýzýn, bu
Sözleþmenin konusundan doðan veya bir biçimde bu Sözleþmenin konusu ile
ilgili olan IBM'e ve Size ait tüm haklar, görevler ve
yükümlülüklerin, Program lisansýný edindiðiniz ülkenin kanunlarýna tabi
olup, yine bunlarýn yorumlanmasý ve uygulanmasýnda bu kanunlarýn
geçerli olduðunu kabul ederler.  
  
Birleþmiþ Milletler'in Uluslararasý Mal Satýþlarýna iliþkin
Sözleþme Bildirgesi geçerli deðildir.  
  
Yetkili Mahkeme  
  
Taraflarýn tüm hak, görev ve yükümlülükleri, Program
lisansýný edindiðiniz ülkenin mahkemelerine tabidir.  
  
Bölüm 2 - Ülkeye Özgü Koþullar  
  
AVRUPA, ORTA DOÐU, AFRÝKA (EMEA)  
  
Verilere Ýliþkin Haklar (Bölüm 3):  
  
EMEA'da, aþaðýdaki maddeler bu bölümde yer alan koþullarýn
tümünün yerini alýr:  
  
IBM'e, 1) bu Programý kullanýmýnýzla iliþkili olan ve 2)
IBM'e Sizin tarafýnýzdan saðlanan herhangi bir veri, öneri ve
yazýlý malzeme üzerindeki telif hakký da dahil olmak üzere tüm
hak, ünvan ve menfaatlerini devredersiniz. Haklara iliþkin bu
devir iþlemine, yazýlý malzemeden türetilen iþlerin hazýrlanmasý
ve hazýrlatýlmasý, yazýlý malzemenin ve bu tür türetilmiþ
iþlerin herhangi bir ortamda ya da daðýtým teknolojisinde
kullanýlmasý ve kullandýrýlmasý, yürütülmesi, yeniden üretilmesi,
aktarýlmasý, görüntülenmesi, gerçekleþtirilmesi, devredilmesi,
daðýtýlmasý ve lisanslanmasý ile iþbu belgede yetkilendirilen bazý ya
da tüm haklarýn baþka kiþilere tüm bu hak, ünvan ve menfaatler
süresince verilmesine iliþkin haklar dahildir, ancak hepsi bunlarla
sýnýrlý deðildir. IBM isterse, bu haklarý devretmek için uygun bir
belgeyi imzalamayý kabul edersiniz. Bu Programla iliþkili olan ve
Sizin ve Sizin çalýþanlarýnýz tarafýndan deðerlendirme süresi
boyunca hazýrlanan, patenti alýnabilen veya alýnamayan tüm
fikirlerle, teknolojik bilgiyle, kavramlarla, teknikle, buluþlarla,
keþiflerle veya geliþtirmelerle iliþkili olarak yukarýda
belirtilenlerin tüm ürün veya hizmetlerde yer almasý ve bu tür ürün veya
hizmetlerin kullanýlmasý, üretilmesi ve pazarlanmasý ve yukarýda
belirtilenlerin diðer kiþilerce yapýlmasýna olanak saðlamak amacýyla IBM'e
münhasýr olmayan, geri alýnamaz, sýnýrsýz, dünya çapýnda ve telif
ücreti ödenmiþ bir hak ve lisans verirsiniz. Taraflardan hiçbiri,
verilere iliþkin haklar ya da bu Sözleþme'nin bir sonucu olarak
gerçekleþtirilen herhangi bir iþle ilgili olarak diðer taraftan ücret talep
etmeyecektir.  
  
Garantinin Uygulanmamasý (Bölüm 4):  
  
Avrupa Birliði'nde, bu bölümün baþýna aþaðýdaki cümle
eklenir:  
  
Avrupa Birliði'nde, tüketiciler, tüketici mallarýnýn
satýþýnýn tabi olduðu geçerli ulusal hukuk mevzuatý kapsamýnda yasal
haklara sahiptir. Bu tür haklar, Bölüm 4'te yer alan hükümler
tarafýndan etkilenmez.   
  
Uygulanacak Hukuk ve Yetkili Mahkeme (Bölüm 7)  
  
Yetkili Mahkeme  
  
Bu bölüme aþaðýdaki özel durum eklenmiþtir:  
  
Türkiye'de bu Sözleþmeden kaynaklanan ya da bu Sözleþmeyle
ilgili tüm anlaþmazlýklarýn çözülmesinde Türkiye Cumhuriyeti'nin,
Ýstanbul Merkez Ticaret Mahkemeleri ve Ýcra Daireleri yetkili
olacaktýr.  
  
Z125-5544-03 (10/2005)  
LÝSANS BÝLGÝLERÝ  
  
Aþaðýda listelenen programlar, Programlarýn Ön Yayýn
Düzeyleri Ýçin Uluslararasý Lisans Sözleþmesi kayýt ve koþullarýna ek
olarak aþaðýdaki kayýt ve koþullar kapsamýnda lisanslanmýþtýr.  
  
Program Adý: alphaWorks Emerging Technology  
Program Numarasý: N/A  
  
Tanýmlanmýþ Ýþletim Ortamý  
  
Programýn belirtimleri ve tanýmlanmýþ iþletim ortamý
bilgileri, Programla birlikte gönderilen, varsa READ-ME dosyasý gibi
belgelerde veya IBM tarafýndan yayýnlanan duyuru mektubu gibi diðer
bilgilerde bulunabilir.  
  
Deðerlendirme Süresi  
  
Deðerlendirme süresi, bu Sözleþmenin koþullarýný kabul
ettiðiniz tarihte baþlar ve 90 gün sonra sona erer.  
  
D/N: L-JLCO-6HQ6QK  
P/N: L-JLCO-6HQ6QK   
