# Supplementary material for: Finding New Order in Biological Functions from the Network Structure of Gene Annotations
Source: PLoS Comput Biol. 2015 Nov 20;11(11):e1004565. doi: 10.1371/journal.pcbi.1004565 (PMC4654495; doi:10.1371/journal.pcbi.1004565)
Supplement: S1 Code — This file contains the input human annotation files and all the code needed to reproduce the analyses and figures presented in this manuscript. The complete collection of intermediate files (such as the predicted term-term networks, word clouds for all communities, etc), can be obtained from [34]. (TGZ) [file pcbi.1004565.s004.tgz › TermCommunities_code/MakeCloudFiles/IBM Word Cloud/license/zh.html]

Software License

Ô¤·¢ÐÐ°æÈí¼þµÄ¹ú¼ÊÐí¿ÉÐ­Òé  
  
µÚÒ»²¿·Ö ¡ª Í¨ÓÃÌõ¿î  
  
´ËÔ¤·¢ÐÐ°æÈí¼þµÄ¹ú¼ÊÐí¿ÉÐ­Òé£¨¡°Ð­Òé¡±£©ÊÇÄúÓë IBM Ö®¼äµÄºÏ·¨Ð­Òé¡£Ò»µ©ÏÂÔØ¡¢°²×°¡¢¸´ÖÆ¡¢·ÃÎÊ»òÊ¹ÓÃ±¾Èí¼þ£¬¼´±íÃ÷Äú
Í¬Òâ±¾Ð­ÒéµÄÌõ¿î¡£Èç¹ûÄú´ú±íÆäËûÈË»òÄ³¸ö¹«Ë¾»òÆäËû·¨ÂÉÊµÌå½ÓÊÜ´ËÀàÌõ¿î£¬ÄúÉùÃ÷²¢±£Ö¤ÄúÒÑ¾­»ñµÃÍêÕûµÄÊÚÈ¨ÒÔ´ú±í´ËÈË¡¢¹«Ë¾»ò
·¨ÂÉÊµÌå½ÓÊÜ´ËÀàÌõ¿îµÄÔ¼Êø¡£  
  
¡°Ô¤·¢ÐÐ°æ¡±ÊÇ·ûºÏÒÔÏÂÇé¿öÖ®Ò»µÄ·¢ÐÐ°æÈí¼þ£º£¨1£©¸ÃÈí¼þ¿ÉÄÜÈÔ´¦ÓÚ¿ª·¢ÆÚÖÐ£¨Òò´Ë¾ßÓÐÇ±ÔÚµÄ²»¿É¿¿ÐÔ£©£»£¨2£©¿ÉÄÜ²»ÔÙ´¦ÓÚ
¿ª·¢ÆÚÖÐ£¬µ«»¹Ã»ÓÐÉÏÊÐÌá¹©¸øÓÃ»§Ê¹ÓÃ¡£  
  
¡°IBM¡±Ö¸ International Business Machines Corporation »òÆäÈÎÒ»×Ó¹«Ë¾¡£  
  
¡°Ðí¿ÉÐÅÏ¢¡±£¨¡°LI¡±£©Ö¸Ìá¹©×¨ÓÃÓÚÄ³Ò»Èí¼þµÄÐÅÏ¢ºÍÌõ¿îµÄÎÄµµ¡£¿ÉÒÔÍ¨¹ýÊ¹ÓÃÏµÍ³ÃüÁîÔÚÈí¼þÄ¿Â¼ÏÂµÄÄ³¸öÎÄ¼þÖÐÕÒµ½Èí¼þµÄ
¡°Ðí¿ÉÐÅÏ¢¡±¡£¡°Ðí¿ÉÐÅÏ¢¡±»¹¿É×÷ÎªÊÖ²áÓëÈí¼þÒ»ÆðËæ¸½¡£  
  
¡°Èí¼þ¡±Ö¸ÒÔÏÂÒ»Ïî»ò¶àÏî£¬°üÀ¨Ô­¼þºÍËùÓÐÍêÕû¸±±¾»ò²¿·Ö¸±±¾£º1£©»ú¶ÁÖ¸ÁîºÍÊý¾Ý£¬2£©¿É¶ÁÈí¼þ×é¼þ£¬3£©ÊÓÌýÄÚÈÝ£¨ÀýÈçÍ¼
Ïñ¡¢ÎÄ±¾¡¢Â¼Òô»òÍ¼Æ¬£©£¬4£©Ïà¹ØµÄ±»Ðí¿ÉµÄ²ÄÁÏ£¬5£©Ðí¿ÉÊ¹ÓÃÎÄµµ»òÃÜÔ¿£¬6£©¸½¼ÓÎÄµµ£¬7£©ÈÎºÎ IBM ¿ÉÒÔ×ÔÐÐÑ¡Ôñ×÷ÎªÖ§³Ö
Ìá¹©¸øÄúµÄÇ¿Ôöµã¡¢¸üÐÂ»ò×ÊÁÏ£¨Çë²ÎÔÄÏÂÃæµÄÃèÊö£©  
  
¡°Äú¡±ºÍ¡°ÄúµÄ¡±Ö¸¸öÈË»òµ¥¸öµÄ·¨ÂÉÊµÌå¡£  
  
±¾Ð­Òé°üÀ¨µÚÒ»²¿·Ö£¨Í¨ÓÃÌõ¿î£©¡¢µÚ¶þ²¿·Ö£¨¹ú¼Ò»òµØÇøÌØ±ðÌõ¿î£©£¨ÈçÓÐ£©ºÍÐí¿ÉÐÅÏ¢£¬ÎªÄúÓë IBM Ö®¼äÓÐ¹ØÈí¼þÊ¹ÓÃµÄÍêÕû
Ð­Òé¡£±¾Ð­ÒéÌæ´úÄúÓë IBM Ö®¼äÓÐ¹ØÈí¼þÊ¹ÓÃµÄÈÎºÎÏÈÇ°µÄ¿ÚÍ·»òÊéÃæÍ¨ÐÅ¡£µÚ¶þ²¿·ÖµÄÌõ¿îºÍÐí¿ÉÐÅÏ¢¿ÉÄÜÌæ´ú»òÐÞ¸ÄµÚÒ»²¿·ÖµÄ
ÏàÓ¦ÄÚÈÝ¡£  
  
1. Ðí¿É  
  
Èí¼þÓÉ IBM »òÆä¹©Ó¦ÉÌËùÓÐ£¬ÊÜ°æÈ¨±£»¤¡£Èí¼þÏµÐí¿ÉÊ¹ÓÃ£¬¶ø·Ç³öÊÛ¡£  
  
IBM ÊÚÓèÄúÓÐÏÞµÄ¡¢·Ç×¨ÊôµÄ²¢ÇÒ²»¿É×ªÈÃµÄÐí¿ÉÒÔÏÂÔØ¡¢°²×°ºÍ½öÎªÓÚÆÀ¹ÀÖÜÆÚÄÚ½øÐÐÄÚ²¿²âÊÔºÍÆÀ¹ÀÄ¿µÄ¶øÊ¹ÓÃ¸ÃÈí¼þ£¬²¢ÇÒÄú
ÒªÏò IBM Ìá¹©·´À¡Òâ¼û¡£  
  
ÎªÁËÖ§³Ö´ËÀàÊ¹ÓÃ£¬Äú¿ÉÒÔÖÆ×÷¸ÃÈí¼þµÄ±¸·Ý¸±±¾¡£ÄúÎ´±»ÊÚÈ¨½«±¾Èí¼þÓÃÓÚÉú²ú»ò·Ö·¢±¾Èí¼þ»òÆäÈÎºÎ²¿·Ö¡£Äú²»ÄÜÐÞ¸Ä»ò´´½¨±¾³ÌÐò
µÄÑÝÒï×÷Æ·¡£±¾Ðí¿ÉµÄËùÓÐÌõ¿îÊÊÓÃÓÚÄúÖÆ×÷µÄÃ¿Ò»·Ý¸±±¾¡£ÄúÐëÔÚ±¾Èí¼þµÄÃ¿Ò»ÍêÕû¸±±¾»ò²¿·Ö¸±±¾ÖÐ¸´ÖÆËùÓÐµÄ°æÈ¨ÉùÃ÷ºÍËùÓÐÆäËûËù
ÓÐÈ¨ËµÃ÷¡£  
  
ÄúÐë 1£©±£ÁôÈí¼þËùÓÐ¸´ÖÆ¼þµÄ¼ÇÂ¼£¬²¢ 2£©È·±£ËùÓÐÊ¹ÓÃÈí¼þµÄÈËÔ±£¨²»ÂÛÊÇ±¾µØ»òÔ¶³Ì·ÃÎÊ£©¶¼½öÔÚÄú±»ÊÚÈ¨µÄ·¶Î§ÄÚÕâÑùÊ¹ÓÃ
Èí¼þ£¬²¢ÇÒ×ñÊØ±¾Ð­ÒéÖÐµÄÌõ¿î¡£  
  
Äú²»¿ÉÒÔ£º1£©Ê¹ÓÃ¡¢¸´ÖÆ¡¢ÐÞ¸Ä¡¢×ªÈÃ»ò·Ö·¢±¾Èí¼þ£¬³ý·Ç±¾Ð­ÒéÁíÓÐ¹æ¶¨£»2£©·´»ã±à¡¢·´±àÒë»òÒÔÆäËû·½·¨±àÒë±¾³ÌÐò£¬µ«·¨ÂÉ¹æ
¶¨²»µÃÍ¨¹ýÔ¼¶¨¼ÓÒÔ·ÅÆúÕß£¬²»ÔÚ´ËÏÞ£»»ò 3£©ÔÙÐí¿É¡¢³ö×â»ò×âÁÞ±¾Èí¼þ£»4£©ÔÚ·þÎñ´¦Ïà¹ØÊÂÎñÖÐÊ¹ÓÃ±¾Èí¼þ¡£  
  
¸ÃÐí¿ÉÃ»ÓÐÊÚÓèÄú½ÓÊÕ IBM Ó²¿½±´ÎÄµµ¡¢Ö§³Ö¡¢µç»°°ïÖú»òÈí¼þµÄÔöÇ¿µãºÍ¸üÐÂ£¨È«²¿µÄ¡°Ö§³Ö¡±£©µÄÈ¨Àû£¬¾¡¹Ü IBM ¿ÉÒÔ
×ÔÐÐÑ¡ÔñÌá¹©´ËÀàÖ§³Ö¡£ÈÎºÎÓÉ IBM ×÷ÎªÖ§³ÖµÄÒ»²¿·ÖÌá¹©µÄÔöÇ¿µã¡¢¸üÐÂºÍÆäËû×ÊÁÏ½«×÷ÎªÈí¼þµÄÒ»²¿·Ö¶øÊÜ±¾Ð­ÒéµÄ¹ÜÏ½¡£  
  
Èí¼þ¿ÉÄÜ°üº¬½ûÓÃ×°ÖÃ£¬ÒÔ·ÀÆÀ¹ÀÆÚÏÞ¹ýºóÈÔ¼ÌÐø±»Ê¹ÓÃ¡£Äú²»µÃÉÃ¸Ä½ûÓÃ×°ÖÃ»ò±¾Èí¼þ¡£ÄúÓ¦µ±²ÉÈ¡Ô¤·À´ëÊ©ÒÔ±ÜÃâÒòÈí¼þ²»ÄÜ¼ÌÐø±»
Ê¹ÓÃ¶ø¿ÉÄÜµ¼ÖÂµÄÈÎºÎÊý¾ÝµÄËðÊ§¡£  
  
2. Ìõ¿î  
  
ÆÀ¹ÀÆÚÏÞÓÚÄúÍ¬Òâ±¾Ð­ÒéÌõ¿îÊ±¿ªÊ¼£¬ÓÚÏÂÁÐÇéÐÎÖ®Ò»·¢ÉúÖ®ÈÕÖÕÖ¹£º1£©µ½ÁË¡°Ðí¿ÉÐÅÏ¢¡±ÖÐ¹æ¶¨µÄ½áÊøÈÕÆÚ£¨Èç¹ûÓÐµÄ»°£©£»2£©Èí
¼þ×Ô¶¯½ûÓÃ£»3£©IBM ½«Èí¼þÉÏÊÐ¡£ÄúµÄÈí¼þÐí¿ÉÖ¤ÔÚÆÀ¹ÀÆÚ½áÊøÊ±µ½ÆÚ£¬ÄúÐëÔÚÆÀ¹ÀÆÚÏÞ½áÊøºóÊ®£¨10£©ÌìÄÚÏú»Ù±¾Èí¼þ¼°ÆäËùÓÐ
¸±±¾¡£  
  
ÔÚÆÀ¹ÀÆÚÏÞÄÚ¿ÉÃâ·ÑÊ¹ÓÃ±¾Èí¼þ¡£  
  
Èç¹ûÄúÎ´ÄÜ×ñÊØ±¾Ð­ÒéµÄÌõ¿î£¬Ôò IBM ¿ÉÒÔÖÕÖ¹ÄúµÄÐí¿É¡£Èç¹û IBM ÖÕÖ¹ÄúµÄÐí¿É£¬Äú±ØÐëÏú»Ù±¾³ÌÐòµÄËùÓÐ¸±±¾¡£  
  
3. ÓëÊý¾ÝÏà¹ØµÄÈ¨Àû  
  
Äú½« 1£©Óë¸ÃÈí¼þÏà¹ØµÄ£¬²¢ÇÒ 2£©ÓÉÄúÌá¹©¸ø IBM µÄÈÎºÎÊý¾Ý¡¢½¨ÒéºÍÊéÃæ²ÄÁÏµÄËùÓÐÈ¨Àû¡¢ËùÓÐÈ¨ºÍÈ¨Òæ£¨°üÀ¨°æÈ¨£©×ª
ÈÃ¸ø IBM¡£Èç¹û IBM ÒªÇó£¬Äú½«Ç©ÊðÏàÓ¦µÄÎÄµµÒÔ×ªÈÃ´ËÀàÈ¨Àû¡£Èç¹ûÃ»ÓÐ±»±¾µÚÈý£¨3£©½ÚµÚÒ»¾ä»°ÖÐÄú×¼ÐíµÄÄÚÈÝº­¸Ç£¬¹Ø
ÓÚÄúÌá¹©¸ø IBM µÄÈÎºÎ¹Ûµã¡¢Êµ¼ùÖªÊ¶¡¢¸ÅÄî¡¢¼¼Êõ¡¢·¢Ã÷¡¢·¢ÏÖ»ò¸Ä½ø£¬ÎÞÂÛÊÇ·ñ»ñµÃ×¨ÀûÒÔ¼°ÓëÈí¼þÏà¹Ø£¬ÄúÊÚÓè IBM ÈÎ
ºÎÇ°ÊöµÄ²úÆ·Óë·þÎñ·Ç×¨ÊôµÄ¡¢²»¿ÉÈ¡Ïû²¢²»ÊÜÏÞÖÆµÄÈ«Çò·¶Î§ÒÑ¸¶·ÑµÄÈ¨ÀûºÍÐí¿É£¬ÔÊÐí IBM Ê¹ÓÃ¡¢Éú²úºÍÓªÏúÈÎºÎ´ËÀà²úÆ·»ò·þ
Îñ£¬Í¬Ê±ÔÊÐíËûÈËÊµÊ©Ç°ÊöµÄÄÚÈÝ¡£  
  
4. ²»±£Ö¤ÉùÃ÷  
  
³ýÁËÄ³Ð©²»ÄÜ±»ÅÅ³ýµÄ·¨¶¨±£Ö¤£¨Èç¹ûÓÐµÄ»°£©£¬IBM ²»¶ÔÓÐ¹ØÈí¼þ»ò¼¼ÊõÖ§³ÖÌá¹©ÈÎºÎÆäËûÃ÷Ê¾»ò°µº¬µÄ±£Ö¤ºÍÌõ¼þ£¬°üÀ¨µ«²»ÏÞ
ÓÚ°µº¬µÄÓÐ¹ØÁ¼ºÃÆ·ÖÊ¡¢ÊÊÏú¡¢ÊÊÓÃÓÚÄ³ÖÖÌØ¶¨ÓÃÍ¾¡¢²úÈ¨±£Ö¤ºÍ·ÇÇÖÈ¨µÄ±£Ö¤»òÌõ¼þ£¨Èç¹ûÓÐµÄ»°£©¡£  
  
´ËÏîÅÅ³ýÒ²ÊÊÓÃÓÚÈÎºÎ IBM µÄÈí¼þ¿ª·¢ÉÌºÍ¹©Ó¦ÉÌ¡£  
  
·Ç IBM Èí¼þµÄÉú²ú³§¼Ò¡¢¹©Ó¦ÉÌ»ò·¢ÐÐÈË¿ÉÒÔÌá¹©ËûÃÇ×Ô¼ºµÄÓÐ¹ØÈí¼þµÄ±£Ö¤¡£  
  
5. Åâ³¥ÔðÈÎ  
  
Èç¹ûÒò IBM µÄ¹ýÊ§»òÆäËûÔðÈÎ£¬ÄúÓÐÈ¨ÒªÇó IBM Åâ³¥ËðÊ§¡£ÔÚ´ËÖÖÇé¿öÏÂ£¬ÎÞÂÛÄúÒÔºÎÖÖÒÀ¾Ý¶øÓÐÈ¨ÒªÇó IBM Åâ³¥Ëð
Ê§£¬¶ÔÓÚÈÎºÎÖ¸¿Ø£¨°üÀ¨¸ù±¾Î¥Ô¼¡¢Êèºö¡¢´íÎó³ÂÊö»òÆäËûºÏÔ¼»òÇÖÈ¨·½ÃæµÄË÷Åâ£©£¬IBM ³Ðµ£µÄÔðÈÎ½öÏÞÓÚ£º1£©ÈËÉíÉËº¦£¨°üÀ¨ËÀ
Íö£©µÄËðº¦Åâ³¥¼°¶Ô²»¶¯²úºÍÓÐÐÎ¶¯²úµÄËðº¦Åâ³¥£¬2£©ÈÎºÎÆäËûÊµ¼ÊÖ±½ÓËðº¦Åâ³¥£¬Åâ³¥½ð¶î×î¸ßÎª 25,000 ÃÀÔª£¨»òµÈÖµµÄÄúµ±
µØµÄ»õ±Ò£©¡£´ËÅâ³¥ÔðÈÎÒ²ÊÊÓÃÓÚ IBM µÄÈí¼þ¿ª·¢ÉÌºÍ¹©Ó¦ÉÌ¡£¸ÃÏÞ¶îÊÇ IBM ÓëÆäÈí¼þ¿ª·¢ÉÌºÍ¹©Ó¦ÉÌÐë¹²Í¬µÄ×î¸ßÔðÈÎÏÞ
¶î¡£  
  
ÎÞÂÛÈçºÎ£¬IBM ¼°ÆäÈí¼þ¿ª·¢ÉÌºÍ¹©Ó¦ÉÌ¾ù²»¶ÔÏÂÁÐ¸÷Ïî³Ðµ£ÔðÈÎ£¬¼´Ê¹±»¸æÖªÆä·¢ÉúµÄ¿ÉÄÜÐÔÊ±£¬Ò²ÊÇÈç´Ë£º  
  
1. ¼ÇÂ¼»òÊý¾ÝµÄ¶ªÊ§»òËð»µ£»  
2. ÌØ±ðµÄ¡¢¸½´øµÄ¡¢¼ä½ÓµÄ¡¢³Í½äÐÔ»ò´¦·£ÐÔËðÊ§£¬»òÈÎºÎºó¹ûÐÔ¾­¼ÃËðÊ§£»»ò  
3. ÀûÈóµÄËðÊ§¡¢ÉúÒâµÄ¶ªÊ§¡¢ÓªÒµ¶îµÄ¼õÉÙ¡¢ÉÌÓþ»òÔ¤ÆÚ¿É½ÚÊ¡½ð¶îµÄËðÊ§¡£  
  
6. Í¨Ôò  
  
1. ±¾Ð­ÒéÖÐµÄÈÎºÎ¹æ¶¨¾ù²»Ó°ÏìÏû·ÑÕßÓµÓÐµÄ£¬¶øÇÒ²»µÃÒÔÐ­ÒéÐÎÊ½¼ÓÒÔÏÞ¶¨»òÃâ³ýµÄ·¨¶¨È¨Àû¡£  
2. Èç¹û±¾Ð­ÒéµÄÈÎºÎÌõ¿î±»²Ã¶¨ÎªÎÞÐ§»ò²»¿ÉÖ´ÐÐ£¬¸ÃÎÞÐ§»ò²»¿ÉÖ´ÐÐÌõ¿î²»Ó°Ïì±¾Ð­ÒéÆäËûÌõ¿îµÄÐ§Á¦¡£  
3. Äú³ö¿Ú±¾³ÌÐò»ò¶ÔÆä²ÉÈ¡ÈÎºÎ×ö·¨²»µÃÎ¥·´ÊÊÓÃµÄ³ö¿Ú¹ÜÖÆ·¨Áî¡£  
4. ÄúÍ¬ÒâÔÊÐí International Business Machines Corporation ¼°Æä×Ó¹«Ë¾ÔÚÆä
ÈÎºÎÓªÒµ³¡Ëù´æ´¢ºÍÊ¹ÓÃÄúµÄÒµÎñÁªÏµÐÅÏ¢£¬°üÀ¨ÐÕÃû¡¢ÒµÎñµç»°ºÅÂëºÍÒµÎñµç×ÓÓÊ¼þµØÖ·¡£´ËÀàÐÅÏ¢½«ÎªË«·½ÒµÎñ¹ØÏµµÄÏà¹ØÊÂÏî½øÐÐ´¦
ÀíºÍÊ¹ÓÃ£¬²¢¿ÉÌá¹©¸ø´ú±í IBM µÄ³Ð°üÉÌ£¬Ðû´«¡¢ÏúÊÛ²¢Ö§³ÖÒ»¶¨µÄ IBM ²úÆ·ºÍ·þÎñµÄ IBM ÒµÎñºÏ×÷»ï°éºÍ
International Business Machines Corporation ¼°Æä×Ó¹«Ë¾µÄÊÜÈÃÈËÒÔ¹©Æä¹²Í¬µÄÒµÎñÍùÀ´Ê¹ÓÃ¡£  
5. IBM ²»±£Ö¤ÕýÊ½·¢ÐÐ»òÉÏÊÐµÄÈí¼þ£¨Èç¹ûÓÐµÄ»°£©½«ÓëÔ¤·¢ÐÐ°æÏàËÆ»ò¼æÈÝ¡£  
6. ÎÞÂÛÊÇÄú»¹ÊÇ IBM£¬¾ù²»µÃÔÚËßÒò·¢ÉúÁ½£¨2£©Äêºó·½¾Ý´ËÐ­ÒéÌáÆðËßËÏ£¬µ«µ±µØ·¨ÂÉÁíÓÐ¹æ¶¨²»µÃÒÔÔ¼¶¨·ÅÆú»òÏÞÖÆÕß³ý
Íâ¡£  
7. ÎÞÂÛÊÇÄú»¹ÊÇ IBM£¬¾ù²»¶ÔÒò²»¿É¿ØÖÆµÄÔ­Òò¶øÎÞ·¨ÂÄÐÐµÄÈÎºÎÒåÎñ¸ºÔð¡£  
8. ±¾Ð­Òé½«²»ÎªÈÎºÎµÚÈý·½´´½¨ÈÎºÎÈ¨Àû»òËßÒò£¬IBM Ò²²»¶ÔÈÎºÎµÚÈý·½¶ÔÄúµÄË÷Åâ¸ºÔð£¬µ«£¬ÒÀ¾ÝÒÔÉÏ¡°ÔðÈÎÏÞÖÆ¡±Ò»½ÚµÄÐí
¿É£¬¶ÔÓÚ IBM Ó¦¸º·¨ÂÉÔðÈÎµÄÈËÉíÉËº¦£¨°üÀ¨ËÀÍö£©»ò²»¶¯²ú»òÓÐÐÎ¶¯²úµÄË÷Åâ³ýÍâ¡£  
9. Ã»ÓÐ IBM ÊÂÏÈµÄÊéÃæÍ¬Òâ£¬Äú²»ÄÜ×ªÈÃ±¾Ð­ÒéÈ«²¿»ò²¿·ÖÄÚÈÝ¡£ÈÎºÎÕâÑùµÄ³¢ÊÔ¶¼ÎÞ·¨ÂÉÐ§Á¦¡£  
  
7. ÊÊÓÃ·¨ÂÉºÍË¾·¨Ï½Çø  
  
ÊÊÓÃ·¨ÂÉ  
  
ÄúÓë IBM Ë«·½¾ùÍ¬Òâ£¬Äú»ñµÃÈí¼þÐí¿ÉÖ¤ËùÔÚµÄ¹ú¼Ò»òµØÇøµÄ·¨ÂÉ½«¹ÜÏ½¡¢½âÊÍºÍÇ¿ÖÆÖ´ÐÐ±¾Ð­Òé±êµÄÒýÆðµÄ»òÒÔÈÎºÎ·½Ê½ÓëÖ®Ïà
¹ØµÄÄúÓë IBM µÄÈ¨Àû¡¢ÔðÈÎºÍÒåÎñ£¬¶ø²»¿¼ÂÇ³åÍ»·¨Ô­Ôò¡£  
  
¡¶ÁªºÏ¹ú¹ú¼Ê»õÎïÏúÊÛºÏÍ¬¹«Ô¼¡·²»ÊÊÓÃ±¾Ð­Òé¡£  
  
Ë¾·¨Ï½Çø  
  
Ë«·½µÄËùÓÐÈ¨Àû¡¢ÔðÈÎºÍÒåÎñ¾ùÊÜÄú»ñµÃÈí¼þÐí¿ÉÖ¤ËùÔÚµÄ¹ú¼Ò»òµØÇøµÄ·¨ÔºµÄ¹ÜÏ½¡£  
  
µÚ¶þ²¿·Ö ¡ª ¹ú¼Ò»òµØÇøÌØ±ðÌõ¿î  
  
ÑÇÌ«µØÇø  
  
ÖÐ¹úÏã¸ÛÌØ±ðÐÐÕþÇøÓë°ÄÃÅÌØ±ðÐÐÕþÇø£ºÊÊÓÃ·¨ÂÉºÍË¾·¨Ï½Çø£¨µÚÆß½Ú£©£º ¡°ÊÊÓÃ·¨ÂÉ¡±·Ö¿îÖÐµÄ¡°Äú»ñµÃÈí¼þÐí¿ÉÖ¤ËùÔÚµÄ¹ú¼Ò»òµØ
ÇøµÄ·¨ÂÉ¡±Ìæ»»Îª£º  
  
ÖÐ¹úÏã¸ÛÌØ±ðÐÐÕþÇøµÄ·¨ÂÉ  
  
ÖÐ»ªÈËÃñ¹²ºÍ¹ú£ºÊÊÓÃ·¨ÂÉºÍË¾·¨Ï½Çø£¨µÚÆß½Ú£©£º ¡°ÊÊÓÃ·¨ÂÉ¡±·Ö¿îÖÐµÄ¡°Äú»ñµÃÈí¼þÐí¿ÉÖ¤ËùÔÚµÄ¹ú¼Ò»òµØÇøµÄ·¨ÂÉ¡±Ìæ»»Îª£º  
  
ÃÀ¹úÅ¦Ô¼ÖÝ·¨ÂÉ£¨µ«µ±µØ·¨ÂÉÁíÓÐÒªÇóµÄ³ýÍâ£©  
  
Z125-5544-03 (10/2005)  
Ðí¿ÉÐÅÏ¢  
  
³ý Ô¤·¢ÐÐ°æÈí¼þµÄ¹ú¼ÊÐí¿ÉÐ­Òé ÖÐµÄÌõ¿îºÍÌõ¼þÍâ£¬ÏÂÁÐ¸÷³ÌÐò°´ÕÕÏÂÊöÌõ¿îºÍÌõ¼þ±»ÌØÐíÊ¹ÓÃ¡£  
  
³ÌÐòÃû³Æ£ºalphaWorks Emerging Technology  
³ÌÐòºÅ£ºN/A  
  
Ö¸¶¨²Ù×÷»·¾³  
  
ÓÐ¹Ø±¾³ÌÐòËµÃ÷ºÍÖ¸¶¨²Ù×÷»·¾³µÄÐÅÏ¢£¬¿ÉÒÔÔÚËæ±¾³ÌÐòÌá¹©µÄÎÄµµÀýÈçËµÃ÷ÎÄ¼þÖÐ£¨ÈçÓÐ£©ÕÒµ½£¬Ò²¿ÉÍ¨¹ý IBM ¹«²¼µÄÆäËûÐÅÏ¢
£¬ÀýÈç¹«¸æº¯¼þÕÒµ½¡£  
  
ÆÀ¹ÀÆÚÏÞ  
  
ÆÀ¹ÀÆÚÏÞÓÚÄúÍ¬Òâ±¾Ð­ÒéÌõ¿îÖ®ÈÕ¿ªÊ¼£¬ÓÚ 90 ÈÕºóÖÕÖ¹¡£  
  
D/N: L-JLCO-6HQ6QK  
P/N: L-JLCO-6HQ6QK   
