# Supplementary material for: Finding New Order in Biological Functions from the Network Structure of Gene Annotations
Source: PLoS Comput Biol. 2015 Nov 20;11(11):e1004565. doi: 10.1371/journal.pcbi.1004565 (PMC4654495; doi:10.1371/journal.pcbi.1004565)
Supplement: S1 Code — This file contains the input human annotation files and all the code needed to reproduce the analyses and figures presented in this manuscript. The complete collection of intermediate files (such as the predicted term-term networks, word clouds for all communities, etc), can be obtained from [34]. (TGZ) [file pcbi.1004565.s004.tgz › TermCommunities_code/MakeCloudFiles/IBM Word Cloud/readme.html]

IBM Word Cloud Generator


## IBM Word Cloud Generator

### Build 32

The IBM Word Cloud Generator is a Java application that creates
word clouds from your source texts. It's the same technology
that powers the popular "Wordle" web application.

## Usage

|  |
| --- |
| ``` java -jar ibm-word-cloud.jar options     Options:          mandatory: -c|--config /path/to/configuration          optional: -w|--width integer           optional: -h|--height integer           optional: -s|--seed longInteger          optional: -i|--infile /path/to/input.txt          optional: -o|--outfile /path/to/output.png          optional: -p|--print ``` |

The application uses a configuration file to control all of the
settings that affect the output, such as font, layout, the
treatment of stop-words, etc.

The default width and height are 800 and 1200, respectively.

If an input file is not provided, the application will read standard in.

If an output file is not provided, the application will write to standard out.
The output is always a 24-bit PNG.

You may only specify one of `-p/--print` or `-o/--outfile`. If you use the
print option, then the width and height are used to determine an aspect
ratio. You could tell the word cloud generator to try to make a square
by doing

|  |
| --- |
| ``` $java -jar ibm-word-cloud.jar -c myconfig.txt -w 1 -h 1 -p ``` |

for example.

The `-s` or `--seed` option allows
you to seed the random number generator so that any two runs
using the same configuration *and having the same aspect ratio
with respect to the width and height arguments*
will produce identical output.

## Sample Usages

|  |
| --- |
| ``` $java -jar ibm-word-cloud.jar -c myconfig.txt < /path/to/text > /path/to/output.png $cat file1 file2 | java -jar ibm-word-cloud.jar -s 133277 -c myconfig.txt -o /path/to/output.png $java -jar ibm-word-cloud.jar --config myconfig.txt  --input /path/to/text --output /path/to/output.png ``` |

See the included `run-example.bat` or `run-example.sh`
for a command line that should work with this distribution.

## Configuration

See a heavily commented sample configuration file.

A configuration file is a UTF-8-encoded text file. Blank lines, and
lines beginning with the `#` character are ignored.
Lines that set properties consist of a property name, optional
whitespace, the `:` character, optional whitespace,
and the desired property value. Example:

|  |
| --- |
| ``` # This is a comment  # The next line sets the "font" preperty font: /opt/fonts/ttf/helvetica.ttf ``` |
