# Supplementary material for: Finding New Order in Biological Functions from the Network Structure of Gene Annotations
Source: PLoS Comput Biol. 2015 Nov 20;11(11):e1004565. doi: 10.1371/journal.pcbi.1004565 (PMC4654495; doi:10.1371/journal.pcbi.1004565)
Supplement: S1 Code — This file contains the input human annotation files and all the code needed to reproduce the analyses and figures presented in this manuscript. The complete collection of intermediate files (such as the predicted term-term networks, word clouds for all communities, etc), can be obtained from [34]. (TGZ) [file pcbi.1004565.s004.tgz › TermCommunities_code/MakeCommunities/igraph_mod.tar.gz!/igraph/inst/tkigraph_help/communities.html]

Community structure detection


Bla-bla-bla
