# Supplementary material for: Finding New Order in Biological Functions from the Network Structure of Gene Annotations
Source: PLoS Comput Biol. 2015 Nov 20;11(11):e1004565. doi: 10.1371/journal.pcbi.1004565 (PMC4654495; doi:10.1371/journal.pcbi.1004565)
Supplement: S1 Code — This file contains the input human annotation files and all the code needed to reproduce the analyses and figures presented in this manuscript. The complete collection of intermediate files (such as the predicted term-term networks, word clouds for all communities, etc), can be obtained from [34]. (TGZ) [file pcbi.1004565.s004.tgz › TermCommunities_code/MakeCommunities/igraph_mod.tar.gz!/igraph/inst/tkigraph_help/index.html]

tkigraph online help


# The tkigraph manual

tkigraph is a basic Graphical User Interface (GUI) to some
igraph functions.

## What is tkigraph?

tkigraph is a simple Graphical User Interface to the igraph R
package. R is a general purpose programming language and
environment, used mostly but not exclusively for statistical
analysis. igraph is an extension package to R. tkigraph lets you
use some basic features of igraph via a GUI, instead of typing in
R commands.

## Installing and starting tkigraph

Well, if you are reading these lines, then you probably already
know how to install and start tkigraph.
If not, here is how to do it.

First, install the GNU R software package. It can be downloaded
from *the R website*, but
first check your system, because it might be already
installed. You can also ask your system administrator to
install it for you.

Second, you need to install the igraph extension
package. First, start R by clicking on its icon in Windows, or
by typing "R" into a terminal and pressing ENTER on Linux.
Now type in

```
	install.package("igraph")
```

and press ENTER. After choosing an appropriate mirror site, R
downloads and installs the igraph package.

Third, you need to load the igraph package and start
tkigraph. This can be done by typing

```
	library(igraph)
	tkigraph()
```

(in two separate lines, pressing ENTER after each line)
into your R session. You should see a new windows appear, it
looks like the one on the picture below.

## The tkigraph window

The main window of tkigraph look like this:

Almost all the window is occupied by the list of graphs in the
workspace. Unlike on the picture for you this is initially
empty. Every graph has a number, in the `#` column, a
name that is not necessarily unique, you can change the name of
the graph to whatever you like. In the last three columns you
can see the number of vertices and edges in the graph, and
whether it is directed or not.

In the leftmost column there is a checkbox for every graph, you
can select one or more graphs using this and then perform
operations on them. Some operations require exactly one graph to
be selected, others work happily on many graphs as well. You
will always get an error message if not the appropriate number
of graphs were selected for an operation.

## The tkigraph menus

Creating new graphs or performing operations on them can be
done by selecting entries from the main menu. Let us discuss
briefly what the various menus are good for.

### Graph menu

The Graph menu lets you create and delete graphs, show them in
an edge list format, calculate some basic properties for
them. Moreover all file-related operations are here a well.

### Draw menu

In this menu you can draw your graphs using various layouts,
possibly also interactively. There are two entries in the menu.
The first one (Simple) tries to do the plotting automatically;
first it chooses an appropriate layout for the graph and then
tries to guess the graphical parameters to make the plot look
good. Finally it creates a non-interactive plot.

The advanced plotting lets you choose various graphical
parameters, and you also have the possibility to create an
interactive plot.

### Centrality menu

Lets you calculate various degree centrality measures, plus edge
betweenness. The results are always shown in a table that can be
sorted according to all of its columns and the data can also be
exported into a text file.

### Distances menu

Various measures related to path lengths in the network are
included in this menu.

### Subgraphs menu

This menu contains three slightly related entries. Components
are maximal connected subgraphs of a graph. Communities are
natural modules in the graph, a module is a subgraph that has
more edges within the module than between the module and the
rest of the graph. (Loosely speaking.) In the 'Communities' menu
you can run the Spinglass algorithm by J Reichardt and S
Bornholdt. Cohesion measures how difficult it is to disconnect a
graph by removing vertices from it. The last menu entry
calculates cohesion for all components in the selected graph.

### Motifs menu

Motifs are small subgraphs with a given structure. The first
menu entry in this menu just plots all possible motifs of a
given size in a directed or directed graphs. The second menu
entry finds all the different motifs in the selected graph and
plots all the different motifs annotated with the number of
motifs of that kind found in the graph. It also plots a
histogram for the various motifs.

### Help menu

This is what you are reading right now.

### Quit

Not really a menu, just a button. Lets you quit from tkigraph.
